# Supplementary material for: Ten-years cardiovascular risk among Bangladeshi population using non-laboratory-based risk chart of the World Health Organization: Findings from a nationally representative survey
Source: PLoS One. 2021 May 26;16(5):e0251967. doi: 10.1371/journal.pone.0251967 (PMC8153482; doi:10.1371/journal.pone.0251967)
Supplement: S1 Table — (DOCX) [file pone.0251967.s001.docx]

**Supplementary Table 1: Complete case analysis: Univariable and multivariable logistic regression results with potential determinants of elevated CVD risk ≥10% (For males)**

| Variables | Univariable Logistic Regression | | | Multivariable Logistic Regression | | |
| --- | --- | --- | --- | --- | --- | --- |
|  | COR | 95% CI | P value | AOR | 95% CI | P-value |
| Place of residence |  |  |  |  |  |  |
| Rural | Ref |  |  | Ref |  |  |
| Non-slum urban | 1.03 | 0.86, 1.23 | 0.766 | NA | NA | NA |
| Slum | 1.07 | 0.86, 1.33 | 0.544 | NA | NA | NA |
| Educational status |  |  |  |  |  |  |
| No formal education | Ref |  |  | Ref |  |  |
| 1-5 years | 0.95 | 0.78, 1.17 | 0.653 | 0.89 | 0.72, 1.11 | 0.308 |
| 6-10 years | 0.88 | 0.75, 1.04 | 0.131 | 0.76 | 0.64, 0.92 | 0.004 |
| >10 years | 0.88 | 0.73, 1.07 | 0.213 | 0.65 | 0.52, 0.83 | <0.001 |
| Household income |  |  |  |  |  |  |
| Lowest (Q1) | Ref |  |  | Ref |  |  |
| Lower (Q2) | 1.19 | 0.96, 1.47 | 0.109 | 1.16 | 0.93, 1.45 | 0.189 |
| Middle (Q3) | 1.04 | 0.85, 1.28 | 0.675 | 1.09 | 0.88, 1.36 | 0.419 |
| Higher (Q4) | 1.37 | 1.10, 1.72 | 0.005 | 1.34 | 1.05, 1.7 | 0.017 |
| Highest(Q5) | 1.36 | 1.10, 1.68 | 0.004 | 1.27 | 1.00, 1.62 | 0.046 |
| Marital Status |  |  |  |  |  |  |
| Currently married | Ref |  |  | Ref |  |  |
| Others^£^ | 3.06 | 2.14, 4.36 | <0.001 | 2.76 | 1.88, 4.03 | <0.001 |
| Religion |  |  |  |  |  |  |
| Muslim | Ref |  |  | Ref |  |  |
| Others^££^ | 1.09 | 0.91, 1.31 | 0.359 | NA | NA | NA |
| Physical Activity |  |  |  |  |  |  |
| >=150 Minutes/week | Ref |  |  | Ref |  |  |
| <150 Minutes/week | 1.88 | 1.63, 2.16 | <0.001 | 1.84 | 1.55, 2.17 | <0.001 |
| Sedentary time per day |  |  |  |  |  |  |
| 0 to 240 minutes | Ref |  |  | Ref |  |  |
| 241 to 360 minutes | 1.47 | 1.24, 1.74 | <0.001 | 1.36 | 1.14, 1.61 | 0.001 |
| >360 minutes | 1.47 | 1.24, 1.74 | <0.001 | 1.19 | 0.99, 1.43 | 0.058 |
| Fruits & Vegetables Consumption |  |  |  |  |  |  |
| >=5 servings/day | Ref |  |  | Ref |  |  |
| <5 servings/day | 1.21 | 1.00, 1.46 | 0.045 | 1.11 | 0.91, 1.35 | 0.310 |
| Current smokeless tobacco user |  |  |  |  |  |  |
| No | Ref |  |  | Ref |  |  |
| Yes | 1.19 | 1.04, 1.36 | 0.014 | 1.27 | 1.10, 1.47 | 0.001 |
| Self-reported diabetes |  |  |  |  |  |  |
| No | Ref |  |  | Ref |  |  |
| Yes | 1.64 | 1.30, 2.07 | <0.001 | 1.48 | 1.13, 1.92 | 0.004 |
| Waist Circumference |  |  |  |  |  |  |
| Male: <90 cm/ Female: <80 cm | Ref |  |  | Ref |  |  |
| Male: >= 90 cm/ Female: >=80 cm | 1.28 | 1.10, 1.49 | 0.002 | 1.17 | 0.98, 1.39 | 0.087 |

^£^Never married, widows, divorced and separated

^££^Hindu, Christian, Buddhist and others except Muslims

CI: Confidence Interval; COR: Crude Odds Ratio; AOR: Adjusted Odds Ratio; Ref: Reference category

NA: Not applicable, these variables were not included in the adjusted analysis as these were dropped due to significance level was >0.2 in the crude analysis
