# Supplementary material for: Ten-years cardiovascular risk among Bangladeshi population using non-laboratory-based risk chart of the World Health Organization: Findings from a nationally representative survey
Source: PLoS One. 2021 May 26;16(5):e0251967. doi: 10.1371/journal.pone.0251967 (PMC8153482; doi:10.1371/journal.pone.0251967)
Supplement: S2 Table — (DOCX) [file pone.0251967.s002.docx]

**Supplementary Table 2: Complete case analysis: Univariable and multivariable logistic regression results with potential determinants of elevated CVD risk ≥10% (For females)**

| Variables | Univariable Logistic Regression | | | Multivariable Logistic Regression | | |
| --- | --- | --- | --- | --- | --- | --- |
|  | COR | 95% CI | P value | AOR | 95% CI | P-value |
| Place of residence |  |  |  |  |  |  |
| Rural | Ref |  |  | Ref |  |  |
| Non-slum urban | 1.38 | 1.11, 1.71 | 0.004 | 1.31 | 0.97, 1.76 | 0.074 |
| Slum | 0.98 | 0.75, 1.29 | 0.895 | 0.68 | 0.50, 0.92 | 0.014 |
| Educational status |  |  |  |  |  |  |
| No formal education | Ref |  |  | Ref |  |  |
| 1-5 years | 0.57 | 0.43, 0.77 | <0.001 | 0.70 | 0.51, 0.98 | 0.036 |
| 6-10 years | 0.57 | 0.44, 0.73 | <0.001 | 0.57 | 0.42, 0.77 | <0.001 |
| >10 years | 0.29 | 0.17, 0.5 | <0.001 | 0.34 | 0.19, 0.64 | 0.001 |
| Household income |  |  |  |  |  |  |
| Lowest (Q1) | Ref |  |  | Ref |  |  |
| Lower (Q2) | 1.23 | 0.95, 1.58 | 0.116 | 1.16 | 0.87, 1.54 | 0.319 |
| Middle (Q3) | 1.27 | 0.99, 1.62 | 0.057 | 1.21 | 0.92, 1.59 | 0.179 |
| Higher (Q4) | 1.00 | 0.75, 1.35 | 0.975 | 1.05 | 0.75, 1.46 | 0.788 |
| Highest(Q5) | 1.17 | 0.91, 1.51 | 0.229 | 1.23 | 0.90, 1.67 | 0.196 |
| Marital Status |  |  |  |  |  |  |
| Currently married | Ref |  |  | Ref |  |  |
| Others | 5.04 | 4.18, 6.09 | <0.001 | 3.64 | 2.96, 4.48 | <0.001 |
| Religion |  |  |  |  |  |  |
| Muslim | Ref |  |  | Ref |  |  |
| Others | 1.29 | 1.03, 1.62 | 0.026 | 1.58 | 1.22, 2.04 | <0.001 |
| Physical Activity |  |  |  |  |  |  |
| >=150 Minutes/week | Ref |  |  | Ref |  |  |
| <150 Minutes/week | 3.06 | 2.56, 3.65 | <0.001 | 2.49 | 2.02, 3.08 | <0.001 |
| Sedentary time per day |  |  |  |  |  |  |
| 0 to 240 minutes | Ref |  |  | Ref |  |  |
| 241 to 360 minutes | 1.38 | 1.10, 1.71 | 0.004 | 1.16 | 0.92, 1.47 | 0.207 |
| >360 minutes | 1.92 | 1.54, 2.4 | <0.001 | 1.27 | 1.00, 1.63 | 0.053 |
| Fruits & Vegetables Consumption |  |  |  |  |  |  |
| >=5 servings/day | Ref |  |  | Ref |  |  |
| <5 servings/day | 1.48 | 1.06, 2.05 | 0.020 | 1.03 | 0.72, 1.47 | 0.887 |
| Current smokeless tobacco user |  |  |  |  |  |  |
| No | Ref |  |  | Ref |  |  |
| Yes | 1.36 | 1.14, 1.61 | <0.001 | 1.25 | 1.03, 1.53 | 0.025 |
| Self-reported diabetes |  |  |  |  |  |  |
| No | Ref |  |  | Ref |  |  |
| Yes | 1.51 | 1.19, 1.91 | 0.001 | 1.45 | 1.09, 1.92 | 0.010 |
| Waist Circumference |  |  |  |  |  |  |
| Male: <90 cm/ Female: <80 cm | Ref |  |  | Ref |  |  |
| Male: >= 90 cm/ Female: >=80 cm | 1.06 | 0.89, 1.25 | 0.520 | NA | NA | NA |

^£^Never married, widows, divorced and separated

^££^Hindu, Christian, Buddhist and others except Muslims

CI: Confidence Interval; COR: Crude Odds Ratio; AOR: Adjusted Odds Ratio; Ref: Reference category

NA: Not applicable, these variables were not included in the adjusted analysis as these were dropped due to significance level was >0.2 in the crude analysis
