# Supplementary material for: Ten-years cardiovascular risk among Bangladeshi population using non-laboratory-based risk chart of the World Health Organization: Findings from a nationally representative survey
Source: PLoS One. 2021 May 26;16(5):e0251967. doi: 10.1371/journal.pone.0251967 (PMC8153482; doi:10.1371/journal.pone.0251967)
Supplement: S1 File — (PDF) [file pone.0251967.s004.pdf]

1.1.1.1.1.1Questionnaire for the Selection of HH member

Section 1: Information about the interview

No.	Variable	Question	Response	Code	Instruction
101	HS_101	Interviewers Name and ID	|____|____|	Write down your Full Name and Id	
102	HS_102	Cluster Name and Code	______________________ |____|____|	Filled out before starting Interview	
104	HS_104	Union name and code	_______ _______________ |____|____|		
106	HS_106	Upazilla name and code	______________________ |____|____|		
108	HS_108	District name and code	______________________ |____|____|		
110	HS_110	Division name and code	______________________ |____|____|		
112	HS_112	Household Code	|____|____|____|____|____|		
113	HS_113	Total member of Household	|____|____|		
114	HS_114	HH Head Name	______________________		
	HS_114A	HH head Father/Husband Name(Nick Name)	______________________		
104	HS_104A	House name	______________________		
	HS_104B	Village/Para Name	______________________		
115	HS_115A	How many member of your HH/Family	|____|____|	Exclude the HH member who are staying outside for a long time	
			A. Total	B. Sex	
	HS_115B	0-5 Years Child	|____|		
	HS_115BA	Namet______________________		1 = Male<br>2 = Female	
	HS_115BB	Namet ______________________		1 = Male<br>2 = Female	
	HS_115BC	Namet ______________________		1 = Male<br>2 = Female	
	HS_115C	6-9 years of age child	|____|		
	HS_115CA	Namet ______________________		1 = Male<br>2 = Female	
	HS_115CB	Namet ______________________		1 = Male<br>2 = Female	
	HS_115CC	Namet ______________________		1 = Male<br>2 = Female	
	HS_115D	10-19 years Adolecent Boys and Girls	|____|		
	HS_115DA	Namet ______________________		1 = Male<br>2 = Female	
	HS_115DB	Namet ______________________		1 = Male<br>2 = Female	
	HS_115DC	Namet ______________________		1 = Male<br>2 = Female	
	HS_115E	20-59 years of age Male/Female	|____|		
	HS_115EA	Namet ______________________		1 = Male	

No.	Variable	Question	Response		Code	Instruction
				2 = Female		
	HS_115EB	Namet ______________________		1 = Male<br>2 = Female		
	HS_115EC	Namet ______________________		1 = Male<br>2 = Female		
	HS_115F	60 or more than 60 years Male/Female	|___|			
	HS_115FA	Namet ______________________		1 = Male<br>2 = Female		
	HS_115FB	Namet ______________________		1 = Male<br>2 = Female		
	HS_115FC	Namet ______________________		1 = Male<br>2 = Female		
116	HS_116	Household Head Contact No	|___|___|___|___|___|___|___|___|___|___|___|			
117	HS_117	Household Head Contact Alternative No	|___|___|___|___|___|___|___|___|___|___|___|			
118	HS_118	Do you need reinterview ?		1 = Yes 2 = No		If No,go to next section
119	HS_119	Cause of reinterview	1 = Absent= Incomplete Interview<br>99 =Others (please specify)_______			

Module-1:General Information

No	Variable	Question	Response		Code	Instruction
101	GI_101	Interviewers Name and ID	|___|___|			Write down Full Name and Code
102	GI_102	Date of Interview	|___|___|/|___|___|/20|___|___| Day / Month / Year			Fill out the section from scheduler
103	GI_103	HH Number	|___|___|___|___|___|			
104	GI_104	Information would be taken from Module ?	A= Module 2 : HH Information	1 = Yes<br>2 = No		
			B= Module 3 : Child Information	1 = Yes<br>2 = No		
			C= Module 4 : Adolocent Boy	1 = Yes<br>2 = No		
			D= Module 5 : Adolocent Girl	1 = Yes<br>2 = No		
			E= Module 6 : Adult Male	1 = Yes<br>2 = No		
			F= Module 7 : Adult Female	1 = Yes<br>2 = No		
			G = Module 8: Elderly	1 = Yes<br>2 = No		
105	GI_105	Interview Start Time	|___|___|: |___|___| Hour : Minute			Use 24 Hr Formate

Module 2: Household Head Questionnaire

Section:1: General Information and HH Socioeconomic Status(SES)

No.	Variable	Question	Responses and code categories	Code	Instructions/ Skip
101	SE_101	Interviewer's name and ID	|____|____|		Write your full name and code
102	SE_102	Name of the household head (with nick name)			
103	SE_103	Sex of the household head	1 = Male<br>2 = Female		
104	SE_104	Date of birth of household head	|____|____|/|____|____|/20|____|___| Day / month / year		
105	SE_105	Age of household head (full year)	|____|____|____|year		
106	SE_106	Total years of institutional education, you have completed?<br>(Write in completed years| Not acceptable below the class one and preschool educations are not accepted.)<br>*Instruction:Write down,Total years of institutional education,respondent has completed.If not completed any institutional education,write 00*	|____|____|Year<br>Primary education/ebtedayee=5<br>Secondary/Dakhil=10<br>Higher Secondary/Diploma/Alim=12<br>Graduete/Fazil=16<br>Post graduate/kamil/dawra=18		
107	SE_107	What is your main occupation from last 12 month?<br>**Instruction:**<br>If the respondent involved more than one profession since last 12 months, then consider the main occupation which he spent most of the time and write down the answer code.	1 = Agricultural work(paddy)<br>2 = Agricultural work(without paddy)<br>3 = Agricultural day labor<br>4 = Unskilled day labor<br>5 = Skilled day labor<br>6= Rickshaw puller/ van/Wheel barrow/Baby taxi/ Boatman<br>7=Fisherman<br>8=Employer<br>9=Professionals<br>10=Businessman<br>11= Petty businessman<br>12= House maid<br>13=Zoom fermar<br>14= Do not earn<br>15=Poultry/dairy fermar<br>16=Handicrafts<br>17 =Spinach vegitable<br>18=Fishery<br>19 = Homemaker<br>20= Student<br>77 = Don,t know<br>66 =Age below 06 years<br>99 = Others(specify bellow)<br>...................................		

No.	Variable	Question	Responses and code categories	Code	Instructions/ Skip
108	SE_108	Amount of Own HH land	|____|____|____|____| decimal		
109	SE_109	Amount of own cultivating land	|____|____|____|____| decimal		
110	SE_110	What type of fuel did you usually use for cooking?	1= Electricity<br>2= LPG<br>3= Piped Natural gas<br>4= Kerosene<br>5= Coal/Lignate<br>6= Charcoal<br>7= Wood<br>8= Straw/grass/agri waste/leaves<br>9= Animal dung<br>10 = Bio-gas<br>99 = Others (Specify)____		

Do you have livestock or poultry currently at your household? If yes,how many you have?

No.	Variable		A. 1 = Yes 2 = No	B. Number (If Yes)		
111	SE_111A	Cow/ Buffalo	1= Yes 2 = No	|____|____|____|		
	SE_111B	Sheep/Goat/pig	1 = Yes 2 = No	|____|____|____|		
	SE_111C	Chickens/ Duck/Geese	1 = Yes 2 = No	|____|____|____|		
	SE_111D	Small game (rabbits, pigeon etc)	1 = Yes 2 = No	|____|____|____|		
	SE_111E	Others (Specify)_____	1 = Yes 2 = No	|____|____|____|		

Which of the following items are currently active at your household?

No.	Variable	Item	Responses	Code	Instructions/ Skip
112	SE_112A	Electricity	1= Yes 2= No		
	SE_112B	Solar Electricity	1= Yes 2= No		
	SE_112C	Radio/ Television	1= Yes 2= No		
	SE_112D	Telephone/Mobile telephone	1= Yes 2= No		
	SE_112E	Electric fan	1= Yes 2= No		
	SE_112F	Bedstead	1= Yes 2= No		
	SE_112G	Almirah/wardrobe	1= Yes 2= No		
	SE_112H	Refrigerator	1= Yes 2= No		
	SE_112I	Table/chair	1= Yes 2= No		
	SE_112J	Watch/ wall clock	1= Yes 2= No		
	SE_112K	Bicycle	1= Yes 2= No		
	SE_112L	Motorcycle / motor scooter/tempu	1= Yes 2= No		
	SE_112M	Animal powered cars	1= Yes 2= No		
	SE_112N	Car/truck	1= Yes 2= No		
	SE_112O	Boat	1= Yes 2= No		
	SE_112P	Ingine powered boats?	1= Yes 2= No		
	SE_112Q	Rickshaw/ van	1= Yes 2= No		
	SE_112R	Power tiler	1= Yes 2= No		
	SE_112S	Water pump	1= Yes 2= No		
	SE_112T	Plow	1= Yes 2= No		
	SE_112U	IPS/generator ?	1= Yes 2= No		
	SE_112V	Net for fishing	1= Yes 2= No		

No.	Variable	Question	Responses and code categories	Code	Instructions/ Skip
113	SE_113	What is the main construction material used for the outer wall of respondent's house?<br><br>(Record based on your own observation)	**Natural walls**<br>1= No walls<br>2= Cane/ Palm/ Trunk<br>3= Dirt<br><br>**Rudimentary walls**<br>4= Bamboo with mud<br>5= Stone with mud<br>6= Playwood<br>7 = Cardboard/polythene<br><br>**Finished walls**<br>8= Tin<br>9=Bricks/ Cement<br>10= Stone with lime/ Cement<br>11= Wood planks/ Shingles<br>99 = Other (Specify)______		(Record answer after observation)
114	SE_114	What is the main construction material used for the roof of respondent's house?<br><br>(Record based on your own observation)	**Natural Roofing**<br>1= No roof<br>2= Thatch/ Palm leaf<br><br>**Rudimentary roofing**<br>3= Bamboo/Mud<br>4= Wood Planks<br>5 = Cardboard/ polythene<br><br>**Finished roofing**<br>6= Tin<br>7= Wood<br>8= Ceramic Tiles<br>9=Bricks/ Cement<br>10=Wooden blade<br>99 = Other (Specify)___		
115	SE_115	What is the main construction material used for the floor of respondent's house?<br><br>(Record based on your own observation)	**Natural Floor**<br>1= Earth/Sand<br><br>**Rudimentary Floor**<br>2= Wood Planks<br>3= Palm/ Bamboo<br><br>**Finished Floor**<br>4= Ceramic Tiles<br>5=Bricks/ Cement<br>6=Mojaic<br>99 = Others (Specify)______		
116	SE_116	How many living rooms does your household have (Including homemaid room)	|_____|_____|		
117	SE_117	At present, any member of your household has been taking any advantage from the specified government program?<br>((May be multiple answers,if coded "food for work"then justify,is it running government program?)	1 =Get nothing<br>2 =Money for education<br>3 =Freedom fighter allowance<br>4 =Elderly allowance<br>5 =V.G.D<br>6 =Widow allowance<br>7 = 100 days program(money for work,current program)<br>8 =V.G.F<br>9 =National service program<br>77 =Don,t know<br>99 Others (specify bellow) __________		
118	SE_118	Religion<br>*Instruction:*<br>Write down the religions of respondant	1 = Islam 2= Hindu<br>3 = Christian 4 = Buddhist<br>99 = Others (Specify)__________		
119	SE_119	What is your ethnicity?	|____|____|		
**Code**1 =Chakma; 2 =Marma; 3 =Tripurav; 4 =Murong; 5 =Toncongav; 6 =Bom; 7 =Pankho; 8 =Chak; 9 =Khiyangs; 10 =Khumi;11 =Lusai; 12 =Koch; 13 =Saowtal; 14 =Daluy; 15 =Lusai; 16 =Rakhain; 17 =Monipuri; 18 =Garow; 19 =Hajong; 20 =Khasiyav; 21 =Mongs; 22 =Oraow; 23 =Bormon; 24 =Pahari; 25 =Mal pahari; 26 =Munda; 27 =Khol; 28 =Bangali; 99 = Others (Specify)__________					
120	SE_120	Did you receive money from anyone during last 6 month who are not in your HH (who	1 = No<br>2 = Yes<br>77 = Dont Know		If answer No then go to 122

No.	Variable	Question	Responses and code categories	Code	Instructions/ Skip
		are from abroad or other area)			
121	SE_121	On average, How much amount you have received per month ? (Considering last 6 month)	|____|____|____|____|____|____| Taka		
122	SE_122	What is your average monthly household income ?	|___|___|___|___|___|___|___| Taka<br>88 = Disagreed		

Section 2: Household water, sanitation and Hygiene

No.	Variable	Question	Response		Code	Instruction
201	WH_201	What is the main source of drinking water of your household?	**Piped Water**<br>1= Piped to dwelling<br>2= Piped to yard/plot<br>3= Public tap<br><br>**Tube Well or Borehole**<br>4= Shared<br>5 = Household	**Dug Well**<br>6= Protected<br>7= Unprotected<br>8= Rainwater<br>**Groud water**<br>9= Surface water (Pond/ River/ Canal/Hawar/ irrigation channels)<br>10= Water tanker<br>**Spring**<br>11 = Protected<br>12 = Unprotected<br>99 = Others (Specify)______		
202	WH_202	Do you have any soap in your household?	1= Yes ; 2= No			
203	WH_203	Did you use soap within the last 24 hours?	1= Yes ; 2= No			If No, go to 205 question.
204	WH_204	If you used soap today or yesterday,then why you used soap?<br><br>*If the respondent answered, washed hand myself or* my child then try to know other purpose of use, just probe it don't read out the answer code. (Multiple answers can be coded, Do not read out the answers code, ask "what else" until get right answer.)	1= Washing cloths	1= Yes<br>2= No		
			2= Washing my body	1= Yes<br>2= No		
			3= Washing my children	1= Yes<br>2= No		
			4= Washing child's bottoms	1= Yes<br>2= No		
			5= Washing my children's hands	1= Yes<br>2= No		
			6= Washing hands after defecating	1= Yes<br>2= No		
			7= Washing hands after cleaning child	1= Yes<br>2= No		
			8= Washing hands before feeding child	1= Yes<br>2= No		
			9= Washing hands before preparing food	1= Yes<br>2= No		
			10= Washing hands before eating	1= Yes<br>2= No		
			11= Others (Specify)______	1= Yes<br>2= No		
205	WH_205	What type of toilet used by your family members?<br><br>(Ensured after your own observation)	1= Piped sewer system<br>2= Septic tank<br>3= Ring slab with water seal<br>4= Ring slab without water seal<br>5= Pit latrine with slab<br>6= Pit latrine without slab<br>7= Hanging latrine<br>8= No facility (Bush/open field/river pond side)<br>99= Others (Specify)_______			If Answer '8' ,go to WH_207
206	WH_206	Is your toilet shared or a public toilet?<br><br>*(Ask regardless of location. A **shared** toilet is a joint asset between a set numbers of household.*<br>***Public** means the number of people sharing is not precisely know...i.e a community toilet)*	1= Not shared<br>2= Shared<br>3= Communal<br>4= Don't know			

No.	Variable	Question	Response	Code	Instruction
207	WH_207	Do you go to the toilet wearing shoes?	1= Yes ; 2= No		
208	WH_208	Could you show me where you usually wash your hands?<br><br>*(Ask and observe)*	0= Modern piped facilities in dwelling<br>1= Inside/near toilet facility<br>2= Inside/near kitchen/cooking place<br>3= Elsewhere in yard<br>4=Inside yard<br>5= Outside yard<br>6= No specific place<br>8= No permission to see		If Answer 6/8,then go to section 3A .
209	WH_209	Is there any soap or detergent or locally used cleansing agent? Circle the answer code after your observation.<br><br>This item should be either in place or brought by the interviewee within one minute then code "yes". If the item is not present within one minute check none, even if brought out later then code "5".	1= Soap<br>2= Detergent<br>3= Ash<br>4= Mud/sand<br>5= None<br>99= Other (Specify)_______		
210	WH_210	Is there water? Circle the answer code after your observation.<br>Interviewer: turn on tap and/or a check container and note if water is present. This item should be either in place or brought by the interviewee within one minute.<br>If the item is not present within one minute check no, even if brought out later.	1= Yes ; 2= No		
211	WH_211	Are there any hand washing materials such as a tap, basin, bucket, sink, or tippy tap? Please circle the answer code based on your observation.<br>This item should be either in place or brought by the interviewee within one minute.<br><br>If the item is not present within one minute check no, even if brought out later.	1= Yes ; 2= No		

Section 3A: Household Food Security and Coping Strategies (Last one month)

Now I would like to take a few minutes of your time to ask you about food security in your household. I am going to ask you to think back over the past four weeks and try to remember how often and in what ways your household experienced food insecurity. Please note that whenever I say "household" I mean a group of people who usually share food from the same cooking pots. If you have any questions at any time, please feel free to stop me and ask.

No.	Variable	Question	Response	Code	Instruction
301	FS_301	In the past four weeks, did you ever worry that your family would not have enough food due to lack of resources or money to buy food?	0= Never<br>1= Rarely (Once or twice)<br>2= Sometimes (Three or ten times)<br>3= Often (More than ten times)		
302	FS_302	In the past four weeks, were you or any family members ever not able to eat the kinds of foods you preferred because of a lack of resources or money to buy food?	0= Never<br>1= Rarely (Once or twice)<br>2= Sometimes (Three or ten times)<br>3= Often (More than ten times)		"Preferred foods" might be things like *polao, biriyani,* fish, meat, yoghurt, sweets or anything your household prefers to eat.
303	FS_303	In the past four weeks, did you or any household member ever have to eat a limited variety of foods due to lack of resources?(e.g:onion,garlic	0= Never<br>1= Rarely (Once or twice)<br>2= Sometimes (Three or ten times)<br>3= Often (More than ten times)		
304	FS_304	In the last 4 weeks did you or any member of your family Had to eat any food that usually did not eat (such as wild/non food/ instead of rice any other food that usually does not eat) ?	0= Never<br>1= Rarely (Once or twice)<br>2= Sometimes (Three or ten times)<br>3= Often (More than ten times)		
305	FS_305	In the past four weeks, did you or any household member ever have to eat a smaller meal at breakfast, lunch, or dinner than you felt you needed because there was not enough food for your family?	0= Never<br>1= Rarely (Once or twice)<br>2= Sometimes (Three or ten times)<br>3= Often (More than ten times)		
306	FS_306	In the past four weeks, did you or any other household member ever have to eat fewer than three meals in a day because there were not enough resources or money to purchase food?	0= Never<br>1= Rarely (Once or twice)<br>2= Sometimes (Three or ten times)<br>3= Often (More than ten times)		
307	FS_307	In the past four weeks, did you or any household member ever go to sleep at night hungry because there was not enough money to purchase food?	0= Never<br>1= Rarely (Once or twice)<br>2= Sometimes (Three or ten times)<br>3= Often (More than ten times)		
308	FS_308	In the past four weeks, did you or any household member ever go to sleep at night hungry because there was not enough money to purchase food?	0= Never<br>1= Rarely (Once or twice)<br>2= Sometimes (Three or ten times)<br>3= Often (More than ten times)		

No.	Variable	Question	Response	Code	Instruction
309	FS_309	In the past four weeks, did you or any household member ever go a whole day and night without eating anything because there was not enough money to purchase food?	0= Never<br>1= Rarely (Once or twice)<br>2= Sometimes (Three or ten times)<br>3= Often (More than ten times)		If answer 0 then go to 310.
310	FS_310	Did you (household) have to do the following activities to overcome food shortage during the last 4 weeks??	A. Sale/ Mortgage if assets — 1= Yes, 2 = No		
			B. Eat low quality food — 1= Yes, 2 = No		
			C. Eat less items of foods — 1= Yes, 2 = No		
			D. Stop schooling of HH members — 1= Yes, 2 = No		
			E. Borrow food — 1= Yes, 2 = No		
			F. Take loan — 1= Yes, 2 = No		

Section 4: Household consumption of cooking oil and Salt

No.	Variable	Question	Response	Code	Instruction
401	OS_401	What type of cooking oil do you use in your household?	1=Soybean<br>2=Mustard<br>3= Supper/Palm<br>4= Coconut<br>5=Sunflower oil6=canola oil<br>7= Rice brand oil<br>99= Others (Specify)____		
402	OS_402	Which kind of cooking oil did you buy?	1= Brand oil<br>2= Open oil<br>3= Both<br>99= Others (Specify)____		If "2" go to 406
	OS_402A	Which brand of cooking oil did you buy for household cooking?	1= Teer<br>2= Fresh<br>3= Rupchanda<br>99= Others (Specify)____		
403	OS_403	Which brand you used for cooking, Is it Vitamin A" fortified?	1 = Yes 2= No<br>77 =Don't know		Please check for the labeling on HH oil container
404	OS_404	Which brand you used for cooking,Is it Vitamin D" fortified?	1 = Yes 2= No<br>77 =Don't know		Please check for the labeling on HH oil container
405	OS_405	Which brand you used for cooking,Is it Vitamin E" fortified?	1 = Yes 2= No<br>77 =Don't know		Please check for the labeling on HH oil container
406	OS_406	Cooking oil sample collected	1 = Yes 2= No<br>77 =Don't know		If answer 2 then go to 408
407	OS_407	Sample label/ID			
408	OS_408 A/B/C	What type of salt used in your household?<br>(Read the following type and answer Yes or No Code)	Open salt: 1 = Yes 2= No		If answer for Open salt is 'No' then go to 410
			Packing salt (Without brand): 1 = Yes 2= No		
			Packing salt (Brand): 1 = Yes 2= No		
409	OS_409	Which brand used in your household.	1 =Molla Salt<br>2 =ACI<br>3 =Brac Salt<br>4 =Tir Salt<br>5= Fresh Salt<br>6=Confidence<br>99=Others(specify ……………………….)		
410	OS_410	How much salt required per week in your household for food and food preparation?	|____|____|____|____| gm		

No.	Variable	Question	Response	Code	Instruction
411	OS_411	We want to test your household salt for iodine presence status.Can I get some salt from you which you used for your household food last night[To see the result after test then circle code]	1 = No Iodine presence(0 ppm)<br>2 = less than 15 PPM<br>3 = 15 or more<br>4 =less than 25 ppm<br>5 = less than 50<br>6 =less than 75ppm<br>7 =100 or more Zvi †ekx<br>8 = Not testified		

মডিউল ৩: শিশুর (০-৫ বছর) প্রশ্নপত্র

পরিচ্ছেদ ১ঃসাধারণ তথ্যাবলী

No.	Variable	Question	Response	Code	Instruction
101	CI_101	Child Name (with nick name)	______________________		
102	CI_102	Child Date of birth	|____|____|/|____|____|/20|____|____| DD / MM / YYYY		
103	CI_103	Age of child (Write completed years of age)	A. |____|____|Year<br>B. |____|____|Month		
104	CI_104	Sex of the child	1 = Male 2= Female		
105	CI_105	Completed years of education of Child Mother<br>***[Instruction:** Write down in total, the years the respondent spent in school for formal education (Is completed years) [Excluding pre-school and informal education). Write down "00" if no formal education.]*	|__|__| Years<br>• Primary/Ebtedayee=5<br>• Secondary/Dakhil=10<br>• Highersecondary/Diploma/Alim =12<br>• Graduate/Fazil=16<br>• Post graduate=18		
106	CI_106	Main occupation of Child Mother during last 12 month?<br>**Instruction:**<br>If the respondent involved more than one profession since last 12 months, then consider the main occupation which she spent most of the time and write down the answer code.	1 = Agricultural work(paddy)<br>2 = Agricultural work(without paddy)<br>3 = Agricultural day labor<br>4 = Unskilled day labor<br>5 = Skilled day labor<br>6= Rickshaw puller/ van/Wheel barrow/Baby taxi/ Boatman<br>7=Fisherman<br>8=Employer<br>9=Professionals<br>10=Businessman<br>11= Petty businessman<br>12= House maid<br>13=Zoom fermar<br>14= Do not earn<br>15=Poultry/dairy fermar<br>16=Handicrafts<br>17 =Spinach vegitable<br>18=Fishery<br>19 = Housewife<br>20= Student<br>77 = Don,t know<br>66 =Age below 06 years<br>99 = Others(specify bellow)<br>...................................		
107	CI_107	Completed years of education of Child (Child name) Father<br>***[Instruction:** Write down in total, the years the respondent spent in school for formal education (Is completed years) [Excluding pre-school and informal education). Write down "00" if no formal education.]*	|__|__| Years<br>• Primary/Ebtedayee=5<br>• Secondary/Dakhil=10<br>• Highersecondary/Diploma/Alim= 12<br>• Graduate/Fazil=16<br>• Post graduate=18		

No.	Variable	Question	Response	Code	Instruction
108	CI_108	Main occupation of Child Father during last 12 month?	1 = Agricultural work(paddy)<br>2 = Agricultural work(without paddy)<br>3 = Agricultural day labor<br>4 = Unskilled day labor<br>5 = Skilled day labor<br>6= Rickshaw puller/ van/Wheel barrow/Baby taxi/ Boatman<br>7=Fisherman<br>8=Employer<br>9=Professionals<br>10=Businessman<br>11= Petty businessman<br>12= House maid<br>13=Zoom fermar<br>14= Do not earn<br>15=Poultry/dairy fermar<br>16=Handicrafts<br>17 =Spinach vegitable<br>18=Fishery<br>19 = Housewife<br>20= Student<br>77 = Don,t know<br>66 =Age below 06 years<br>99 = Others(specify bellow)<br>....................................		

Section 2: Infant and Young Child Feeding

No.	Variable	Question	Response	Code	Instruction
201	IF_201	Mention Child Age group?	1=< 6 months<br>2 = <24 months<br>3 = Over 24 months		If answer"3" go to section 3
202	IF_202	Have you ever breastfed your child?	1= Yes 2= No		
203	IF_203	First time after birth, When you gave breastmilk to your infant,?<br><br>If respondent reports she gave brestmilk to the infant immediately after birth, circle "0" For 'Immediately'. AND RECORD "00"<br>If less than 1 hour, circle "1" for hours AND RECORD "00" hours.If less than 24 hours, circle "1" and record number of completed hours, from 01 to 23.Otherwise, circle '2" and record number of completed days.	0 = Immediately<br>**Or**<br>1 = |____|____| Hours<br>**Or**<br>2= |____|____| Days		
204	IF_204	Did you give colostrums to your baby?	1= Yes<br>2 = No<br>77= Don't know		
205	IF_205	Did you give any food or drink into (name's) mouth before the first breastmilk?	1= Yes<br>2 = No<br>77= Don't know		
206	IF_206	Did you give any food or drink into (name's) mouth within 6 months after birth? (Excluding breastfeeding)	1 = Yes<br>2 = No<br>8 = Don't know		If answer "2" go to 208

				A.1=Yes,2=No	B.Number if 'Yes'	A 1=Yes 2=No
	IF_207A	*Did your child (Name) take any of the following food/drinks yesterday during the day and night?*	Breastmilk	1 = Yes 2 = No	|____|____|	
	IF_207B		Only water	1 = Yes 2 = No	|____|____|	
	IF_207C		Sugar water	1 = Yes 2 = No	|____|____|	
	IF_207D		Infant formula (ie. Serelac,lactogen)	1 = Yes 2 = No	|____|____|	
	IF_207E		Tinted, powder or fresh animal milk(cow, goat, Sheep, Buffalo)	1 = Yes 2 = No	|____|____|	
	IF_207F		Juice , Juice drink, green coconut	1 = Yes 2 = No	|____|____|	
	IF_207G		Yogurt	1 = Yes 2 = No	|____|____|	
	IF_207H		ORS	1 = Yes 2 = No	|____|____|	
	IF_207I		Thin soup	1 = Yes 2 = No	|____|____|	
	IF_207J		ORS Thin sugary	1 = Yes 2 = No	|____|____|	
	IF_207K		Vitamin/drop of medicine/syrup	1 = Yes 2 = No	|____|____|	

No.	Variable	Question	Response	Code	Instruction
208	IF_208	Yesterday, over the day or night did child (name) drink anything from a bottle with a nipple?	1 = Yes 2 = No<br>77= Don't know		

No.	Variable	Question	Response	Code	Instruction
209	IF_209	During the last one month, did you hear/see/read any message/information about Child breast-feeding or supplementary feeding?	1 = Yes 2 = No<br>77= Don't know		
210	IF_210	If yes, where did you hear/see/read the message?	1 =Radio<br>2 =Telivision<br>3= News paper<br>4 =Mobile telephone<br>5 =Doctor/health worker/nurse<br>6 =Poster/liflet/bilboard<br>7 =Neighbour<br>99 =Others(specify bellow)<br>----------------------		
When did you start to give the following foods/drinks to your (< 24 month) baby(if < 1 month/0-29 days; code 0)					
211	IF_211A	Water	|____|____|		
	IF_211B	Liquids without breast milk (Sugar, glucose, tea, fruit juice etc)	|____|____|		
	IF_211C	Cow/goat/buffalo milk	|____|____|		
	IF_211D	Suji, smashed rice	|____|____|		
	IF_211E	Semi solid foods (smashed rice, hochpoch, smashed potato, ripe banana, smashed family foods etc)	|____|____|		
	IF_211F	Solid food (rice, wheat, puffed rice/flattened rice etc)	|____|____|		
	IF_211G	Fish	|____|____|		
	IF_211H	Meat (chicken, beef, mutton etc)	|____|____|		
	IF_211I	Egg	|____|____|		
	IF_211J	Pluses (lentil, peas, peas daletc)	|____|____|		
	IF_211K	Green leafy vegetables	|____|____|		
	IF_211L	Snacks (chanachur,Chips, biscuit, nuts etc)	|____|____|		
	IF_211M	Monimix, Micronutrient powder	|____|____|		
	**Code:**0= Within 30 days (in 0 month); 1= After 1 month; 2= After 2 month; 3= After 3 month; 4= After 4 month; 5= After 5 month; 6= After 6 month; 7= After 7 month; 8 = After 8 month; 9 = After 9 month; 10 = After 10 month; 11 = After 11 month; 12 = After 12 month; 13 = Yetn't started 77 = Don't know/ don't remember				
212	IF_212	Yesterday, during the day or night, how many times did your child (name) eat solid, semi-solid, or soft foods other than liquids at home or outside home (*exclude liquids*)?	Number of times<br>|____|____| 77= Don't know		
213	IF_213	Yesterday, did your child (name) consume containing iron drops or tablets (including sprinkles/monimix)?	1 = Yes 2 = No<br>77 = Don't know		
214	IF_214	From the last 7 days, did your child (name) consume any food with added any nutrient powder (sprinkles/Monimix)?	1 = Yes 2 = No<br>77 = Don't know		
215	IF_215	Did (name) you ever fed your child any food to which you added a nutrient powder (sprin; kles/Monimix)?	1 = Yes 2 = No<br>77 = Don't know		If answer "2" go to section 3
216	IF_216	If yes, then how long times? Please specify	|____|____| w`b		If 201Answer 1,then go to section 4

Section 3: Dietary diversity

No	Variable	Question		Yesterday during the day or night, did your child consume the following food items? (A)	During the last 07 days, how many days did your child consume the following food items (B)	[illegible]
Now I would like to requst describing the food items that you have feed your child(child name) at home and outside of home during the last day o from yesterday morning 6.00 am to today morning 6.00 am) .Please tell me,all types of food, drinks that fed your child (child name) at morning, n dinner, or breakfast / light breakfast. Remembering If you fed any of food during the cooking, please tell us including all.<br><br>Start the interview like, which food give your child at the morning time .<br><br>-What did you feed (child name) in the morning wake up **?** Did you feed anything more**?**<br>-What did you feed more in the morning**?** Did you feed anything more**?**<br>-What did you feed at noon**?** Did you feed anything more**?**<br>-What did you feed at afternoon**?** Did you feed anything more**?**<br>-What did you feed at evening**?** Did you feed anything more**?**<br>-What did you feed at dinner**?** Did you feed anything more**?**<br>**Record first the answer of the questions of column A. collect the answer for column B/C after recording the answer of the questions of A.**						
		Food Category	Food Type	(A)	(B)	
301	CDD_301 A/B/C	Starchy Staples/ Food made from grains	Rice,flour bread, wheat, muri, maize,kichuri,barli,oot,kinoya,noodles,pasta	1 = Yes<br>2 = No	|____|____| days	|_
302	CDD_302 A/B/C	Starchy Staples/ (root,tubers,tissue)	potatoes, sweet potatoes,sagu, erarut,cave,shalgam,kuchu,wiggle,ripe banana shaloo	1 = Yes<br>2 = No	|____|____| days	|_
303	CDD_303 A/B/C	Lentils/Pulses	Dal/khicuri,peas,motor,soybean,tofu peanuts, humas	1 = Yes<br>2 = No	|____|____| days	|_
304	CDD_304 A/B/C	Legumes and Nuts	Peanut, Pistachios, kazu or any kond of peanut, ciya seeds, til, tisi,sunflower seeds,sweet pumpkin seeds	1 = Yes<br>2 = No	|____|____| days	|_
305	CDD_305 A/B/C	Dark green leafy vegetables	All kinds of leafy vegetables (poo,kuchu, kalmi) Brooklyn	1 = Yes<br>2 = No	|____|____| days	|_
306	CDD_306 A/B/C	Red/orange/yellow vegetables	Orange sweet potato,pumpkin, carrot or other yellow or orange vegetable	1 = Yes<br>2 = No	|____|____| days	|_
307	CDD_307 A/B/C	Red/orange/yellow fruits	Ripe mangoes, papaya, jackfruits other red/yellow or orange fruit	1 = Yes<br>2 = No	|____|____| days	|_
308	CDD_308 A/B/C	Vitamin C rich fruits	Guava, Strawberry, Lemon, Orange,grapes, Pineapple,raw mango,amalaki,kueue,tomatos	1 = Yes<br>2 = No	|____|____| days	|_
309	CDD_309 A/B/C	Vitamin C rich vegetables	Raw tomato,chilli pepper, brussels sprouts,cauliflower,cabbage	1 = Yes<br>2 = No	|____|____| days	|_
310	CDD_310 A/B/C	Other vegetables or fruits	peas, asparagas, bit,koci bash, cauliflower,selari,shawsha,brinjal,letus,masrum,mula,jukini	1 = Yes<br>2 = No	|____|____| days	|_
311	CDD_311 A/B/C	Other fruits	Apple, avakado,bam, cherry,ripe jackfruit	1 = Yes<br>2 = No	|____|____| days	|_

No	Variable	Question		Yesterday during the day or night, did your child consume the following food items? (A)	During the last 07 days, how many days did your child consume the following food items (B)	[illegible]
312	CDD_312 A/B/C	Eggs	Hen/duck, other birds,	1 = Yes<br>2 = No	|____|____| days	|_
313	CDD_313 A/B/C	Organ meat	Cheeks, liver, Stomach, Ridpind, Kidney	1 = Yes<br>2 = No	|____|____| days	|_
314	CDD_314 A/B/C	Flesh	Beef, Pork, Veal, Lamb, Goat, Chicken, Duck or flesh of any other animal	1 = Yes<br>2 = No	|____|____| days	|_
315	CDD_315 A/B/C	Small Fish	Small Fish Eaten Whole with Bones (i.e.Kachki, mola, dhela, chapila, batashi, small prawn, dried fish)	1 = Yes<br>2 = No	|____|____| days	|_
316	CDD_316 A/B/C	Large fish/sea food	Large Whole Fish and Shell Fish and dried fish	1 = Yes<br>2 = No	|____|____| days	|_
317	CDD_317 A/B/C	Dairy	Milk, cheese, yogurt or other milk products	1 = Yes<br>2 = No	|____|____| days	|_
318	CDD_318 A/B/C	Insects and other protein foods	Fish egg, insect, snail	1 = Yes<br>2 = No	|____|____| days	|_
319	CDD_319 A/B/C	Edible Oil	Ghee, butter,cream, sour, fat, margarine, mayonnaise, palm oil, vegetable oil,	1 = Yes<br>2 = No	|____|____| days	|_
320	CDD_320 A/B/C	Savory and fried snacks	Crisps and chips, fried dough or other fried snacks singaru, samarcha	1 = Yes<br>2 = No	|____|____| days	|_
321	CDD_321 A/B/C	Sweets	Sugary foods, such as chocolates, candies, cookies/sweet biscuits and cakes, sweet pastries or ice cream, any kind of sweets ,honey ,halua , condensed milk,tiler khaza	1 = Yes<br>2 = No	|____|____| days	|_
322	CDD_322 A/B/C	Miscellaneous (Sweet drinks)	Tea with suger, soft drinks, juice, energy drinks,yogurt drinks,chocolate drinks, horlicks,moltova	1 = Yes<br>2 = No	|____|____| days	|_
323	CDD_323 A/B/C	Other beverages and foods	Tea or coffee if not sweetened, clear broth, alcohol, Pickles, olives and similar	1 = Yes<br>2 = No	|____|____| days	|_
324	CDD_324 A/B/C	Condiments/spices	Spices, coriander leaves, sausage, garlic, ketchup, lemon juice, mint leaves, drinks, betel leaves, tobacco leaves, jars	1 = Yes<br>2 = No	|____|____| days	|_

Section 4: Child Morbidity:
I will ask you some questions about your child illness during the last 2 weeks (If information is collected on Wednesday then tell from the last wendsday to privious wendsday to yesterday)

No.	Variable	Question	Response	Code	Instruction
401	CM_401	Was your child (name) sick in the last 2 weeks/ 14 days?	1 = Yes 2 = No		If answer "2" go to 407
**Has your child (Name) been suffering from any of the following diseases/conditions from last 2 weeks/14 days?**					
402	CM_402A	Fever in the last two weeks(child name)	1 = Yes 2 = No<br>77= don't know		
	CM_402B	Runny Nose/ Cough in last 2 weeks(child name)	1 = Yes 2 = No<br>77= don't know		
	CM_402C	Difficulty breathing in the last 2 weeks (child name)	1 = Yes 2 = No<br>77= don't know		
	CM_402D	Diarrhea in the last 2 weeks (child name)<br>Please use local term of diarrhea if needed.	1 = Yes 2 = No<br>77= don't know		
403	CM_403	In the last 2 weeks/14 days where did you seek treatment/ advice for any of these mentioned illnesses/diseases?	**GOVERNMENT PROVIDERS**<br>1= Govt. Field worker<br>2= Govt. satellite clinic/ EPI Outreach clinic<br>3= Govt. Community clinic<br>4=Govt. Health & Family Welfare Centre<br>5=Govt. Upazila Health Complex<br>6= Govt. Maternal & Child Welfare Centre Government<br>7=Other government Hospital<br>**NGO PROVIDERS**<br>8= NGO health clinic and hospital<br>9=NGO Field Worker<br>10= Community Nutrition Promoter (CNP)<br>**PRIVATE PROVIDERS**<br>11 = Private doctor/clinic/hospital<br>12 = Pharmacy<br>13 = Traditional healer<br>14 = Homeopath<br>15= from none<br>99 = Others (Specify)___________		If not suffered by any disease within 14 days then skip this question
404	CM_404	How much amount of food, did you given to child (Name) when he/she was in sick/ill?<br>Given less than usual/as usuall amount, more than usual? (including breast milk)	1= Less<br>2= About same<br>3= More<br>4= Nothing to eat<br>5= Not yet began feeding<br>77 = Don't know		If no disease with in last 2 weeks,skip this question

No.	Variable	Question	Response	Code	Instruction
405	CM_405	(শিশুর নাম)Amount of drink did you given to child(Name) during diarrhoeal sickness?<br>Given less than usual/as usuall amount, more than usual?<br>(including breast milk)	1= Less than usuall<br>2= As usuall<br>3= More than usuall<br>4= Nothing to eat<br>5= yetn't started feeding<br>77= Don't know		If no occured diarrhea then skip this question
406	CM_406	Did you give (child name) the following liquids/drinks during illness in diarrhea (last 14 days?<br><br>(IF ORS packet was given ask to see it and code if the packet contained zinc)	Yes / No<br>A.Packet saline (ORS): 1 / 2<br>B. Home made saline (Salt molasses / Salt sugar s aline) (লবণ গুড়/ লবণ চিনির স্যালাইন): 1 / 2<br>C.Rice saline(packet): 1 / 2<br>D. Rice saline (home made): 1 / 2<br>E. Zinc syrup/tablet/ORS: 1 / 2		If not diarrhea skip this question
407	CM_407	Did your child(child name) eat Vitamin 'A' Capsule in the last six month?	1=Yes, National VAC day/ VAC campaign<br>2=Yes, other sources (specify)________<br>3=No<br>77= Don't know<br>88= Not applicable (< 6 month)________		
408	CM_408	Did your child (name) received any allopathic deworming agents the last six month?	1=Yes, National VAC day/ VAC campaign<br>2=Yes, other sources (specify)________<br>3=No<br>77= Don't know<br>88= Not applicable (< 6 month)________		
409	CM_409	Was (Child's name) the children suffered from Jaundice in the last six months	1 = Yes 2 = No<br>77= don't know		
410	CM_410	Is any your child identified (06 to 59 months) as SAM/MAM?	1= Yes (with card)<br>2= Yes, (without card)<br>3=No<br>77= Don't remember<br>88=Not applicable(< 6 month)		If 'no' or 8 go to next section
411	CM_411	Is any of your child (06 to 59 months) received any treatment who was/were suffering from SAM/MAM?	1 = Yes 2 = No<br>77= don't know		
412	CM_412	If answer "Yes"<br>Ask from where you received the treatment?	1=SAM corner<br>2=MBBS doctor<br>3=CHCP/community clinic<br>99=Others (specify bellow)…….		
413	CM_413	Sickness outcome?	1= Cured<br>2= Non-recovered<br>3= The treatment was not completed<br>4= Others (specify bellow)………..		

Section 5: Child Anthropometry

No.	Variable	Question	Response(s)	Code	Instructions

No.	Variable	Question	Response(s)	Code	Instructions
501	CA_501	Start Time Anthropometry	|____|____|:|____|____| Hour : Minute		Use 24 Hr Format
502	CA_502	Weight Scale ID	|____|____|		
503	CA_5403	Height/length Scale ID	|____|____|		
504	CA_504	Clothing type during weight measurement	1 = Light clothing<br>2 = Slightly heavy clothing<br>3 = Heavy clothing		
505	CA_505	Name of measurer			
506	CA_506	Code of Measurer	|____|____|		

No	Variable	Measurement	Measurement 1	Measurement 2	Measurement 3
507	CA_507 A/B/C	Weight(Kg)	A.|____|____|.|____|	B. |____|____|.|____|	C. |____|____|.|____| If A and B differs more than 0.05 Kg, take 3rdmeasurement
508	CA_508 A/B/C	Left hand circumference(MUAC) (Cm)	A.|____|____|.|____|	B.|____|____|.|____|	C.|____|____|.|____| If A and B differs more than 0.5 cm, take 3rd measurement
509	CA_509 A/B/C	Length(Cm)	A.|____|____|.|____|	B.|____|____|.|____|	C.|____|____|.|____| If A and B differs more than 0.5 cm, take 3rd measurement
510	CA_510	Length/height measured in system	1 = Standing position<br>2 = Recumbent position		
511	CA_511	Edema	1 = Yes 2 = No 77 = Don't know		
512	CA_512	Is the child co-operative	1 = Cooperative<br>2 = Non-cooperative<br>88= Not applicable		
513	CA_513	Interviewers comments about measurement			
514	CA_514	End Time of measurement	|____|____|:|____|____| Hr : Min		Use 24 hr format

Section 6: Referral

No.	Variable	Question	Responses and code categories	Code	Instructions
601	CA_601	Was the child referred?	1 = Yes 2 = No		If 'No', Skip to next section
602	CA_602 A/B/C /D/E/F	Reason of referral	A.|____|____| B.|____|____| C.|____|____|D.|____|____| E.|____|____| F.|____|____|		Multiple reasons possible
**Cause of referral code :**1= Weight for height Z score <- 2; 2= Weight for height Z score <- 3; 3= MUAC 12.4-11.5 cm; 4= MUAC <11.5 cm; 99 = Others (Specify) _________					
603	CA_603	Where referred?	1= Upazilla Health Complex<br>2= District Hospital<br>3= Medical College Hospital<br>4= Doctor's private chamber<br>99 = Others (Specify) _________		

Module 4: Adolescent Boys Module

Section 1: General Information

No	Variable	Question	Response Options	Code	Instructions/ Skip
101	AB_101	What's your name?(with nick name)	___________________________		
102	AB_102	What's your date of birth	|____|____|/|____|____|/20|____|____| DD / MM / YYYY		
103	AB_103	Age of the respondent (Write complete year)	|____|____| year		
104	AB_104	What is your marital status?	1 = Never married<br>2 = Currently married<br>3 = Separated<br>4 = Divorced<br>5 = Widowed<br>88 = Refused		
105	AB_105	Completed years of education of respondent **[Instruction:** Write down in total, the years the respondent spent in school for formal education (Is completed years) [Excluding pre-school and informal education). Write down "00" if no formal education.]	|____|____| Years<br>• Primary/Ebtedayee=5<br>• Secondary/Dakhil=10<br>• Higher secondary/ Diploma/Alim=12<br>• Graduate/Fazil=16<br>• Post graduate/kamil/dawra=18		
106	AB_106	Main Occupation of the respondent(adolescent name) over the last 12 months<br>**Instruction:**<br>If the respondent involved more than one profession since last 12 months, then consider the main occupation which she spent most of the time and write down the answer code.	1 = Agricultural work(paddy)<br>2 = Agricultural work(without paddy)<br>3 = Agricultural day labor<br>4 = Un skild day labor<br>5 = Skiled day labor<br>6= Driver of Rickshaw/ van/Wheel barrow/Baby taxi/ Boatman<br>7=Fisherman<br>8=Employer<br>9=Profesionals<br>10=Businessman<br>11= Petty businessman<br>12= House maid<br>13=Zoom fermar<br>14= Do not earn<br>15=Poultry/dairy fermar<br>16=Handicrafts<br>17 =Spinach vegitable<br>18=Fishery<br>19 = Homemaker<br>20= Student<br>77 = Don,t know<br>66 =Age below 06 years		
107	AB_107	Completed years of education of the respondent mother **Instruction:**<br>If the respondent involved more than one profession since last 12 months, then consider the main	|__|__| Years<br>• Primary/Ebtedayee=5<br>• Secondary/Dakhil=10<br>• Higher Secondary/Diploma/Alim=12<br>• Graduate/Fazil=16<br>• Post graduate/kamil/dawra=18		

No	Variable	Question	Response Options	Code	Instructions/ Skip
		occupation which she spent most of the time and write down the answer code			
108	AB_108	Respondent(adolescent name)<br>Mother main occupation over the last 12 months?<br>Instruction :If the participant has been employed more than one occupation in the last 12 months, consider the profession that he has spent most of his time as the main occupation and write it down	1 = Agricultural work(paddy)<br>2 = Agricultural work(without paddy)<br>3 = Agricultural day labor<br>4 = Un skild day labor<br>5 = Skiled day labor<br>6= Driver of Rickshaw/ van/Wheel barrow/Baby taxi/ Boatman<br>7=Fisherman<br>8=Employer<br>9=Profesionals<br>10=Businessman<br>11= Petty businessman<br>12= House maid<br>13=Zoom fermar<br>14= Do not earn<br>15=Poultry/dairy fermar<br>16=Handicrafts<br>17 =Spinach vegitable<br>18=Fishery<br>19 = Homemaker<br>20= Student<br>77 = Don,t know<br>66 =Age below 06 years		
109	AB_109	Completed years of education of the father of the respondent<br>**[Instruction:** Write down in total, the years the respondent spent in school for formal education (Is completed years) [Excluding pre-school and informal education). Write down "00" if no formal education.]	|__|__| Years<br>• Primary/Ebtedayee=5<br>• Secondary/Dakhil=10<br>• Higher/ Secondary/ Diploma/Alim=12<br>• Graduate/Fazil=16<br>• Post graduate/kamil/dawra=18		
110	AB_110	Father's occupation of the respondent over the past 12 months<br>**Instruction :**If the participant has been employed more than one occupation in the last 12 months, consider the profession that he has spent most of his time as the main occupation and write it down.	1 = Agricultural work(paddy)<br>2 = Agricultural work(without paddy)<br>3 = Agricultural day labor<br>4 = Un skild day labor<br>5 = Skiled day labor<br>6= Driver of Rickshaw/ van/Wheel barrow/Baby taxi/ Boatman<br>7=Fisherman<br>8=Employer<br>9=Profesionals<br>10=Businessman<br>11= Petty businessman<br>12= House maid<br>13=Zoom fermar<br>14= Do not earn<br>15=Poultry/dairy fermar<br>16=Handicrafts		

No	Variable	Question	Response Options	Code	Instructions/ Skip
			17 =Spinach vegitable<br>18=Fishery<br>19 = Homemaker<br>20= Student<br>77 = Don,t know<br>66 =Age below 06 years		

Section 2: Dietary diversity of adolescent boys

**No**	**Variable**		**Question**	**Yesterday during the day or night, did your child consume the following food items? (A)**	**During the last 07 days how many days did your child consume the following food items (B)**	**During the last 07 dayshow many times did your child consume the following food items (C)**
Now I would like to requst describing the food items that you ate at home and outside of home during the last day or night(from yesterday morning 6.00 am to today morning 6.00 am) .Please tell me,all types of food, drinks that ate you at morning, noon and dinner, or breakfast / light breakfast. Remembering If you ate any of food during the cooking, please tell us including all.<br><br>Start the interview like, which food give your child at the morning time .<br><br>-What did you eat in the morning wake up ? Did you eat anything more?<br>-What did you eat more in the morning? Did you eat anything more?<br>-What did you eat at noon? Did you eat anything more?<br>-What did you eat at afternoon? Did you eat anything more?<br>-What did you eat at evening? Did you eat anything more?<br>-What did you eat at dinner? Did you eat anything more?<br>**Record first the answer of the questions of column A. collect the answer for column B/C after recording the answer of the questions of column A.**						
		Lvevi aiY	Lvevi bgybv			
201	ABD_201 A/B/C	Starchy Staples/ Food made from grains	Rice,flour bread, wheat, muri, maize,kichuri,barli,oot,kinoya,noodles,pasta	1 = Yes<br>2 = No	|____|____| days	|____|____| times
202	ABD_202 A/B/C	Starchy Staples/ (root,tubers, tissue)	potatoes, sweet potatoes,sagu, erarut,cave,shalgam,kuchu,wiggle, ripe banana shaloo	1 = Yes<br>2 = No	|____|____| days	|____|____| times
203	ABD_203 A/B/C	Lentils/Pulses	Dal/khicuri,peas,motor,soybean,tofu peanuts, humas	1 = Yes<br>2 = No	|____|____| days	|____|____| times
204	ABD_204 A/B/C	Legumes and Nuts	Peanut, Pistachios, kazu or any kond of peanut, ciya seeds, til, tisi,sunflower seeds,sweet pumpkin seeds	1 = Yes<br>2 = No	|____|____| days	|____|____| times
205	ABD_205 A/B/C	Dark green leafy vegetables	All kinds of leafy vegetables (poo,kuchu, kalmi) Brooklyn	1 = Yes<br>2 = No	|____|____| days	|____|____| times
206	ABD_206 A/B/C	Red/orange/ yellow vegetables	Orange sweet potato,pumpkin, carrot or other yellow or orange vegetable	1 = Yes<br>2 = No	|____|____| days	|____|____| times
207	ABD_207 A/B/C	Red/orange/ yellow fruits	Ripe mangoes, papaya, jackfruits other red/yellow or orange fruit	1 = Yes<br>2 = No	|____|____| days	|____|____| times

No	Variable	Question		Yesterday during the day or night, did your child consume the following food items? (A)	During the last 07 days how many days did your child consume the following food items (B)	During the last 07 dayshow many times did your child consume the following food items (C)
208	ABD_208 A/B/C	Vitamin C rich fruits	Guava, Strawberry, Lemon, Orange,grapes, Pineapple,raw mango,amalaki,kueue,tomatos	1 = Yes<br>2 = No	|____|____| days	|____|____| times
209	ABD_209 A/B/C	Vitamin C rich vegetables	Raw tomato,chilli pepper, brussels sprouts,cauliflower,cabbage	1 = Yes<br>2 = No	|____|____| days	|____|____| times
210	ABD_210 A/B/C	Other vegetables or fruits	peas, asparagas, bit,koci bash, cauliflower,selari,shawsha,brinjal,letus,masrum,mula,jukini	1 = Yes<br>2 = No	|____|____| days	|____|____| times
211	ABD_211 A/B/C	Other fruits	Apple, avakado,bam, cherry,ripe jackfruit	1 = Yes<br>2 = No	|____|____| days	|____|____| times
212	ABD_212 A/B/C	Eggs	Hen/duck, other birds,	1 = Yes<br>2 = No	|____|____| days	|____|____| times
213	ABD_213 A/B/C	Organ meat	Cheeks, liver, Stomach, Ridpind, Kidney	1 = Yes<br>2 = No	|____|____| days	|____|____| times
214	ABD_214 A/B/C	Flesh	Beef, Pork, Veal, Lamb, Goat, Chicken, Duck or flesh of any other animal	1 = Yes<br>2 = No	|____|____| days	|____|____| times
215	ABD_215 A/B/C	Small Fish	Small Fish Eaten Whole with Bones (i.e.Kachki, mola, dhela, chapila, batashi, small prawn, dried fish)	1 = Yes<br>2 = No	|____|____| days	|____|____| times
216	ABD_216 A/B/C	Large fish/sea food	Big whole Fish and Shell Fish and dried fish	1 = Yes<br>2 = No	|____|____| days	|____|____| times
217	ABD_217 A/B/C	Dairy	Milk, cheese, yogurt or other milk products	1 = Yes<br>2 = No	|____|____| days	|____|____| times
218	ABD_218 A/B/C	Insects and other protein foods	Fish egg, insect, snail	1 = Yes<br>2 = No	|____|____| days	|____|____| times
219	ABD_219 A/B/C	Edible Oil	Ghee, butter,cream, sour, fat, margarine, mayonnaise, palm oil, vegetable oil,	1 = Yes<br>2 = No	|____|____| days	|____|____| times
220	ABD_220 A/B/C	Savory and fried snacks	Crisps and chips, fried dough or other fried snacks singaru, samarcha	1 = Yes<br>2 = No	|____|____| days	|____|____| times
221	ABD_221 A/B/C	Sweets	Sugary foods, such as chocolates, candies, cookies/sweet biscuits and cakes, sweet pastries or ice cream, any kind of sweets ,honey ,halua ,	1 = Yes<br>2 = No	|____|____| days	|____|____| times

No	Variable	Question		Yesterday during the day or night, did your child consume the following food items? (A)	During the last 07 days how many days did your child consume the following food items (B)	During the last 07 dayshow many times did your child consume the following food items (C)
			condensed milk,tiler khaza			
222	ABD_222 A/B/C	Miscellaneous (Sweet drinks)	Tea with suger, soft drinks, juice, energy drinks,yogurt drinks,chocolate drinks, horlicks,moltova	1 = Yes<br>2 = No	|____|____| days	|____|____| times
223	ABD_223 A/B/C	Other beverages and foods	Tea or coffee if not sweetened, clear broth, alcohol, Pickles, olives and similar	1 = Yes<br>2 = No	|____|____| days	|____|____| times
224	ABD_224 A/B/C	Condiments /spices	Spices, coriander leaves, sausage, garlic, ketchup, lemon juice, mint leaves, drinks, betel leaves, tobacco leaves, jars	1 = Yes<br>2 = No	|____|____| days	|____|____| times

No	Variable	Question	Response Options	Code	Instructions/ Skip
The next ,I will ask about the **fruits** and **vegetables** that you usually eat; I have a nutrition card/picture here that shows you some examples of local fruits and vegetables; Each picture represents the size of a serving; To answer these questions, please think of a typical week.(If 1-2 times/month then record it as "00.")					
225	ABD_225	In a typical week, on how many days do you **eat fruit**?<br><br>***Instruction:*** *Demonstrate fruit's show card and ask the participant to think about it. Do not consider packaged fruit juice but consider fruit juice prepared at home using fresh fruits. A typical week refers to "A usual week except religious event or any other festival. If 1-2 times/month then record it as "00."* [USE SHOWCARD]	Number of days |____|____| 77 = Don't Know<br>[If '00' days, go to ABD_227]		
226	ABD_226	How many **servings** of fruit do you eat on **one** of those days? (USE SHOWCARD)<br><br>***Instruction:*** *Ask the participant to think about any one of those days of a typical week. Use supplied bowl and show card to measure servings.*	Number of servings<br>|___|___|.|___| 77.7 = Don't Know		
227	ABD_227	In a typical week, on how many days do you **eat vegetables**?<br><br>***Instruction:*** *Demonstrate vegetable's show card and ask the participant to think about it. Do not consider potato as vegetables. Demonstrate serving with the help of supplied cup and show card as cooked and uncooked vegetables. Consider only vegetables serving in case of cooked vegetables with fish. A typical week refers to "A usual week except religious event or any other festival. If 1-2 times/month then record it as "00."* [USE SHOWCARD]	Number of days<br>|____|____| 77 = Don't Know<br>[If '0' days, go to ABD_229]		
228	ABD_228	How many **servings** of vegetables do you eat on one of those days?<br><br>***Instruction:*** *Ask the participant to think about any one of those days of a typical week. Use supplied cup and show card to measure serving.*[USE SHOWCARD]	Number of servings<br>|___|___|.|___| 77.7 = Don't Know		
229	ABD_229	Are you eating any tablet, capsul,syrup which contaned vitamin/minarels currently.	1=Yes 2=No		If Answer No ,go to next section
230	ABD_230	Write the name of medicine by observed the prescription and medication	A.____________<br>B.____________<br>C.____________		

Section 3: Behavioral risk factor (Smoking/smokeless tobacco physical activity)

b¤^i	m~PK	cÖkœ	DËi	‡KvW	wb‡`©kbv
**g~jt Zvgv‡Ki e¨envi**					
GLb Avwg Avcbv‡K ZvgvK I ZvgvKRvZ `ª‡e¨i (†hgbt a~gcvb, †avuqvwenxb Zvgv‡Ki) e¨envi m¤ú‡K© wKQz cÖkœ wRÄvmv Kie|					
301	ABR_301	Do you <u>currently</u> smoke any tobacco products, such as cigarettes, *bidis, hookah, cigars* or pipes?<br><br>*Instruction: Demonstrate show card to the participant and ask which smoke tobacco products/he use.* [USE SHOWCARD]	1 = Yes; 2= No<br>If No, go to ABR_304		
302	ABR_302	Do you <u>currently</u> smoke tobacco products <u>daily</u>?<br><br>*Instruction: This question is applicable for those respondents who smoke tobacco product daily. Daily meanus" tobacco smoking is at least one time/day for almost one month or more than that If that is the case respondant started somoking from 25 days and still running, that is regarded as everyday.*	1 = Yes; 2= No		
303	ABR_303	How old were you when you **first started** smoking?	Age └─┴─┘ years<br>77 = Don't know		
304	ABR_304	Do you currently use anysmokeless tobacco products such as Betel quid with zarda, zarda only or zarda with supari, Betel quid with sadapata, pan masala withtobacco, sadapata chewing, gul, Khoinee, Nossi, gutka?<br>***Instruction:*** *Ask respondent to think and answer whether h/she use any smokeless tobacco products such as Betel quid with zarda, zarda only or zarda with supari, Betel quid with sadapata, pan masala with tobacco, sadapata chewing, gul, Khoinee, Nossi, gutka. Here, only betel quid, supari and lime will not be considered. It will be considered as smokeless tobacco if respondent use Betel quid with zarda, zarda only or zarda with supari, Betel quid with sadapata, pan masala with tobacco, sadapata chewing, gul, Khoinee, Nossi, gutka.* [USE SHOWCARD]	1 = Yes; 2= No<br><br>If No, go to ABR_307		
305	ABR_305	Do you <u>currently</u> use <u>smokeless tobacco</u> <u>daily</u>?<br>***Instruction:*** *Daily means "using smokeless tobacco is at least one time/day for almost one month or more than that.*<br>*If that is the case respondant started somoking from 25 days and still running, that is regarded as everyday.*	1 = Yes; 2= No		
306	ABR_306	How old were you when you started smokeless tobacco?	Age └─┴─┘ years<br>77 = Don't know		
307	ABR_307	Which of your parents or guardians use any form of tobacco?	1= Neither<br>2= My father or male guardian<br>3= My mother or female guardian<br>4= Both<br>5= I do not know<br>99= Others (Specify)_______		
**Physical activity**					

<table>
<tr><td colspan="6">Next I am going to ask you about the time you spend doing different types of physical activity in a typical week. Please answer these questions even if you do not consider yourself to be a physically active person.
Think first about the time you spend doing work. Think of work as the things that you have to do such as paid or unpaid work, study/training, household chores, harvesting food/crops, fishing or hunting for food, seeking employment. [Insert other examples if needed]. In answering the following questions 'vigorous-intensity activities' are activities that require hard physical effort and cause large increases in breathing or heart rate, 'moderate-intensity activities' are activities that require moderate physical effort and cause small increases in breathing or heart rate.
Instruction: Please read out the above statement for respondent. Do not drop this part. At first respondent will must think about his/her daily activities (Paid or unpaid work, study/training, household chores, harvesting food/crops, fishing or hunting for food, seeking employment). Then will think time for travelling from one place to another and at last time spent during leisure period.
Do not forget to demonstrate show card to the respondent which will help them to respond.
It is needed to let participant remember when answer the following things
Vigorous intensity activity: Such kind of physical activity that causes large increases in breathing or heart rate.
Moderate-intensity activity: Such kind of physical activity that causes small increases in breathing or heart rate.</td></tr>
<tr><td colspan="6">Now I would like to know about the 'Vigorous-intensity activities' you perform</td></tr>
<tr><td colspan="6">'Vigorous-intensity activities' performed as a part of daily work / professional work/ outside daily work / professional work.</td></tr>
<tr><td>308</td><td>ABR_308</td><td>Does your daily work / professional work, sports, fitness or recreational(leisure) activities involve vigorous-intensity activity that causes large increases in breathing or heart rate like [carrying or lifting heavy loads, digging or construction work, harvesting paddy, fishing using net, running, football, Kabaddi, Dariabandha, Gollachut etc.] for at least 10 minutes continuously?
Instruction: Ask participant to think about those vigorous-intensity activities performed as a part of professional work/ recreational activities. Those activities will be considered as vigorous-intensity activities that causes large increase in breathing or heart rate. [USE SHOWCARD]</td><td>1 = Yes 2 = No
[If 'No', please go to 311]</td><td></td><td></td></tr>
<tr><td>309</td><td>ABR_309</td><td>In a typical week, on how many days do you do vigorous- intensity activities/sports/fitness as part of daily work / professional work/ recreational activities?
Instruction: In a typical week refers to "activities performed by respondent in a typical week". Valid response range is 1-7 days.</td><td>|__|__| Day
77 = Don't know
[If 'Don't know', please go to ABR_311]</td><td></td><td></td></tr>
<tr><td>310</td><td>ABR_310</td><td>How much time do you spend doing vigorous-intensity activities/sports/fitness at daily work / professional work/recreational activities on a typical day?
Instruction: Ask respondent to think about a single day (which can be remembered easily) s/he performed vigorous-intensity activities as part of professional work/ recreational activities. Respondent will consider only those activities that are performed for at least 10 minutes or more continuously. Check unusual (more than 04 hours) values.</td><td>__________
Minutes</td><td></td><td></td></tr>
<tr><td colspan="6">Now I would like to know about the 'Moderate-intensity activity' you perform</td></tr>
<tr><td colspan="6">'Moderate-intensity activity' performed as a part of daily work / professional work</td></tr>
</table>

311	ABR_311	Does your daily work / professional work/ spots/fitness/ recreational (leisure) work involve moderate-intensity activity that causes small increases in breathing or heart rate such as brisk walking [or carrying light loads, cycling, swimming, volleyball] for at least 10 minutes continuously? ***Instruction:*** *Ask participant to think about those moderate-intensity activities performed as a part of professional work/ recreational activities. Those activities will be considered as moderate-intensity activities that causes small increase in breathing or heart rate.* [USESHOWCARD]	1 = Yes 2 = No [If 'No', please go to ABR_314]		
312	ABR_312	In a typical week, on how many days do you do moderate- intensity activities as part of your daily work / professional work/sports/fitness or recreational work? ***Instruction:*** *In a typical week refers to "activities done by respondent in a typical week". Valid response range is 1-7 days.*	└─┴─┘ Day 77 = Don't know [If 'Don't know', please go to ABR_314]		
313	ABR_313	How much time do you spend doing moderate-intensity activities at daily work / professional work/ sports/fitness or recreational work on a typical day? ***Instruction:*** *Ask respondent to think about a single day (which can be remembered easily) s/he performed moderate-intensity activities as part of professional work/recreational activities. Respondent will consider only those activities that are performed for at least 10 minutes or more continuously. Check unusual (more than 04 hours) values.*	________ Minutes		
**Sedentary behavior** The following question is about sitting or reclining at work, at home, getting to and from places, or with friends including time spent sitting at a desk, sitting with friends, traveling in car, bus, train, reading, playing cards or watching television, but do not include time spent sleeping.					
314	ABR_314	How much time do you usually spend sitting or reclining on a typical day? ***Instruction:*** *Ask respondent to think about time spent on sitting or reclining at work, office, study, watching television, computer use, at kitchen, during rest.Do not include time spent during sleeping.*	________ Minutes		
315	ABR_315	How much time do you watch television daily?	________ Minutes		

Section 4: Mental Health

No	Variable	Question	Response Options	Code	Instructions
Over the last 2 weeks how often you have faced the following problems?					
401	ABM_401	Less interest or less joy to do something	0=Not at all<br>1=Some time<br>2=Most of the time<br>3= All time		
402	ABM_402	Feeling Upset, depressed,irritable,hopeless	0=Not at all<br>1=Some time<br>2=Most of the time<br>3= All time		
403	ABM_403	Difficulty to sleeping or sleeping too much	0=Not at all<br>1=Some time		

No	Variable	Question	Response Options	Code	Instructions
Over the last 2 weeks how often you have faced the following problems?					
			2=Most of the time<br>3= All time		
404	ABM_404	Tired or low strength	0=Not at all<br>1=Some time<br>2=Most of the time<br>3= All time		
405	ABM_405	Loss of appetite,weight loss or eat more	0=Not at all<br>1=Some time<br>2=Most of the time<br>3= All time		
406	ABM_406	Have a bad feeling about self, feel self as a failure, Making myself or my family smaller feel like this	0=Not at all<br>1=Some time<br>2=Most of the time<br>3= All time		
407	ABM_407	Difficulty to concentrate at the time of work at school,read something or watch television	0=Not at all<br>1=Some time<br>2=Most of the time<br>3= All time		
408	ABM_408	Talk so lightly or move around that others might notice or the opposite of this that means more severity or more movement than usual.	0=Not at all<br>1=Some time<br>2=Most of the time<br>3= All time		
409	ABM_409	It is better to die or hit yourself in any way feel like this	0=Not at all<br>1=Some time<br>2=Most of the time<br>3= All time		If "0" go to section 5

Referral

No	Variable	Question	Response Options	Code	Instructions
410	ABM_410	Was the adolescent referred?	1 = Yes 2 = No		If 'No', Skip to next section
411	ABM_411	Where referred?	1= Upazilla Health Complex<br>2= District Hospital<br>3= Medical College Hospital<br>4= Doctor's private chamber<br>99 = Others (Specify) __________		

Section 5: Anthropometry

No	Variable	Question	Responses and code categories	Code	Instructions
501	ABA_501	Start Time of anthropometry	|___|___| : |___|___| Hr : Min		Use 24 hr format
502	ABA_502	Weight Scale ID	|___|___|		
503	ABA_503	Height Scale ID	|___|___|		
504	ABA_504	Clothing type during weight measurement	1 = Light clothing<br>2 = Slightly heavy clothing<br>3 = Heavy clothing		
505	ABA_505	Name of measurer			
506	ABA_506	Code of measurer	|___|___|		

No	Measurement	Measurement 1	Measurement 2	Measurement 3

No	Variable	Question	Responses and code categories		Code / Instructions
507	ABA_507 A/B/C	Height (Cm)	A.|____|____|____|.|____|	B.|____|____|____|.|____|	C.|____|____|____|.|____| If A and B differs more than 0.5cm, take 3rd measurement
508	ABA_508 A/B/C	Weight (Kg)	A.|___|____|____|.|____|	B.|____|____|____|.|____|	C.|____|____|____|.|____| If A and B differs more than 0.1kg, take 3rd measurement
509	ABA_509A/B/C	Fat%	A.|____|____|____|.|____|	B.|____|____|____|.|____|	
510	ABA_510A/B/C	Water%	A.|____|____|____|.|____|	B.|____|____|____|.|____|	
511	ABA_511 A/B/C	Waist circumference (Cm)	A.|____|____|.|____|	B.|____|____|.|____|	C.|____|____|.|____| If A and B differs more than 0.5 cm, take 3rd measurement
512	ABA_512	Comment			
513	ABA_513	End Time of measurement	|____|____|:|____|____| Hr : Min		Use 24 hr format

Module 5: Adolescent Girls Module

Section 1: General Information

No	Variable	Question	Response Options	Code	Instructions/ Skip
101	AG_101	What's your name?(with nick name)	________________________		
102	AG_102	What's your date of birth	|____|____|/|____|____|/20|____|____| DD / MM / YYYY		
103	AG_103	Age of the respondent (Write complete year)	|____|____| year		
104	AG_104	What is your marital status?	1 = Never married<br>2 = Currently married<br>3 = Separated<br>4 = Divorced<br>5 = Widowed<br>88 = Refused		
105	AG_105	Completed years of education the respondent completed<br><br>***[Instruction:** Write down in total, the years the respondent spent in school for formal education (Is completed years) [Excluding pre-school and informal education). Write down "00" if no formal education.]*	|____|____| Years<br><br>• Primary/Ebtedayee=5<br>• Secondary/Dakhil=10<br>• Higher secondary/ Diploma/Alim=12<br>• Graduate/Fazil=16<br>• Post graduate/kamil/dawra=18		
106	AG_106	Occupation of the respondent(adolescent name) over the past 12 months<br>If the participant has been	1 = Agricultural work(paddy)<br>2 = Agricultural work(without paddy)<br>3 = Agricultural day labor<br>4 = Un skild day labor<br>5 = Skiled day labor		

No	Variable	Question	Response Options	Code	Instructions/ Skip
		employed more than one occupation in the last 12 months, consider the profession that he has spent most of his time as the main occupation and write it down	6= Driver of Rickshaw/ van/Wheel barrow/Baby taxi/ Boatman<br>7=Fisherman<br>8=Employer<br>9=Profesionals<br>10=Businessman<br>11= Petty businessman<br>12= House maid<br>13=Zoom fermar<br>14= Do not earn<br>15=Poultry/dairy fermar<br>16=Handicrafts<br>17 =Spinach vegitable<br>18=Fishery<br>19 = Homemaker<br>20= Student<br>77 = Don,t know<br>66 =Age below 06 years		
107	AG_107	Completed years of education of the mother of the respondent (adolescent girls name)<br><br>***[Instruction:** Write down in total, the years the respondent spent in school for formal education (Is completed years) [Excluding pre-school and informal education). Write down "00" if no formal education.]*	|__|__| Years<br>• Primary/Ebtedayee=5<br>• Secondary/Dakhil=10<br>• Higher Secondary/Diploma/Alim=12<br>• Graduate/Fazil=16<br>• Post graduate/kamil/dawra=18		
108	AG_108	Mother's occupation of the respondent(adolescent name) over the past 12 months<br>If the participant has been employed more than one occupation in the last 12 months, consider the profession that he has spent most of his time as the main occupation and write it down	1 = Agricultural work(paddy)<br>2 = Agricultural work(without paddy)<br>3 = Agricultural day labor<br>4 = Un skild day labor<br>5 = Skiled day labor<br>6= Driver of Rickshaw/ van/Wheel barrow/Baby taxi/ Boatman<br>7=Fisherman<br>8=Employer<br>9=Profesionals<br>10=Businessman<br>11= Petty businessman<br>12= House maid<br>13=Zoom fermar<br>14= Do not earn<br>15=Poultry/dairy fermar<br>16=Handicrafts<br>17 =Spinach vegitable<br>18=Fishery<br>19 = Homemaker<br>20= Student<br>77 = Don,t know<br>66 =Age below 06 years		

No	Variable	Question	Response Options	Code	Instructions/ Skip
109	AG_109	Completed years of education of the father of the respondent ***[Instruction:** Write down in total, the years the respondent spent in school for formal education (Is completed years) [Excluding pre-school and informal education). Write down "00" if no formal education.]*	└┴┘ Years<br>• Primary/Ebtedayee=5<br>• Secondary/Dakhil=10<br>• Higher/ Secondary/ Diploma/Alim=12<br>• Graduate/Fazil=16<br>• Post graduate/kamil/dawra=18		
110	AG_110	Father's occupation of the respondent over the past 12 months<br>If the participant has been employed more than one occupation in the last 12 months, consider the profession that he has spent most of his time as the main occupation and write it down	1 = Agricultural work(paddy)<br>2 = Agricultural work(without paddy)<br>3 = Agricultural day labor<br>4 = Un skild day labor<br>5 = Skiled day labor<br>6= Driver of Rickshaw/ van/Wheel barrow/Baby taxi/ Boatman<br>7=Fisherman<br>8=Employer<br>9=Profesionals<br>10=Businessman<br>11= Petty businessman<br>12= House maid<br>13=Zoom fermar<br>14= Do not earn<br>15=Poultry/dairy fermar<br>16=Handicrafts<br>17 =Spinach vegitable<br>18=Fishery<br>19 = Homemaker<br>20= Student<br>77 = Don,t know<br>66 =Age below 06 years		

Section 2: Dietary diversity

	Variable	Question	Yesterday during the day or night, did your child consume the following food items? (A)	During the last 07 days how many days did your child consume the following food items (B)	During the last 07 dayshow many times did your child consume the following food items (C)
Now I requst you to describe the food that you have eaten at home and out of home during the day or night(yesterday morning 6.00 am to today morning 6.00 am) yesterday. Please tell all types of food, drinks that you have eaten with morning, noon and dinner, or have breakfast / light breakfast. Tell us by think if you ate any food while preparing food.<br><br>Get started with what you ate yesterday morning.<br>-What did you eat in the morning wake up? Did you eat anything more?<br>-What did you eat more in the morning? Did you eat anything more?<br>-What did you eat at noon? Did you eat anything more?<br>-What did you eat at afternoon? Did you eat anything more?<br>-What did you eat at evening? Did you eat anything more?					

	Variable	Question		Yesterday during the day or night, did your child consume the following food items? (A)	During the last 07 days how many days did your child consume the following food items (B)	During the last 07 dayshow many times did your child consume the following food items (C)
-What did you eat at dinner? Did you eat anything more? **Record first the answer of the questions of column A. collect the answer for column B/C after recording the answer of the questions of column A.**						
		Food Category	Examples of foods	A	B	C
201	AGD_201 A/B/C	Starchy Staples/ Food made from grains	Rice,flour bread, wheat, muri, maize,kichuri,barli,oot,kinoya,noodles,pasta	1 = Yes 2 = No	|____|____| days	|____|____| times
202	AGD_202 A/B/C	White roots and tubers and plantains	potatoes, sweet potatoes,sagu, erarut,cave,shalgam,kuchu,wiggle,ripe banana shaloo	1 = Yes 2 = No	|____|____| days	|____|____| times
203	AGD_203 A/B/C	Pulses	Dal/khicuri,peas,motor,soybean,tofu peanuts, humas	1 = Yes 2 = No	|____|____| days	|____|____| times
204	AGD_204 A/B/C	Legumes and Nuts	Peanut, Pistachios, kazu or any kond of peanut, ciya seeds, til, tisi,sunflower seeds,sweet pumpkin seeds	1 = Yes 2 = No	|____|____| days	|____|____| times
205	AGD_205 A/B/C	Dark green leafy vegetables	All kinds of leafy vegetables (poo,kuchu, kalmi) Brooklyn	1 = Yes 2 = No	|____|____| days	|____|____| times
206	AGD_206 A/B/C	Red/orange/yellow vegetables	Orange sweet potato,pumpkin, carrot or other yellow or orange vegetable	1 = Yes 2 = No	|____|____| days	|____|____| times
207	AGD_207 A/B/C	Red/orange/yellow fruits	Ripe mangoes, papaya, jackfruits other red/yellow or orange fruit	1 = Yes 2 = No	|____|____| days	|____|____| times
208	AGD_208 A/B/C	Vitamin C rich fruits	Guava, Strawberry, Lemon, Orange,grapes, Pineapple,raw mango,amalaki,kueue,tomatos	1 = Yes 2 = No	|____|____| days	|____|____| times
209	AGD_209 A/B/C	Vitamin C rich vegetables	Raw tomato,chilli pepper, brussels sprouts,cauliflower,cabbage	1 = Yes 2 = No	|____|____| days	|____|____| times
210	AGD_210 A/B/C	Other vegetables or fruits	peas, asparagas, bit,koci bash, cauliflower,selari,shawsha,brinjal,letus, masrum,mula,jukini	1 = Yes 2 = No	|____|____| days	|____|____| times
211	AGD_211 A/B/C	Other fruits	Apple, avakado,bam, cherry,ripe jackfruit	1 = Yes 2 = No	|____|____| days	|____|____| times

	Variable	Question		Yesterday during the day or night, did your child consume the following food items? (A)	During the last 07 days how many days did your child consume the following food items (B)	During the last 07 dayshow many times did your child consume the following food items (C)
212	AGD_212 A/B/C	Eggs	Hen/duck, other birds,	1 = Yes<br>2 = No	|____|____| days	|____|____| times
213	AGD_213 A/B/C	Organ meat	Cheeks, liver, Stomach, Ridpind, Kidney	1 = Yes<br>2 = No	|____|____| days	|____|____| times
214	AGD_214 A/B/C	Flesh	Beef, Pork, Veal, Lamb, Goat, Chicken, Duck or flesh of any other animal	1 = Yes<br>2 = No	|____|____| days	|____|____| times
215	AGD_215 A/B/C	Small Fish	Small Fish Eaten Whole with Bones (i.e.Kachki, mola, dhela, chapila, batashi, small prawn, dried fish)	1 = Yes<br>2 = No	|____|____| days	|____|____| times
216	AGD_216 A/B/C	Large fish/sea food	Large Whole Fish and Shell Fish and dried fish	1 = Yes<br>2 = No	|____|____| days	|____|____| times
217	AGD_217 A/B/C	Dairy	Milk, cheese, yogurt or other milk products	1 = Yes<br>2 = No	|____|____| days	|____|____| times
218	AGD_218	Insects and other protein foods	Fish egg, insect, snail	1 = Yes<br>2 = No	|____|____| days	|____|____| times
219	AGD_219 A/B/C	Edible Oil	Ghee, butter,cream, sour, fat, margarine, mayonnaise, palm oil, vegetable oil,	1 = Yes<br>2 = No	|____|____| days	|____|____| times
220	AGD_220 A/B/C	Savory and fried snacks	Crisps and chips, fried dough or other fried snacks singaru, samarcha	1 = Yes<br>2 = No	|____|____| days	|____|____| times
221	AGD_221 A/B/C	Sweets	Sugary foods, such as chocolates, candies, cookies/sweet biscuits and cakes, sweet pastries or ice cream, any kind of sweets ,honey ,halua , condensed milk,tiler khaza	1 = Yes<br>2 = No	|____|____| days	|____|____| times
222	AGD_222 A/B/C	Miscellaneous (Sweet drinks)	Tea with suger, soft drinks, juice, energy drinks,yogurt drinks,chocolate drinks, horlicks,moltova	1 = Yes<br>2 = No	|____|____| days	|____|____| times
223	AGD_223	Other beverages and foods	Tea or coffee if not sweetened, clear broth, alcohol, Pickles, olives and	1 = Yes<br>2 = No	|____|____| days	|____|____| times

	Variable	Question		Yesterday during the day or night, did your child consume the following food items? (A)	During the last 07 days how many days did your child consume the following food items (B)	During the last 07 dayshow many times did your child consume the following food items (C)
	A/B/C		similar			
224	AGD_224 A/B/C	Condiments/spices	Spices, coriander leaves, sausage, garlic, ketchup, lemon juice, mint leaves, drinks, betel leaves, tobacco leaves, jars	1 = Yes<br>2 = No	|____|____ | days	|____|____ | times

No	Variable	Question	Response Options	Code	Instructions/ Skip
The next questions I will ask about the **<u>fruits</u>** and **<u>vegetables</u>** that you usually eat; I have a nutrition card/picture here that shows you some examples of local fruits and vegetables; Each picture represents the size of a serving; To answer these questions, please think of a typical week.(If 1-2 times/month then record it as "00.")					
225	AGD_225	In a <u>typical week</u>, on how many <u>days</u> do you **eat <u>fruit</u>**?<br><br>***Instruction:*** *Demonstrate fruit's show card and ask the participant to think about it. Do not consider packaged fruit juice but consider fruit juice prepared at home using fresh fruits. A typical week refers to "A usual week except religious event or any other festival. If 1-2 times/month then record it as "00."* [USE SHOWCARD]	Number of days<br>|____|____| 77 = Don't Know<br>[If '00' days, go to ABD_227]		
226	AGD_226	How many **<u>servings</u>** of fruit do you eat on **<u>one</u>** of those days? (USE SHOWCARD)<br><br>***Instruction:*** *Ask the participant to think about any one of those days of a typical week. Use supplied bowl and show card to measure servings.*	Number of servings<br>|___|___|.|___| 77.7 = Don't Know		
227	AGD_227	In a <u>typical week</u>, on how many <u>days</u> do you **eat <u>vegetables</u>**?<br><br>***Instruction:****Demonstrate vegetable's show card and ask the participant to think about it. Do not consider potato as vegetables. Demonstrate serving with the help of supplied cup and show card as cooked and uncooked vegetables. Consider only vegetables serving in case of cooked vegetables with fish. A typical week refers to "A usual week except religious event or any other festival. If 1-2 times/month then record it as "00."* [USE SHOWCARD]	Number of days<br>|____|____| 77 = Don't Know<br>[If '0' days, go to ABD_229]		

	Variable	Question	Yesterday during the day or night, did your child consume the following food items? (A)	During the last 07 days how many days did your child consume the following food items (B)	During the last 07 dayshow many times did your child consume the following food items (C)
228	AGD_228	How many **servings** of vegetables do you eat on one of those days?<br><br>***Instruction:*** *Ask the participant to think about any one of those days of a typical week. Use supplied cup and show card to measure serving.*[USE SHOWCARD]	Number of servings<br>|___|___|.|___| 77.7 = Don't Know		
229	AGD_229	Do you currently eat any tablet, capsul,syrup contaning vitamin/minarels	1=Yes 2=No		If "No" go to next section
230	AGD_230	Write the name of medicine by observed the prescription and medication	A.___________<br>B.___________<br>C.___________		

Section 3: Behavioral risk factor (Smoking/smokeless tobacco physical activity)

No	Variable	Question	Response Options	Code	Instructions/ Skip
**Tobacco Use**<br>Now I am going to ask you some questions about tobacco (Smoke tobacco and smokeless tobacco) use.					
301	AGR_301	Do you currently smoke any tobacco products, such as cigarettes, *bidis, hookah, cigars* or pipes?<br><br>*Instruction: Demonstrate show card to the participant and ask which smoke tobacco products/he use*. [USE SHOWCARD]	1 = Yes; 2= No<br>If No, go to ABR_304		
302	AGR_302	Do you currently smoke tobacco products daily?<br><br>*Instruction: This question is applicable for those respondents who smoke tobacco product daily.*<br>*Daily meanus" tobacco smoking is at least one time/day for almost one month or more than that*<br>*If that is the case respondant started somoking from 25 days and still running, that is regarded as everyday.*	1 = Yes; 2= No		
303	AGR_303	How old were you when you **first started** smoking?	Age |___|___| years<br>77 = Don't know		

No	Variable	Question	Response Options	Code	Instructions/ Skip
304	AGR_304	Do you currently use anysmokeless tobacco products such as Betel quid with zarda, zarda only or zarda with supari, Betel quid with sadapata, pan masala withtobacco, sadapata chewing, gul, Khoinee, Nossi, gutka?<br>***Instruction:*** *Ask respondent to think and answer whether h/she use any smokeless tobacco products such as Betel quid with zarda, zarda only or zarda with supari, Betel quid with sadapata, pan masala with tobacco, sadapata chewing, gul, Khoinee, Nossi, gutka. Here, only betel quid, supari and lime will not be considered. It will be considered as smokeless tobacco if respondent use Betel quid with zarda, zarda only or zarda with supari, Betel quid with sadapata, pan masala with tobacco, sadapata chewing, gul, Khoinee, Nossi, gutka.* [USE SHOWCARD]	1 = Yes; 2= No<br><br>If No, go to ABR_307		
305	AGR_305	Do you currently use smokeless tobacco daily?<br>***Instruction:*** *Daily means "using smokeless tobacco is at least one time/day for almost one month or more than that.*<br>*If that is the case respondant started somoking from 25 days and still running, that is regarded as everyday.*	1 = Yes; 2= No		
306	AGR_306	How old were you when you started smokeless tobacco?	Age └─┴─┘ years<br>77 = Don't know		
307	AGR_307	Which of your parents or guardians use any form of tobacco?	1= Neither<br>2= My father or male guardian<br>3= My mother or female guardian<br>4= Both<br>5= I do not know<br>99= Others (Specify)_______		

Physical activity

Next I am going to ask you about the time you spend doing different types of physical activity in a typical week. Please answer these questions even if you do not consider yourself to be a physically active person.

Think first about the time you spend doing work. Think of work as the things that you have to do such as paid or unpaid work, study/training, household chores, harvesting food/crops, fishing or hunting for food, seeking employment. [Insert other examples if needed]. In answering the following questions 'vigorous-intensity activities' are activities that require hard physical effort and cause large increases in breathing or heart rate, 'moderate-intensity activities' are activities that require moderate physical effort and cause small increases in breathing or heart rate.

Instruction: *Please read out the above statement for respondent. Do not drop this part. At first respondent will must think about his/her daily activities (Paid or unpaid work, study/training, household chores, harvesting food/crops, fishing or hunting for food, seeking employment). Then will think time for travelling from one place to another and at last time spent during leisure period.*

Do not forget to demonstrate show card to the respondent which will help them to respond.

It is needed to let participant remember when answer the following things

Vigorous intensity activity*: Such kind of physical activity that causes large increases in breathing or heart rate.*

Moderate-intensity activity: *Such kind of physical activity that causes small increases in breathing or heart rate.*

Now I would like to know about the 'Vigorous-intensity activities' you perform

'Vigorous-intensity activities' performed as a part of daily work / professional work/ outside daily work / professional work.					
308	AGR_308	Does your daily work / professional work, sports, fitness or recreational(leisure) activities involve vigorous-intensity activity that causes large increases in breathing or heart rate like [carrying or lifting heavy loads, digging or construction work, harvesting paddy, fishing using net, running, football, Kabaddi, Dariabandha, Gollachut etc.] for at least 10 minutes continuously?<br>***Instruction:*** *Ask participant to think about those vigorous-intensity activities performed as a part of professional work/ recreational activities. Those activities will be considered as vigorous-intensity activities that causes large increase in breathing or heart rate.* [USE SHOWCARD]	1 = Yes 2 = No<br>[If 'No', please go to 311]		
309	AGR_309	In a typical week, on how many days do you do vigorous- intensity activities/sports/fitness as part of daily work / professional work/ recreational activities?<br>***Instruction:*** *In a typical week refers to "activities performed by respondent in a typical week". Valid response range is 1-7 days.*	└─┴─┘ Day<br>77 = Don't know<br>[If 'Don't know', please go to ABR_311]		
310	AGR_310	How much time do you spend doing vigorous-intensity activities/sports/fitness at daily work / professional work/recreational activities on a typical day?<br>***Instruction:*** *Ask respondent to think about a single day (which can be remembered easily) s/he performed vigorous-intensity activities as part of professional work/ recreational activities. Respondent will consider only those activities that are performed for at least 10 minutes or more continuously. Check unusual (more than 04 hours) values.*	________<br>Minutes		
Now I would like to know about the 'Moderate-intensity activity' you perform					
'Moderate-intensity activity' performed as a part of daily work / professional work					
311	AGR_311	Does your daily work / professional work/ spots/fitness/ recreational (leisure) work involve moderate-intensity activity that causes small increases in breathing or heart rate such as brisk walking [or carrying light loads, cycling, swimming, volleyball] for at least 10 minutes continuously?<br>***Instruction:*** *Ask participant to think about those moderate-intensity activities performed as a part of professional work/ recreational activities. Those activities will be considered as moderate-intensity activities that causes small increase in breathing or heart rate.* [USESHOWCARD]	1 = Yes 2 = No<br>[If 'No', please go to ABR_314]		
312	AGR_312	In a typical week, on how many days do you do moderate- intensity activities as part of your daily work / professional work/sports/fitness or recreational work?<br>***Instruction:*** *In a typical week refers to "activities done by respondent in a typical week". Valid response range is 1-7 days.*	└─┴─┘ Day<br>77 = Don't know<br>[If 'Don't know', please go to ABR_314]		

313	AGR_313	How much time do you spend doing moderate-intensity activities at daily work / professional work/ sports/fitness or recreational work on a typical day?<br>***Instruction:*** *Ask respondent to think about a single day (which can be remembered easily) s/he performed moderate-intensity activities as part of professional work/recreational activities. Respondent will consider only those activities that are performed for at least 10 minutes or more continuously. Check unusual (more than 04 hours) values.*	________<br>Minutes		
**Sedentary behavior** The following question is about sitting or reclining at work, at home, getting to and from places, or with friends including time spent sitting at a desk, sitting with friends, traveling in car, bus, train, reading, playing cards or watching television, but do not include time spent sleeping.					
314	AGR_314	How much time do you usually spend sitting or reclining on a typical day?<br>***Instruction:*** *Ask the respondent to think about time spent on sitting or reclining at work, office, study, watching television, computer use, at the kitchen, during rest.Do not include time spent during sleeping.*	________<br>Minutes		
315	AGR_315	How much time do you watch television daily?	________<br>Minutes		

Section 4: Reproductive history, menstrual hygiene

No	Variable	Question	Response Options	Code	Instructions/ Skip
401	AGM_401	When your menarche started?	|__|__| years		Write "00" if not started yet
402	AGM_402	What is your marital status?	1 = Never married<br>2 = Currently married<br>3 = Separated;<br>4 = Divorced<br>5 = Widowed;<br>88 = Refused		If answer 1, go to 40
403	AGM_403	How many times you became pregnant in your life? (Including abortion, MR, stillbirth and live birth)?	|__|__| Times		If "0" go to section 5
404	AGM_404	Are you pregnant now?	1 = Yes 2=No<br>8=Unsure		
Menstrual Hygiene (Please ask the following questions if menarche/period started, if not then skip to anthropometry section)					
405	AGM_405	What material do you use during period/menstruation? Did you	1= Sanitary pad<br>2= Old cloth<br>3= New Cloth<br>99= Others(Specify)_____		

Section5: Health Care(Ask these questions only for married women)

No	Variable	Question	Response Options	Code	Instructions
501	AGH_501	Did You deliver any live birth or stillbirth in the last 24 months?	1=Yes 2=No		If "No" go to section 6
502	AGH_502	Date of delivery of live birth or still birth.	|___|___|/|___|___|/20|___|___| Day/Month/Year		

No	Variable	Question	Response Options	Code	Instructions
503	AGH_503	Does your child is living now ?	1=Yes 2=No<br>88=Not applicable		
504	AGH_504	Have you been registered as a pregnant woman when you were pregnant?	1=Yes 2=No		
505	AGH_505	How many times you took ante natal care (ANC) when you were pregnant?	|____|____|Times		
506	AGH_506	From whom you usually took the ANC service when you were pregnant?<br><br>(may be multiple answer)	1 =Passed doctor<br>2 =Nurses/Midwives/Paramedics<br>3 = FWV<br>4 = Medical assistant/Sub assistant<br>5 = NGO health worker<br>6 = Skilled midwife<br>7 = Trained conventional midwife<br>8 = Conventional midwife<br>77 =Don't know<br>99 = Others (specify bellow)_____		
507	AGH_507	Have you been measured your weight when you were pregnant?	1=Yes 2=No		
508	AGH_508	Have you eaten Iron and Folic acid tablet when you were pregnant?	1=Yes 2=No		If" No" go to 512
509	AGH_509	From where you got Iron and Folic acid tablet when you were pregnant?<br><br>(may be multiple answer)	**GOVERNMENT PROVIDERS**<br>1= Govt. Field worker<br>2= Govt. satellite clinic/ EPI Outreach clinic<br>3= Govt. Community clinic<br>4=Govt. Health & Family Welfare Centre<br>5=Govt. Upazila Health Complex<br>6= Govt. Maternal & Child Welfare Centre Government<br>7=Other government Hospital<br>**NGO PROVIDERS**<br>8= NGO health clinic and hospital<br>9=NGO Field Worker<br>10= Community Nutrition Promoter (CNP)<br>**PRIVATE PROVIDERS**<br>11 = Private doctor/clinic/hospital<br>12 = Pharmacy<br>13 = Traditional healer<br>14 = Homeopath<br>17=Don't know<br>99 = Others (Specify)___________		
510	AGH_510	How many months did you consume Iron and Folic acid tablet when you were pregnant?	|____|____|Months		
511	AGH_511	How many tablet of Iron and Folic acid did you consume in a week when you were pregnant?	|____|____|Tablet		
512	AGH_512	Did you consume calciam tablet whwen you were pregnant?	1=Yes 2=No		If" No" go to 516
513	AGH_513	From where you got Calciam tablet when you were pregnant?<br><br>(may be multiple answer)	**GOVERNMENT PROVIDERS**<br>1= Govt. Field worker<br>2= Govt. satellite clinic/ EPI Outreach clinic<br>3= Govt. Community clinic<br>4=Govt. Health & Family Welfare<br>**NGO PROVIDERS**<br>8= NGO health clinic and hospital<br>9=NGO Field Worker<br>10= Community Nutrition Promoter (CNP)<br>**PRIVATE PROVIDERS**<br>11 = Private doctor/clinic/hospital<br>12 = Pharmacy<br>13 = Traditional healer		

No	Variable	Question	Response Options		Code	Instructions
			Centre<br>5=Govt. Upazila Health Complex<br>6= Govt. Maternal & Child Welfare Centre Government<br>7=Other government Hospital	14 = Homeopath<br>17=Don't know<br>99 = Others (Specify)___________		
514	AGH_514	How many months did you consume Calciam tablet when you were pregnant?	|____|____|Months			
515	AGH_515	How many tablet of Calciam did you consume in a week when you were pregnant?	|____|____|Tablet			
516	AGH_516	Have any doctor or any other health care provider gave you nutrition related counseling when you were pregant?	1=Yes 2=No			
517	AGH_517	Advice on what matters ?	1 =Take enough food<br>2 =Enough rest<br>3 =Category of nutritional food<br>4 =Rules of iron and Folic acid tabet intake<br>5 =Rules of Calciam tablet intake			
If stillbirth, need not to ask following questions.						
518	AGH_518	Had your child been measured birth weight within 3 days after birth?	1=Yes 2=No<br>88=Not applicable			If not applicable go to next section
519	AGH_519	What was your child's birth weight?	|____|.|____|____|____| kg			
520	AGH_520	Afterwards had any doctor or any health care provider took your child's weight?	1=Yes 2=No<br>88=Not applicable			
521	AGH_521	Do you have any charts for your child's weight for age?	1=Yes 2=No			
522	AGH_522	After your baby is born, Have any doctor or any other health care provider gave you nutrition related counseling?	1=Yes 2=No			
523	AGH_523	Advice on what matters ?	1 =Take enough food<br>2 =Enough rest<br>3 =Category of nutritional food<br>4 =Rules of iron and Folic acid tabet intake<br>5 =Rules of Calciam tablet intake	6=Exclusive breastfeeding at first 6 months<br>7=Continue breastfeeding up to 2 years age<br>8=Rules of supplementary food intake<br>9=The rules for feeding the child at the time of illness<br>10=Do not feed the store food		

Section6: Mental Health

No	Variable	Question	Response Options	Code	Instructions
Over the last 2 weeks how often you have faced the following problems?					
601	AGM_601	Less interest or less joy to do something	0=Not at all<br>1=Some time<br>2=Most of the time<br>3= All time		
602	AGM_602	Feeling Upset, depressed,irritable,hopeless	0=Not at all<br>1=Some time<br>2=Most of the time<br>3= All time		

No	Variable	Question	Response Options	Code	Instructions
Over the last 2 weeks how often you have faced the following problems?					
603	AGM_603	Difficulty to sleeping or sleeping too much	0=Not at all<br>1=Some time<br>2=Most of the time<br>3= All time		
604	AGM_604	Tired or low strength	0=Not at all<br>1=Some time<br>2=Most of the time<br>3= All time		
605	AGM_605	Loss of appetite,weight loss or eat more	0=Not at all<br>1=Some time<br>2=Most of the time<br>3= All time		
606	AGM_606	Have a bad feeling about self, feel self as a failure, Making myself or my family smaller feel like this	0=Not at all<br>1=Some time<br>2=Most of the time<br>3= All time		
607	AGM_607	Difficulty to concentrate at the time of work at school,read something or watch television	0=Not at all<br>1=Some time<br>2=Most of the time<br>3= All time		
608	AGM_608	Talk so lightly or move around that others might notice or the opposite of this that means more severity or more movement than usual.	0=Not at all<br>1=Some time<br>2=Most of the time<br>3= All time		
609	AGM_609	It is better to die or hit yourself in any way feel like this.	0=Not at all<br>1=Some time<br>2=Most of the time<br>3= All time		If "0" go to section 5

Referral

No.	Variable	Question	Responses and code categories	Code	Instructions/Skip
610	AGM_610	Referral needed?	1 = Yes 2 = No		If 2, go to section 7
611	AGM_611	Where referred?	1 = Upazila Health Complex<br>2 = District Hospital<br>3= Medical College Hospital<br>4= Doctor's private chamber<br>99 = Others (Specify)_____________		

Section 7: Anthropometry

No	Variable	Question	Responses and code categories	Code	Instructions
701	AGA_701	Start Time of anthropometry	|____|____| : |____|____| Hr : Min		Use 24 hr format
702	AGA_702	Weight Scale ID	|____|____|		
703	AGA_703	Height Scale ID	|____|____|		
704	AGA_704	Clothing type during weight measurement	1 = Light clothing<br>2 = Slightly heavy clothing<br>3 = Heavy clothing		
705	AGA_705	Name of measurer			
706	AGA_706	Code of measurer	|____|____|		

No	Variable	Measurement	Measurement 1	Measurement 2	Measurement 3

<table>
<tr><th>No</th><th>Variable</th><th>Question</th><th colspan="2">Responses and code categories</th><th>Code / Instructions</th></tr>
<tr><td>707</td><td>AGA_707A /B/C</td><td>Height (Cm)</td><td>A.|____|____|____|.|____|</td><td>B.|____|____|____|.|____|</td><td>C.|____|____|____|.|____|
If A and B differs more than 0.5 cm, take 3rd measurement</td></tr>
<tr><td>708</td><td>AGA_708A /B/C</td><td>Weight (kg)</td><td>A.|____|____|____|.|____|</td><td>B.|____|____|____|.|____|</td><td>C.|____|____|____|.|____|
If A and B differs more than 0.1kg, take 3rd measurement</td></tr>
<tr><td>709</td><td>AGA_709A /B/C</td><td>Fat%</td><td>A.|____|____|____|.|____|</td><td>B.|____|____|____|.|____|</td><td></td></tr>
<tr><td>710</td><td>AGA_710A /B/C</td><td>Water%</td><td>A.|____|____|____|.|____|</td><td>B.|____|____|____|.|____|</td><td></td></tr>
<tr><td>711</td><td>AGA_711A /B/C</td><td>Waist circumfer ence (Cm)</td><td>A.|____|____|.|____|</td><td>B.|____|____|.|____|</td><td>C.|____|____|.|____|
If A and B differs more than 0.5 cm, take 3rd measurement</td></tr>
<tr><td>712</td><td>AGA_712</td><td>Comment</td><td colspan="3"></td></tr>
<tr><td>713</td><td>AGA_713</td><td>End Time of measurement</td><td colspan="2">|____|____|:|____|____|
Hr : Min</td><td>Use 24 hr format</td></tr>
</table>

Module 6: Adult Men Module

Section 1: General Information

No	Variable	Question	Response Options	Code	Instructions/ Skip
101	AM_101	What's your name?(with nick name)	________________________		
102	AM_102	What's your date of birth	|____|____|/|____|____|/20|____|____| DD / MM / YYYY		
103	AM_103	Age of the respondent (Write complete year)	|____|____| year		
104	AM_104	What is your marital status?	1 = Never married<br>2 = Currently married<br>3 = Separated<br>4 = Divorced<br>5 = Widowed<br>88 = Refused		
105	AM_105	Completed years of education the respondent completed<br><br>***[Instruction:** Write down in total, the years the respondent spent in school for formal education (Is completed years) [Excluding pre-school and informal education). Write down "00" if no formal education.]*	|____|____| Years<br>• Primary/Ebtedayee=5<br>• Secondary/Dakhil=10<br>• Higher secondary/ Diploma/Alim=12<br>• Graduate/Fazil=16<br>• Post graduate/kamil/dawra=18		
106	AM_106	Occupation of the respondent over the past 12 months<br>If the participant has been employed more than one occupation in the last 12 months, consider the profession that he has spent most of his time as the main occupation and write it down	1 = Agricultural work(paddy)<br>2 = Agricultural work(without paddy)<br>3 = Agricultural day labor<br>4 = Un skild day labor<br>5 = Skiled day labor<br>6= Driver of Rickshaw/ van/Wheel barrow/Baby taxi/ Boatman<br>7=Fisherman<br>8=Employer<br>9=Profesionals<br>10=Businessman<br>11= Petty businessman<br>12= House maid<br>13=Zoom fermar<br>14= Do not earn<br>15=Poultry/dairy fermar<br>16=Handicrafts<br>17 =Spinach vegitable<br>18=Fishery<br>19 = Homemaker<br>20= Student<br>77 = Don,t know<br>66 =Age below 06 years		
107	AM_107	Completed years of education of the mother of the respondent<br><br>***[Instruction:** Write down in total, the years the respondent spent in school for formal*	|__|__| Years<br>• Primary/Ebtedayee=5<br>• Secondary/Dakhil=10<br>• Higher Secondary/Diploma/Alim=12<br>• Graduate/Fazil=16		

No	Variable	Question	Response Options	Code	Instructions/ Skip
		education (Is completed years) [Excluding pre-school and informal education). Write down "00" if no formal education.]	• Post graduate/kamil/dawra=18		
108	AM_108	Mother's occupation of the respondent over the past 12 months<br>If the participant has been employed more than one occupation in the last 12 months, consider the profession that he has spent most of his time as the main occupation and write it down	1 = Agricultural work(paddy)<br>2 = Agricultural work(without paddy)<br>3 = Agricultural day labor<br>4 = Un skild day labor<br>5 = Skiled day labor<br>6= Driver of Rickshaw/ van/Wheel barrow/Baby taxi/ Boatman<br>7=Fisherman<br>8=Employer<br>9=Profesionals<br>10=Businessman<br>11= Petty businessman<br>12= House maid<br>13=Zoom fermar<br>14= Do not earn<br>15=Poultry/dairy fermar<br>16=Handicrafts<br>17 =Spinach vegitable<br>18=Fishery<br>19 = Homemaker<br>20= Student<br>77 = Don,t know<br>66 =Age below 06 years		
109	AM_109	Completed years of education of the father of the respondent<br>***[Instruction:** Write down in total, the years the respondent spent in school for formal education (Is completed years) [Excluding pre-school and informal education). Write down "00" if no formal education.]*	|__|__| Years<br>• Primary/Ebtedayee=5<br>• Secondary/Dakhil=10<br>• Higher/ Secondary/ Diploma/Alim=12<br>• Graduate/Fazil=16<br>• Post graduate/kamil/dawra=18		
110	AM_110	Father's occupation of the respondent over the past 12 months<br>If the participant has been employed more than one occupation in the last 12 months, consider the profession that he has spent most of his time as the main occupation and write it down	1 = Agricultural work(paddy)<br>2 = Agricultural work(without paddy)<br>3 = Agricultural day labor<br>4 = Un skild day labor<br>5 = Skiled day labor<br>6= Driver of Rickshaw/ van/Wheel barrow/Baby taxi/ Boatman<br>7=Fisherman<br>8=Employer<br>9=Profesionals<br>10=Businessman<br>11= Petty businessman<br>12= House maid<br>13=Zoom fermar<br>14= Do not earn<br>15=Poultry/dairy fermar<br>16=Handicrafts<br>17 =Spinach vegitable		

No	Variable	Question	Response Options	Code	Instructions/ Skip
			18=Fishery<br>19 = Homemaker<br>20= Student<br>77 = Don,t know<br>66 =Age below 06 years		

Section 2: Dietary diversity

	Variable		Question	Yesterday during the day or night, did your child consume the following food items? (A)	During the last 07 days how many days did your child consume the following food items (B)	During the last 07 dayshow many times did your child consume the following food items (C)
Now I requst you to describe the food that you have eaten at home and out of home during the day or night(yesterday morning 6.00 am to today morning 6.00 am) yesterday. Please tell all types of food, drinks that you have eaten with morning, noon and dinner, or have breakfast / light breakfast. Tell us by think if you ate any food while preparing food.<br><br>Get started with what you ate yesterday morning.<br>-What did you eat in the morning wake up? Did you eat anything more?<br>-What did you eat more in the morning? Did you eat anything more?<br>-What did you eat at noon? Did you eat anything more?<br>-What did you eat at afternoon? Did you eat anything more?<br>-What did you eat at evening? Did you eat anything more?<br>-What did you eat at dinner? Did you eat anything more?<br>**Record first the answer of the questions of column A. collect the answer for column B/C after recording the answer of the questions of column A.**						
		Food Category	Examples of foods	A	B	C
201	AMD_201 A/B/C	Starchy Staples/ Food made from grains	Rice,flour bread, wheat, muri, maize,kichuri,barli,oot,kinoya,noodles,pasta	1 = Yes<br>2 = No	|____|____| days	|____|____| times
202	AMD_202 A/B/C	White roots and tubers and plantains	potatoes, sweet potatoes,sagu, erarut,cave,shalgam,kuchu,wiggle,ripe banana shaloo	1 = Yes<br>2 = No	|____|____| days	|____|____| times
203	AMD_203 A/B/C	Pulses	Dal/khicuri,peas,motor,soybean,tofu peanuts, humas	1 = Yes<br>2 = No	|____|____| days	|____|____| times
204	AMD_204 A/B/C	Legumes and Nuts	Peanut, Pistachios, kazu or any kond of peanut, ciya seeds, til, tisi,sunflower seeds,sweet pumpkin seeds	1 = Yes<br>2 = No	|____|____| days	|____|____| times
205	AMD_205 A/B/C	Dark green leafy vegetables	All kinds of leafy vegetables (poo,kuchu, kalmi) Brooklyn	1 = Yes<br>2 = No	|____|____| days	|____|____| times

	Variable	Question		Yesterday during the day or night, did your child consume the following food items? (A)	During the last 07 days how many days did your child consume the following food items (B)	During the last 07 dayshow many times did your child consume the following food items (C)
206	AMD_2 06 A/B/C	Red/orange/yellow vegetables	Orange sweet potato,pumpkin, carrot or other yellow or orange vegetable	1 = Yes 2 = No	|____|____ | days	|____|____ | times
207	AMD_2 07 A/B/C	Red/orange/yellow fruits	Ripe mangoes, papaya, jackfruits other red/yellow or orange fruit	1 = Yes 2 = No	|____|____ | days	|____|____ | times
208	AMD_2 08 A/B/C	Vitamin C rich fruits	Guava, Strawberry, Lemon, Orange,grapes, Pineapple,raw mango,amalaki,kueue,tomatos	1 = Yes 2 = No	|____|____ | days	|____|____ | times
209	AMD_2 09 A/B/C	Vitamin C rich vegetables	Raw tomato,chilli pepper, brussels sprouts,cauliflower,cabbage	1 = Yes 2 = No	|____|____ | days	|____|____ | times
210	AMD_2 10 A/B/C	Other vegetables or fruits	peas, asparagas, bit,koci bash, cauliflower,selari,shawsha,brinjal,letus, masrum,mula,jukini	1 = Yes 2 = No	|____|____ | days	|____|____ | times
211	AMD_2 11 A/B/C	Other fruits	Apple, avakado,bam, cherry,ripe jackfruit	1 = Yes 2 = No	|____|____ | days	|____|____ | times
212	AMD_2 12 A/B/C	Eggs	Hen/duck, other birds,	1 = Yes 2 = No	|____|____ | days	|____|____ | times
213	AMD_2 13 A/B/C	Organ meat	Cheeks, liver, Stomach, Ridpind, Kidney	1 = Yes 2 = No	|____|____ | days	|____|____ | times
214	AMD_2 14 A/B/C	Flesh	Beef, Pork, Veal, Lamb, Goat, Chicken, Duck or flesh of any other animal	1 = Yes 2 = No	|____|____ | days	|____|____ | times
215	AMD_2 15 A/B/C	Small Fish	Small Fish Eaten Whole with Bones (i.e.Kachki, mola, dhela, chapila, batashi, small prawn, dried fish)	1 = Yes 2 = No	|____|____ | days	|____|____ | times
216	AMD_2 16 A/B/C	Large fish/sea food	Large Whole Fish and Shell Fish and dried fish	1 = Yes 2 = No	|____|____ | days	|____|____ | times
217	AMD_2 17 A/B/C	Dairy	Milk, cheese, yogurt or other milk products	1 = Yes 2 = No	|____|____ | days	|____|____ | times
218	AMD_2	Insects and other	Fish egg, insect, snail	1 = Yes	|____|____	|____|____

	Variable	Question		Yesterday during the day or night, did your child consume the following food items? (A)	During the last 07 days how many days did your child consume the following food items (B)	During the last 07 dayshow many times did your child consume the following food items (C)
	18	protein foods		2 = No	| days	| times
219	AMD_219 A/B/C	Edible Oil	Ghee, butter,cream, sour, fat, margarine, mayonnaise, palm oil, vegetable oil,	1 = Yes 2 = No	|____|____ | days	|____|____ | times
220	AMD_220 A/B/C	Savory and fried snacks	Crisps and chips, fried dough or other fried snacks singaru, samarcha	1 = Yes 2 = No	|____|____ | days	|____|____ | times
221	AMD_221 A/B/C	Sweets	Sugary foods, such as chocolates, candies, cookies/sweet biscuits and cakes, sweet pastries or ice cream, any kind of sweets ,honey ,halua , condensed milk,tiler khaza	1 = Yes 2 = No	|____|____ | days	|____|____ | times
222	AMD_222 A/B/C	Miscellaneous (Sweet drinks)	Tea with suger, soft drinks, juice, energy drinks,yogurt drinks,chocolate drinks, horlicks,moltova	1 = Yes 2 = No	|____|____ | days	|____|____ | times
223	AMD_223 A/B/C	Other beverages and foods	Tea or coffee if not sweetened, clear broth, alcohol, Pickles, olives and similar	1 = Yes 2 = No	|____|____ | days	|____|____ | times
224	AMD_224 A/B/C	Condiments/spices	Spices, coriander leaves, sausage, garlic, ketchup, lemon juice, mint leaves, drinks, betel leaves, tobacco leaves, jars	1 = Yes 2 = No	|____|____ | days	|____|____ | times

No	Variable	Question	Response Options	Code	Instructions/ Skip
The next questions I will ask about the **fruits** and **vegetables** that you usually eat; I have a nutrition card/picture here that shows you some examples of local fruits and vegetables; Each picture represents the size of a serving; To answer these questions, please think of a typical week.(If 1-2 times/month then record it as “00.”)					
225	AMD_225	In a typical week, on how many days do you **eat fruit**?<br><br>***Instruction:*** *Demonstrate fruit’s show card and ask the participant to think about it. Do not consider packaged fruit juice but consider fruit juice prepared at home using fresh fruits. A typical week refers to “A usual week except religious event or any other festival. If 1-2 times/month then record it as “00.”* [USE SHOWCARD]	Number of days<br>|____|____| 77 = Don't Know<br>[If ‘00’ days, go to ABD_227]		

	Variable	Question	Yesterday during the day or night, did your child consume the following food items? (A)	During the last 07 days how many days did your child consume the following food items (B)	During the last 07 dayshow many times did your child consume the following food items (C)
226	AMD_226	How many **servings** of fruit do you eat on **one** of those days? (USE SHOWCARD)<br><br>***Instruction:*** *Ask the participant to think about any one of those days of a typical week. Use supplied bowl and show card to measure servings.*	Number of servings<br>|___|___|.|___| 77.7 = Don't Know		
227	AMD_227	In a typical week, on how many days do you **eat vegetables**?<br><br>***Instruction:****Demonstrate vegetable's show card and ask the participant to think about it. Do not consider potato as vegetables. Demonstrate serving with the help of supplied cup and show card as cooked and uncooked vegetables. Consider only vegetables serving in case of cooked vegetables with fish. A typical week refers to "A usual week except religious event or any other festival. If 1-2 times/month then record it as "00."* [USE SHOWCARD]	Number of days<br>|____|____| 77 = Don't Know<br>[If '0' days, go to ABD_229]		
228	AMD_228	How many **servings** of vegetables do you eat on one of those days?<br><br>***Instruction:*** *Ask the participant to think about any one of those days of a typical week. Use supplied cup and show card to measure serving.*[USE SHOWCARD]	Number of servings<br>|___|___|.|___| 77.7 = Don't Know		
229	AMD_229	Do you currently eat any tablet, capsul,syrup contaning vitamin/minarels	1=Yes 2=No		If "No" go to next section
230	AMD_230	Write the name of medicine by observed the prescription and medication	A.___________<br>B.___________<br>C.___________		

Section 3: Behavioral risk factor (Smoking/smokeless tobacco physical activity)

No	Variable	Question	Response Options	Code	Instructions/ Skip
**Tobacco Use**<br>Now I am going to ask you some questions about tobacco (Smoke tobacco and smokeless tobacco) use.					

No	Variable	Question	Response Options	Code	Instructions/ Skip
301	AMR_301	Do you <u>currently</u> smoke any tobacco products, such as cigarettes, *bidis, hookah, cigars* or pipes?<br><br>*Instruction: Demonstrate show card to the participant and ask which smoke tobacco products/he use*. [USE SHOWCARD]	1 = Yes; 2= No<br>If No, go to ABR_304		
302	AMR_302	Do you <u>currently</u> smoke tobacco products <u>daily</u>?<br><br>*Instruction: This question is applicable for those respondents who smoke tobacco product daily.*<br>*Daily meanus" tobacco smoking is at least one time/day for almost one month or more than that*<br>*If that is the case respondant started somoking from 25 days and still running, that is regarded as everyday.*	1 = Yes; 2= No		
303	AMR_303	How old were you when you **first started** smoking?	Age └─┴─┘ years<br>77 = Don't know		
304	AMR_304	Do you currently use anysmokeless tobacco products such as Betel quid with zarda, zarda only or zarda with supari, Betel quid with sadapata, pan masala withtobacco, sadapata chewing, gul, Khoinee, Nossi, gutka?<br>***Instruction:*** *Ask respondent to think and answer whether h/she use any smokeless tobacco products such as Betel quid with zarda, zarda only or zarda with supari, Betel quid with sadapata, pan masala with tobacco, sadapata chewing, gul, Khoinee, Nossi, gutka. Here, only betel quid, supari and lime will not be considered. It will be considered as smokeless tobacco if respondent use Betel quid with zarda, zarda only or zarda with supari, Betel quid with sadapata, pan masala with tobacco, sadapata chewing, gul, Khoinee, Nossi, gutka.* [USE SHOWCARD]	1 = Yes; 2= No<br><br>If No, go to ABR_307		
305	AMR_305	Do you <u>currently</u> use <u>smokeless tobacco daily</u>?<br>***Instruction:*** *Daily means "using smokeless tobacco is at least one time/day for almost one month or more than that.*<br>*If that is the case respondant started somoking from 25 days and still running, that is regarded as everyday.*	1 = Yes; 2= No		
306	AMR_306	How old were you when you started smokeless tobacco?	Age └─┴─┘ years<br>77 = Don't know		

No	Variable	Question	Response Options	Code	Instructions/ Skip
307	AMR_307	Which of your parents or guardians use any form of tobacco?	1= Neither<br>2= My father or male guardian<br>3= My mother or female guardian<br>4= Both<br>5= I do not know<br>99= Others (Specify)_______		
**Physical activity**					
Next I am going to ask you about the time you spend doing different types of physical activity in a typical week. Please answer these questions even if you do not consider yourself to be a physically active person.<br>Think first about the time you spend doing work. Think of work as the things that you have to do such as paid or unpaid work, study/training, household chores, harvesting food/crops, fishing or hunting for food, seeking employment. [Insert other examples if needed]. In answering the following questions 'vigorous-intensity activities' are activities that require hard physical effort and cause large increases in breathing or heart rate, 'moderate-intensity activities' are activities that require moderate physical effort and cause small increases in breathing or heart rate.<br>***Instruction:*** *Please read out the above statement for respondent. Do not drop this part. At first respondent will must think about his/her daily activities (Paid or unpaid work, study/training, household chores, harvesting food/crops, fishing or hunting for food, seeking employment). Then will think time for travelling from one place to another and at last time spent during leisure period.*<br>*Do not forget to demonstrate show card to the respondent which will help them to respond.*<br>*It is needed to let participant remember when answer the following things*<br>***Vigorous intensity activity****: Such kind of physical activity that causes large increases in breathing or heart rate.*<br>***Moderate-intensity activity:*** *Such kind of physical activity that causes small increases in breathing or heart rate.*					
Now I would like to know about the 'Vigorous-intensity activities' you perform					
'Vigorous-intensity activities' performed as a part of daily work / professional work/ outside daily work / professional work.					
308	AMR_308	Does your daily work / professional work, sports, fitness or recreational(leisure) activities involve vigorous-intensity activity that causes large increases in breathing or heart rate like [carrying or lifting heavy loads, digging or construction work, harvesting paddy, fishing using net, running, football, Kabaddi, Dariabandha, Gollachut etc.] for at least 10 minutes continuously?<br>***Instruction:*** *Ask participant to think about those vigorous-intensity activities performed as a part of professional work/ recreational activities. Those activities will be considered as vigorous-intensity activities that causes large increase in breathing or heart rate.* [USE SHOWCARD]	1 = Yes 2 = No<br>[If 'No', please go to 311]		
309	AMR_309	In a typical week, on how many days do you do vigorous- intensity activities/sports/fitness as part of daily work / professional work/ recreational activities?<br>**Instruction:** *In a typical week refers to "activities performed by respondent in a typical week". Valid response range is 1-7 days.*	|__|__| Day<br>77 = Don't know<br>[If 'Don't know', please go to ABR_311]		
310	AMR_310	How much time do you spend doing vigorous-intensity activities/sports/fitness at daily work / professional work/recreational activities on a typical day?<br>***Instruction:*** *Ask respondent to think about a single day (which can be remembered easily) s/he performed vigorous-intensity activities as part of professional work/ recreational activities. Respondent will consider only those activities that are performed for at least 10 minutes or more continuously. Check unusual (more than 04 hours) values.*	________<br>Minutes		

Now I would like to know about the 'Moderate-intensity activity' you perform					
'Moderate-intensity activity' performed as a part of daily work / professional work					
311	AMR_311	Does your daily work / professional work/ spots/fitness/ recreational (leisure) work involve moderate-intensity activity that causes small increases in breathing or heart rate such as brisk walking [or carrying light loads, cycling, swimming, volleyball] for at least 10 minutes continuously?<br>***Instruction:*** *Ask participant to think about those moderate-intensity activities performed as a part of professional work/ recreational activities. Those activities will be considered as moderate-intensity activities that causes small increase in breathing or heart rate.* [USESHOWCARD]	1 = Yes 2 = No<br>[If 'No', please go to ABR_314]		
312	AMR_312	In a typical week, on how many days do you do moderate- intensity activities as part of your daily work / professional work/sports/fitness or recreational work?<br>***Instruction:*** *In a typical week refers to "activities done by respondent in a typical week". Valid response range is 1-7 days.*	└─┴─┘ Day<br>77 = Don't know<br>[If 'Don't know', please go to ABR_314]		
313	AMR_313	How much time do you spend doing moderate-intensity activities at daily work / professional work/ sports/fitness or recreational work on a typical day?<br>***Instruction:*** *Ask respondent to think about a single day (which can be remembered easily) s/he performed moderate-intensity activities as part of professional work/recreational activities. Respondent will consider only those activities that are performed for at least 10 minutes or more continuously. Check unusual (more than 04 hours) values.*	________<br>Minutes		
**Sedentary behavior** The following question is about sitting or reclining at work, at home, getting to and from places, or with friends including time spent sitting at a desk, sitting with friends, traveling in car, bus, train, reading, playing cards or watching television, but do not include time spent sleeping.					
314	AMR_314	How much time do you usually spend sitting or reclining on a typical day?<br>***Instruction:*** *Ask respondent to think about time spent on sitting or reclining at work, office, study, watching television, computer use, at kitchen, during rest.Do not include time spent during sleeping.*	________<br>Minutes		
315	AMR_315	How much time do you watch television daily?	________<br>Minutes		

Section 4: Chronic Disease

No	Variable	Question	Response Options	Code	Instructions/ Skip
401	MCD_401	Has a health care provider ever told you that you have high blood pressure also called hypertension (other than during pregnancy)?	1 = Yes 2 = No		
402	MCD_402	Has a health care provider ever told you that you have heart disease?	1 = Yes 2 = No		
403	MCD_403	Has a health care provider ever told you that you have asthma/bronchitis?	1 = Yes 2 = No		

No	Variable	Question	Response Options	Code	Instructions/ Skip
404	MCD_404	Has a health care provider ever told you that you have Kidney Disease?	1 = Yes 2 = No		New
405	MCD_405	Has a health care provider ever told you that you have Diabetes?	1 = Yes 2 = No		New
406	MCD_406	Has a health care provider ever told you that you have stroke?	1 = Yes 2 = No		New
407	MCD_407	Has a health care provider ever told you that you have cancer?	1 = Yes 2 = No		New
408	MCD_408	Has a health care provider ever told you that you have mental problem?	1 = Yes 2 = No		New

Section 5: Blood Pressure

No.	Variable	Question	Responses and code categories		Instructions/Skip
501	MBP_501	Start Time of measuring blood pressure	|____|____| : |____|____| Hr : Min		Use 24 hr format
**Measurement -1** (Take the first measurement considering at least 15 minutes rest)					
**No.**	**Variable**	**Measurement Name**	**Systolic**	**Diastolic**	**Time of Measurement**
502	MBP_502A/ B/C	Blood Pressure (mm of Hg)	A.|___|___|___|	B.|___|___|___|	C.|____|____|:|____|____| Hr : Min
**Measurement - 2** (Allow 3 minutes' interval between two measurements)					
			**Systolic**	**Diastolic**	**Time of Measurement**
503	MBP_503 A/B/C	Blood Pressure (mm of Hg)	A.|___|___|___|	B.|___|___|___|	C.|____|____|:|____|____| Hr : Min
**Measurement - 3 (**If difference of between 1st and 2nd measurement is more than 10 mm of Hg, then take 3rd measurement for Blood Pressure)					
			**Systolic**	**Diastolic**	**Time of Measurement**
504	MBP_504 A/B/C	Blood Pressure (mm of Hg)	A.|___|___|___|	B.|___|___|___|	C.|____|____|:|____|____| Hr : Min
505	MBP_505	Blood pressure measuring machine ID	|____|____|		
506	MBP_506	Measurer's Name and Code	|____|____|		Write your full name and code
507	MBP_507	End Time of measuring blood pressure	|____|____| : |____|____| Hr : Min		Use 24 hr format
**[During any measurement, If Systolic BP ≥ 140 mm of Hg, Diastolic BP ≥ 90 mm of Hg - refer the respondent to appropriate health center]**					

Referral:

No.	Variable	Question	Responses and code categories	Code	Instructions/Skip
508	MBP_508	Referral needed?	1 = Yes 2 = No		If 2, go to section 6
509	MBP_509	Where referred?	1 = Upazila Health Complex<br>2 = District Hospital<br>3= Medical College Hospital<br>4= Doctor's private chamber<br>99 = Others (Specify)_______________		

Section 6: Anthropometry

No	Variable	Question	Responses and code categories	Code	Instructions
601	AMA_601	Start Time of anthropometry	|____|____| : |____|____| Hr : Min		Use 24 hr format
602	AMA_602	Weight Scale ID	|____|____|		
603	AMA_603	Height Scale ID	|____|____|		

No	Variable	Question	Responses and code categories	Code	Instructions
604	AMA_604	Clothing type during weight measurement	1 = Light clothing<br>2 = Slightly heavy clothing<br>3 = Heavy clothing		
605	AMA_605	Name of measurer			
606	AMA_606	Code of measurer	|___|___|		

No	Variable	Measurement	Measurement 1	Measurement 2	Measurement 3
607	AMA_607A /B/C	Height (Cm)	A.|___|___|___|.|___|	B.|___|___|___|.|___|	C.|___|___|___|.|___| If A and B differs more than 0.5 cm, take 3rd measurement
608	AMA_608A /B/C	Weight (kg)	A.|___|___|___|.|___|	B.|___|___|___|.|___|	C.|___|___|___|.|___| If A and B differs more than 0.1kg, take 3rd measurement
609	AMA_609A /B/C	Fat%	A.|___|___|___|.|___|	B.|___|___|___|.|___|	
610	AMA_610A /B/C	Water%	A.|___|___|___|.|___|	B.|___|___|___|.|___|	
611	AMA_611A /B/C	Waist circumference (Cm)	A.|___|___|.|___|	B.|___|___|.|___|	C.|___|___|.|___| If A and B differs more than 0.5 cm, take 3rd measurement
612	AMA_612	Comment			
613	AMA_613	End Time of measurement	|___|___|:|___|___| Hr : Min		Use 24 hr format

Module 7: Adult Women Module

Section 1: General Information

No	Variable	Question	Response Options	Code	Instructions/ Skip
101	AW_101	What's your name?(with nick name)	______________________		
102	AW_102	What's your date of birth	|___|___|/|___|___|/20|___|___| DD / MM / YYYY		
103	AW_103	Age of the respondent (Write complete year)	|___|___| year		
104	AW_104	What is your marital status?	1 = Never married<br>2 = Currently married<br>3 = Separated<br>4 = Divorced<br>5 = Widowed<br>88 = Refused		
105	AW_105	Completed years of education the respondent completed<br><br>***[Instruction:** Write down in total, the years the respondent*	|___|___| Years<br><br>• Primary/Ebtedayee=5<br>• Secondary/Dakhil=10		

No	Variable	Question	Response Options	Code	Instructions/ Skip
		spent in school for formal education (Is completed years) [Excluding pre-school and informal education). Write down "00" if no formal education.]	• Higher secondary/ Diploma/Alim=12<br>• Graduate/Fazil=16<br>• Post graduate/kamil/dawra=18		
106	AW_106	Occupation of the respondent over the past 12 months<br>If the participant has been employed more than one occupation in the last 12 months, consider the profession that he has spent most of his time as the main occupation and write it down	1 = Agricultural work(paddy)<br>2 = Agricultural work(without paddy)<br>3 = Agricultural day labor<br>4 = Un skild day labor<br>5 = Skiled day labor<br>6= Driver of Rickshaw/ van/Wheel barrow/Baby taxi/ Boatman<br>7=Fisherman<br>8=Employer<br>9=Profesionals<br>10=Businessman<br>11= Petty businessman<br>12= House maid<br>13=Zoom fermar<br>14= Do not earn<br>15=Poultry/dairy fermar<br>16=Handicrafts<br>17 =Spinach vegitable<br>18=Fishery<br>19 = Homemaker<br>20= Student<br>77 = Don,t know<br>66 =Age below 06 years		
107	AW_107	Completed years of education of the mother of the respondent<br><br>***[Instruction:** Write down in total, the years the respondent spent in school for formal education (Is completed years) [Excluding pre-school and informal education). Write down "00" if no formal education.]*	└─┴─┘ Years<br>• Primary/Ebtedayee=5<br>• Secondary/Dakhil=10<br>• Higher Secondary/Diploma/Alim=12<br>• Graduate/Fazil=16<br>• Post graduate/kamil/dawra=18		
108	AW_108	Mother's occupation of the respondent over the past 12 months<br>If the participant has been employed more than one occupation in the last 12 months, consider the profession that he has spent most of his time as the main occupation and write it down	1 = Agricultural work(paddy)<br>2 = Agricultural work(without paddy)<br>3 = Agricultural day labor<br>4 = Un skild day labor<br>5 = Skiled day labor<br>6= Driver of Rickshaw/ van/Wheel barrow/Baby taxi/ Boatman<br>7=Fisherman<br>8=Employer<br>9=Profesionals<br>10=Businessman<br>11= Petty businessman<br>12= House maid<br>13=Zoom fermar<br>14= Do not earn		

No	Variable	Question	Response Options	Code	Instructions/ Skip
			15=Poultry/dairy fermar<br>16=Handicrafts<br>17 =Spinach vegitable<br>18=Fishery<br>19 = Homemaker<br>20= Student<br>77 = Don,t know<br>66 =Age below 06 years		
109	AW_109	Completed years of education of the father of the respondent ***[Instruction:** Write down in total, the years the respondent spent in school for formal education (Is completed years) [Excluding pre-school and informal education). Write down "00" if no formal education.]*	└─┴─┘ Years<br>• Primary/Ebtedayee=5<br>• Secondary/Dakhil=10<br>• Higher/ Secondary/ Diploma/Alim=12<br>• Graduate/Fazil=16<br>• Post graduate/kamil/dawra=18		
110	AW_110	Father's occupation of the respondent over the past 12 months<br>If the participant has been employed more than one occupation in the last 12 months, consider the profession that he has spent most of his time as the main occupation and write it down	1 = Agricultural work(paddy)<br>2 = Agricultural work(without paddy)<br>3 = Agricultural day labor<br>4 = Un skild day labor<br>5 = Skiled day labor<br>6= Driver of Rickshaw/ van/Wheel barrow/Baby taxi/ Boatman<br>7=Fisherman<br>8=Employer<br>9=Profesionals<br>10=Businessman<br>11= Petty businessman<br>12= House maid<br>13=Zoom fermar<br>14= Do not earn<br>15=Poultry/dairy fermar<br>16=Handicrafts<br>17 =Spinach vegitable<br>18=Fishery<br>19 = Homemaker<br>20= Student<br>77 = Don,t know<br>66 =Age below 06 years		

Section 2: Dietary diversity

	Variable	Question	Yesterday during the day or night, did your child consume the following food items? (A)	During the last 07 days how many days did your child consume the following food items (B)	During the last 07 dayshow many times did your child consume the following food items (C)

	Variable	Question		Yesterday during the day or night, did your child consume the following food items? (A)	During the last 07 days how many days did your child consume the following food items (B)	During the last 07 dayshow many times did your child consume the following food items (C)
Now I request you to describe the food that you have eaten at home and out of home during the day or night(yesterday morning 6.00 am to today morning 6.00 am) yesterday. Please tell all types of food, drinks that you have eaten with morning, noon and dinner, or have breakfast / light breakfast. Tell us by think if you ate any food while preparing food.<br><br>Get started with what you ate yesterday morning.<br>-What did you eat in the morning wake up? Did you eat anything more**?**<br>-What did you eat more in the morning? Did you eat anything more**?**<br>-What did you eat at noon? Did you eat anything more**?**<br>-What did you eat at afternoon? Did you eat anything more**?**<br>-What did you eat at evening? Did you eat anything more**?**<br>-What did you eat at dinner? Did you eat anything more**?**<br>**Record first the answer of the questions of column A. collect the answer for column B/C after recording the answer of the questions of column A.**						
		Food Category	Examples of foods	A	B	C
201	AWD_201 A/B/C	Starchy Staples/ Food made from grains	Rice,flour bread, wheat, muri, maize,kichuri,barli,oot,kinoya,noodles,pasta	1 = Yes<br>2 = No	|____|____| days	|____|____| times
202	AWD_202 A/B/C	White roots and tubers and plantains	potatoes, sweet potatoes,sagu, erarut,cave,shalgam,kuchu,wiggle,ripe banana shaloo	1 = Yes<br>2 = No	|____|____| days	|____|____| times
203	AWD_203 A/B/C	Pulses	Dal/khicuri,peas,motor,soybean,tofu peanuts, humas	1 = Yes<br>2 = No	|____|____| days	|____|____| times
204	AWD_204 A/B/C	Legumes and Nuts	Peanut, Pistachios, kazu or any kond of peanut, ciya seeds, til, tisi,sunflower seeds,sweet pumpkin seeds	1 = Yes<br>2 = No	|____|____| days	|____|____| times
205	AWD_205 A/B/C	Dark green leafy vegetables	All kinds of leafy vegetables (poo,kuchu, kalmi) Brooklyn	1 = Yes<br>2 = No	|____|____| days	|____|____| times
206	AWD_206 A/B/C	Red/orange/yellow vegetables	Orange sweet potato,pumpkin, carrot or other yellow or orange vegetable	1 = Yes<br>2 = No	|____|____| days	|____|____| times
207	AWD_207 A/B/C	Red/orange/yellow fruits	Ripe mangoes, papaya, jackfruits other red/yellow or orange fruit	1 = Yes<br>2 = No	|____|____| days	|____|____| times
208	AWD_208 A/B/C	Vitamin C rich fruits	Guava, Strawberry, Lemon, Orange,grapes, Pineapple,raw mango,amalaki,kueue,tomatos	1 = Yes<br>2 = No	|____|____| days	|____|____| times

<table>
<tr><th></th><th>Variable</th><th colspan="2">Question</th><th>Yesterday during the day or night, did your child consume the following food items? (A)</th><th>During the last 07 days how many days did your child consume the following food items (B)</th><th>During the last 07 dayshow many times did your child consume the following food items (C)</th></tr>
<tr><td>209</td><td>AWD_209 A/B/C</td><td>Vitamin C rich vegetables</td><td>Raw tomato,chilli pepper, brussels sprouts,cauliflower,cabbage</td><td>1 = Yes
2 = No</td><td>|____|____ | days</td><td>|____|____ | times</td></tr>
<tr><td>210</td><td>AWD_210 A/B/C</td><td>Other vegetables or fruits</td><td>peas, asparagas, bit,koci bash, cauliflower,selari,shawsha,brinjal,letus, masrum,mula,jukini</td><td>1 = Yes
2 = No</td><td>|____|____ | days</td><td>|____|____ | times</td></tr>
<tr><td>211</td><td>AWD_211 A/B/C</td><td>Other fruits</td><td>Apple, avakado,bam, cherry,ripe jackfruit</td><td>1 = Yes
2 = No</td><td>|____|____ | days</td><td>|____|____ | times</td></tr>
<tr><td>212</td><td>AWD_212 A/B/C</td><td>Eggs</td><td>Hen/duck, other birds,</td><td>1 = Yes
2 = No</td><td>|____|____ | days</td><td>|____|____ | times</td></tr>
<tr><td>213</td><td>AWD_213 A/B/C</td><td>Organ meat</td><td>Cheeks, liver, Stomach, Ridpind, Kidney</td><td>1 = Yes
2 = No</td><td>|____|____ | days</td><td>|____|____ | times</td></tr>
<tr><td>214</td><td>AWD_214 A/B/C</td><td>Flesh</td><td>Beef, Pork, Veal, Lamb, Goat, Chicken, Duck or flesh of any other animal</td><td>1 = Yes
2 = No</td><td>|____|____ | days</td><td>|____|____ | times</td></tr>
<tr><td>215</td><td>AWD_215 A/B/C</td><td>Small Fish</td><td>Small Fish Eaten Whole with Bones (i.e.Kachki, mola, dhela, chapila, batashi, small prawn, dried fish)</td><td>1 = Yes
2 = No</td><td>|____|____ | days</td><td>|____|____ | times</td></tr>
<tr><td>216</td><td>AWD_216 A/B/C</td><td>Large fish/sea food</td><td>Large Whole Fish and Shell Fish and dried fish</td><td>1 = Yes
2 = No</td><td>|____|____ | days</td><td>|____|____ | times</td></tr>
<tr><td>217</td><td>AWD_217 A/B/C</td><td>Dairy</td><td>Milk, cheese, yogurt or other milk products</td><td>1 = Yes
2 = No</td><td>|____|____ | days</td><td>|____|____ | times</td></tr>
<tr><td>218</td><td>AWD_218</td><td>Insects and other protein foods</td><td>Fish egg, insect, snail</td><td>1 = Yes
2 = No</td><td>|____|____ | days</td><td>|____|____ | times</td></tr>
<tr><td>219</td><td>AWD_219 A/B/C</td><td>Edible Oil</td><td>Ghee, butter,cream, sour, fat, margarine, mayonnaise, palm oil, vegetable oil,</td><td>1 = Yes
2 = No</td><td>|____|____ | days</td><td>|____|____ | times</td></tr>
<tr><td>220</td><td>AWD_220 A/B/C</td><td>Savory and fried snacks</td><td>Crisps and chips, fried dough or other fried snacks singaru, samarcha</td><td>1 = Yes
2 = No</td><td>|____|____ | days</td><td>|____|____ | times</td></tr>
<tr><td>221</td><td>AWD_221</td><td>Sweets</td><td>Sugary foods, such as chocolates, candies, cookies/sweet biscuits and</td><td>1 = Yes
2 = No</td><td>|____|____ | days</td><td>|____|____ | times</td></tr>
</table>

	Variable	Question		Yesterday during the day or night, did your child consume the following food items? (A)	During the last 07 days how many days did your child consume the following food items (B)	During the last 07 dayshow many times did your child consume the following food items (C)
	A/B/C		cakes, sweet pastries or ice cream, any kind of sweets ,honey ,halua , condensed milk,tiler khaza			
222	AWD_222 A/B/C	Miscellaneous (Sweet drinks)	Tea with suger, soft drinks, juice, energy drinks,yogurt drinks,chocolate drinks, horlicks,moltova	1 = Yes<br>2 = No	|____|____| days	|____|____| times
223	AWD_223 A/B/C	Other beverages and foods	Tea or coffee if not sweetened, clear broth, alcohol, Pickles, olives and similar	1 = Yes<br>2 = No	|____|____| days	|____|____| times
224	AWD_224 A/B/C	Condiments/spices	Spices, coriander leaves, sausage, garlic, ketchup, lemon juice, mint leaves, drinks, betel leaves, tobacco leaves, jars	1 = Yes<br>2 = No	|____|____| days	|____|____| times

No	Variable	Question	Response Options	Code	Instructions/ Skip
The next questions I will ask about the **fruits** and **vegetables** that you usually eat; I have a nutrition card/picture here that shows you some examples of local fruits and vegetables; Each picture represents the size of a serving; To answer these questions, please think of a typical week.(If 1-2 times/month then record it as "00.")					
225	AWD_225	In a typical week, on how many days do you **eat fruit**?<br><br>***Instruction:*** *Demonstrate fruit's show card and ask the participant to think about it. Do not consider packaged fruit juice but consider fruit juice prepared at home using fresh fruits. A typical week refers to "A usual week except religious event or any other festival. If 1-2 times/month then record it as "00."* [USE SHOWCARD]	Number of days<br>|____|____| 77 = Don't Know<br>[If '00' days, go to ABD_227]		
226	AWD_226	How many **servings** of fruit do you eat on **one** of those days? (USE SHOWCARD)<br><br>***Instruction:*** *Ask the participant to think about any one of those days of a typical week. Use supplied bowl and show card to measure servings.*	Number of servings<br>|___|___|.|___| 77.7 = Don't Know		
227	AWD_227	In a typical week, on how many days do you **eat vegetables**?<br><br>***Instruction:*** *Demonstrate vegetable's show card and ask the participant to think about it. Do not consider potato as vegetables. Demonstrate serving with the*	Number of days<br>|____|____| 77 = Don't Know<br>[If '0' days, go to ABD_229]		

	Variable	Question	Yesterday during the day or night, did your child consume the following food items? (A)	During the last 07 days how many days did your child consume the following food items (B)	During the last 07 dayshow many times did your child consume the following food items (C)
		help of supplied cup and show card as cooked and uncooked vegetables. Consider only vegetables serving in case of cooked vegetables with fish. A typical week refers to "A usual week except religious event or any other festival. If 1-2 times/month then record it as "00." [USE SHOWCARD]			
228	AWD_228	How many **servings** of vegetables do you eat on one of those days?<br><br>***Instruction:*** *Ask the participant to think about any one of those days of a typical week. Use supplied cup and show card to measure serving.*[USE SHOWCARD]	Number of servings<br>|___|___|.|___| 77.7 = Don't Know		
229	AWD_229	Do you currently eat any tablet, capsul,syrup contaning vitamin/minarels	1=Yes 2=No		If "No" go to next section
230	AWD_230	Write the name of medicine by observed the prescription and medication	A.___________<br>B.___________<br>C.___________		

Section 3: Behavioral risk factor (Smoking/smokeless tobacco physical activity)

No	Variable	Question	Response Options	Code	Instructions/ Skip
**Tobacco Use**<br>Now I am going to ask you some questions about tobacco (Smoke tobacco and smokeless tobacco) use.					
301	AWR_301	Do you currently smoke any tobacco products, such as cigarettes, *bidis, hookah, cigars* or pipes?<br><br>*Instruction: Demonstrate show card to the participant and ask which smoke tobacco products/he use.* [USE SHOWCARD]	1 = Yes; 2= No<br>If No, go to ABR_304		
302	AWR_302	Do you currently smoke tobacco products daily?<br><br>*Instruction: This question is applicable for those respondents who smoke tobacco product daily.*<br>*Daily meanus" tobacco smoking is at least one time/day for almost one month or more than that*<br>*If that is the case respondant started*	1 = Yes; 2= No		

No	Variable	Question	Response Options	Code	Instructions/ Skip
		somoking from 25 days and still running, that is regarded as everyday.			
303	AWR_303	How old were you when you **first started** smoking?	Age └─┴─┘ years<br>77 = Don't know		
304	AWR_304	Do you currently use anysmokeless tobacco products such as Betel quid with zarda, zarda only or zarda with supari, Betel quid with sadapata, pan masala withtobacco, sadapata chewing, gul, Khoinee, Nossi, gutka?<br>***Instruction:*** *Ask respondent to think and answer whether h/she use any smokeless tobacco products such as Betel quid with zarda, zarda only or zarda with supari, Betel quid with sadapata, pan masala with tobacco, sadapata chewing, gul, Khoinee, Nossi, gutka. Here, only betel quid, supari and lime will not be considered. It will be considered as smokeless tobacco if respondent use Betel quid with zarda, zarda only or zarda with supari, Betel quid with sadapata, pan masala with tobacco, sadapata chewing, gul, Khoinee, Nossi, gutka.* [USE SHOWCARD]	1 = Yes; 2= No<br><br>If No, go to ABR_307		
305	AWR_305	Do you currently use smokeless tobacco daily?<br>***Instruction:*** *Daily means "using smokeless tobacco is at least one time/day for almost one month or more than that.*<br>*If that is the case respondant started somoking from 25 days and still running, that is regarded as everyday.*	1 = Yes; 2= No		
306	AWR_306	How old were you when you started smokeless tobacco?	Age └─┴─┘ years<br>77 = Don't know		
307	AWR_307	Which of your parents or guardians use any form of tobacco?	1= Neither<br>2= My father or male guardian<br>3= My mother or female guardian<br>4= Both<br>5= I do not know<br>99= Others (Specify)_______		
		**Physical activity**			

<table>
<tr><td colspan="6">Next I am going to ask you about the time you spend doing different types of physical activity in a typical week. Please answer these questions even if you do not consider yourself to be a physically active person.
Think first about the time you spend doing work. Think of work as the things that you have to do such as paid or unpaid work, study/training, household chores, harvesting food/crops, fishing or hunting for food, seeking employment. [Insert other examples if needed]. In answering the following questions 'vigorous-intensity activities' are activities that require hard physical effort and cause large increases in breathing or heart rate, 'moderate-intensity activities' are activities that require moderate physical effort and cause small increases in breathing or heart rate.
Instruction: Please read out the above statement for respondent. Do not drop this part. At first respondent will must think about his/her daily activities (Paid or unpaid work, study/training, household chores, harvesting food/crops, fishing or hunting for food, seeking employment). Then will think time for travelling from one place to another and at last time spent during leisure period.
Do not forget to demonstrate show card to the respondent which will help them to respond.
It is needed to let participant remember when answer the following things
Vigorous intensity activity: Such kind of physical activity that causes <u>large increases</u> in breathing or heart rate.
Moderate-intensity activity: Such kind of physical activity that causes <u>small increases</u> in breathing or heart rate.</td></tr>
<tr><td colspan="6">Now I would like to know about the 'Vigorous-intensity activities' you perform</td></tr>
<tr><td colspan="6">'Vigorous-intensity activities' performed as a part of daily work / professional work/ outside daily work / professional work.</td></tr>
<tr><td>308</td><td>AWR_308</td><td>Does your daily work / professional work, sports, fitness or recreational(leisure) activities involve <u>vigorous-intensity activity</u> that causes large increases in breathing or heart rate like [carrying or lifting heavy loads, digging or construction work, harvesting paddy, fishing using net, running, football, Kabaddi, Dariabandha, Gollachut etc.] <u>for at least 10 minutes continuously?</u>
<u>Instruction: Ask participant to think about those vigorous-intensity activities performed as a part of professional work/ recreational activities. Those activities will be considered as vigorous-intensity activities that causes large increase in breathing or heart rate.</u> [USE SHOWCARD]</td><td>1 = Yes 2 = No
[If 'No', please go to 311]</td><td></td><td></td></tr>
<tr><td>309</td><td>AWR_309</td><td>In a typical week, on how many days do you do <u>vigorous- intensity activities/sports/fitness</u> as part of <u>daily work / professional work/ recreational activities</u>?
Instruction: In a typical week refers to "activities performed by respondent in a typical week". Valid response range is 1-7 days.</td><td>|__|__| Day
77 = Don't know
[If 'Don't know', please go to ABR_311]</td><td></td><td></td></tr>
<tr><td>310</td><td>AWR_310</td><td><u>How much time</u> do you spend doing <u>vigorous-intensity activities/sports/fitness</u> at <u>daily work / professional work/recreational activities</u> on a typical day?
Instruction: Ask respondent to think about a single day (which can be remembered easily) s/he performed vigorous-intensity activities as part of professional work/ recreational activities. Respondent will consider only those activities that are performed for at least 10 minutes or more continuously. Check unusual (more than 04 hours) values.</td><td>________
Minutes</td><td></td><td></td></tr>
<tr><td colspan="6">Now I would like to know about the 'Moderate-intensity activity' you perform</td></tr>
<tr><td colspan="6">'Moderate-intensity activity' performed as a part of daily work / professional work</td></tr>
</table>

311	AWR_311	Does your daily work / professional work/ spots/fitness/ recreational (leisure) work involve moderate-intensity activity that causes small increases in breathing or heart rate such as brisk walking [or carrying light loads, cycling, swimming, volleyball] for at least 10 minutes continuously?<br>***Instruction:*** *Ask participant to think about those moderate-intensity activities performed as a part of professional work/ recreational activities. Those activities will be considered as moderate-intensity activities that causes small increase in breathing or heart rate.* [USESHOWCARD]	1 = Yes 2 = No<br>[If 'No', please go to ABR_314]		
312	AWR_312	In a typical week, on how many days do you do moderate- intensity activities as part of your daily work / professional work/sports/fitness or recreational work?<br>***Instruction:*** *In a typical week refers to "activities done by respondent in a typical week". Valid response range is 1-7 days.*	└─┴─┘ Day<br>77 = Don't know<br>[If 'Don't know', please go to ABR_314]		
313	AWR_313	How much time do you spend doing moderate-intensity activities at daily work / professional work/ sports/fitness or recreational work on a typical day?<br>***Instruction:*** *Ask respondent to think about a single day (which can be remembered easily) s/he performed moderate-intensity activities as part of professional work/recreational activities. Respondent will consider only those activities that are performed for at least 10 minutes or more continuously. Check unusual (more than 04 hours) values.*	________<br>Minutes		
**Sedentary behavior** The following question is about sitting or reclining at work, at home, getting to and from places, or with friends including time spent sitting at a desk, sitting with friends, traveling in car, bus, train, reading, playing cards or watching television, but do not include time spent sleeping.					
314	AWR_314	How much time do you usually spend sitting or reclining on a typical day?<br><br>***Instruction:*** *Ask respondent to think about time spent on sitting or reclining at work, office, study, watching television, computer use, at kitchen, during rest.Do not include time spent during sleeping.*	________<br>Minutes		
315	AWR_315	How much time do you watch television daily?	________<br>Minutes		

Section 4: Chronic Disease

No	Variable	Question	Response Options	Code	Instructions/ Skip
401	WCD_401	Has a health care provider ever told you that you have high blood pressure also called hypertension (other than during pregnancy)?	1 = Yes<br>2 = No		
402	WCD_402	Has a health care provider ever told you that you have heart disease?	1 = Yes<br>2 = No		
403	WCD_403	Has a health care provider ever told you that you have asthma?	1 = Yes<br>2 = No		
404	WCD_404	Has a health care provider ever told you that you have Kidney Disease?	1 = Yes<br>2 = No		

No	Variable	Question	Response Options	Code	Instructions/ Skip
405	WCD_405	Has a health care provider ever told you that you have Diabetes?	1 = Yes<br>2 = No		
406	WCD_406	Has a health care provider ever told you that you have stroke?	1 = Yes<br>2 = No		
407	WCD_407	Has a health care provider ever told you that you have cancer?	1 = Yes<br>2 = No		
408	WCD_408	Has a health care provider ever told you that you have mental problem?	1 = Yes<br>2 = No		

Section 5: Reproductive history, menstruation/Menopause, menstrual hygiene

I would like to ask some questions regarding your reproductive history, menstruation, menopause and menopausal hygiene. This information is collecting for the surveillance purpose and I am ensuring you the confidentiality of your information. Can I ask?

No	Variable	Question	Response Options	Code	Instructions/ Skip
501	AWM_501	When your menarche started?	|____|____|Years		Write "00" if not started yet
502	AWM_502	What material do you use during period/menstruation?	1= Sanitary pad<br>2= Old cloth<br>3= New Cloth<br>99= Others (Specify)___		
503	AWM_503	How many times you became pregnant in your life?<br>(Including abortion, MR, still birth and live birth)?	|____|____|Number		If"0" go to 505
504	AWM_504	How many times you delivered live birth in your life	|____|____|Number		
505	AWM_505	Are you currently in menstruation?	1= Yes 2=No		If "Yes" go to 507
506	AWM_506	What is the reason for your not being menstruation?	1 = I am pregnant<br>2 = I may become pregnant<br>3 = menopause<br>99= others (specify below)_____		
507	AWM_507	What is your Last date of menustration (LMP)	|____|____|/|____|____|/20|____|____| DD / MM / YY		
508	AWM_508	Year of menopause	|____|____|Years		

Section 6 : Health Care

No	Variable	Question	Response Options	Code	Instructions
601	AWH_601	Did You deliver any live birth or stillbirth in the last 24 months ?	1=Yes 2=No		If "No" go to section 6
602	AWH_602	Date of delivery of live birth or still birth.	|___|___|/|___|___|/20|___|___| Day/Month/Year		
603	AWH_603	Does your child is living now ?	1=Yes 2=No<br>88=Not applicable		
604	AWH_604	Have you been registered as a pregnant	1=Yes 2=No		

No	Variable	Question	Response Options	Code	Instructions
		woman when you were pregnant?			
605	AWH_605	How many times you took ante natal care (ANC) when you were pregnant?	|____|____|Times		
606	AWH_606	From whom you usually took the ANC service when you were pregnant?<br><br>(may be multiple answer)	1 =Passed doctor<br>2 =Nurses/Midwives/Paramedics<br>3 = FWV<br>4 = Medical assistant/Sub assistant<br>5 = NGO health worker<br>6 = Skilled midwife<br>7 = Trained conventional midwife<br>8 = Conventional midwife<br>77 =Don't know<br>99 = Others (specify bellow)_____		
607	AWH_607	Have you been measured your weight when you were pregnant?	1=Yes 2=No		
608	AWH_608	Have you eaten Iron and Folic acid tablet when you were pregnant?	1=Yes 2=No		If" No" go to 512
609	AWH_609	From where you got Iron and Folic acid tablet when you were pregnant?<br><br>(may be multiple answer)	**GOVERNMENT PROVIDERS**<br>1= Govt. Field worker<br>2= Govt. satellite clinic/ EPI Outreach clinic<br>3= Govt. Community clinic<br>4=Govt. Health & Family Welfare Centre<br>5=Govt. Upazila Health Complex<br>6= Govt. Maternal & Child Welfare Centre Government<br>7=Other government Hospital<br>**NGO PROVIDERS**<br>8= NGO health clinic and hospital<br>9=NGO Field Worker<br>10= Community Nutrition Promoter (CNP)<br>**PRIVATE PROVIDERS**<br>11 = Private doctor/clinic/hospital<br>12 = Pharmacy<br>13 = Traditional healer<br>14 = Homeopath<br>17=Don't know<br>99 = Others (Specify)___________		
610	AWH_610	How many months did you consume Iron and Folic acid tablet when you were pregnant?	|____|____|Months		
611	AWH_611	How many tablet of Iron and Folic acid did you consume in a week when you were pregnant?	|____|____|Tablet		
612	AWH_612	Did you consume calciam tablet whwen you were pregnant?	1=Yes 2=No		If" No" go to 516
613	AWH_513	From where you got Calciam tablet when you were pregnant?<br><br>(may be multiple answer)	**GOVERNMENT PROVIDERS**<br>1= Govt. Field worker<br>2= Govt. satellite clinic/ EPI Outreach clinic<br>3= Govt. Community clinic<br>4=Govt. Health & Family Welfare Centre<br>5=Govt. Upazila Health Complex<br>6= Govt. Maternal &<br>**NGO PROVIDERS**<br>8= NGO health clinic and hospital<br>9=NGO Field Worker<br>10= Community Nutrition Promoter (CNP)<br>**PRIVATE PROVIDERS**<br>11 = Private doctor/clinic/hospital<br>12 = Pharmacy<br>13 = Traditional healer<br>14 = Homeopath<br>17=Don't know<br>99 = Others		

No	Variable	Question	Response Options	Code	Instructions
			Child Welfare Centre Government<br>7=Other government Hospital (Specify)__________		
614	AWH_614	How many months did you consume Calciam tablet when you were pregnant?	|____|____|Months		
615	AWH_615	How many tablet of Calciam did you consume in a week when you were pregnant?	|____|____|Tablet		
616	AWH_616	Have any doctor or any other health care provider gave you nutrition related counseling when you were pregant?	1=Yes 2=No		
617	AWH_617	Advice on what matters ?	1 =Take enough food<br>2 =Enough rest<br>3 =Category of nutritional food<br>4 =Rules of iron and Folic acid tabet intake<br>5 =Rules of Calciam tablet intake		
If stillbirth, need not to ask following questions.					
618	AWH_618	Had your child been measured birth weight within 3 days after birth?	1=Yes 2=No<br>88=Not applicable		If not applicable go to next section
619	AWH_619	What was your child's birth weight?	|____|.|____|____|____| kg		
620	AWH_620	Afterwards had any doctor or any health care provider took your child's weight?	1=Yes 2=No<br>88=Not applicable		
621	AWH_621	Do you have any charts for your child's weight for age?	1=Yes 2=No		
622	AWH_622	After your baby is born, Have any doctor or any other health care provider gave you nutrition related counseling?	1=Yes 2=No		
623	AWH_623	Advice on what matters ?	1 =Take enough food<br>2 =Enough rest<br>3 =Category of nutritional food<br>4 =Rules of iron and Folic acid tabet intake<br>5 =Rules of Calciam tablet intake<br>6=Exclusive breastfeeding at first 6 months<br>7=Continue breastfeeding up to 2 years age<br>8=Rules of supplementary food intake<br>9=The rules for feeding the child at the time of illness<br>10=Do not feed the store food		

Section 7: Blood Pressure

No.	Variable	Question	Responses and code categories	Instructions/Skip
701	WBP_701	Start Time of measuring blood pressure	|____|____| : |____|____| Hr : Min	Use 24 hr format

Measurement -1 (Take the first measurement considering at least 15 minutes rest)

No.		Measurement Name	Systolic	Diastolic	Time of Measurement
702	WBP_702 A/B/C	Blood Pressure (mm of Hg)	A.|___|___|___|	B.|___|___|___|	C.|____|____|:|____|____| Hr : Min
**Measurement - 2** (Allow 3 minutes' interval between two measurements)					
			Systolic	Diastolic	Time of Measurement
703	WBP_703 A/B/C	Blood Pressure (mm of Hg)	A.|___|___|___|	B.|___|___|___|	C.|____|____|:|____|____| Hr : Min

<table>
<tr><td colspan="6">Measurement - 3 (If difference of between 1st and 2nd measurement is more than 10 mm of Hg, then take 3rd measurement for Blood Pressure)</td></tr>
<tr><td rowspan="2">704</td><td rowspan="2">WBP_704 A/B/C</td><td rowspan="2">Blood Pressure (mm of Hg)</td><td>Systolic</td><td>Diastolic</td><td>Time of Measurement</td></tr>
<tr><td>A.|___|___|___|</td><td>B.|___|___|___|</td><td>C.|____|____|:|____|____|
Hr : Min</td></tr>
<tr><td>705</td><td>WBP_705</td><td>Blood pressure measuring machine ID</td><td colspan="2">|____|____|</td><td></td></tr>
<tr><td>706</td><td>WBP_706</td><td>Measurer's Name and Code</td><td colspan="2">|____|____|____|</td><td>Write your full name and code</td></tr>
<tr><td>707</td><td>WBP_707</td><td>End Time of measuring blood pressure</td><td colspan="2">|____|____| : |____|____|
Hr : Min</td><td>Use 24 hr format</td></tr>
<tr><td colspan="6">[During any measurement, If Systolic BP ≥ 140 mm of Hg, Diastolic BP ≥ 90 mm of Hg - refer the respondent to appropriate health center]</td></tr>
</table>

Referral:

No.	Variable	Question	Responses and code categories	Code	Instructions/Skip
708	WBP_708	Referral needed?	1 = Yes 2 = No		If 2, go to section 8
709	WBP_709	Where referred?	1 = Upazila Health Complex<br>2 = District Hospital<br>3= Medical College Hospital<br>4= Doctor's private chamber<br>6 = Others (Specify)____________		

Section 8: Anthropometry

No	Variable	Question	Responses and code categories	Code	Instructions
801	AWA_801	Start Time of anthropometry	|____|____| : |____|____| Hr : Min		Use 24 hr format
802	AWA_802	Weight Scale ID	|____|____|		
803	AWA_803	Height Scale ID	|____|____|		
804	AWA_804	Clothing type during weight measurement	1 = Light clothing<br>2 = Slightly heavy clothing<br>3 = Heavy clothing		
805	AWA_805	Name of measurer			
806	AWA_806	Code of measurer	|____|____|		

No	Variable	Measurement	Measurement 1	Measurement 2	Measurement 3
807	AWA_807A /B/C	Height (Cm)	A.|____|____|____|.|____|	B.|____|____|____|.|____|	C.|____|____|____|.|____| If A and B differs more than 0.5 cm, take 3rd measurement
808	AWA_808A /B/C	Weight (kg)	A.|____|____|____|.|____|	B.|____|____|____|.|____|	C.|____|____|____|.|____| If A and B differs more than 0.1kg, take 3rd measurement
809	AWA_809A /B/C	Fat%	A.|____|____|____|.|____|	B.|____|____|____|.|____|	
810	AWA_810A /B/C	Water%	A.|____|____|____|.|____|	B.|____|____|____|.|____|	

No	Variable	Question	Responses and code categories		Code / Instructions
811	AWA_811A /B/C	Waist circumference (Cm)	A.|____|____|.|____|	B.|____|____|.|____|	C.|____|____|.|____| If A and B differs more than 0.5 cm, take 3rd measurement
812	AWA_812	Comment			
813	AWA_813	End Time of measurement	|____|____|:|____|____| Hr : Min		Use 24 hr format

Module 8: Elderly Men/Women Module

Section 1: General Information

No	Variable	Question	Response Options	Code	Instructions/ Skip
101	EM_101	Name of the respondent(with nick name	______________________		
102	EM_102	Date of birth of the respondent	|____|____|/|____|____|/20|____|____| DD / MM / YYYY		
103	EM_103	Age in completed years	|____|____| Years		
104	EM_104	Sex of respondent	1 = Male 2 = Female		
105	EM_105	What is your marital status?	1 = Never married<br>2 = Currently married<br>3 = Separated; 4 = Divorced<br>5 = Widowed; 88 = Refused		
106	EM_106	Completed years of education the respondent completed *[**Instruction:** Write down in total, the years the respondent spent in school for formal education (Is completed years) [Excluding pre-school and informal education). Write down "00" if no formal education.]*	|____|____| Years<br>• Primary/Ebtedayee=5<br>• Secondary/Dakhil=10<br>• Higher secondary/Diploma/Alim=12<br>• Graduate/Fazil=16<br>• Post graduate/kamil/dawra=18		
107	EM_107	Occupation of the respondent over the past 12 months	1 = Agricultural work(paddy)<br>2 = Agricultural work(without paddy)<br>3 = Agricultural day labor<br>4 = Un skild day labor<br>5 = Skiled day labor<br>6= Driver of Rickshaw/ van/Wheel barrow/Baby taxi/ Boatman<br>7=Fisherman<br>8=Employer<br>9=Profesionals<br>10=Businessman<br>11= Petty businessman<br>12= House maid<br>13=Zoom fermar<br>14= Do not earn<br>15=Poultry/dairy fermar<br>16=Handicrafts<br>17 =Spinach vegitable<br>18=Fishery		JICA Q: C8

No	Variable	Question	Response Options	Code	Instructions/ Skip
			19 = Homemaker<br>20= Student<br>77 = Don,t know<br>66 =Age below 06 years		
108	EM_108	Do you get pension from government or non government institution?	1 =Yes 2 =No		
109	EM_109	Do you get any kind of adult allowance?	1 =Yes 2 =No		
110	EM_110	What is your monthly income from all sources?			if no income write "00"

Section 2: Dietary diversity

	Variable	Question		Yesterday during the day or night, did your child consume the following food items? (A)	During the last 07 days how many days did your child consume the following food items (B)	During the last 07 dayshow many times did your child consume the following food items (C)
Now I requst you to describe the food that you have eaten at home and out of home during the day or night(yesterday morning 6.00 am to today morning 6.00 am) yesterday. Please tell all types of food, drinks that you have eaten with morning, noon and dinner, or have breakfast / light breakfast. Tell us by think if you ate any food while preparing food.<br><br>Get started with what you ate yesterday morning.<br>-What did you eat in the morning wake up? Did you eat anything more?<br>-What did you eat more in the morning? Did you eat anything more?<br>-What did you eat at noon? Did you eat anything more?<br>-What did you eat at afternoon? Did you eat anything more?<br>-What did you eat at evening? Did you eat anything more?<br>-What did you eat at dinner? Did you eat anything more?<br>**Record first the answer of the questions of column A. collect the answer for column B/C after recording the answer of the questions of column A.**						
		Food Category	Examples of foods	A	B	C
201	EDD_201 A/B/C	Starchy Staples/ Food made from grains	Rice,flour bread, wheat, muri, maize,kichuri,barli,oot,kinoya,noodles,pasta	1 = Yes<br>2 = No	|___|___| days	|___|___| times
202	EDD_202 A/B/C	White roots and tubers and plantains	potatoes, sweet potatoes,sagu, erarut,cave,shalgam,kuchu,wiggle,ripe banana shaloo	1 = Yes<br>2 = No	|___|___| days	|___|___| times
203	EDD_203 A/B/C	Pulses	Dal/khicuri,peas,motor,soybean,tofu peanuts, humas	1 = Yes<br>2 = No	|___|___| days	|___|___| times

	Variable	Question		Yesterday during the day or night, did your child consume the following food items? (A)	During the last 07 days how many days did your child consume the following food items (B)	During the last 07 dayshow many times did your child consume the following food items (C)
204	EDD_204 A/B/C	Legumes and Nuts	Peanut, Pistachios, kazu or any kond of peanut, ciya seeds, til, tisi,sunflower seeds,sweet pumpkin seeds	1 = Yes 2 = No	|____|____| days	|____|____| times
205	EDD_205 A/B/C	Dark green leafy vegetables	All kinds of leafy vegetables (poo,kuchu, kalmi) Brooklyn	1 = Yes 2 = No	|____|____| days	|____|____| times
206	EDD_206 A/B/C	Red/orange/yellow vegetables	Orange sweet potato,pumpkin, carrot or other yellow or orange vegetable	1 = Yes 2 = No	|____|____| days	|____|____| times
207	EDD_207 A/B/C	Red/orange/yellow fruits	Ripe mangoes, papaya, jackfruits other red/yellow or orange fruit	1 = Yes 2 = No	|____|____| days	|____|____| times
208	EDD_208 A/B/C	Vitamin C rich fruits	Guava, Strawberry, Lemon, Orange,grapes, Pineapple,raw mango,amalaki,kueue,tomatos	1 = Yes 2 = No	|____|____| days	|____|____| times
209	EDD_209 A/B/C	Vitamin C rich vegetables	Raw tomato,chilli pepper, brussels sprouts,cauliflower,cabbage	1 = Yes 2 = No	|____|____| days	|____|____| times
210	EDD_210 A/B/C	Other vegetables or fruits	peas, asparagas, bit,koci bash, cauliflower,selari,shawsha,brinjal,letus, masrum,mula,jukini	1 = Yes 2 = No	|____|____| days	|____|____| times
211	EDD_211 A/B/C	Other fruits	Apple, avakado,bam, cherry,ripe jackfruit	1 = Yes 2 = No	|____|____| days	|____|____| times
212	EDD_212 A/B/C	Eggs	Hen/duck, other birds,	1 = Yes 2 = No	|____|____| days	|____|____| times
213	EDD_213 A/B/C	Organ meat	Cheeks, liver, Stomach, Ridpind, Kidney	1 = Yes 2 = No	|____|____| days	|____|____| times
214	EDD_214 A/B/C	Flesh	Beef, Pork, Veal, Lamb, Goat, Chicken, Duck or flesh of any other animal	1 = Yes 2 = No	|____|____| days	|____|____| times
215	EDD_215 A/B/C	Small Fish	Small Fish Eaten Whole with Bones (i.e.Kachki, mola, dhela, chapila, batashi, small prawn, dried fish)	1 = Yes 2 = No	|____|____| days	|____|____| times
216	EDD_216 A/B/C	Large fish/sea food	Large Whole Fish and Shell Fish and dried fish	1 = Yes 2 = No	|____|____| days	|____|____| times
217	EDD_217 A/B/C	Dairy	Milk, cheese, yogurt or other milk products	1 = Yes 2 = No	|____|____| days	|____|____| times

	Variable	Question		Yesterday during the day or night, did your child consume the following food items? (A)	During the last 07 days how many days did your child consume the following food items (B)	During the last 07 dayshow many times did your child consume the following food items (C)
218	EDD_218	Insects and other protein foods	Fish egg, insect, snail	1 = Yes<br>2 = No	|____|____| days	|____|____| times
219	EDD_219 A/B/C	Edible Oil	Ghee, butter,cream, sour, fat, margarine, mayonnaise, palm oil, vegetable oil,	1 = Yes<br>2 = No	|____|____| days	|____|____| times
220	EDD_220 A/B/C	Savory and fried snacks	Crisps and chips, fried dough or other fried snacks singaru, samarcha	1 = Yes<br>2 = No	|____|____| days	|____|____| times
221	EDD_221 A/B/C	Sweets	Sugary foods, such as chocolates, candies, cookies/sweet biscuits and cakes, sweet pastries or ice cream, any kind of sweets ,honey ,halua , condensed milk,tiler khaza	1 = Yes<br>2 = No	|____|____| days	|____|____| times
222	EDD_222 A/B/C	Miscellaneous (Sweet drinks)	Tea with suger, soft drinks, juice, energy drinks,yogurt drinks,chocolate drinks, horlicks,moltova	1 = Yes<br>2 = No	|____|____| days	|____|____| times
223	EDD_223 A/B/C	Other beverages and foods	Tea or coffee if not sweetened, clear broth, alcohol, Pickles, olives and similar	1 = Yes<br>2 = No	|____|____| days	|____|____| times
224	EDD_224 A/B/C	Condiments/spices	Spices, coriander leaves, sausage, garlic, ketchup, lemon juice, mint leaves, drinks, betel leaves, tobacco leaves, jars	1 = Yes<br>2 = No	|____|____| days	|____|____| times

No	Variable	Question	Response Options	Code	Instructions/ Skip
The next questions I will ask about the **fruits** and **vegetables** that you usually eat; I have a nutrition card/picture here that shows you some examples of local fruits and vegetables; Each picture represents the size of a serving; To answer these questions, please think of a typical week.(If 1-2 times/month then record it as "00.")					
225	EDD_225	In a typical week, on how many days do you **eat fruit**?<br><br>***Instruction:*** *Demonstrate fruit's show card and ask the participant to think about it. Do not consider packaged fruit juice but consider fruit juice prepared at home using fresh fruits. A typical week refers to "A usual week except religious event or any other festival. If 1-2 times/month then record it as "00."* [USE SHOWCARD]	Number of days<br>|____|____| 77 = Don't Know<br>[If '00' days, go to ABD_227]		

	Variable	Question	Yesterday during the day or night, did your child consume the following food items? (A)	During the last 07 days how many days did your child consume the following food items (B)	During the last 07 dayshow many times did your child consume the following food items (C)
226	EDD_226	How many **servings** of fruit do you eat on **one** of those days? (USE SHOWCARD)<br><br>***Instruction:*** *Ask the participant to think about any one of those days of a typical week. Use supplied bowl and show card to measure servings.*	Number of servings<br>|___|___|.|___| 77.7 = Don't Know		
227	EDD_227	In a typical week, on how many days do you **eat vegetables**?<br><br>***Instruction:****Demonstrate vegetable's show card and ask the participant to think about it. Do not consider potato as vegetables. Demonstrate serving with the help of supplied cup and show card as cooked and uncooked vegetables. Consider only vegetables serving in case of cooked vegetables with fish. A typical week refers to "A usual week except religious event or any other festival. If 1-2 times/month then record it as "00."* [USE SHOWCARD]	Number of days<br>|____|____| 77 = Don't Know<br>[If '0' days, go to ABD_229]		
228	EDD_228	How many **servings** of vegetables do you eat on one of those days?<br><br>***Instruction:*** *Ask the participant to think about any one of those days of a typical week. Use supplied cup and show card to measure serving.*[USE SHOWCARD]	Number of servings<br>|___|___|.|___| 77.7 = Don't Know		
229	EDD_229	Do you currently eat any tablet, capsul,syrup contaning vitamin/minarels	1=Yes 2=No		If "No" go to next section
230	EDD_230	Write the name of medicine by observed the prescription and medication	A.___________<br>B.___________<br>C.___________		

Section 3: Behavioral risk factor (Smoking/smokeless tobacco physical activity)

No	Variable	Question	Response Options	Code	Instructions/ Skip
**Tobacco Use**<br>Now I am going to ask you some questions about tobacco (Smoke tobacco and smokeless tobacco) use.					

No	Variable	Question	Response Options	Code	Instructions/ Skip
301	ER_301	Do you currently smoke any tobacco products, such as cigarettes, *bidis, hookah, cigars* or pipes?<br><br>*Instruction: Demonstrate show card to the participant and ask which smoke tobacco products/he use*. [USE SHOWCARD]	1 = Yes; 2= No<br>If No, go to ABR_304		
302	ER_302	Do you currently smoke tobacco products daily?<br><br>*Instruction: This question is applicable for those respondents who smoke tobacco product daily.*<br>*Daily meanus" tobacco smoking is at least one time/day for almost one month or more than that*<br>*If that is the case respondant started somoking from 25 days and still running, that is regarded as everyday.*	1 = Yes; 2= No		
303	ER_303	How old were you when you **first started** smoking?	Age └─┴─┘ years<br>77 = Don't know		
304	ER_304	Do you currently use anysmokeless tobacco products such as Betel quid with zarda, zarda only or zarda with supari, Betel quid with sadapata, pan masala withtobacco, sadapata chewing, gul, Khoinee, Nossi, gutka?<br>***Instruction:*** *Ask respondent to think and answer whether h/she use any smokeless tobacco products such as Betel quid with zarda, zarda only or zarda with supari, Betel quid with sadapata, pan masala with tobacco, sadapata chewing, gul, Khoinee, Nossi, gutka. Here, only betel quid, supari and lime will not be considered. It will be considered as smokeless tobacco if respondent use Betel quid with zarda, zarda only or zarda with supari, Betel quid with sadapata, pan masala with tobacco, sadapata chewing, gul, Khoinee, Nossi, gutka.* [USE SHOWCARD]	1 = Yes; 2= No<br><br>If No, go to ABR_307		
305	ER_305	Do you currently use smokeless tobacco daily?<br>***Instruction:*** *Daily means "using smokeless tobacco is at least one time/day for almost one month or more than that.*<br>*If that is the case respondant started somoking from 25 days and still running, that is regarded as everyday.*	1 = Yes; 2= No		
306	ER_306	How old were you when you started smokeless tobacco?	Age └─┴─┘ years<br>77 = Don't know		

No	Variable	Question	Response Options	Code	Instructions/ Skip
307	ER_307	Which of your parents or guardians use any form of tobacco?	1= Neither<br>2= My father or male guardian<br>3= My mother or female guardian<br>4= Both<br>5= I do not know<br>99= Others (Specify)_______		

Physical activity

Next I am going to ask you about the time you spend doing different types of physical activity in a typical week. Please answer these questions even if you do not consider yourself to be a physically active person.
Think first about the time you spend doing work. Think of work as the things that you have to do such as paid or unpaid work, study/training, household chores, harvesting food/crops, fishing or hunting for food, seeking employment. [Insert other examples if needed]. In answering the following questions 'vigorous-intensity activities' are activities that require hard physical effort and cause large increases in breathing or heart rate, 'moderate-intensity activities' are activities that require moderate physical effort and cause small increases in breathing or heart rate.
Instruction: *Please read out the above statement for respondent. Do not drop this part. At first respondent will must think about his/her daily activities (Paid or unpaid work, study/training, household chores, harvesting food/crops, fishing or hunting for food, seeking employment). Then will think time for travelling from one place to another and at last time spent during leisure period.*
Do not forget to demonstrate show card to the respondent which will help them to respond.
It is needed to let participant remember when answer the following things
Vigorous intensity activity*: Such kind of physical activity that causes large increases in breathing or heart rate.*
Moderate-intensity activity: *Such kind of physical activity that causes small increases in breathing or heart rate.*

Now I would like to know about the 'Vigorous-intensity activities' you perform

'Vigorous-intensity activities' performed as a part of daily work / professional work/ outside daily work / professional work.

308	ER_308	Does your daily work / professional work, sports, fitness or recreational(leisure) activities involve vigorous-intensity activity that causes large increases in breathing or heart rate like [carrying or lifting heavy loads, digging or construction work, harvesting paddy, fishing using net, running, football, Kabaddi, Dariabandha, Gollachut etc.] for at least 10 minutes continuously?<br>***Instruction:*** *Ask participant to think about those vigorous-intensity activities performed as a part of professional work/ recreational activities. Those activities will be considered as vigorous-intensity activities that causes large increase in breathing or heart rate.* [USE SHOWCARD]	1 = Yes 2 = No<br>[If 'No', please go to 311]		
309	ER_309	In a typical week, on how many days do you do vigorous- intensity activities/sports/fitness as part of daily work / professional work/ recreational activities?<br>***Instruction:*** *In a typical week refers to "activities performed by respondent in a typical week". Valid response range is 1-7 days.*	|__|__| Day<br>77 = Don't know<br>[If 'Don't know', please go to ABR_311]		
310	ER_310	How much time do you spend doing vigorous-intensity activities/sports/fitness at daily work / professional work/recreational activities on a typical day?<br>***Instruction:*** *Ask respondent to think about a single day (which can be remembered easily) s/he performed vigorous-intensity activities as part of professional work/ recreational activities. Respondent will consider only those activities that are performed for at least 10 minutes or more continuously. Check unusual (more than 04 hours) values.*	__________<br>Minutes		

Now I would like to know about the 'Moderate-intensity activity' you perform					
'Moderate-intensity activity' performed as a part of daily work / professional work					
311	ER_311	Does your daily work / professional work/ spots/fitness/ recreational (leisure) work involve moderate-intensity activity that causes small increases in breathing or heart rate such as brisk walking [or carrying light loads, cycling, swimming, volleyball] for at least 10 minutes continuously? ***Instruction:** Ask participant to think about those moderate-intensity activities performed as a part of professional work/ recreational activities. Those activities will be considered as moderate-intensity activities that causes small increase in breathing or heart rate.* [USESHOWCARD]	1 = Yes 2 = No [If 'No', please go to ABR_314]		
312	ER_312	In a typical week, on how many days do you do moderate- intensity activities as part of your daily work / professional work/sports/fitness or recreational work? ***Instruction:** In a typical week refers to "activities done by respondent in a typical week". Valid response range is 1-7 days.*	|__|__| Day 77 = Don't know [If 'Don't know', please go to ABR_314]		
313	ER_313	How much time do you spend doing moderate-intensity activities at daily work / professional work/ sports/fitness or recreational work on a typical day? ***Instruction:** Ask respondent to think about a single day (which can be remembered easily) s/he performed moderate-intensity activities as part of professional work/recreational activities. Respondent will consider only those activities that are performed for at least 10 minutes or more continuously. Check unusual (more than 04 hours) values.*	________ Minutes		
**Sedentary behavior** The following question is about sitting or reclining at work, at home, getting to and from places, or with friends including time spent sitting at a desk, sitting with friends, traveling in car, bus, train, reading, playing cards or watching television, but do not include time spent sleeping.					
314	ER_314	How much time do you usually spend sitting or reclining on a typical day? ***Instruction:** Ask respondent to think about time spent on sitting or reclining at work, office, study, watching television, computer use, at kitchen, during rest.Do not include time spent during sleeping.*	________ Minutes		
315	ER_315	How much time do you watch television daily?	________ Minutes		

Section 4: Chronic Disease

No	Variable	Question	Response Options	Code	Instructions/ Skip
401	ECD_401	Has a health care provider ever told you that you have high blood pressure also called hypertension (other than during pregnancy)?	1 = Yes 2 = No		
402	ECD_402	Has a health care provider ever told you that you have heart disease?	1 = Yes 2 = No		
403	ECD_403	Has a health care provider ever told you that you have asthma/bronchitis?	1 = Yes 2 = No		

No	Variable	Question	Response Options	Code	Instructions/ Skip
404	ECD_404	Has a health care provider ever told you that you have Kidney Disease?	1 = Yes 2 = No		
405	ECD_405	Has a health care provider ever told you that you have Diabetes?	1 = Yes 2 = No		
406	ECD_406	Has a health care provider ever told you that you have stroke?	1 = Yes 2 = No		
407	ECD_407	Has a health care provider ever told you that you have cancer?	1 = Yes 2 = No		
408	ECD_408	Has a health care provider ever told you that you have mental problem?	1 = Yes 2 = No		
409	ECD_409	Has a health care provider ever told you that you have high closterol in your blood?	1 = Yes 2 = No		

Section 5: Nutritional Assessment Screening question

No	Variable	Question	Response Options	Code	Instructions/ Skip
501	EMN_501	Has food intake declined over the past 3 months due to loss of appetite, digestive problems, chewing or swallowing difficulties?	0 = Severe decrease in food intake<br>1 = Moderate decrease in food intake<br>2 = No decrease in food intake		
502	EMN_502	Weight loss during the last 3 months	0 = weight loss greater than 3 kg (6.6 lbs)<br>1 = does not know<br>2 = weight loss between 1 and 3 kg (2.2 and 6.6 lbs)<br>3 = no weight loss		
503	EMN_503	Mobility	0 = bed or chair bound<br>1 = able to get out of bed / chair but does not go out<br>2 = goes out		
504	EMN_504	Has suffered psychological stress or acute disease in the past 3 months?	1 = Yes 2 = No		
505	EMN_505	Neuropsychological problems	0 = severe dementia or depression<br>1 = mild dementia<br>2 = no psychological problems		

Section 6: Quality of Life (QoL)

No	Variable	Question	Response Options	Code	Instructions/ Skip
601	EQL_601	Mobility	1= I have no problems in walking about<br>2= I have slight problems in walking about<br>3= I have moderate problems in walking about<br>4= I have severe problems in walking about<br>5= I am unable to walk about		
602	EQL_602	Self-Care	1= I have no problems washing or dressing myself<br>2= I have slight problems washing or dressing myself<br>3= I have moderate problems washing or dressing myself<br>4= I have severe problems washing or dressing myself<br>5= I am unable to wash or dress myself		

No	Variable	Question	Response Options	Code	Instructions/ Skip
603	EQL_603	Usual activities (e.g. work, study, housework, family or leisure activities)	1= I have no problems doing my usual activities<br>2= I have slight problems doing my usual activities<br>3= I have moderate problems doing my usual activities<br>4= I have severe problems doing my usual activities<br>5= I am unable to do my usual activities		
604	EQL_604	Pain/ Discomfort	1= I have no pain or discomfort<br>2= I have slight pain or discomfort<br>3= I have moderate pain or discomfort<br>4= I have severe pain or discomfort<br>5= I have extreme pain or discomfort		
605	EQL_605	Anxiety/ Depression	1= I am not anxious or depressed<br>2= I am slightly anxious or depressed<br>3= I am moderately anxious or depressed<br>4= I am severely anxious or depressed<br>5= I am extremely anxious or depressed		
Now we would like to know how good or bad your health is today. This scale is numbered from 0 to 100.100 means the best health you can imagine. 0 means the worst health you can imagine. Mark an X on the scale to indicate how your health is today.<br>[Show the EQVAS scale to the respondant and ask to mark X]					
605	EQL_605	Health Score	|___|___|___|		

Section 7: Blood Pressure(BP)

No.	Variable	Question	Responses and code categories	Instructions/Skip
701	EBP_701	Start Time of measuring blood pressure	|____|____| : |____|____| Hr : Min	Use 24 hr format

Measurement -1 (Take the first measurement considering at least 15 minutes rest)

No.	Variable	Measurement Name	Systolic	Diastolic	Time of Measurement
702	EBP_702 A/B/C	Blood Pressure (mm of Hg)	A.|___|___|___|	B.|___|___|___|	C.|____|____|:|____|____| Hr : Min
**Measurement - 2** (Allow 3 minutes' interval between two measurements)					
			Systolic	Diastolic	Time of Measurement
703	EBP_703 A/B/C	Blood Pressure (mm of Hg)	A.|___|___|___|	B.|___|___|___|	C.|____|____|:|____|____| Hr : Min
**Measurement - 3 (**If difference of between 1st and 2nd measurement is more than 10 mm of Hg, then take 3rd measurement for Blood Pressure)					
			Systolic	Diastolic	Time of Measurement
704	EBP_704 A/B/C	Blood Pressure (mm of Hg)	A.|___|___|___|	B.|___|___|___|	C.|____|____|:|____|____| Hr : Min

No.	Variable	Question	Responses and code categories	Instructions/Skip
705	EBP_705	Blood pressure measuring machine ID	|____|____|	
706	EBP_706	Measurer's Name and Code	|____|____|____|	Write your full name and code
707	EBP_707	End Time of measuring blood pressure	|____|____| : |____|____| Hr : Min	Use 24 hr format
**[During any measurement, If Systolic BP ≥ 140 mm of Hg, Diastolic BP ≥ 90 mm of Hg - refer the respondent to appropriate health center]**				

Referral:

No.	Variable	Question	Responses and code categories	Code	Instructions/Skip
708	EBP_708	Referral needed?	1 = Yes 2 = No		If 2, go to section 8

709	EBP_709	Where referred?	1 = Upazila Health Complex<br>2 = District Hospital<br>3= Medical College Hospital<br>4= Doctor's private chamber<br>99 = Others (Specify)____________		

Section 8: Anthropometry

No	Variable	Question	Responses and code categories	Code	Instructions
801	EA_801	Start Time of anthropometry	|____|____| : |____|____| Hr : Min	Use 24 hr format	
802	EA_802	Weight Scale ID	|____|____|		
803	EA_803	Height Scale ID	|____|____|		
804	EA_804	Clothing type during weight measurement	1 = Light clothing;<br>2 = Slightly heavy clothing<br>3 = Heavy clothing		
805	EA_805	Name of measurer			
806	EA_806	Code of measurer	|____|____|		

No	Variable	Measurement	Measurement 1	Measurement 2	Measurement 3
807	EA_807 A/B/C	Height (Cm)	A.|____|____|____|.|____|	B.|____|____|____|.|____|	C.|____|____|____|.|____| If A and B differs more than 0.5 cm, take 3rd measurement
808	EA_808 A/B/C	Weight (Kg)	A.|____|____|____|.|____|	B.|____|____|____|.|____|	C.|____|____|____|.|____| If A and B differs more than 0.1kg, take 3rd measurement
809	EA_809A /B/C	Fat%	A.|____|____|____|.|____|	B.|____|____|____|.|____|	
810	EA_810A /B/C	Water%	A.|____|____|____|.|____|	B.|____|____|____|.|____|	
811	EA_811A /B/C	MUAC	A.|____|____|____|.|____|	B.|____|____|____|.|____|	C.|____|____|____|.|____| If A and B differs more than 0.5 cm, take 3rd measurement
812	EA_812 A/B/C	Calf circumference (Cm)	A.|____|____|.|____|	B.|____|____|.|____|	C.|____|____|.|____| If A and B differs more than 0.5 cm, take 3rd measurement
813	EA_813A /B/C	Waist circumference (Cm)	A.|____|____|.|____|	B.|____|____|.|____|	C.|____|____|____|.|____| If A and B differs more than 0.5 cm, take 3rd measurement
814	EA_814	Comment			
815	EA_815	End Time of measurement	|____|____|:|____|____| Hr : Min		Use 24 hr format

No.	Variable	Question	Responses and code categories		Code	Instructions/Skip
101	PM_101	Modules to be completed	A= Module 2 : Household module	1= Yes<br>2= No		
			B= Module 3 : Child module	1= Yes<br>2= No		
			C= Module 4 : Adolescent boys module	1= Yes<br>2= No		
			D= Module 5 : Adolescents girls module	1= Yes<br>2= No		
			E= Module 6 : Adult men module	1= Yes<br>2= No		
			F= Module 7 : Adule women module	1= Yes<br>2= No		
			G= Module 8 : Elderly person module	1= Yes<br>2= No		
102	PM_102	Completed modules	A= Module 2 : Household module	1= Yes<br>2= No		
			B= Module 3 : Child module	1= Yes<br>2= No		
			C= Module 4 : Adolescent boys module	1= Yes<br>2= No		
			D= Module 5 : Adolescents girls module	1= Yes<br>2= No		
			E= Module 6 : Adult men module	1= Yes<br>2= No		
			F= Module 7 : Adule women module	1= Yes<br>2= No		
			G= Module 8 : Elderly person module	1= Yes<br>2= No		
103	PM_103	Revisit needed	1= Yes ; 2= No			
104	PM_104	Date of revisit	|____|____|/|____|____|/20|____|____| DD / MM / YYYY			
105	PM_105	Date of interview completion	|____|____|/|____|____|/20|____|____| DD / MM / YYYY			
106	PM_106	Time of interview completion	|____|____|: |____|____| HH : MM			Use 24H format
107	PM_107	Comment of interviewer				

Thank you for your participation and cooperation
