# Supplementary material for: Ten-years cardiovascular risk among Bangladeshi population using non-laboratory-based risk chart of the World Health Organization: Findings from a nationally representative survey
Source: PLoS One. 2021 May 26;16(5):e0251967. doi: 10.1371/journal.pone.0251967 (PMC8153482; doi:10.1371/journal.pone.0251967)
Supplement: S2 File — (PDF) [file pone.0251967.s005.pdf]

অংশগ্রহণকারীর আইডি:|___|___|___|___|___|___|___|___|___|___|___|___|___|___|___|___|

খাদ্য নিরাপত্তা ও পুষ্টি বিষয়ক সার্ভিলেন্স

খানার সদস্য নির্বাচনের প্রশ্নমালা

পরিচ্ছেদ ১: তথ্যগ্রহণ সম্পর্কিত তথ্য

নম্বর	সূচক	প্রশ্ন	উত্তর		কোড	নির্দেশনা
101	HS_101	সাক্ষাৎকার গ্রহণকারীর নাম এবং আইডি	|___|___|			আপনার পুরো নাম এবং কোড লিখুন
102	HS_102	ক্লাষ্টারের নাম ও কোড	____________ |___|___|			সাক্ষাৎকার শুরুর আগে পুরুন করুন
104	HS_104	ইউনিয়নের নাম ও কোড	____________ |___|___|			
106	HS_106	উপজেলার নাম ও কোড	____________ |___|___|			
108	HS_108	জেলার নাম ও কোড	____________ |___|___|			
110	HS_110	বিভাগের নাম ও কোড	____________ |___|___|			
112	HS_112	খানার কোড	|___|___|___|___|___|			
113	HS_113	খানার সর্বমোট সদস্য সংখ্যা	|___|___|			
114	HS_114	খানা প্রধানের নাম	____________			
	HS_114A	খানা প্রধানের বাবা/স্বামীর নাম (ডাক নামসহ)	____________			
104	HS_104A	বাড়ির নাম	____________			
	HS_104B	গ্রাম/মহল্লার নাম	____________			
115	HS_115A	খানায় বসবাসরত সর্বমোট সদস্য	|___|___|			খানার এমন সদস্যদের বাদ দিন যারা স্থায়ীভাবে বাহিরে থাকে
			A. মোট	B. লিঙ্গ		
	HS_115B	0-5 বছরের কম বয়সের শিশু	|___|			
	HS_115BA	নামঃ____________		1 = পুরুষ 2 = মহিলা		
	HS_115BB	নামঃ____________		1 = পুরুষ 2 = মহিলা		
	HS_115BC	নামঃ____________		1 = পুরুষ 2 = মহিলা		
	HS_115C	6-9 বছরের শিশু	|___|			
	HS_115CA	নামঃ____________		1 = পুরুষ 2 = মহিলা		
	HS_115CB	নামঃ____________		1 = পুরুষ 2 = মহিলা		
	HS_115CC	নামঃ____________		1 = পুরুষ 2 = মহিলা		
	HS_115D	10-19 বছর বয়সের কিশোর-কিশোরী	|___|			
	HS_115DA	নামঃ____________		1 = পুরুষ 2 = মহিলা		
	HS_115DB	নামঃ____________		1 = পুরুষ 2 = মহিলা		
	HS_115DC	নামঃ____________		1 = পুরুষ 2 = মহিলা		
	HS_115E	20-59 বছর বয়সের পুরুষ/মহিলা	|___|			
	HS_115EA	নামঃ____________		1 = পুরুষ 2 = মহিলা		

অংশগ্রহণকারীর আইডি:|___|___|___|___|___|___|___|___|___|___|___|___|___|___|___|___|

নম্বর	সূচক	প্রশ্ন	উত্তর		কোড	নির্দেশনা
	HS_115EB	নামঃ___________________		1 = পুরুষ 2 = মহিলা		
	HS_115EC	নামঃ___________________		1 = পুরুষ 2 = মহিলা		
	HS_115F	60 বা 60 বছরের বেশী বয়সের পুরুষ/মহিলা	|___|			
	HS_115FA	নামঃ___________________		1 = পুরুষ 2 = মহিলা		
	HS_115FB	নামঃ___________________		1 = পুরুষ 2 = মহিলা		
	HS_115FC	নামঃ___________________		1 = পুরুষ 2 = মহিলা		
116	HS_116	খানা প্রধানের যোগাযোগের নাম্বার	|___|___|___|___|___|___|___|___|___|___|___|			
117	HS_117	খানা প্রধানের সাথে যোগাযোগের বিকল্প নাম্বার	|___|___|___|___|___|___|___|___|___|___|___|			
118	HS_118	পুনরায় সাক্ষাৎকার গ্রহণের প্রয়োজন আছে কি?	1 = হ্যাঁ 2 = না			যদি না হয়, তাহলে অন্য সেকশনে যান
119	HS_119	পুনরায় সাক্ষাৎকার গ্রহণের কারন	1 = অনুপস্থিত 2 = অসম্পুর্ন সাক্ষাৎকার 99 = অন্যান্য (নির্দিষ্ট করুন) _______			

মডিউল-১: সাধারণ তথ্যাবলী

নম্বর	সূচক	প্রশ্ন	উত্তর		কোড	নির্দেশনা
101	GI_101	সাক্ষাৎকার গ্রহণকারীর নাম ও আইডি	|____|____|			আপনার পুরো নাম এবং কোড লিখুন
102	GI_102	সাক্ষাৎকার গ্রহণের তারিখ	|___|___|/|___|___|/20|___|___| দিন / মাস / বছর			তথ্যসংগ্রহ শুরু করার পূর্বে সিডিউলার দেখে পূরণ করুন
103	GI_103	খানার নাম্বার	|____|____|____|____|____|			
104	GI_104	কোন্ কোন্ মডিউলের তথ্য নিতে হবে?	A= মডিউল 2 : খানার তথ্য	1 = হ্যাঁ<br>2 = না		
			B= মডিউল 3 : শিশু তথ্য	1 = হ্যাঁ<br>2 = না		
			C= মডিউল 4 : কিশোরের তথ্য	1 = হ্যাঁ<br>2 = না		
			D= মডিউল 5 : কিশোরীর তথ্য	1 = হ্যাঁ<br>2 = না		
			E= মডিউল 6 : প্রাপ্তবয়স্ক পুরুষের তথ্য	1 = হ্যাঁ<br>2 = না		
			F= মডিউল 7 : প্রাপ্তবয়স্ক মহিলার তথ্য	1 = হ্যাঁ<br>2 = না		
			G = মডিউল 8: বৃদ্ধ/বৃদ্ধার তথ্য	1 = হ্যাঁ<br>2 = না		
105	GI_105	সাক্ষাৎকার শুরুর সময়	|____|____|: |____|____| ঘন্ট : মিনিট			24 ঘন্টর ফরমেট ব্যবহার করুন

অংশগ্রহণকারীর আইডি:|___|___|___|___|___|___|___|___|___|___|___|___|___|___|___|___|

খাদ্য নিরাপত্তা ও পুষ্টি বিষয়ক সার্ভিলেন্স

মডিউল 2: খানাপ্রধানের প্রশ্নপত্র

মডিউল-১: সাধারণ তথ্যাবলী এবং খানার আর্থ-সামাজিক অবস্থা

নম্বর	সূচক	প্রশ্ন	উত্তর	কোড	নির্দেশনা
101	SE_101	সাক্ষাৎকার গ্রহণকারীর নাম ও কোড	|____|____|		সাক্ষাৎকার গ্রহণকারীর পুরো নাম ও কোড লিখুন?
102	SE_102	খানা প্রধানের নাম (ডাক নামসহ)			
103	SE_103	খানা প্রধানের লিঙ্গ	1 = পুরুষ 2 = মহিলা		
104	SE_104	খানা প্রধানের জন্ম তারিখ	|____|____|/|____|____|/20|____|____| দিন মাস বছর		
105	SE_105	খানা প্রধানের বয়স (পূর্ণ বছর)	|____|____|____| বছর		
106	SE_106	আপনি সর্বমোট কত বছর প্রাতিষ্ঠানিক শিক্ষা গ্রহণ করেছেন?<br><br>(সম্পূর্ণ বছর লিখুন। প্রথম শ্রেণীর নিচে এবং উপানুষ্ঠিক শিক্ষা অন্তর্ভুক্ত হবে না)<br>*নির্দেশনাঃ*<br>*তথ্য প্রদানকারীর সর্বমোট কত বছর প্রাতিষ্ঠানিক শিক্ষা সম্পন্ন করেছেন তা লিপিবদ্ধ করুন। প্রাতিষ্ঠানিক শিক্ষা না থাকলে 00 লিখুন।*	|____|____| বছর<br><br>প্রাথমিক শিক্ষা/এবতেদায়ী = 5<br>মাধ্যমিক/দাখিল =10<br>উচ্চ মাধ্যমিক/ ডিপ্লোমা/আলিম= 12<br>স্নাতক/ফাজিল =16<br>স্নাতাকোত্তর/কামিল/দাওরা =18		
107	SE_107	গত 12 মাসে আপনার পেশা কি ছিল?<br><br>*নির্দেশনাঃ*<br>যদি তথ্য প্রদানকারী গত 12 মাসে একাধিক পেশায় নিয়ুক্ত থাকেন তাহলে তিনি যে পেশাটিতে বেশী সময় ব্যয় করেছেন সেটিকে প্রধান পেশা হিসাবে বিবেচনা করুন এবং তা লিপিবদ্ধ করুন।	1 = কৃষিকাজ (ধান)<br>2 = কৃষিকাজ (ধান ছাড়া অন্যান্য)<br>3 = কৃষি দিনমজুর<br>4 = অদক্ষ দিনমজুর<br>5 = দক্ষ দিনমজুর<br>6 = রিক্সা/ ভ্যান/ ঠেলাগাড়ী/ বেবীট্যাক্সি ড্রাইভার/ নৌকার মাঝি<br>7 = জেলে<br>8 = চাকুরীজীবি<br>9 = পেশাজীবি<br>10 = ব্যবসায়ী<br>11 = ক্ষুদে ব্যবসায়ী<br>12 = গৃহপরিচারিকা<br>13 = জুমচাষী<br>14 = উপার্জন করে না<br>15 = হাঁস/ মুরগী পালন/ পশু পালন<br>16 = হস্তশিল্প<br>17 = শাক-সব্জি চাষ<br>18 = মৎস চাষ<br>19 = গৃহিনী<br>20 = ছাত্র/ছাত্রী<br>77 = জানিনা<br>66 = বয়স 6 বছরের কম<br>99 = অন্যান্য (নির্দিষ্ট করুন) ___________		
108	SE_108	আপনার খানার নিজস্ব বসতভিটার জমির পরিমাণ কত?	|____|____|____|____| শতাংশ		
109	SE_109	আপনাদের খানার নিজস্ব চাষাবাদের জমির পরিমাণ কত?	|____|____|____|____| শতাংশ		

অংশগ্রহণকারীর আইডি:|___|___|___|___|___|___|___|___|___|___|___|___|___|___|___|___|

নম্বর	সূচক	প্রশ্ন	উত্তর		কোড	নির্দেশনা
110	SE_110	আপনার খানার রান্নার কাজে প্রধানত কি ধরনের জ্বালানী ব্যবহার করেন?	1 = বিদ্যুৎ<br>2 = এলপিজি<br>3 = পাইপযুক্ত প্রাকৃতিক গ্যাস<br>4 = কেরোসিন<br>5 = কয়লা/লিগনাইট<br>6 = চারকোল	7 = কাঠ<br>8 = পাটখড়ি/খড়/নাড়া/পাতা<br>9 = গোবর<br>10 = বায়োগ্যাস<br>99 = অন্যান্য (নির্দিষ্ট করুন)<br>__________		
**আপনার খানায় বর্তমানে গৃহপালিত পশু বা পাখি আছে?<br>যদি থাকে, তবে কতগুলো?**						
111			A. 1 = হ্যাঁ 2 = না	B. সংখ্যা (যদি হ্যাঁ হয়)		
	SE_111A	গরু/মহিষ	1 = হ্যাঁ 2 = না	|___|___|___|		
	SE_111B	ভেড়া/ছাগল/শুকর	1 = হ্যাঁ 2 = না	|___|___|___|		
	SE_111C	মুরগী/হাঁস/রাজহাঁস	1 = হ্যাঁ 2 = না	|___|___|___|		
	SE_111D	ছোট পশু/পাখি (যেমন: *খরগোশ, কবুতর ইত্যাদি*)	1 = হ্যাঁ 2 = না	|___|___|___|		
	SE_111E	অন্যান্য (নির্দিষ্ট করুন)---	1 = হ্যাঁ 2 = না	|___|___|___|		
**আপনার খানায় নিম্নে বর্ণিত কোন্ কোন্ জিনিসগুলো সচল অবস্থায় আছে কি?**						
112	SE_112A	বিদ্যুৎ সংযোগ	1 = হ্যাঁ 2 = না			
	SE_112B	সৌর বিদ্যুৎ	1 = হ্যাঁ 2 = না			
	SE_112C	রেডিও/টেলিভিশন	1 = হ্যাঁ 2 = না			
	SE_112D	টেলিফোন/মোবাইল ফোন	1 = হ্যাঁ 2 = না			
	SE_112E	ফ্যান	1 = হ্যাঁ 2 = না			
	SE_112F	খাট/চৌকি	1 = হ্যাঁ 2 = না			
	SE_112G	আলমীরা/ওয়াড্রব/শোকেস	1 = হ্যাঁ 2 = না			
	SE_112H	রেফ্রিজারেটর/ফ্রিজ	1 = হ্যাঁ 2 = না			
	SE_112I	টেবিল/ চেয়ার	1 = হ্যাঁ 2 = না			
	SE_112J	ঘড়ি/দেয়াল ঘড়ি	1 = হ্যাঁ 2 = না			
	SE_112K	বাই-সাইকেল	1 = হ্যাঁ 2 = না			
	SE_112L	মোটরসাইকেল/স্কুটার/টেম্পু	1 = হ্যাঁ 2 = না			
	SE_112M	পশুচালিত গাড়ি	1 = হ্যাঁ 2 = না			
	SE_112N	কার/ট্রাক	1 = হ্যাঁ 2 = না			
	SE_112O	নৌকা	1 = হ্যাঁ 2 = না			
	SE_112P	ইঞ্জিনচালিত নৌকা	1 = হ্যাঁ 2 = না			
	SE_112Q	রিক্সা/ভ্যান	1 = হ্যাঁ 2 = না			
	SE_112R	পাওয়ার টিলার	1 = হ্যাঁ 2 = না			
	SE_112S	শ্যালো মেশিন	1 = হ্যাঁ 2 = না			
	SE_112T	লাঙ্গল	1 = হ্যাঁ 2 = না			
	SE_112U	আইপিএস/ বৈদ্যুতিক জেনারেটর	1 = হ্যাঁ 2 = না			
	SE_112V	মাছ ধরার জাল	1 = হ্যাঁ 2 = না			

নম্বর	সূচক	প্রশ্ন	উত্তর	কোড	নির্দেশনা
113	SE_113	আপনার প্রধান বসত ঘরের দেয়াল প্রধানত কি দিয়ে তৈরী?<br><br>(পর্যবেক্ষণ করে লিপিবদ্ধ করুন)	**স্বাভাবিক দেয়াল:**<br>1 = দেয়াল নেই<br>2 = বেত, ইক্ষু জাতীয় পাতা/ তালপাতা / গাছের গুড়ি<br>3 = কাদা মাটি<br>**প্রাথমিক পর্যায়ের দেয়াল:**<br>4 = বাঁশও মাটি<br>5 = পাথর ও মাটি<br>6 = প্লাইউড<br>7 = পিচ বোর্ড/ পলিথিন<br>**পরিপূর্ণ দেয়াল:**<br>8 = টিন<br>9 = ইট-সিমেন্ট<br>10 = পাথর ও চুন/ সিমেন্ট<br>11 = কাঠের তক্তা/ ফলক<br>99 = অন্যান্য (নির্দিষ্ট করুন) __________		পর্যবেক্ষণ করে লিপিবদ্ধ করুন
114	SE_114	আপনার প্রধান বসত ঘরের চাল/ছাদ প্রধানত কি দিয়ে তৈরী?<br><br>(পর্যবেক্ষণ করে লিপিবদ্ধ করুন)	**স্বাভাবিক ছাদ:**<br>1 = ছাদ নেই<br>2 = খড়/তালপাতা/ গোল পাতা প্রভৃতি<br>**কাঁচা ছাদ:**<br>3 = বাঁশ ও মাটি<br>4 = কাঠের তক্তা<br>5 = পিচ বোর্ড/ পলিথিন<br>**পরিপূর্ণ ছাদ:**<br>6 = টিন<br>7 = কাঠ<br>8 = সিরামিক টাইল্স<br>9 = ইট-সিমেন্ট<br>10 = কাঠের ফলক<br>99 = অন্যান্য (নির্দিষ্ট করুন) __________		
115	SE_115	আপনার প্রধান বসত ঘরের মেঝে প্রধানত কি দিয়ে তৈরী?<br><br>(পর্যবেক্ষণ করে লিপিবদ্ধ করুন)	**কাঁচা মেঝে:**<br>1 = মাটি/বালি<br>**প্রাথমিক পর্যায়ের মেঝে:**<br>2 = কাঠের তক্তা<br>3 = তাল গাছ/বাঁশ<br>**পরিপূর্ণ মেঝে:**<br>4 = সিরামিক টাইল্স<br>5 = ইট-সিমেন্ট<br>6 = মোজাইক<br>99 = অন্যান্য (নির্দিষ্ট করুন) __________		
116	SE_116	আপনার খানায় বসবাসের জন্য কয়টি কক্ষ আছে?<br>(গৃহকর্মীদের কক্ষসহ)	|_____|_____|		
117	SE_117	বর্তমানে আপনার খানার কোন সদস্য কি উল্লেখিত সরকারী কর্মসূচি থেকে কোন সুবিধা গ্রহণ করছেন?<br>(একাধিক উত্তর হতে পারে, যদি 'কাজের বিনিময়ে টাকা' কোড করা হয়, তবে যাচই করুন তা চলমান সরকারী কর্মসূচি কি না।)	1 = কিছুই পাই না<br>2 = শিক্ষার জন্য টাকা<br>3 = মুক্তিযোদ্ধা ভাতা<br>4 = বয়স্ক ভাতা<br>5 = ভিজিডি<br>6 = বিধাবা ভাতা<br>7 = 100 দিনের কর্মসূচি (কাজের বিনিময়ে টাকা, বর্তমান কর্মসূচি)<br>8 = ভিজিএফ<br>9 = ন্যাশনাল সার্ভিস কার্যক্রম<br>77 = জানিনা<br>99 = অন্যান্য (নির্দিষ্ট করুন) __________		
118	SE_118	ধর্ম<br>*নির্দেশনাঃ*<br>*উত্তরতাদা যে ধর্মের অনুসারী তা লিপিবদ্ধ করুন।*	1 = ইসলাম<br>2 = হিন্দু<br>3 = খ্রিস্টান<br>4 = বৌদ্ধ<br>99 = অন্যান্য (নির্দিষ্ট করুন)<br>__________		
119	SE_119	আপনি কোন জাতিসত্ত্বার অন্তর্ভূক্ত?	|____|____|		

অংশগ্রহণকারীর আইডি:|___|___|___|___|___|___|___|___|___|___|___|___|___|___|___|___|

খাদ্য নিরাপত্তা ও পুষ্টি বিষয়ক সার্ভিলেন্স

নম্বর	সূচক	প্রশ্ন	উত্তর	কোড	নির্দেশনা
**কোডঃ** 1 = চাকমা; 2 = মারমা; 3 = ত্রিপুরা; 4 = মুরং; 5 = তনচঙ্গ্যা; 6 = বম; 7 = পাংখো; 8 = চাক; 9 = খিয়াং; 10 = খুমি; 11 = লুসাই; 12 = কোচ; 13 = সাঁওতাল; 14 = ডালু; 15 = লুসাই; 16 = রাখাইন; 17 = মনিপুরী; 18 = গারো; 19 = হাজং; 20 = খাসিয়া; 21 = মং; 22 = ওরাও; 23 = বর্মন; 24 = পাহাড়ি; 25 = মাল পাহাড়ি; 26 = মুন্ডা; 27 = খোল; 28 = বাঙ্গালী; 99 = অন্যান্য (নির্দিষ্ট করুন) ___________					
120	SE_120	এই খানায় থাকেন না (যিনি দেশের বাহিরে বা দেশের মধ্যে অন্য কোথাও থাকেন) এমন কারও নিকট থেকে গত 6 মাসে টাকা পেয়েছেন কি?	1 = না<br>2 = হ্যাঁ<br>77 = জানিনা		যদি না হয় তাহলে 122 এ যান
121	SE_121	প্রতি মাসে আপনি গড়ে কত টাকা পেয়েছেন? (গত 6 মাসকে বিবেচনা করুন)	|____|____|____|____|____|____| টাকা		
122	SE_122	আপনার পরিবারের গড় মাসিক আয় কত?	|___|___|___|___|___|___|___| টাকা<br>88 = জানাতে অসম্মতি		

অংশগ্রহণকারীর আইডি:|___|___|___|___|___|___|___|___|___|___|___|___|___|___|___|___|

খাদ্য নিরাপত্তা ও পুষ্টি বিষয়ক সার্ভিলেন্স

পরিচ্ছেদ 2: খানার পানি সরবরাহ, পয়ঃনিস্কাশন ও পরিষ্কার-পরিচ্ছন্নতা

নম্বর	সূচক	প্রশ্ন	উত্তর	কোড	নির্দেশনা
201	WH_201	আপনারা প্রধানত কিসের পানি পান করেন?	*পাইপের পানি*<br>1 = ঘরের ভিতর পাইপ<br>2 = বাড়ীর উঠানে পাইপ<br>3 = পাবলিক ট্যাপ<br>*টিউবওয়েল অথবা গর্তপাইপ*<br>4 = যৌথ<br>5 = একক<br>*কুয়ার পানি*<br>6 = ঢাকনাযুক্ত<br>7 = ঢাকনা ছাড়া<br>8 = বৃষ্টির পানি<br>**ভূপৃষ্ঠ পানি**<br>9 = পুকুর/নদী/ খাল/হাওর/সেচ নালা<br>10 = পানির ট্যাংক<br>*ঝর্ণার পানি*<br>11 = ঢাকনাযুক্ত<br>12 = ঢাকনা ছাড়া<br>99 = অন্যান্য(নির্দিষ্ট করুন)<br>__________		
202	WH_202	আপনার খানায় কোন সাবান আছে কি?	1 = হ্যাঁ 2 = না		
203	WH_203	গত 24 ঘন্টায় আপনি কোন সাবান ব্যবহার করেছেন কি?	1 = হ্যাঁ 2 = না		যদি না হয় তাহলে 205 এ যান
204	WH_204	যদি আপনি আজ বা গতকাল সাবান ব্যবহার করে থাকেন, তবে কি কাজে ব্যবহার করেছিলেন?<br><br>*উত্তরদাতা যদি বলেন নিজের বা শিশুর হাত ধুয়েছি, তবে কারণ গুলো জানুন। উত্তর কোড পড়বেন না।*<br><br>*(একাধিক উত্তর হতে পারে, উত্তর গুলো পড়বেন না, যতক্ষণ সঠিক উত্তর না পান ততক্ষণ আর কি করেছেন এভাবে জিজ্ঞাসা করুন।)*	1 = কাপড় ধোয়ার জন্য	1 = হ্যাঁ<br>2 = না	
			2 = নিজে গোসলের জন্য	1 = হ্যাঁ<br>2 = না	
			3 = শিশুর গোসলের জন্য	1 = হ্যাঁ<br>2 = না	
			4 = শিশুর নিম্নাংশ ধোয়ানোর জন্য	1 = হ্যাঁ<br>2 = না	
			5 = শিশুর হাত ধোয়ানোর জন্য	1 = হ্যাঁ<br>2 = না	
			6 = শৌচকাজের পর হাত ধোয়ার জন্য	1 = হ্যাঁ<br>2 = না	
			7 = শিশুকে পরিষ্কার করার পর হাত ধোঁয়ার জন্য	1 = হ্যাঁ<br>2 = না	
			8 = শিশুকে খাওয়ানোর আগে হাত ধোয়ার জন্য	1 = হ্যাঁ<br>2 = না	
			9 = খাবার তৈরীর আগে হাত ধোয়ার জন্য	1 = হ্যাঁ<br>2 = না	
			10 = খাবার আগে হাত ধোয়ার জন্য	1 = হ্যাঁ<br>2 = না	
			11 = অন্যান্য(নির্দিষ্ট করুন)	1 = হ্যাঁ<br>2 = না	
205	WH_205	আপনারা কি ধরনের পায়খানা ব্যবহার করেন?<br><br>(পর্যবেক্ষণ করে লিপিবদ্ধ করুন)	1 = স্যুয়ারেজ লাইনের সাথে যুক্ত<br>2 = স্যাপ্টিক ট্যাংকসহ পায়খানা<br>3 = ওয়াটার সিল সহ রিংস্লাব<br>4 = ওয়াটার সিল ছাড়া রিংস্লাব<br>5 = আবদ্ধ পিট<br>6 = খোলাপিট<br>7 = ঝুলন্ত পায়খানা		যদি উত্তর 8 হয় তাহলে WH_207এ যান।

নম্বর	সূচক	প্রশ্ন	উত্তর	কোড	নির্দেশনা
			8 = পায়খানা নাই (রাস্তা/নদীর ধারে/খোলা মাঠে/ যেখানে-সেখানে/ঝোপ-জঙ্গল)<br>99 = অন্যান্য (নির্দিষ্ট করুন) __________		
206	WH_206	আপনারা কি নিজস্ব/যৌথ/পাবলিক টয়লেট ব্যবহার করেন?<br><br>*(পায়খানাটি যেখানেই অবস্থিত হোক না কেন, প্রশ্নটি করতে হবে।*<br>**যৌথ টয়লেট:** নির্দিষ্ট সংখ্যক ব্যবহারকারীর মালিকানাধীন টয়লেট।<br>**পাবলিক টয়লেট:** ব্যবহারকারীর সংখ্যা নির্দিষ্ট নয় এমন টয়লেট।)	1 = নিজের<br>2 = যৌথভাবে<br>3 = পাবলিক টয়লেট<br>77 = জানিনা		
207	WH_207	আপনি কি জুতা পরে পায়খানায় যান?	1 = হ্যাঁ 2 = না		
208	WH_208	আপনি সাধারণতঃ কোথায় হাত ধৌত করেন, অনুগ্রহ করে আমাকে দেখাবেন?<br><br>*(জিজ্ঞাসা করুন ও দেখুন)*	1 = ঘরের ভিতর আধুনিক পাইপ<br>2 = পায়খানার ভিতর/পায়খানার নিকট<br>3 = রান্না ঘরের ভিতর/রান্নার স্থানের পাশে<br>4 = আঙ্গিনার ভিতরে<br>5 = আঙ্গিনার বাইরে<br>6 = নির্দিষ্ট স্থান নেই<br>8 = দেখার জন্য অনুমতি দেয়নি		যদি উত্তর 6/8 হয় তাহলে পরিচ্ছেদে 3A এ যান
209	WH_209	উক্ত স্থানে সাবান, ডিটারজেন্ট বা স্থানীয়ভাবে ব্যবহৃত হাত পরিষ্কারের কোন দ্রব্য আছে কিনা, তা পর্যবেক্ষণ করুন।<br><br>*উক্ত দ্রব্যগুলো যদি নির্দিষ্ট স্থানে থাকে বা সাক্ষাৎকার প্রদানকারী যদি 1 মিনিটের মধ্যে এনে রাখে, তবে হ্যাঁ কোড করুন।*<br>*কিছুই না পেলে/সাক্ষাৎকার প্রদানকারী 1 মিনিটের মধ্যে এনে না রাখলে, কিছুই নেই---5 কোড করুন।*	1 = সাবান<br>2 = ডিটারজেন্ট<br>3 = ছাই<br>4 = মাটি/বালি<br>5 = কিছুই নেই<br>99 = অন্যান্য (নির্দিষ্ট করুন)<br>__________		
210	WH_210	উক্ত স্থানে পানি আছে কিনা, তা পর্যবেক্ষণ করুন?<br><br>*সাক্ষাৎকার গ্রহণকারী টেপ ছেড়ে/পানির পাত্রে পানি পেলে/ সাক্ষাৎকার প্রদানকারী যদি 1 মিনিটের মধ্যে পানি এনে রাখে, তবে 'হ্যাঁ' কোড করুন ।*<br>*পানি না পেলে/সাক্ষাৎকার প্রদানকারী 1 মিনিটের মধ্যে পানি না রাখলে, 'না' কোড করুন।*	1 = হ্যাঁ 2 = না		
211	WH_211	উক্ত স্থানে হাত ধোয়ার জন্য কোন উপকরণ- যেমনঃ- টেপ, বেসিন, বালতি, কলস, হাড়ি, বদনা, সিন্ক, টিপ্পি টেপ ইত্যাদি আছে কিনা, তা পর্যবেক্ষণ করুন?<br><br>*উক্ত দ্রব্যগুলো যদি যথাস্থানে থাকে/ সাক্ষাৎকার প্রদানকারী 1 মিনিটের এনে রাখেন, তবে 'হ্যাঁ' কোড করুন।*<br>*উক্ত দ্রব্যগুলো না পেলে/সাক্ষাৎকার প্রদানকারী 1 মিনিটের মধ্যে এনে না রাখলে, 'না' কোড করুন।*	1 = হ্যাঁ 2 = না		

অংশগ্রহণকারীর আইডি:|___|___|___|___|___|___|___|___|___|___|___|___|___|___|___|___|

পরিচ্ছেদ 3Aঃ খাদ্যনিরাপত্তা এবং মানিয়ে নেয়ার কৌশল

এখন, আমি আপনাকে আপনার খানার খাদ্য নিরাপত্তা বিষয়ক কিছু প্রশ্ন জিজ্ঞাসা করার জন্য আপনার আরও কিছু সময় নিব। আমি আপনাকে পূর্বের 4 সপ্তাহের কথা চিন্তা করে বলতে বলব যে, কতটা ঘনঘন এবং কিভাবে আপনার খানায় খাদ্যের নিরাপত্তাহীনতার অভিজ্ঞতা হয়েছে। মনে রাখবেন যে, আমি খানা বলতে বুঝাতে চাইছি ঐ সকল সদস্য যারা সাধারণত একই হাড়ি থেকে খাবার ভাগাভাগি করে খান। যদি আপনার কোন প্রশ্ন থাকে তাহলে আপনি আমায় থামিয়ে জিজ্ঞাসা করবেন।

নম্বর	সূচক	প্রশ্ন	উত্তর	কোড	নির্দেশনা
301	FS_301	গত 4 সপ্তাহে আপনি আপনার খানায় পর্যাপ্ত খাদ্য নাই অথবা কিভাবে যোগাড় করবেন, এই দুঃচিন্তায় ছিলেন কি?	0 = কখনো না<br>1 = হ্যাঁ, হলে কতবার কদাচিৎ (একবার অথবা দুইবার)<br>2 = মাঝেমধ্যে (3 থেকে 10 বার)<br>3 = প্রায়ই (10 বার এর অধিক)		
302	FS_302	গত 4 সপ্তাহে এমন হয়েছিলে কি, আপনি বা আপনার খানার কোন সদস্য খাবার অথবা টাকার অভাবের কারণে পছন্দের খাবার খেতে পারেনি?	0 = কখনো না<br>1 = হ্যাঁ, হলে কতবার কদাচিৎ (একবার অথবা দুইবার)<br>2 = মাঝেমধ্যে (3 থেকে 10 বার)<br>3 = প্রায়ই (10 বার এর অধিক)		
303	FS_303	গত 4 সপ্তাহে আপনি বা খানার কোনো সদস্যকে অভাবের কারণে শুধুমাত্র ভাত খেয়ে থাকতে হয়েছিল কি *(লবণ, মরিচ ও পিঁয়াজ ইত্যাদি দিয়ে)?*	0 = কখনো না<br>1 = হ্যাঁ, হলে কতবার কদাচিৎ (একবার অথবা দুইবার)<br>2 = মাঝেমধ্যে (3 থেকে 10 বার)<br>3 = প্রায়ই (10 বার এর অধিক)		
304	FS_304	গত 4 সপ্তাহে আপনি বা খানার কোনো সদস্যকে অভাবের কারণে এমন কোন খাবার খেতে হয়েছিল যা সাধারনত খান না (যেমনঃ বন্য/অনাবাদী খাবার/ ভাতের পরিবর্তে অন্য কোন খাবার, যা সাধারনত খান না।)?	0 = কখনো না<br>1 = হ্যাঁ, হলে কতবার কদাচিৎ (একবার অথবা দুইবার)<br>2 = মাঝেমধ্যে (3 থেকে 10 বার)<br>3 = প্রায়ই (10 বার এর অধিক)		
305	FS_305	খানায় পর্যাপ্ত খাবার অথবা খাবার কেনার জন্য টাকা না থাকার কারণে গত 4 সপ্তাহে আপনি বা আপনার খানার কোনো সদস্যকে কি কোন বেলা (যেমন: সকাল, দুপুর অথবা রাতের খাবার) প্রয়োজনের তুলনায় কম খেতে হয়েছিল?	0 = কখনো না<br>1 = হ্যাঁ, হলে কতবার কদাচিৎ (একবার অথবা দুইবার)<br>2 = মাঝেমধ্যে (3 থেকে 10 বার)<br>3 = প্রায়ই (10 বার এর অধিক)		
306	FS_306	গত 4 সপ্তাহে আপনি বা আপনার খানার কোনো সদস্যকে খাবারের অভাব অথবা খাবার কেনার টাকা না থাকার কারণে কি কোন বেলা না খেয়ে থাকতে হয়েছিল?	0 = কখনো না<br>1 = হ্যাঁ, হলে কতবার কদাচিৎ (একবার অথবা দুইবার)<br>2 = মাঝেমধ্যে (3 থেকে 10 বার)<br>3 = প্রায়ই (10 বার এর অধিক)		
307	FS_307	গত 4 সপ্তাহে কখনও কি আপনার ঘরের খাবার শেষ হয়ে গিয়েছিল এবং ঐ দিন খাবার কেনার জন্য কোনো টাকাও ছিল না?	0 = কখনো না<br>1 = হ্যাঁ, হলে কতবার কদাচিৎ (একবার অথবা দুইবার)<br>2 = মাঝেমধ্যে (3 থেকে 10 বার)<br>3 = প্রায়ই (10 বার এর অধিক)		
308	FS_308	গত 4 সপ্তাহে কি কখনও এমন হয়েছিল যে, আপনাকে অথবা খানার কোনো সদস্যকে অভাবের কারণে ক্ষুধার্ত/অভুক্ত অবস্থায় রাতে ঘুমাতে যেতে হয়েছিল?	0 = কখনো না<br>1 = হ্যাঁ, হলে কতবার কদাচিৎ (একবার অথবা দুইবার)<br>2 = মাঝেমধ্যে (3 থেকে 10 বার)<br>3 = প্রায়ই (10 বার এর অধিক)		

<table>
<tr><th>নম্বর</th><th>সূচক</th><th colspan="2">প্রশ্ন</th><th colspan="2">উত্তর</th><th>কোড</th><th>নির্দেশনা</th></tr>
<tr><td>309</td><td>FS_309</td><td colspan="2">গত 4 সপ্তাহে কখনও এমন হয়েছিল যে আপনি বা খানার কোনো সদস্যকে খাবারের অভাবের কারণে না খেয়ে সারা দিন-রাত কাটাতে হয়েছিল?</td><td colspan="2">0 = কখনো না
1 = হ্যাঁ, হলে কতবার কদাচিৎ (একবার অথবা দুইবার)
2 = মাঝেমধ্যে (3 থেকে 10 বার)
3 = প্রায়ই (10 বার এর অধিক)</td><td></td><td>উত্তর 0 হলে 310 এ যান</td></tr>
<tr><td rowspan="6">310</td><td rowspan="6">FS_310</td><td rowspan="6">গত 4 সপ্তাহে মধ্যে খাদ্য ঘাটতি মোকাবেলার জন্য নিম্নের কাজ গুলো করেছিলেন কি?</td><td>A. সম্পদ বিক্রী/বন্ধক দেওয়া</td><td>1 = হ্যাঁ</td><td>2 = না</td><td></td><td></td></tr>
<tr><td>B. নিম্ন মানের খাবার খাওয়া</td><td>1 = হ্যাঁ</td><td>2 = না</td><td rowspan="5"></td><td rowspan="5"></td></tr>
<tr><td>C. কম ধরনের/পদের খাবার খাওয়া</td><td>1 = হ্যাঁ</td><td>2 = না</td></tr>
<tr><td>D. সদস্যদের লেখা পড়া বন্ধ করে দেওয়া</td><td>1 = হ্যাঁ</td><td>2 = না</td></tr>
<tr><td>E. খাবার ধার করা</td><td>1 = হ্যাঁ</td><td>2 = না</td></tr>
<tr><td>F. টাকা ধার করা</td><td>1 = হ্যাঁ</td><td>2 = না</td></tr>
</table>

অংশগ্রহণকারীর আইডি:|___|___|___|___|___|___|___|___|___|___|___|___|___|___|___|___|

খাদ্য নিরাপত্তা ও পুষ্টি বিষয়ক সার্ভিলেন্স

পরিচ্ছেদ 4ঃ খানায় রান্না করা তেল এবং লবণ ব্যবহার সম্পর্কিত তথ্য

নম্বর	সূচক	প্রশ্ন	উত্তর	কোড	নির্দেশনা
401	OS_401	আপনার বাড়িতে খাবার রান্নায় কোন ধরনের তেল ব্যবহার করেন?	1 = সয়াবিন তেল<br>2 = সরিষার তেল<br>3 = সুপার/ পাম অয়েল<br>4 = নারিকেল তেল<br>5 = সূর্যমুখী তেল<br>6 = ক্যানোলা তেল<br>7 = রাইস ব্র্যান্ড তেল<br>99 = অন্যান্য (নির্দিষ্ট করুন) _________		
402	OS_402	আপনার পরিবারে রান্নার কাজে ব্যবহার করার জন্য কি ধরনের তেল কিনে থাকেন?	1 = ব্র্যান্ড তেল<br>2 = খোলা তেল<br>3 = উভয়ই<br>99 = অন্যান্য (নির্দিষ্ট করুন)_________		উত্তর 2 হলে 406 এ যান
	OS_402A	আপনার পরিবারে রান্নার কাজে কোন ব্র্যান্ডের তেল কিনে থাকেন?	1 = তীর<br>2 = ফ্রেশ<br>3 = রূপচাঁদা<br>99 = অন্যান্য (নির্দিষ্ট করুন) ________		
403	OS_403	খানায় যে ব্র্যান্ড এর তেল ব্যবহার করা হয়/খাওয়া হয় তা কি ভিটামিন এ ফরটিফাইড বা ভিটামিন এ সমৃদ্ধ?	1 = হ্যাঁ 2 = না<br>77 = জানিনা		লেবেলিং দেখার জন্য খানার তেলের বোতল পর্যবেক্ষণ করুন
404	OS_404	খানায় যে ব্রেন্ড এর তেল ব্যবহার করা হয়/খাওয়া হয় তা কি ভিটামিন ডি ফরটিফাইড বা ভিটামিন ডি সমৃদ্ধ?	1 = হ্যাঁ 2 = না<br>77 = জানিনা		লেবেলিং দেখার জন্য খানার তেলের বোতল পর্যবেক্ষণ করুন
405	OS_405	খানায় যে ব্রেন্ড এর তেল ব্যবহার করা হয়/খাওয়া হয় তা কি ভিটামিন ই ফরটিফাইড বা ভিটামিন ই সমৃদ্ধ?	1 = হ্যাঁ 2 = না<br>77 = জানিনা		লেবেলিং দেখার জন্য খানার তেলের বোতল পর্যবেক্ষণ করুন
406	OS_406	খাবার তেল সংগ্রহ করা হয়েছে?	1 = হ্যাঁ 2 = না<br>77 = জানিনা		উত্তর 2 হলে 408 এ যান
407	OS_407	স্যাম্পল লেবেল/আইডি			
408	OS_408 A/B/C	আপনার খানায় কোন ধরণের লবণ ব্যবহার করেন?<br><br>(নিম্নের লবণের ধরণগুলো পড়ুন এবং নির্দিষ্ট ঘরে হ্যাঁ বা না কোড করুন)	খোলা লবণ: 1 = হ্যাঁ 2 = না<br>প্যাকেটে সংরক্ষিত লবণ (ব্র্যান্ড ছাড়া): 1 = হ্যাঁ 2 = না<br>প্যাকেটে সংরক্ষিত লবণ (ব্র্যান্ড সহ): 1 = হ্যাঁ 2 = না		যদি উত্তর খোলা লবণের কোড 'না' হয় তাহলে 410 এ যান
409	OS_409	আপনার খানায় সাধারণত কোন্ ব্র্যান্ড এর লবণ ব্যবহার করে থাকেন?	1 = মোল্লা সল্ট<br>2 = এ সি আই<br>3 = ব্র্যাক সল্ট<br>4 = তীর সল্ট<br>5 = ফ্রেশ সল্ট<br>6 = কনফিডেন্স<br>99 = অন্যান্য (নির্দিষ্ট করুন) _______		
410	OS_410	প্রতি সপ্তাহে খাবার এবং খাবার প্রস্তুতির জন্য কি পরিমান লবণ আপনার পরিবারে প্রয়োজন হয়?	|____|____|____|____| গ্রাম		
411	OS_411	আমরা যাচাই করে দেখতে চাই যে, আপনার খানায় যে লবণ ব্যবহৃত হয় তাতে আয়োডিন আছে কিনা। আমি কি ঐ লবণের কিছু নমুনা পেতে পারি যে লবণ আপনার খানার সদস্যদের খাবার তৈরীর জন্য গত রাতে ব্যবহার করা হয়েছিল?<br>*[লবণ পরীক্ষা করার পর যে ফলাফল আসবে তার সাপেক্ষে নম্বর বৃত্তায়িত করুন]*	1 = আয়োডিন নাই (0 পিপিএম)<br>2 = 15 পিপিএম এর কম<br>3 = 15 পিপিএম অথবা তার বেশী<br>4 = 25 পিপিএম এর কম<br>5 = 50 পিপিএম এর কম<br>6 = 75 পিপিএম এর কম<br>7 = 100 পিপিএম অথবা তার বেশী<br>8 = লবণ পরীক্ষা করা হয় নাই		

অংশগ্রহণকারীর আইডি:|___|___|___|___|___|___|___|___|___|___|___|___|___|___|___|___|

খাদ্য নিরাপত্তা ও পুষ্টি বিষয়ক সার্ভিলেন্স

মডিউল 3: শিশুর (0-5 বছর) প্রশ্নপত্র

পরিচ্ছেদ 1ঃ সাধারণ তথ্যাবলী

নম্বর	সূচক	প্রশ্ন	উত্তর	কোড	নির্দেশনা
101	CI_101	শিশুর নাম (ডাক নামসহ)	______________________________		
102	CI_102	আপনার শিশুর (শিশুর নাম বলুন) জন্ম তারিখ কত?	|____|____|/|____|____|/20|____|____| দিন মাস বছর		
103	CI_103	আপনার শিশুর বয়স (বয়স পূর্ণ বছরে লিখুন)	A. |____|____| বছর<br>B. |____|____| মাস		
104	CI_104	আপনার শিশুর লিঙ্গ	1 = ছেলে 2 = মেয়ে		
105	CI_105	আপনি (শিশুর মা) সর্বমোট কত বছর প্রাতিষ্ঠানিক শিক্ষা গ্রহণ করেছেন?<br>(সম্পূর্ণ বছর লিখুন। প্রথম শ্রেণীর নিচে এবং উপানুষ্ঠিক শিক্ষা অন্তর্ভুক্ত হবে না)<br>*নির্দেশনাঃ*<br>*তথ্য প্রদানকারীর সর্বমোট কত বছর প্রাতিষ্ঠানিক শিক্ষা সম্পন্ন করেছেন তা লিপিবদ্ধ। প্রাতিষ্ঠানিক শিক্ষা না থাকলে 00 লিখুন।*	|____|____| বছর<br>প্রাথমিক শিক্ষা/এবতেদায়ী = 5<br>মাধ্যমিক/দাখিল =10<br>উচ্চ মাধ্যমিক/ ডিপ্লোমা/আলিম= 12<br>স্নাতক/ফাজিল =16<br>স্নাতাকোত্তর/কামিল/দাওরা =18		
106	CI_106	গত 12 মাসে আপনার (শিশুর মায়ের) পেশা কি ছিল?<br>*নির্দেশনাঃ*<br>যদি তথ্য প্রদানকারী গত 12 মাসে একাধিক পেশায় নিযুক্ত থাকেন তাহলে তিনি যে পেশাটিতে বেশী সময় ব্যয় করেছেন সেটিকে প্রধান পেশা হিসাবে হিসাবে বিবেচনা করুন এবং তা লিপিবদ্ধ করুন।	1 = কৃষিকাজ (ধান)<br>2 = কৃষিকাজ (ধান ছাড়া অন্যান্য)<br>3 = কৃষি দিনমজুর<br>4 = অদক্ষ দিনমজুর<br>5 = দক্ষ দিনমজুর<br>6 = রিক্সা/ ভ্যান/ ঠেলাগাড়ী/ বেবীট্যাক্সি ড্রাইভার/ নৌকার মাঝি<br>7 = জেলে<br>8 = চাকুরীজীবি<br>9 = পেশাজীবি<br>10 = ব্যবসায়ী<br>11 = ক্ষুদে ব্যবসায়ী<br>12 = গৃহপরিচারিকা<br>13 = জুমচাষী<br>14 = উপার্জন করে না<br>15 = হাঁস/ মুরগী পালন/ পশু পালন<br>16 = হস্তশিল্প<br>17 = শাক-সজি চাষ<br>18 = মৎস চাষ<br>19 = গৃহিনী<br>20 = ছাত্র/ছাত্রী<br>77 = জানিনা<br>66 = বয়স 6 বছরের কম<br>99 = অন্যান্য (নির্দিষ্ট করুন) ___		
107	CI_107	শিশুর (শিশুর নাম) বাবা সর্বমোট কত বছর প্রাতিষ্ঠানিক শিক্ষা গ্রহণ করেছেন?<br>(সম্পূর্ণ বছর লিখুন। প্রথম শ্রেণীর নিচে এবং উপানুষ্ঠিক শিক্ষা অন্তর্ভুক্ত হবে না)<br>*নির্দেশনাঃ তথ্য প্রদানকারীর সর্বমোট কত বছর প্রাতিষ্ঠানিক শিক্ষা সম্পন্ন করেছেন তা লিপিবদ্ধ করুন। প্রাতিষ্ঠানিক শিক্ষা না থাকলে 00 লিখুন।*	|____|____| বছর<br>প্রাথমিক শিক্ষা/এবতেদায়ী = 5<br>মাধ্যমিক/দাখিল =10<br>উচ্চ মাধ্যমিক/ ডিপ্লোমা/আলিম= 12<br>স্নাতক/ফাজিল =16<br>স্নাতাকোত্তর/কামিল/দাওরা =18		
108	CI_108	গত 12 মাসে শিশুর বাবার (শিশুর নাম) পেশা কি ছিল?	1 = কৃষিকাজ (ধান)<br>2 = কৃষিকাজ (ধান ছাড়া অন্যান্য)<br>3 = কৃষি দিনমজুর<br>4 = অদক্ষ দিনমজুর<br>5 = দক্ষ দিনমজুর<br>6 = রিক্সা/ ভ্যান/ ঠেলাগাড়ী/ বেবীট্যাক্সি ড্রাইভার/ নৌকার মাঝি<br>7 = জেলে<br>8 = চাকুরীজীবি<br>9 = পেশাজীবি<br>10 = ব্যবসায়ী<br>11 = ক্ষুদে ব্যবসায়ী<br>12 = গৃহপরিচারিকা<br>13 = জুমচাষী<br>14 = উপার্জন করে না<br>15 = হাঁস/ মুরগী পালন/ পশু পালন<br>16 = হস্তশিল্প<br>17 = শাক-সজি চাষ<br>18 = মৎস চাষ<br>19 = গৃহিনী<br>20 = ছাত্র/ছাত্রী<br>77 = জানিনা<br>66 = বয়স 6 বছরের কম<br>99 = অন্যান্য (নির্দিষ্ট করুন)<br>__________		

অংশগ্রহণকারীর আইডি:|___|___|___|___|___|___|___|___|___|___|___|___|___|___|___|___|

খাদ্য নিরাপত্তা ও পুষ্টি বিষয়ক সার্ভিলেন্স

পরিচ্ছেদ 2ঃ নবজাতক এবং ছোট শিশুর খাদ্যাভাস সম্পর্কিত তথ্যাবলী

নম্বর	সূচক	প্রশ্ন	উত্তর	কোড	নির্দেশনা
201	IF_201	শিশুটি কোন বয়স শ্রেণীর অন্তর্ভুক্ত?	1 = 6 মাস বা তার কম<br>2 = 24 মাস বা তার কম<br>3 = 24 মাস বা তার বেশী		উত্তর 3 হলে পরিচ্ছেদ-3 নং এ যান
202	IF_202	আপনি কি কখনো (শিশুর নাম) কে বুকের দুধ পান করিয়েছেন?	1 = হ্যাঁ<br>2 = না		
203	IF_203	জন্মের কতক্ষণ পর (শিশুর নাম) কে বুকের দুধ দিয়েছিলেন?<br>*উত্তরদাতা যদি বলেন, জন্মের সাথে সাথেই শিশুকে বুকের দুধ দিয়েছেন, তবে "0" বৃত্তায়ন করুন এবং "00" কোড করুন ।*<br>*যদি 1ঘন্টার কম হয়, তবে "1 " বৃত্তায়ন করুন এবং ঘন্টার বক্সে "00" লিখুন ।*<br>*যদি 24 ঘন্টার কম হয়, তবে "1" বৃত্তায়ন করুন এবং 1 থেকে 23 পর্যন্ত বক্সে পূর্ণ ঘন্টায় লিখুন। যদি 24 ঘন্টার বেশী হয়, তবে "2" বৃত্তায়ন করুন এবং বক্সে পূর্ণ দিন লিখুন।*	0 =সাথে সাথেই |____|____| *অথবা*<br>1 = |____|____| ঘন্টা<br>*অথবা*<br>2 = |____|____| দিন<br>88 = প্রযোজ্য নয়		
204	IF_204	জন্মের পর শিশুকে শাল দুধ খাইয়েছিলেন?	1 = হ্যাঁ 2 = না<br>77 = জানিনা		
205	IF_205	আপনি (শিশুর নাম) কে বুকের প্রথম দুধ খাওয়ানোর পূর্বে মুখে অন্য কোনো কিছু দিয়েছিলেন?	1 = হ্যাঁ 2 = না<br>77 = জানিনা		
206	IF_206	আপনি (শিশুর নাম) কে জন্মের পরবর্তী 6 মাসের মধ্যে তার মুখে কোনো তরল/পানীয়/ খাবার দিয়েছিলেন?	1 = হ্যাঁ 2 = না<br>77 = জানিনা		উত্তর 2 হলে 208 এ যান

নম্বর	সূচক	প্রশ্ন		A. 1 = হ্যাঁ 2 = না	B. সংখ্যা (যদি হ্যাঁ হয়)	
207	IF_207A	(শিশুর নাম) কি গতকাল সারাদিন অথবা সারারাত নিম্নলিখিত তরল/পানীয় পান করেছিল এবং কতবার?	বুকের দুধ	1 = হ্যাঁ 2 = না	|____|____|	
	IF_207B		শুধু পানি	1 = হ্যাঁ 2 = না	|____|____|	
	IF_207C		চিনির পানি	1 = হ্যাঁ 2 = না	|____|____|	
	IF_207D		শিশু (Baby) ফর্মূলা (উদাহরণঃ সেরেলাক, ল্যাকটোজেন)	1 = হ্যাঁ 2 = না	|____|____|	
	IF_207E		টিনজাত দুধ, গুড়া দুধ, প্রাণীজ দুধ (গরু, ছাগল, ভেড়া, মহিষের দুধ)	1 = হ্যাঁ 2 = না	|____|____|	
	IF_207F		ফলের রস/ জুস ড্রিংকস/ডাবের পানি	1 = হ্যাঁ 2 = না	|____|____|	
	IF_207G		দই	1 = হ্যাঁ 2 = না	|____|____|	
	IF_207H		খাবার স্যালাইন	1 = হ্যাঁ 2 = না	|____|____|	
	IF_207I		পাতলা স্যুপ	1 = হ্যাঁ 2 = না	|____|____|	
	IF_207J		পাতলা সুজি	1 = হ্যাঁ 2 = না	|____|____|	
	IF_207K		ভিটামিন/ঔষধের ড্রপ/সিরাপ	1 = হ্যাঁ 2 = না	|____|____|	

অংশগ্রহণকারীর আইডি:|___|___|___|___|___|___|___|___|___|___|___|___|___|___|___|___|

খাদ্য নিরাপত্তা ও পুষ্টি বিষয়ক সার্ভিলেন্স

নম্বর	সূচক	প্রশ্ন	উত্তর	কোড	নির্দেশনা
208	IF_208	(শিশুর নাম) গতকাল সারাদিন অথবা সারারাত নিপলসহ বোতলে (ফিডার) করে কোনো কিছু পান করেছিল?	1 = হ্যাঁ 2 = না<br>77 = জানিনা		
209	IF_209	গত 1 মাসে শিশুকে বুকের দুধ অথবা সম্পূরক খাবার খাওয়ানো সম্পর্কে আপনি কি কোন বার্তা শুনেছেন/ দেখেছেন/ পড়েছেন?	1 = হ্যাঁ 2 = না<br>77 = জানিনা		
210	IF_210	যদি হ্যাঁ হয়, বার্তাটি কোথা থেকে শুনেছেন/ দেখেছেন/ পড়েছেন?	1 = রেডিও<br>2 = টেলিভিশন<br>3 = পত্রিকা<br>4 = মোবাইল ফোন<br>5 = ডাক্তার/ স্বাস্থ্য কর্মী/ সেবিকা আপা<br>6 = পোস্টার/ লিফলেট/ বিলবোর্ড<br>7 = প্রতিবেশী<br>99 = অন্যান্য (নির্দিষ্ট করুন) _______		
**কোন বয়সে আপনি শিশুকে নিচে উল্লেখিত তরল/খাবার দেয়া শুরু করেছিলেন? [জন্মের 1 (0-29 দিন) মাসের ভিতরে হলে 0 লিখুন)]**					
211	IF_211A	পানি	|____|____|		
	IF_211B	বুকের দুধ ব্যতিত তরল (চিনি, গ্লুকোজ পানি, চা, ফলের রস ইত্যাদি)	|____|____|		
	IF_211C	গরু/মহিষ/ছাগল এর দুধ	|____|____|		
	IF_211D	সুজি, চটকানো ভাত ইত্যাদি	|____|____|		
	IF_211E	আধা শক্ত খাবার (নরমভাত, খিচুড়ি, সিদ্ধ চটকানো আলু, পাকা কলা, খানার রান্না করা খাবার চটকানো ইত্যাদি)	|____|____|		
	IF_211F	শক্ত খাবার (ভাত, গম, মুড়ি/ চিড়া ইত্যাদি)	|____|____|		
	IF_211G	মাছ	|____|____|		
	IF_211H	মাংস (মুরগী, খাসি, গরু)	|____|____|		
	IF_211I	ডিম	|____|____|		
	IF_211J	ডাল জাতীয় (মসুর, মটরডাল, মটরসুটি ইত্যাদি)	|____|____|		
	IF_211K	সবুজ শাক-শব্জি	|____|____|		
	IF_211L	স্নাক্স (চানাচুর, চিপস, বিস্কুট, বাদাম ইত্যাদি)	|____|____|		
	IF_211M	মনিমিক্স, অনুপুষ্টি কণা	|____|____|		
	**কোডঃ** 0 = 30 দিনের ভিতরে (0 মাস বয়সে); 1 = 1 মাস পর; 2 = 2 মাস পর; 3 = 3 মাস পর; 4 = 4 মাস পর; 5 = 5 মাস পর; 6 = 6 মাস পর; 7 = 7 মাস পর; 8 = 8 মাস পর; 9 = 9 মাস পর; 10 = 10 মাস পর; 11 = 11 মাস পর; 12 = 12 মাস পর; 13 = এখনও দেয়া হয়নি; 77 = জানিনা				
212	IF_212	গতকাল সারাদিন অথবা সারারাত বাড়িতে বা বাড়ির বাইরে (শিশুর নাম) কে শক্ত, আধাশক্ত অথবা নরম খাবার (পানীয় ছাড়া) কতবার দিয়েছিলেন?	কতবার |____|____| 77 = জানিনা		
213	IF_213	(শিশুর নাম) কে গতকাল আয়রণ সিরাপ বা ট্যাবলেট/ড্রপ/স্প্রীংকল/মনিমিক্স খাওয়ানো হয়েছিল?	1 = হ্যাঁ 2 = না<br>77 = জানিনা		
214	IF_214	(শিশুর নাম) গত 7 দিনে পুষ্টি পাউডার মিশ্রিত কোনো খাবার (যেমন: স্প্রিংকল / মনিমিক্স) খেয়েছিল কি?	1 = হ্যাঁ 2 = না<br>77 = জানিনা		
215	IF_215	(শিশুর নাম) কে কখনও পুষ্টি পাউডার (স্প্রীংকল/ মনিমিক্স সহ) মিশ্রিত কোন খাবার খাওয়ানো হয়েছিল?	1 = হ্যাঁ 2 = না<br>77 = জানিনা		উত্তর 2 হলে পরিচ্ছেদ-3 নং এ যান
216	IF_216	কতদিন খাওয়ানো হয়েছিল? দিন উল্লেখ করুন	|____|____| দিন		201 এর উত্তর 1 হলে পরিচ্ছেদ-4 নং এ যান

অংশগ্রহণকারীর আইডি:|___|___|___|___|___|___|___|___|___|___|___|___|___|___|___|___|

পরিচ্ছেদ 3ঃ খাদ্য গ্রহণের বৈচিত্র্য

নম্বর	সূচক	প্রশ্ন		গতকাল দিনে বা রাতে আপনার শিশু নিম্নবর্ণিত খাবারগুলো খেয়েছেন কি? (A)	গত 7 দিনে আপনার শিশু নিম্নবর্ণিত খাবারগুলো কতদিন খেয়েছেন? (B)	গত 7 দিনে আপনার শিশু নিম্নবর্ণিত খাবারগুলো কতবার খেয়েছেন? (C)
এখন আমি আপনার শিশুকে গতকাল দিনে বা রাতে (গতকাল সকাল 6:00 টা থেকে আজ সকাল 6:00 টা পর্যন্ত) আপনি (শিশুর নাম) বাড়ীতে ও বাড়ীর বাইরে যা যা খাবার খাইয়েছেন তা বর্ণণা করতে অনুরোধ করছি। দয়া করে সব ধরনের খাবার, পানীয় যা আপনি (শিশুর নাম) সকাল, দুপুর ও রাতের খাবারের সাথে খাইয়েছেন বা নাস্তা/হালকা নাস্তা করিয়েছেন সে সম্পর্কে বলুন। আপনি (শিশুর নাম) জন্য খাবার তৈরীর সময় কোন খাবার খাইয়ে থাকলে তাও মনে করে আমাদেরকে বলুন। আপনি (শিশুর নাম) গতকাল সকালে যা খাইয়েছেন তা দিয়েই শুরু করুন।<br>- আপনি (শিশুর নাম) সকালে ঘুম থেকে উঠে কী কী খাইয়েছেন? আরো কিছু খাইয়েছেন কি?<br>- সকালে আরো কী কী খাইয়েছেন? আরো কিছু খাইয়েছেন কি?<br>- দুপুরে কী কী খাইয়েছেন? আরো কিছু খাইয়েছেন কি?<br>- বিকালে কী কী খাইয়েছেন? আরো কিছু খাইয়েছেন কি?<br>- সন্ধ্যায় কী কী খাইয়েছেন? আরো কিছু খাইয়েছেন কি?<br>- রাতে কী কী খাইয়েছেন? আরো কিছু খাইয়েছেন কি?<br>A কলামের প্রশ্নগুলোর উত্তর প্রথমে রেকর্ড করুন। A কলামের প্রশ্নগুলোর উত্তর রেকর্ড করা শেষ হলে B/ C কলামের উত্তর নিন।						
		খাবার ধরণ	খাবার নমুনা	(A)	(B)	(C)
301	CDD_301 A/B/C	শ্বেতসার জাতীয় (শস্য জাতীয় খাবার)	ভাত, আটা রুটি, গম, মুড়ি, ভুট্টা, খিচুড়ি, বার্লি, ওট, কিনোয়া, নুডলস, পান্তা	1 = হ্যাঁ<br>2 = না	|____|____| দিন	|____|____| বার
302	CDD_302 A/B/C	শ্বেতসার জাতীয় (মূল, কন্দ, কলা)	গোলআলু, মিষ্টিআলু, সাগু, এরারুট, কাঁচকলা, শালগম, কাসাভা, কচু, কচুমুখী, পাকাকলা, শালুক	1 = হ্যাঁ<br>2 = না	|____|____| দিন	|____|____| বার
303	CDD_303 A/B/C	ডাল ও ডাল জাতীয় খাবার	ডাল, শিমের বীচি, মটর, সয়বীন, টফু, হুমাস	1 = হ্যাঁ<br>2 = না	|____|____| দিন	|____|____| বার
304	CDD_304 A/B/C	বাদাম ও তৈলবীজ	চীনা বাদাম, পেস্তা, কাজু, অথবা যেকোন বাদাম, চিয়া সীড, তিল, তিসি, সূর্যমুখী বীজ, মিষ্টি কুমড়া বীজ	1 = হ্যাঁ<br>2 = না	|____|____| দিন	|____|____| বার
305	CDD_305 A/B/C	গাঢ় সবুজ পাতা জাতীয় শাক	সকল ধরনের পাতা জাতীয় শাক (পুঁই, কচু, কলমি), ব্রকলি	1 = হ্যাঁ<br>2 = না	|____|____| দিন	|____|____| বার
306	CDD_306 A/B/C	লাল/কমলা/ হলুদ সব্জি	মিষ্টিকুমড়া, গাজর, গাঢ় হলুদ বা কমলা মিষ্টি আলু, ও অন্যান্য লাল/ কমলা/হলুদ রঙের সব্জি	1 = হ্যাঁ<br>2 = না	|____|____| দিন	|____|____| বার
307	CDD_307 A/B/C	লাল/কমলা/ হলুদ ফলমূল	পাকা আম, পাকা পেঁপে ও অন্যান্য লাল/কমলা/ হলুদ ফলমূল	1 = হ্যাঁ<br>2 = না	|____|____| দিন	|____|____| বার
308	CDD_308 A/B/C	ভিটামিন সি-সমৃদ্ধ ফল	পেয়ারা, স্ট্রবেরী, লেবু, কমলালেবু, আঙ্গুর, আনারস, কাঁচা আম, আমলকি, কিউই, টমেটো	1 = হ্যাঁ<br>2 = না	|____|____| দিন	|____|____| বার
309	CDD_309 A/B/C	ভিটামিন সি-সমৃদ্ধ সব্জি	কাঁচা টমেটো, কাঁচা মরিচ, ব্রাসেলস স্প্রাউট, ফুলকপি, বাঁধাকপি	1 = হ্যাঁ<br>2 = না	|____|____| দিন	|____|____| বার
310	CDD_310 A/B/C	অন্যান্য শাক সব্জি	শিম, অ্যাসপারাগাস, বিট, কচি বাঁশ, ফুলকপি, সেলারি, শসা, বেগুন, লেটুস, মাশরুম, মূলা, জুকিনি	1 = হ্যাঁ<br>2 = না	|____|____| দিন	|____|____| বার
311	CDD_311 A/B/C	অন্যান্য ফলমূল	আপেল, আভাকাডো, জাম, চেরী, পাকা কাঁঠাল	1 = হ্যাঁ<br>2 = না	|____|____| দিন	|____|____| বার

নম্বর	সূচক	প্রশ্ন		গতকাল দিনে বা রাতে আপনার শিশু নিম্নবর্ণিত খাবারগুলো খেয়েছেন কি? (A)	গত 7 দিনে আপনার শিশু নিম্নবর্ণিত খাবারগুলো কতদিন খেয়েছেন? (B)	গত 7 দিনে আপনার শিশু নিম্নবর্ণিত খাবারগুলো কতবার খেয়েছেন? (C)
312	CDD_312 A/B/C	ডিম	হাঁস-মুরগী, অন্যান্য পাখী	1 = হ্যাঁ<br>2 = না	|____|____| দিন	|____|____| বার
313	CDD_313 A/B/C	অঙ্গ জাতীয় মাংস	গিলা, কলিজা, পাকস্থলী, হৃদপিণ্ড, কিডনি	1 = হ্যাঁ<br>2 = না	|____|____| দিন	|____|____| বার
314	CDD_314 A/B/C	মাংস	গরু, শুকর, বাছুর, মেষশাবক, ছাগল, মুরগী, হাঁস বা যেকোন প্রাণীর মাংস	1 = হ্যাঁ<br>2 = না	|____|____| দিন	|____|____| বার
315	CDD_315 A/B/C	ছোট মাছ	ছোট মাছের কাঁটা/হাড়সহ (কাঁচকি, মলা, ঢেলা, চাপিলা, বাতাসি, ছোট চিংড়ি, ছোট মাছের শুঁটকি)	1 = হ্যাঁ<br>2 = না	|____|____| দিন	|____|____| বার
316	CDD_316 A/B/C	বড় মাছ/সামুদ্রিক মাছ/ সামুদ্রিক খাবার	বড় মাছ, ঝিনুক, কাঁকড়া, অক্টোপাস, স্কুইড, হাঙর, বড় চিংড়ি, বড় মাছের শুঁটকি	1 = হ্যাঁ<br>2 = না	|____|____| দিন	|____|____| বার
317	CDD_317 A/B/C	দুধ ও দুগ্ধ জাতীয় খাবার	দুধ, পনির, দই এবং অন্যান্য দুগ্ধজাতীয় খাদ্য	1 = হ্যাঁ<br>2 = না	|____|____| দিন	|____|____| বার
318	CDD_318 A/B/C	পোকা ও অন্যান্য আমিষ জাতীয় খাবার	মাছের ডিম, পোকা, শামুক	1 = হ্যাঁ<br>2 = না	|____|____| দিন	|____|____| বার
319	CDD_319 A/B/C	তৈল জাতীয় খাবার	ঘি, মাখন, ক্রিম, সর, চর্বি, মার্জারিন, ম্যায়োনেজ, পাম অয়েল, উদ্ভিজ্জ তেল	1 = হ্যাঁ<br>2 = না	|____|____| দিন	|____|____| বার
320	CDD_320 A/B/C	চিনিযুক্ত এবং ভাজা খাবার	খাস্তা, চিপস ও অন্যান্য ভাজা খাবার, সিংগারা, সমোচা	1 = হ্যাঁ<br>2 = না	|____|____| দিন	|____|____| বার
321	CDD_321 A/B/C	মিষ্টি জাতীয় খাবার	চিনিযুক্ত খাবার, যেমন চকলেটস, ক্যান্ডিস, কুকিস/মিষ্টি বিস্কুট এবং কেকস, মিষ্টি পেষ্ট্রি বা আইসক্রিম, যে কোন মিষ্টি, মধু, হালুয়া, কনডেন্সড দুধ, তিলের খাজা	1 = হ্যাঁ<br>2 = না	|____|____| দিন	|____|____| বার
322	CDD_322 A/B/C	মিষ্টি পানীয়	চিনি যুক্ত চা, কোমল পানীয়, জুস, এনার্জি ড্রিংক, ইয়োগার্ট ড্রিংক, চকলেট ড্রিংক, হরলিকস, মলটোভা	1 = হ্যাঁ<br>2 = না	|____|____| দিন	|____|____| বার
323	CDD_323 A/B/C	অন্যান্য পানীয় এবং খাবার	চিনি ছাড়া চা, মদ, বিয়ার, আচার, সুপ, উপরের তালিকাভুক্ত নয় এমন যেকোন খাবার	1 = হ্যাঁ<br>2 = না	|____|____| দিন	|____|____| বার
324	CDD_324 A/B/C	অন্যান্য খাবার অথবা সুস্বাদু করার দ্রব্যাদি	মসলা, ধনে পাতা, সস, রসুন, কেচাপ, লেবুর রস, পুদিনা পাতা, পান, সুপারি, তামাক পাতা, জর্দা	1 = হ্যাঁ<br>2 = না	|____|____| দিন	|____|____| বার

অংশগ্রহণকারীর আইডি:|___|___|___|___|___|___|___|___|___|___|___|___|___|___|___|___|

খাদ্য নিরাপত্তা ও পুষ্টি বিষয়ক সার্ভিলেন্স

পরিচ্ছেদ 4ঃ শিশুর অসুস্থতা

আমি আপনাকে গত 2 সপ্তাহে আপনার শিশুর অসুস্থতা নিয়ে কিছু প্রশ্ন জিজ্ঞাসা করব (যদি বুধবার তথ্য সংগ্রহ করা হয়ে থাকে তাহলে বলুন গত বুধবারের আগের বুধবার থেকে গত কাল পর্যন্ত)

নম্বর	সূচক	প্রশ্ন	উত্তর	কোড	নির্দেশনা
401	CM_401	(শিশুর নাম) কি গত দুই সপ্তাহের/14 দিনের মধ্যে অসুস্থ হয়েছিল?	1 = হ্যাঁ 2 = না		উত্তর 2 হলে 407 এ যান
**(শিশুর নাম) এর কি গত দুই সপ্তাহের/14 দিনের মধ্যে নিম্নে উল্লেখিত কোন অসুখ হয়েছিল?**					
402	CM_402A	(শিশুর নাম বলুন) এর কি গত দুই সপ্তাহের মধ্যে কখনো জ্বর হয়েছে?	1 = হ্যাঁ 2 = না<br>77 = জানিনা		
	CM_402B	(শিশুর নাম বলুন) এর কি গত দুই সপ্তাহের মধ্যে কখনো সর্দি-কাশির রোগ (ব্যারাম) হয়েছে?	1 = হ্যাঁ 2 = না<br>77 = জানিনা		
	CM_402C	(শিশুর নাম বলুন) এর গত দুই সপ্তাহের মধ্যে স্বাভাবিকের চেয়ে দ্রুত শ্বাস, ঘন ঘন শ্বাস-প্রশ্বাস বা শ্বাস কষ্ট হয়েছিল কি?	1 = হ্যাঁ 2 = না<br>77 = জানিনা		
	CM_402D	(শিশুর নাম বলুন) এর গত দুই সপ্তাহের মধ্যে দিনে 3 বার বা তার বেশি পাতলা পায়খানা (ডায়রিয়া) হয়েছিল কি?<br>**অনুগ্রহ করে, ডায়রিয়ার আঞ্চলিক নাম ব্যবহার করুন।**	1 = হ্যাঁ 2 = না<br>77 = জানিনা		
403	CM_403	গত দুই সপ্তাহের/14 দিনের মধ্যে অসুস্থতার সময় কোথায়/কার কাছ থেকে পরামর্শ বা চিকিৎসা নিয়েছিলেন?	***সরকারী স্বাস্থ্যসেবা***<br>1 = সরকারী মাঠকর্মী<br>2 = সরকারী স্যাটেলাইট ক্লিনিক/অস্থায়ী টিকাদান (ইপিআই)কেন্দ্র<br>3 = সরকারী কমিউনিটি ক্লিনিক<br>4 = সরকারী স্বাস্থ্য ও পরিবার কল্যাণ কেন্দ্র (FWC)<br>5 = সরকারী উপজেলা স্বাস্থ্য কমপ্লেক্স<br>6 = শিশু ও মাতৃমঙ্গল কেন্দ্র<br>7 = অন্য সরকারী হাসপাতাল<br>***এনজিও স্বাস্থ্যসেবা***<br>8 = এনজিও স্বাস্থ্য ক্লিনিক ও হাসপাতাল<br>9 = এনজিও মাঠকর্মী<br>10 = কমিউনিটি নিউট্রিশন প্রোমোটার (সিএনপি)<br>***ব্যক্তিমালিকানাধীন স্বাস্থ্যসেবা***<br>11 = প্রাইভেট ডাক্তার/ক্লিনিক/ হাসপাতাল<br>12 = ফার্মেসী<br>13 = হাতুড়ে ডাক্তার/সনাতন<br>14 = হোমিওপ্যাথিক চিকিৎসক<br>15 = কারো কাছ থেকে নয়<br>99 = অন্যান্য (নির্দিষ্ট করুন)<br>___________		যদি গত 2 সপ্তাহের মধ্যে কোন অসুস্থতা না হয় তাহলে এই প্রশ্ন করার দরকার নাই
404	CM_404	(শিশুর নাম) কে অসুস্থ থাকাকালীন সময়ে কি পরিমাণ খাবার দেয়া হয়েছিল? তাকে স্বাভাবিকের চেয়ে কম/ প্রায় স্বাভাবিক পরিমাণ/ স্বাভাবিকের চেয়ে বেশী খাবার দেয়া হয়েছিল?	1 = স্বাভাবিকের চেয়ে কম<br>2 = স্বাভাবিক পরিমাণ<br>3 = স্বাভাবিকের চেয়ে বেশী<br>4 = কোন খাবার দেয়া হয় নি<br>5 = এখনো খাওয়ানো শুরু করেনি<br>77 = জানিনা		যদি গত 2 সপ্তাহের মধ্যে কোন অসুস্থতা না হয় তাহলে এই প্রশ্ন করার দরকার নাই

নম্বর	সূচক	প্রশ্ন	উত্তর			কোড	নির্দেশনা
405	CM_405	(শিশুর নাম) ডায়রিয়ায় অসুস্থ থাকাকালীন সময়ে কি পরিমাণ পানীয় দেয়া হয়েছিল? তাকে স্বাভাবিকের চেয়ে কম/ স্বাভাবিক পরিমাণ/স্বাভাবিকের চেয়ে বেশী পানীয় দেয়া হয়েছিল (বুকের দুধসহ)?	1 = স্বাভাবিকের চেয়ে কম<br>2 = স্বাভাবিক পরিমাণ<br>3 = স্বাভাবিকের চেয়ে বেশী<br>4 = কোন পানীয় দেয়া হয় নি<br>5 = এখনো খাওয়ানো শুরু করেনি<br>77 = জানিনা				যদি শিশুর ডায়রিয়া না হয় তাহলে এই প্রশ্ন করার দরকার নাই
406	CM_406	(শিশুর নাম) ডায়রিয়ায় অসুস্থ থাকাকালীন সময়ে (গত 14 দিন) তাকে কি উল্লেখিত তরল/ পানীয়গুলো দেয়া হয়েছিল?<br>*(যদি **ORS** খেয়ে থাকে, তবে জেনে নিন/দেখুন যে, **ORS** -এ জিংক ছিল কি না)*		হ্যাঁ	না		যদি শিশুর ডায়রিয়া না হয় তাহলে এই প্রশ্ন করার দরকার নাই
			A. প্যাকেট স্যালাইন (ORS)	1	2		
			B. বাড়ীতে তৈরী স্যালাইন (লবণ গুড়/ লবণ চিনির স্যালাইন)	1	2		
			C. চালের গুড়ের স্যালাইন (প্যাকেট)	1	2		
			D. চালের গুড়ের স্যালাইন (বাড়িতে তৈরী)	1	2		
			E. জিংক (Zinc) সিরাপ/ ট্যাবলেট/ORS	1	2		
407	CM_407	(শিশুর নাম) কি গত 6 মাসে ভিটামিন এ ক্যাপসুল (Vitamin 'A' Capsule) খেয়েছিল?	1 = হ্যাঁ, জাতীয় টিকা দিবস থেকে<br>2 = হ্যাঁ, অন্যান্য উৎস থেকে<br>3 = না<br>77 = জানি না<br>88 = প্রযোজ্য নয় (6 মাসের কম)				
408	CM_408	(শিশুর নাম) কি গত 6 মাসে এ্যালোপেথিক কৃমিনাশক ওষুধ খেয়েছিল?	1 = হ্যাঁ, জাতীয় টিকা দিবস/ভিটামিন **'A +'** ক্যা:<br>2 = হ্যাঁ, অন্যান্য উৎস থেকে<br>3 = না<br>77 = জানি না<br>88 = প্রযোজ্য নয় (12 মাসের কম)				
409	CM_409	গত 6 মাসের মধ্যে আপনার শিশুর (শিশুর নাম বলুন) কি জন্ডিস হয়েছিল?	1 = হ্যাঁ<br>2 = না<br>77 = জানি না				
410	CM_410	আপনার শিশু কি গত 6 মাসে মাঝারি তীব্র/মারাত্মক তীব্র অপুষ্ট শিশু হিসাবে কোন স্বাস্থ্য কর্মী বা চিকিৎসক দ্বারা চিহ্নিত হয়েছিল?	1 = হ্যাঁ (ছাড়পত্র/কার্ড আছে)<br>2= হ্যাঁ (ছাড়পত্র/কার্ড নাই)<br>3 = না<br>77 = জানি না<br>88 = প্রযোজ্য নয় (6 মাসের কম)				উত্তর না হলে পরবর্তী পরিচ্ছেদে এ যান
411	CM_411	আপনার শিশু কি মাঝারি তীব্র /মারাত্মক তীব্র অপুষ্টি জনিত কারণে চিকিৎসা নিয়েছিলেন	1 = হ্যাঁ<br>2 = না<br>77 = জানি না				
412	CM_412	উত্তর হ্যা হলে, কোথা থেকে/কার কাছ থেকে চিকিৎসা নিয়েছিলেন?	1 = *SAM* কর্ণার<br>2 = *MBBS* ডাক্তার<br>3 = *CHCP/কমিউনিটি ক্লিনিক*<br>99 = অন্যান্য (নির্দিষ্ট করুন) ______				
413	CM_413	চিকিৎসার ফলাফল কি ছিল?	1 = সুস্থ হয়েছিল<br>2 = সুস্থ হয়নি<br>3 = চিকিৎসা সমাপ্ত করেনি<br>99 = অন্যান্য (নির্দিষ্ট করুন) ______				

অংশগ্রহণকারীর আইডি:|___|___|___|___|___|___|___|___|___|___|___|___|___|___|___|___|

খাদ্য নিরাপত্তা ও পুষ্টি বিষয়ক সার্ভিলেন্স

পরিচ্ছেদ 5: শিশুর পরিমাপ

নম্বর	সূচক	প্রশ্ন	উত্তর	কোড	নির্দেশনা
501	CA_501	পরিমাপ নেয়া শুরুর সময়	|____|____|:|____|____| ঘন্টা : মিনিট		24 ঘণ্টার ফরম্যাট ব্যবহার করুন
502	CA_502	ওজন পরিমাপক যন্ত্রের আইডি	|____|____|		
503	CA_5403	দৈর্ঘ্য/উচ্চতা পরিমাপক যন্ত্রের আইডি	|____|____|		
504	CA_504	ওজন নেয়ার সময় গায়ের কাপড়ের ধরন	1 = হালকা কাপড়<br>2 = একটু ভারী কাপড়<br>3 = ভারী কাপড়		
505	CA_505	পরিমাপ গ্রহণকারীর নাম			
506	CA_506	পরিমাপ গ্রহণকারীর কোড	|____|____|		

নম্বর	সূচক	পরিমাপের নাম	পরিমাপ 1	পরিমাপ 2	পরিমাপ 3
507	CA_507 A/B/C	ওজন (Kg)	A.|____|____|.|____|____|	B. |____|____|.|____|____|	C. |____|____|.|____|____| যদি A ও B এর মধ্যে পার্থক্য 0.05Kg এর বেশি হয়, তাহলে ৩য় ওজন নিন
508	CA_508 A/B/C	বাম বাহুর পরিধি (Cm)	A.|____|____|.|____|	B.|____|____|.|____|	C. |____|____|.|____| যদি A ও B এর মধ্যে পার্থক্য 0.5cm এর বেশি হয়, তাহলে ৩য় বার মাপুন
509	CA_509 A/B/C	দৈর্ঘ্য/উচ্চতা (Cm)	A.|____|____|.|____|	B.|____|____|.|____|	C. |____|____|.|____| যদি A ও B এর মধ্যে পার্থক্য 0.5cm এর বেশি হয়, তাহলে ৩য় বার মাপুন

নম্বর	সূচক	প্রশ্ন	উত্তর	কোড	নির্দেশনা
510	CA_510	দৈর্ঘ্য/ উচ্চতা পরিমাপের পদ্ধতি	1 = দাঁড়ানো অবস্থায়<br>2 = শোয়ানো অবস্থায়		
511	CA_511	পায়ে পানি আসা	1 = হ্যাঁ 2 = না 77 = জানিনা		
512	CA_512	শিশুটি কি সহযোগিতা করেছিল?	1 = সহযোগী<br>2 = অসহযোগী<br>88 = প্রযোজ্য নয়		
513	CA_513	পরিমাপ সম্পর্কে তথ্য সংগ্রহকারীর মন্তব্য			
514	CA_514	পরিমাপ গ্রহণের শেষের সময়	|____|____| : |____|____| ঘন্টা : মিনিট		24 ঘণ্টার ফরম্যাট ব্যবহার করুন

পরিচ্ছেদ 6: রেফারেল (Referral)

নম্বর	সূচক	প্রশ্ন	উত্তর	কোড	নির্দেশনা
601	CA_601	শিশুকে রেফার করা হয়েছে কি?	1 = হ্যাঁ 2 = না		উত্তর না হলে, পরবর্তী পরিচ্ছেদে যান
602	CA_602 A/B/C /D/E/F	রেফারের কারণ	A.|____|____| B.|____|____| C.|____|____| D.|____|____| E.|____|____| F.|____|____|		একাধিক কারণ হতে পারে
রেফারের কারণ কোড:1 = Weight for height Z score <- 2; 2 = Weight for height Z score <- 3; 3 = MUAC 12.4-11.5 cm; 4 = MUAC <11.5 cm; 99 = অন্যান্য (নির্দিষ্ট করুন) __________					
603	CA_603	কোথায় রেফার করা হয়েছে	1 = উপজেলা স্বাস্থ্য কমপ্লেক্স<br>2 = জেলা হাসপাতাল<br>3 = মেডিকেল কলেজ হাসপাতাল<br>4 = ডাক্তারের চেম্বার<br>99 = অন্যান্য (নির্দিষ্ট করুন) __________		

অংশগ্রহণকারীর আইডি:|___|___|___|___|___|___|___|___|___|___|___|___|___|___|___|___|

খাদ্য নিরাপত্তা ও পুষ্টি বিষয়ক সার্ভিলেন্স

মডিউল 4: কিশোর বালকদের প্রশ্নপত্র

পরিচ্ছেদ 1ঃ সাধারণ তথ্যাবলী

নম্বর	সূচক	প্রশ্ন	উত্তর	কোড	নির্দেশনা
101	AB_101	আপনার নাম (ডাক নামসহ)	____________________________		
102	AB_102	আপনার জন্ম তারিখ কত?	|____|____|/|____|____|/20|____|____| দিন মাস বছর		
103	AB_103	আপনার বয়স (বয়স পূর্ণ বছরে লিখুন)	|____|____| বছর		
104	AB_104	আপনার বৈবাহিক অবস্থা?	1 = কখনই বিবাহ করিনি<br>2 = বর্তমানে বিবাহিত<br>3 = পৃথক<br>4 = তালাকপ্রাপ্ত<br>5 = বিপত্নিক/বিধবা<br>88 = জানাতে অসম্মতি		
105	AB_105	আপনি (কিশোরের নাম) সর্বমোট কত বছর প্রাতিষ্ঠানিক শিক্ষা গ্রহণ করেছেন?<br><br>(প্রথম শ্রেণীর নিচে এবং উপানুষ্ঠিক শিক্ষা অন্তর্ভুক্ত হবে না)<br>***নির্দেশনাঃ***<br>*তথ্য প্রদানকারীর সর্বমোট কত বছর প্রাতিষ্ঠানিক শিক্ষা সম্পন্ন করেছেন তা লিপিবদ্ধ করুন।*<br>*প্রাতিষ্ঠানিক শিক্ষা না থাকলে 00 লিখুন।*	|____|____| বছর<br><br>প্রাথমিক শিক্ষা/এবতেদায়ী = 5<br>মাধ্যমিক/দাখিল =10<br>উচ্চ মাধ্যমিক/ ডিপ্লোমা/আলিম= 12<br>স্নাতক/ফাজিল =16<br>স্নাতাকোত্তর/কামিল/দাওরা =18		
106	AB_106	গত 12 মাসে আপনার (কিশোরের নাম) পেশা কি ছিল?<br><br>যদি তথ্য প্রদানকারী গত 12 মাসে একাধিক পেশায় নিযুক্ত থাকেন তাহলে তিনি যে পেশাটিতে বেশী সময় ব্যয় করেছেন সেটিকে প্রধান পেশা হিসাবে বিবেচনা করুন এবং তা লিপিবদ্ধ করুন।	1 = কৃষিকাজ (ধান)<br>2 = কৃষিকাজ (ধান ছাড়া অন্যান্য)<br>3 = কৃষি দিনমজুর<br>4 = অদক্ষ দিনমজুর<br>5 = দক্ষ দিনমজুর<br>6 = রিক্সা/ ভ্যান/ ঠেলাগাড়ী/ বেবীট্যাক্সি ড্রাইভার/ নৌকার মাঝি<br>7 = জেলে<br>8 = চাকুরীজীবি<br>9 = পেশাজীবি<br>10 = ব্যবসায়ী<br>11 = ক্ষুদে ব্যবসায়ী<br>12 = গৃহপরিচারিকা<br>13 = জুমচাষী<br>14 = উপার্জন করে না<br>15 = হাঁস/ মুরগী পালন/ পশু পালন<br>16 = হস্তশিল্প<br>17 = শাক-সব্জি চাষ<br>18 = মৎস চাষ<br>19 = গৃহিনী<br>20 = ছাত্র/ছাত্রী<br>77 = জানিনা<br>66 = বয়স 6 বছরের কম<br>99 = অন্যান্য (নির্দিষ্ট করুন) __		
107	AB_107	কিশোরের (কিশোরের নাম) মা সর্বমোট কত বছর প্রাতিষ্ঠানিক শিক্ষা গ্রহণ করেছেন?<br><br>(প্রথম শ্রেণীর নিচে এবং উপানুষ্ঠিক শিক্ষা অন্তর্ভুক্ত হবে না)<br>***নির্দেশনাঃ*** *তথ্য প্রদানকারীর সর্বমোট কত বছর প্রাতিষ্ঠানিক শিক্ষা সম্পন্ন করেছেন তা লিপিবদ্ধ করুন। প্রাতিষ্ঠানিক শিক্ষা না থাকলে 00 লিখুন।*	|____|____| বছর<br>প্রাথমিক শিক্ষা/এবতেদায়ী = 5<br>মাধ্যমিক/দাখিল =10<br>উচ্চ মাধ্যমিক/ ডিপ্লোমা/আলিম= 12<br>স্নাতক/ফাজিল =16<br>স্নাতাকোত্তর/কামিল/দাওরা =18		

নম্বর	সূচক	প্রশ্ন	উত্তর	কোড	নির্দেশনা
108	AB_108	গত 12 মাসে কিশোরের মায়ের (কিশোরের নাম) পেশা কি ছিল?<br><br>***নির্দেশনাঃ***<br>যদি তথ্য প্রদানকারী গত 12 মাসে একাধিক পেশায় নিযুক্ত থাকেন তাহলে তিনি যে পেশাটিতে বেশী সময় ব্যয় করেছেন সেটিকে প্রধান পেশা হিসাবে বিবেচনা করুন এবং তা লিপিবদ্ধ করুন।	1 = কৃষিকাজ (ধান)<br>2 = কৃষিকাজ (ধান ছাড়া অন্যান্য)<br>3 = কৃষি দিনমজুর<br>4 = অদক্ষ দিনমজুর<br>5 = দক্ষ দিনমজুর<br>6 = রিক্সা/ ভ্যান/ ঠেলাগাড়ী/ বেবীট্যাক্সি ড্রাইভার/ নৌকার মাঝি<br>7 = জেলে<br>8 = চাকুরীজীবি<br>9 = পেশাজীবি<br>10 = ব্যবসায়ী<br>11 = ক্ষুদে ব্যবসায়ী<br>12 = গৃহপরিচারিকা<br>13 = জুমচাষী<br>14 = উপার্জন করে না<br>15 = হাঁস/ মুরগী পালন/ পশু পালন<br>16 = হস্তশিল্প<br>17 = শাক-সব্জি চাষ<br>18 = মৎস চাষ<br>19 = গৃহিনী<br>20 = ছাত্র/ছাত্রী<br>77 = জানিনা<br>66 = বয়স 6 বছরের কম<br>99 = অন্যান্য (নির্দিষ্ট করুন) __		
109	AB_109	কিশোরের (কিশোরের নাম) বাবা সর্বমোট কত বছর প্রাতিষ্ঠানিক শিক্ষা গ্রহণ করেছেন?<br><br>(প্রথম শ্রেণীর নিচে এবং উপানুষ্ঠিক শিক্ষা অন্তর্ভুক্ত হবে না)<br>***নির্দেশনাঃ***<br>*সর্বমোট কত বছর প্রাতিষ্ঠানিক শিক্ষা সম্পন্ন করেছেন তা লিপিবদ্ধ করুন। প্রাতিষ্ঠানিক শিক্ষা না থাকলে 00 লিখুন।*	|____|____| বছর<br>প্রাথমিক শিক্ষা/এবতেদায়ী = 5<br>মাধ্যমিক/দাখিল =10<br>উচ্চ মাধ্যমিক/ ডিপ্লোমা/আলিম= 12<br>স্নাতক/ফাজিল =16<br>স্নাতাকোত্তর/কামিল/দাওরা =18		
110	AB_110	গত 12 মাসে কিশোরের বাবার (কিশোরের নাম) পেশা কি ছিল?<br><br>***নির্দেশনাঃ***<br>যদি তথ্য প্রদানকারী গত 12 মাসে একাধিক পেশায় নিযুক্ত থাকেন তাহলে তিনি যে পেশাটিতে বেশী সময় ব্যয় করেছেন সেটিকে প্রধান পেশা হিসাবে বিবেচনা করুন এবং তা লিপিবদ্ধ করুন।	1 = কৃষিকাজ (ধান)<br>2 = কৃষিকাজ (ধান ছাড়া অন্যান্য)<br>3 = কৃষি দিনমজুর<br>4 = অদক্ষ দিনমজুর<br>5 = দক্ষ দিনমজুর<br>6 = রিক্সা/ ভ্যান/ ঠেলাগাড়ী/ বেবীট্যাক্সি ড্রাইভার/ নৌকার মাঝি<br>7 = জেলে<br>8 = চাকুরীজীবি<br>9 = পেশাজীবি<br>10 = ব্যবসায়ী<br>11 = ক্ষুদে ব্যবসায়ী<br>12 = গৃহপরিচারিকা<br>13 = জুমচাষী<br>14 = উপার্জন করে না<br>15 = হাঁস/ মুরগী পালন/ পশু পালন<br>16 = হস্তশিল্প<br>17 = শাক-সব্জি চাষ<br>18 = মৎস চাষ<br>19 = গৃহিনী<br>20 = ছাত্র/ছাত্রী<br>77 = জানিনা<br>66 = বয়স 6 বছরের কম<br>99 = অন্যান্য (নির্দিষ্ট করুন) __		

অংশগ্রহণকারীর আইডি:|___|___|___|___|___|___|___|___|___|___|___|___|___|___|___|___|

পরিচ্ছেদ 2ঃ খাদ্য গ্রহণের বৈচিত্র্য

নম্বর	সূচক	প্রশ্ন		গতকাল দিনে বা রাতে আপনি নিম্নবর্ণিত খাবারগুলো খেয়েছেন কি? (A)	গত 7 দিনে আপনি নিম্নবর্ণিত খাবারগুলো কতদিন খেয়েছেন? (B)	গত 7 দিনে আপনি নিম্নবর্ণিত খাবারগুলো কতবার খেয়েছেন? (C)
এখন আমি আপনাকে গতকাল দিনে বা রাতে (গতকাল সকাল 6:00 টা থেকে আজ সকাল 6:00 টা পর্যন্ত) আপনি বাড়ীতে ও বাড়ীর বাইরে যা যা খেয়েছেন তা বর্ণণা করতে অনুরোধ করছি। দয়া করে সব ধরনের খাবার, পানীয় যা আপনি সকাল, দুপুর ও রাতের খাবারের সাথে খেয়েছেন বা নাস্তা/হালকা নাস্তা করেছেন সে সম্পর্কে বলুন। আপনি খাবার তৈরীর সময় কোন খাবার খেয়ে থাকলে তাও মনে করে আমাদেরকে বলুন। আপনি গতকাল সকালে যা খেয়েছেন তা দিয়েই শুরু করুন।<br>- আপনি সকালে ঘুম থেকে উঠে কী কী খেয়েছেন? আরো কিছু খেয়েছেন কি?<br>- সকালে আরো কী কী খেয়েছেন? আরো কিছু খেয়েছেন কি?<br>- দুপুরে কী কী খেয়েছেন? আরো কিছু খেয়েছেন কি?<br>- বিকালে কী কী খেয়েছেন? আরো কিছু খেয়েছেন কি?<br>- সন্ধ্যায় কী কী খেয়েছেন? আরো কিছু খেয়েছেন কি?<br>- রাতে কী কী খেয়েছেন? আরো কিছু খেয়েছেন কি?<br>A কলামের প্রশ্নগুলোর উত্তর প্রথমে রেকর্ড করুন। A কলামের প্রশ্নগুলোর উত্তর রেকর্ড করা শেষ হলে B/ C কলামের উত্তর নিন।						
		খাবার ধরণ	খাবার নমুনা			
201	ABD_201 A/B/C	শ্বেতসার জাতীয় (শস্য জাতীয় খাবার)	ভাত, আটা রুটি, গম, মুড়ি, ভুট্টা, খিচুড়ি, বার্লি, ওট, কিনোয়া, নুডলস, পান্তা	1 = হ্যাঁ<br>2 = না	|____|____| দিন	|____|____| বার
202	ABD_202 A/B/C	শ্বেতসার জাতীয় (মূল, কন্দ, কলা)	গোলআলু, মিষ্টিআলু, সাগু, এরারুট, কাঁচকলা, শালগম, কাসাভা, কচু, কচুমুখী, পাকাকলা, শালুক	1 = হ্যাঁ<br>2 = না	|____|____| দিন	|____|____| বার
203	ABD_203 A/B/C	ডাল ও ডাল জাতীয় খাবার	ডাল, শিমের বীচি, মটর, সয়বীন, টফু, হুমাস	1 = হ্যাঁ<br>2 = না	|____|____| দিন	|____|____| বার
204	ABD_204 A/B/C	বাদাম ও তৈলবীজ	চীনা বাদাম, পেস্তা, কাজু, অথবা যেকোন বাদাম, চিয়া সীড, তিল, তিসি, সূর্যমুখী বীজ, মিষ্টি কুমড়া বীজ	1 = হ্যাঁ<br>2 = না	|____|____| দিন	|____|____| বার
205	ABD_205 A/B/C	গাঢ় সবুজ পাতা জাতীয় শাক	সকল ধরনের পাতা জাতীয় শাক (পুঁই, কচু, কলমি), ব্রকলি	1 = হ্যাঁ<br>2 = না	|____|____| দিন	|____|____| বার
206	ABD_206 A/B/C	লাল/কমলা/ হলুদ সব্জি	মিষ্টিকুমড়া, গাজর, গাঢ় হলুদ বা কমলা মিষ্টি আলু, ও অন্যান্য লাল/ কমলা/হলুদ রঙের সব্জি	1 = হ্যাঁ<br>2 = না	|____|____| দিন	|____|____| বার
207	ABD_207 A/B/C	লাল/কমলা/ হলুদ ফলমূল	পাকা আম, পাকা পেঁপে ও অন্যান্য লাল/কমলা/ হলুদ ফলমূল	1 = হ্যাঁ<br>2 = না	|____|____| দিন	|____|____| বার
208	ABD_208 A/B/C	ভিটামিন সি- সমৃদ্ধ ফল	পেয়ারা, স্ট্রবেরী, লেবু, কমলালেবু, আঙ্গুর, আনারস, কাঁচা আম, আমলকি, কিউই, টমেটো	1 = হ্যাঁ<br>2 = না	|____|____| দিন	|____|____| বার
209	ABD_209 A/B/C	ভিটামিন সি- সমৃদ্ধ সব্জি	কাঁচা টমেটো, কাঁচা মরিচ, ব্রাসেলস স্প্রাউট, ফুলকপি, বাঁধাকপি	1 = হ্যাঁ<br>2 = না	|____|____| দিন	|____|____| বার

অংশগ্রহণকারীর আইডি:|___|___|___|___|___|___|___|___|___|___|___|___|___|___|___|___|

খাদ্য নিরাপত্তা ও পুষ্টি বিষয়ক সার্ভিলেন্স

নম্বর	সূচক	প্রশ্ন		গতকাল দিনে বা রাতে আপনি নিম্নবর্ণিত খাবারগুলো খেয়েছেন কি? (A)	গত 7 দিনে আপনি নিম্নবর্ণিত খাবারগুলো কতদিন খেয়েছেন? (B)	গত 7 দিনে আপনি নিম্নবর্ণিত খাবারগুলো কতবার খেয়েছেন? (C)
210	ABD_210 A/B/C	অন্যান্য শাক সব্জি	শিম, অ্যাসপারাগাস, বিট, কচি বাঁশ, ফুলকপি, সেলারি, শসা, বেগুন, লেটুস, মাশরুম, মূলা, জুকিনি	1 = হ্যাঁ<br>2 = না	|____|____| দিন	|____|____| বার
211	ABD_211 A/B/C	অন্যান্য ফলমূল	আপেল, আভাকাডো, জাম, চেরী, পাকা কাঁঠাল	1 = হ্যাঁ<br>2 = না	|____|____| দিন	|____|____| বার
212	ABD_212 A/B/C	ডিম	হাঁস-মুরগী, অন্যান্য পাখী	1 = হ্যাঁ<br>2 = না	|____|____| দিন	|____|____| বার
213	ABD_213 A/B/C	অঙ্গ জাতীয় মাংস	গিলা, কলিজা, পাকস্থলী, হৃদপিণ্ড, কিডনি	1 = হ্যাঁ<br>2 = না	|____|____| দিন	|____|____| বার
214	ABD_214 A/B/C	মাংস	গরু, শুকর, বাছুর, মেষশাবক, ছাগল, মুরগী, হাঁস বা যেকোন প্রাণীর মাংস	1 = হ্যাঁ<br>2 = না	|____|____| দিন	|____|____| বার
215	ABD_215 A/B/C	ছোট মাছ	ছোট মাছের কাঁটা/হাড়সহ (কাঁচকি, মলা, ঢেলা, চাপিলা, বাতাসি, ছোট চিংড়ি, ছোট মাছের শুঁটকি)	1 = হ্যাঁ<br>2 = না	|____|____| দিন	|____|____| বার
216	ABD_216 A/B/C	বড় মাছ/সামুদ্রিক মাছ/ সামুদ্রিক খাবার	বড় মাছ, ঝিনুক, কাঁকড়া, অক্টোপাস, স্কুইড, হাঙর, বড় চিংড়ি, বড় মাছের শুঁটকি	1 = হ্যাঁ<br>2 = না	|____|____| দিন	|____|____| বার
217	ABD_217 A/B/C	দুধ ও দুগ্ধ জাতীয় খাবার	দুধ, পনির, দই এবং অন্যান্য দুগ্ধজাতীয় খাদ্য	1 = হ্যাঁ<br>2 = না	|____|____| দিন	|____|____| বার
218	ABD_218 A/B/C	পোকা ও অন্যান্য আমিষ জাতীয় খাবার	মাছের ডিম, পোকা, শামুক	1 = হ্যাঁ<br>2 = না	|____|____| দিন	|____|____| বার
219	ABD_219 A/B/C	তৈল জাতীয় খাবার	ঘি, মাখন, ক্রিম, সর, চর্বি, মার্জারিন, ম্যায়োনেজ, পাম অয়েল, উদ্ভিজ্জ তেল	1 = হ্যাঁ<br>2 = না	|____|____| দিন	|____|____| বার
220	ABD_220 A/B/C	চিনিযুক্ত এবং ভাজা খাবার	খাস্তা, চিপস ও অন্যান্য ভাজা খাবার, সিংগারা, সমোচা	1 = হ্যাঁ<br>2 = না	|____|____| দিন	|____|____| বার
221	ABD_221 A/B/C	মিষ্টি জাতীয় খাবার	চিনিযুক্ত খাবার, যেমন চকলেটস, ক্যান্ডিস, কুকিস/মিষ্টি বিস্কুট এবং কেকস, মিষ্টি পেষ্ট্রি বা আইসক্রিম, যে কোন মিষ্টি, মধু, হালুয়া, কনডেন্সড দুধ, তিলের খাজা	1 = হ্যাঁ<br>2 = না	|____|____| দিন	|____|____| বার
222	ABD_222 A/B/C	মিষ্টি পানীয়	চিনি যুক্ত চা, কোমল পানীয়, জুস, এনার্জি ড্রিংক, ইয়োগার্ট ড্রিংক, চকলেট ড্রিংক, হরলিকস, মলটোভা	1 = হ্যাঁ<br>2 = না	|____|____| দিন	|____|____| বার
223	ABD_223 A/B/C	অন্যান্য পানীয় এবং খাবার	চিনি ছাড়া চা, মদ, বিয়ার, আচার, সুপ, উপরের তালিকাভুক্ত নয় এমন যেকোন খাবার	1 = হ্যাঁ<br>2 = না	|____|____| দিন	|____|____| বার
224	ABD_224 A/B/C	অন্যান্য খাবার সুস্বাদু করার দ্রব্যাদি	মসলা, ধনে পাতা, সস, রসুন, কেচাপ, লেবুর রস, পুদিনা পাতা, পান, সুপারি, তামাক পাতা, জর্দা	1 = হ্যাঁ<br>2 = না	|____|____| দিন	|____|____| বার

অংশগ্রহণকারীর আইডি:|___|___|___|___|___|___|___|___|___|___|___|___|___|___|___|___|

নম্বর	সূচক	প্রশ্ন	উত্তর	কোড	নির্দেশনা
আমি আপনাকে পরবর্তীতে যে প্রশ্নগুলো জিজ্ঞাসা করতে যাচ্ছি তা হল সচরাচর আপনি যে সকল ফলমূল ও শাক-সজি খেয়ে থাকেন সে বিষয়ে। আমার কাছে ফল ও শাক-সজির কিছু ছবি আছে। প্রতিটি ছবি এক একটি প্রমান মাপের সমান। উত্তর দেওয়ার সময় সাধারন 1টি সপ্তাহের কথা চিন্তা করুন। (মাসে 1-2 বার হলে উত্তর 00 হবে)					
225	ABD_225	সচরাচর সপ্তাহের কত দিন আপনি ফল খান ? (নমুনা কার্ড দেখান)<br>*নির্দেশনাঃ*<br>*তথ্য প্রদানকারীকে নমুনা কার্ডে প্রদর্শিত ফলগুলো দেখিয়ে চিন্তা করতে বলুন। এখানে প্যাকেটজাত ফলের জুস গ্রহণযোগ্য নয় তবে বাসায় ব্লেন্ড করা ফলের জুস গ্রহণযোগ্য। সপ্তাহ বলতে ধর্মীয় বা অন্য কোন বিশেষ উপলক্ষ্য ব্যতীত একটি স্বাভাবিক সপ্তাহ বুঝায়। মাসে 1-2 বার হলে 00 লিখুন।*	দিনের সংখ্যা |___|___| 77 = জানা নাই<br>(যদি 00 দিন হয়, তাহলে ABD_227-তে যান)		
226	ABD_226	সেই দিন গুলির একদিনে কতটুকু ফল খেয়েছেন? (নমুনা কার্ড ও বাটি দেখান)<br>*নির্দেশনাঃ*<br>*তথ্য প্রদানকারীকে যে কোন একদিনের কথা স্মরণ করতে বলুন এবং বাটি দেখিয়ে পরিমাপ করতে বলুন।*	সারভিং সংখ্যা<br>|___|___|.|___| 77.7 = জানা নাই		
227	ABD_227	সচরাচর সপ্তাহের কত দিন আপনি শাক-সজি খান? (নমুনা কার্ড দেখান)<br>*নির্দেশনাঃ*<br>*তথ্য প্রদানকারীকে নমুনা কার্ডে প্রদর্শিত শাক-সজিগুলো দেখিয়ে চিন্তা করতে বলুন। এখানে আলু শাক-সজি হিসাবে গণ্য হবে না। কাঁচা ও রান্না করা শাক-সজির প্রমান পরিমাপ আলাদা করে দেখান। মাছের সাথে রান্না করা সজির ক্ষেত্রে শুধু সজির পরিমান করতে হবে। সপ্তাহ বলতে ধর্মীয় বা অন্য কোন বিশেষ উপলক্ষ্য ব্যতীত একটি স্বাভাবিক সপ্তাহ বুঝায়। মাসে 1-2 বার হলে '00' হবে।*	দিনের সংখ্যা |___|___| 77 = জানা নাই (যদি '00' দিন হয়, তাহলে ABD_229-তে যান)		
228	ABD_228	সেই দিন গুলির একদিনে কতটুকু পরিমান শাক-সজি খেয়েছেন? (নমুনা কার্ড ও বাটি দেখান)<br>*নির্দেশনাঃ*<br>*তথ্য প্রদানকারীকে যে কোন একদিনের কথা স্মরণ করতে বলুন এবং বাটি দেখিয়ে পরিমাপ করতে বলুন।*	সারভিং সংখ্যা<br>|___|___|.|___| 77.7 = জানা নাই		
229	ABD_229	আপনি কি বর্তমানে কোন ধরণের ভিটামিন/খনিজ লবণ আছে এমন কোন ট্যাবলেট, ক্যাপসুল, সিরাপ খান?	1 = হ্যাঁ<br>2 = না		যদি 'না' হয় তাহলে পরবর্তী পরিচ্ছেদে যান
230	ABD_230	ব্যবস্থাপত্র দেখে বা ঔষধ দেখে নাম লিখুন	A.________________<br>B.________________<br>C.________________		

অংশগ্রহণকারীর আইডি:|___|___|___|___|___|___|___|___|___|___|___|___|___|___|___|___|

খাদ্য নিরাপত্তা ও পুষ্টি বিষয়ক সার্ভিলেন্স

পরিচ্ছেদ 3ঃ আচরণগত ঝুঁকির কারণ (ধুমপান/ধোঁয়াহীন তামাক এবং শারীরিক পরিশ্রম)

নম্বর	সূচক	প্রশ্ন	উত্তর	কোড	নির্দেশনা
**মূলঃ তামাকের ব্যবহার**					
এখন আমি আপনাকে তামাক ও তামাকজাত দ্রব্যের (যেমনঃ ধূমপান, ধোঁয়াবিহীন তামাকের) ব্যবহার সম্পর্কে কিছু প্রশ্ন জিজ্ঞাসা করব।					
301	ABR_301	আপনি কি বর্তমানে কোন প্রকার ধূমপান করেন?<br>(যেমন: সিগারেট, বিড়ি, হুক্কা, চুরুট, সিগার)<br>[নমুনা কার্ড দেখান]<br>*নির্দেশনাঃ*<br>*উত্তরদাতাকে নমুনা কার্ড দেখিয়ে চিন্তা করতে বলুন যে বর্তমানে তিনি কোন দ্রব্যটি ধূমপান/ ব্যবহার করছেন।*	1 = হ্যাঁ<br>2 = না<br>(যদি না হয়, তাহলে ABR_304 এ যান)		
302	ABR_302	আপনি কি বর্তমানে প্রতিদিন ধূমপান করেন?<br><br>*নির্দেশনাঃ*<br>*এই প্রশ্নটি যারা বর্তমানে ধূমপান করেন তাদের জন্য প্রযোজ্য।*<br>***প্রতিদিন অর্থঃ***<br>*প্রায় একমাস বা তার বেশী সময় ধরে প্রতিদিন অন্তত একবার ধূমপান করা কে বুঝায়। যদি এমন হয় যে তথ্য প্রদানকারী 25 দিন হল ধূমপান শুরু করেছে এবং এখনো চলছে, সেক্ষেত্রে প্রতিদিন হিসাবে গণ্য হবে।*	1 = হ্যাঁ<br>2 = না		
303	ABR_303	কত বছর বয়সে আপনি প্রথম ধূমপান শুরু করেন?	বয়স |___|___| 77 = জানা নাই		
304	ABR_304	আপনি কি বর্তমানে কোন প্রকার ধোঁয়াবিহীন তামাক দ্রব্য ব্যবহার করেন?<br>(যেমনঃ পানের সাথে জর্দা, শুধু জর্দা, সুপারির সাথে জর্দা, পানের সাথে সাদাপাতা, তামাকযুক্ত পানমশলা, চিবিয়ে খাওয়া সাদাপাতা, খৈনি, নস্যি, গুল, ইত্যাদি)<br>[নমুনা কার্ড দেখান]<br><br>*নির্দেশনাঃ*<br>*তথ্য প্রদানকারীকে ধোঁয়বিহীন তামাক যেমনঃ জর্দা, গুল, সাদাপাতা, খৈনি, নস্যি দ্রব্যগুলো কি বর্তমানে ব্যবহার করেন কিনা তা চিন্তা করে উত্তর দিতে বলুন। এক্ষেত্রে, শুধু পান সুপারী ও চুন প্রযোজ্য হবে না। যদি তথ্য প্রদানকারী পানের সাথে জর্দা বা শুধু জর্দা, পানের সাথে সাদাপাতা বা শুধু সাদাপাতা, পানের সাথে তামাক যুক্ত পান মশলা বা শুধু তামাক যুক্ত পান মশলা খান তাহলে ধোঁয়াবিহীন তামাক সেবন হিসাবে গণ্য হবে।*	1 = হ্যাঁ<br>2 = না<br>(যদি না হয়, তাহলে ABR_307 এ যান)		
305	ABR_305	আপনি কি বর্তমানে প্রতিদিন এই ধোঁয়াবিহীন তামাক দ্রব্য ব্যবহার করেন?<br>**প্রতিদিন অর্থঃ** প্রায় একমাস বা তার বেশী সময় ধরে প্রতিদিন অন্তঃত একটি ধোঁয়াবিহীন তামাক পণ্য ব্যবহার করা কে বুঝায়। *যদি এমন হয় যে তথ্য প্রদানকারী 25 দিন হল ধূমপান শুরু করেছে এবং এখনো চলছে, সেক্ষেত্রে প্রতিদিন হিসাবে গণ্য হবে।*	1 = হ্যাঁ<br>2 = না		
306	ABR_306	কত বছর বয়সে আপনি প্রথম ধোঁয়াবিহীন তামাক দ্রব্য গ্রহণ শুরু করেন?	বয়স |___|___| 77 = জানা নাই		
307	ABR_307	আপনার বাবা-মা বা অভিভাবকরা কোনও ধরনের তামাক ব্যবহার করেন?	1 = কেউ না<br>2 = আমার বাবা বা পুরুষ অভিভাবক<br>3 = আমার মা বা মহিলা অভিভাবক<br>4 = উভয়<br>5 = আমি জানি না<br>99 = অন্যান্য (নির্দিষ্ট করুন) ______		

অংশগ্রহণকারীর আইডি:|___|___|___|___|___|___|___|___|___|___|___|___|___|___|___|___|

খাদ্য নিরাপত্তা ও পুষ্টি বিষয়ক সার্ভিলেন্স

নম্বর	সূচক	প্রশ্ন	উত্তর	কোড	নির্দেশনা
**মূলঃ শাররীক পরিশ্রম সংক্রান্ত তথ্য**					
এর পর আমি আপনাকে সপ্তাহে আপনি বিভিন্ন ধরনের শারীরিক পরিশ্রমে যে সময় কাটান সে সম্পর্কিত কিছু প্রশ্ন করবো। আপনি নিজেকে শারীরিকভাবে সক্রিয় মনে না করলেও, অনুগ্রহ করে এই প্রশ্নগুলোর উত্তর দিন। প্রথমে আপনি কাজ করার জন্য যে সময় ব্যয় করেন সে সম্পর্কে ভাবুন। সেই কাজগুলোর কথা ভাবুন যে কাজগুলো আপনি টাকার বিনিময়ে বা বিনামূল্যে করে থাকনে। পড়াশুনা, প্রশিক্ষণ, গৃহস্থালীর কাজ, খাদ্যশস্যের চাষাবাদ, মাছ ধরা বা চাকুরী খোঁজা। এখানে অতিমাত্রার ভারী কাজ বলতে সেই কাজগুলোকে বুঝায় যে কাজগুলো করতে বেশী পরিমানে শারীরিক পরিশ্রমের প্রয়োজন হয় এবং কাজগুলো করার ফলে শ্বাস-প্রশ্বাস অথবা হৃদস্পন্দন অনেক বেড়ে যায় এবং মধ্যম মাত্রার কাজ বলতে সেই কাজগুলোকে বোঝায় যে কাজগুলো করতে মাঝারী পরিমানের শারীরিক পরিশ্রমের প্রয়োজন হয় এবং কাজগুলো করার ফলে শ্বাস-প্রশ্বাস অথবা হৃদস্পন্দন সামান্য বেড়ে যায়।<br>নির্দেশনাঃ উপরের ভূমিকাটি তথ্য প্রদানকারীকে পড়ে শুনান। এই অংশটি বাদ দেয়া যাবে না। তথ্য প্রদানকারীকে প্রথমে অবশ্যই তার দৈনন্দিন কাজগুলো সম্পর্কে চিন্তা করবে (পারিশ্রমিক ও পারিশ্রমিকবিহীন কাজ, গৃহস্থালীকাজ, খাদ্য উৎপাদন, খাওয়ার জন্য মাছ ধরা, কাজ খোঁজা, এক জায়গা থেকে অন্য জায়গায় যাওয়ার জন্য ব্যয়িত সময় এবং সবশেষে অবসর সময়ে ব্যয়িত সময়)					
**এখন আমি আপনার করা অতিমাত্রার ভারী কাজ সম্পর্কে জানতে চাইবো**					
দৈনন্দিন কাজের/ পেশাগত কাজের অংশ হিসেবে করা অতিমাত্রার ভারী কাজ					
308	ABR_308	আপনাকে কি দৈনন্দিন কাজের/পেশাগত কাজের অংশ হিসেবে শ্বাস প্রশ্বাস ও হৃদস্পন্দন অনেক বেড়ে যায় এমন কোন অতিমাত্রার ভারী কাজ/ অতিমাত্রার খেলাধুলা, শরীরচর্চা অথবা বিনোদন মূলক কাজ একনাগাড়ে কমপক্ষে 10 মিনিট ধরে করতে হয়? [অতিমাত্রার ভারী কাজ যেমন ভারী জিনিস বহন করা বা তোলা, মাটি কাটা, নির্মাণ কাজ, ধান কাটা, জাল দিয়ে মাছ ধরা ইত্যাদি, অতিমাত্রার খেলাধুলা, শরীরচর্চা অথবা বিনোদন মূলক কাজ: দৌড়ানো, কাবাডি, ফুটবল খেলা, দাড়িয়া বান্ধা, গোল্লাছুট, ইত্যাদি।]<br>[নমুনা কার্ড দেখান]<br>*নির্দেশনাঃ*<br>*তথ্যপ্রদানকারীকে শুধুমাত্র কর্মস্থলের 'ভারী কাজগুলো/ অবসর সময়ে ভারী কাজের কথা' সম্পর্কে চিন্তা করতে বলুন। ঐকাজগুলোই অতিমাত্রার ভারী কাজ হিসেবে গণ্য হবে যার ফলে শ্বাস-প্রশ্বাসের ও হৃদস্পন্দনের হার অতিমাত্রায় বৃদ্ধি পায়।*	1 = হ্যাঁ<br>2 = না<br>(যদি না হয়, তাহলে ABR_311 এ যান)		
309	ABR_309	আপনি দৈনন্দিন কাজের অংশ হিসেবে সপ্তাহে কয়দিন অতিমাত্রার ভারী কাজ / অতিমাত্রার খেলাধুলা, শরীরচর্চা অথবা বিনোদন মূলক কাজ করেন?<br>*নির্দেশনাঃ*<br>*সাধারন একটি সপ্তাহ হচ্ছে উত্তর দাতার একটি স্বাভাবিক সপ্তাহে যে কাজ করে। বৈধ উত্তর সীমা হচ্ছে 1-7 দিন।*	|___|___| দিন<br>77 = জানিনা<br>[জানিনা হলে ABR_311 এ যান]		
310	ABR_310	সাধারনত: আপনি দিনে কত সময় ধরে অতিমাত্রার ভারী কাজ/ অতিমাত্রার খেলাধুলা, শরীরচর্চা অথবা বিনোদন মূলক কাজ করেন?<br>*নির্দেশনাঃ*<br>*উত্তর দাতাকে তার কোন একটি দিনের কথা (যা সহজেই মনে আসে) চিন্তা করতে বলুন যে দিন তিনি কর্মক্ষেত্রে ভারী কাজে নিযুক্ত ছিলেন/যে দিন তিনি অবসর সময়ে ভারী শারীরিক পরিশ্রম করেছিলেন। উত্তর দাতা ঐ সকল ভারী কাজগুলোকেই মনে করবেন যেগুলো একটানা 10 মি বা তার অধিক সময় ধরে করা হয়েছে। অধিক বা অস্বাভাবিক (4 ঘন্টার অধিক) উত্তরগুলো যাচাই করুন।*	________<br>মিনিট		
**এখন আমি আপনার করা মাঝারি মাত্রার ভারী কাজ সম্পর্কে জানতে চাইবো।**					
দৈনন্দিন কাজের/পেশাগত কাজের বাইরে করা মাঝারী মাত্রার কাজ					

অংশগ্রহণকারীর আইডি:|___|___|___|___|___|___|___|___|___|___|___|___|___|___|___|___|

খাদ্য নিরাপত্তা ও পুষ্টি বিষয়ক সার্ভিলেন্স

নম্বর	সূচক	প্রশ্ন	উত্তর	কোড	নির্দেশনা
311	ABR_311	আপনাকে কি দৈনন্দিন কাজের/পেশাগত কাজের অংশ হিসেবে শ্বাসপ্রশ্বাস ও হৃদস্পন্দন সামান্য বেড়ে যায় এমন কোন মাঝারি মাত্রার কাজ/ মাঝারী মাত্রার খেলাধুলা, শরীরচর্চা অথবা বিনোদন মূলক কাজ একনাগাড়ে কমপক্ষে 10 মিনিট ধরে করতে হয়? যেমন, কাপড় ধোয়া, হালকা কিছু তোলা, ঝাড়ু দেওয়া, জানালা পরিষ্কার করা, রেদা বা কুড়ুনি দিয়ে চাঁছার কাজ করা, মেঝে ঝাড়ু দেওয়া, মোছা বা পরিষ্কার করার কাজ ইত্যাদি/ দ্রুত হাঁটা, ট্রেড মিলে হাঁটা, সাইকেল চালনা, সাঁতার কাটা, ভলিবল, জগিং।<br>[নমুনা কার্ড দেখান]<br>***নির্দেশনাঃ***<br>*উত্তর দাতা কে শুধুমাত্র কর্মস্থলের / অবসর সময়ে মাঝারিমাত্রার কাজগুলো' সম্পর্কে চিন্তা করতে বলুন। ঐ কাজগুলোই মাঝারি মাত্রার কাজ হিসেবে গণ্য হবে যার ফলে শ্বাস-প্রশ্বাসের ও হৃদস্পন্দনের হার সামান্য বৃদ্ধি পায়।*	1 = হ্যাঁ<br>2 = না<br>(যদি না হয়, তাহলে ABR_314 এ যান)		
312	ABR_312	আপনি দৈনন্দিন কাজের অংশ হিসেবে সপ্তাহে কয়দিন মাঝারি মাত্রার কাজ/ মাঝারি মাত্রার খেলাধুলা, শরীরচর্চা অথবা বিনোদন মূলক কাজ করেন?<br><br>***নির্দেশনাঃ***<br>*সাধারন একটি সপ্তাহ হচ্ছে উত্তর দাতার একটি স্বাভাবিক সপ্তাহে যে কাজ করে। বৈধ উত্তরসীমা হচ্ছে 1-7 দিন।*	|___|___| দিন<br>77 = জানিনা<br>[জানিনা হলে ABR_314 এ যান]		
313	ABR_313	সাধারনত আপনি দিনে কত সময় ধরে মাঝারিমাত্রার কাজ/ মাঝারি মাত্রার খেলাধুলা, শরীরচর্চা অথবা বিনোদন মূলক কাজ করেন?<br><br>নির্দেশনাঃ<br>উত্তর দাতাকে তার কোন একটি দিনের কথা (যা সহজেই মনে আসে) চিন্তা করতে বলুন যে দিন তিনি কর্মক্ষেত্রে মাঝারিমাত্রার কাজে নিযুক্ত ছিলেন/ যে দিন তিনি অবসর সময়ে মাঝারি মাত্রার কাজে নিযুক্ত ছিলেন। উত্তরদাতা ঐ সকল মাঝারিমাত্রার কাজগুলোকে আমলে আনবেন যেগুলো একটানা *10*মি বা তার অধিক সময় ধরে করা হয়েছে। অধিক/অস্বাভাবিক (*4* ঘন্টার অধিক) উত্তরগুলো যাচাই করুন।	________<br>মিনিট		
**অবসর সময়ের কাজের ধরন**					
**পরবর্তী প্রশ্নগুলো আপনার বসে বা হেলান দিয়ে কাটানো সময় সম্পর্কিত, যা কর্মস্থলে, বাড়িতে, এক জায়গা থেকে অন্য জায়গায় গাড়ী, বাস বা ট্রেনে করে যাতায়াত অথবা বন্ধুদের সাথে আড্ডায়, পড়াশোনা, কার্ড খেলা অথবা টেলিভিশন দেখার ক্ষেত্রে প্রযোজ্য। তবে এখানে ঘুমিয়ে কাটানো সময় অন্তর্ভূক্ত হবে না।**					
314	ABR_314	সাধারণত: দিনে কতটুকু সময় আপনি বসে/ হেলান দিয়ে অতিবাহিত করেন?<br>***নির্দেশনাঃ***<br>*উত্তরদাতাকে কাজকরার সময়, অফিসে, পড়াশোনার সময়, টেলিভিশন দেখার সময়, কম্পিউটার ব্যবহারের সময়, রান্নাঘরে হাতের কাজ করার সময়, বিশ্রামের সময় কতক্ষণ বসে কাটান। এখানে উত্তরদাতার ঘুমানোর সময় বিবেচ্য হবে না।*	________<br>মিনিট		
315	ABR_315	সাধারণত: দিনে কতটুকু সময় আপনি টেলিভিশন দেখেন?	________<br>মিনিট		

অংশগ্রহণকারীর আইডি:|___|___|___|___|___|___|___|___|___|___|___|___|___|___|___|___|

খাদ্য নিরাপত্তা ও পুষ্টি বিষয়ক সার্ভিলেন্স

পরিচ্ছেদ 4: মানসিক স্বাস্থ্য

নম্বর	সূচক	প্রশ্ন	উত্তর	কোড	নির্দেশনা
গত 2 সপ্তাহ ধরে আপনি কতটা ঘন ঘন নীচের সমস্যাগুলোর মুখোমুখি হয়েছেন?					
401	ABM_401	কোন কিছু করতে কম আগ্রহ বা কম আনন্দ	0 = একদমই নয়<br>1 = কোন কোন সময়<br>2 = বেশিরভাগ সময়<br>3 = সব সময়		
402	ABM_402	মন খারাপ, বিষন্ন, খিটখিটে, আশাহীন মনে হওয়া	0 = একদমই নয়<br>1 = কোন কোন সময়<br>2 = বেশিরভাগ সময়<br>3 = সব সময়		
403	ABM_403	ঘুম আসতে বা ঘুমিয়ে থাকতে অসুবিধা হওয়া বা অনেক বেশি ঘুমানো	0 = একদমই নয়<br>1 = কোন কোন সময়<br>2 = বেশিরভাগ সময়<br>3 = সব সময়		
404	ABM_404	ক্লান্ত লাগা বা অল্প শক্তি পাওয়া	0 = একদমই নয়<br>1 = কোন কোন সময়<br>2 = বেশিরভাগ সময়<br>3 = সব সময়		
405	ABM_405	খাবারে অরুচি, ওজন কমে যাওয়া বা বেশি বেশি খাওয়া	0 = একদমই নয়<br>1 = কোন কোন সময়<br>2 = বেশিরভাগ সময়<br>3 = সব সময়		
406	ABM_406	নিজের সম্পর্কে খারাপ অনুভুতি হওয়া, নিজেকে ব্যর্থ মনে করা, নিজেকে বা নিজের পরিবারকে ছোট করছি এমন মনে হওয়া	0 = একদমই নয়<br>1 = কোন কোন সময়<br>2 = বেশিরভাগ সময়<br>3 = সব সময়		
407	ABM_407	স্কুলের কাজ, কোন কিছু পড়া অথবা টিভি দেখার সময় মনোযোগ দিতে সমস্যা হওয়া	0 = একদমই নয়<br>1 = কোন কোন সময়<br>2 = বেশিরভাগ সময়<br>3 = সব সময়		
408	ABM_408	এত আস্তে কথা বলা বা চলাফেরা করা যা অন্যরা খেয়াল করতে পারে অথবা এর বিপরীত অর্থাৎ বেশি চঞ্চলতা বা স্বাভাবিকের চেয়ে বেশি নড়াচড়া করা	0 = একদমই নয়<br>1 = কোন কোন সময়<br>2 = বেশিরভাগ সময়<br>3 = সব সময়		
409	ABM_409	মরে গেলে বা নিজেকে কোনভাবেও আঘাত করলে ভালো হবে এমন মনে হওয়া<br>উত্তর না হলে, পরিচ্ছেদ 5 এ যান	0 = একদমই নয়<br>1 = কোন কোন সময়<br>2 = বেশিরভাগ সময়<br>3 = সব সময়		উত্তর 0 হলে, পরিচ্ছেদ 5 এ যান

রেফারেল (Referral)

নম্বর	সূচক	প্রশ্ন	উত্তর	কোড	নির্দেশনা
410	ABM_410	রেফার করা হয়েছে কি?	1 = হ্যাঁ 2 = না		উত্তর না হলে, পরিচ্ছেদ 5 এ যান
411	ABM_411	কোথায় রেফার করা হয়েছে	1 = উপজেলা স্বাস্থ্য কমপ্লেক্স<br>2 = জেলা হাসপাতাল<br>3 = মেডিকেল কলেজ হাসপাতাল<br>4 = ডাক্তারের চেম্বার<br>99 = অন্যান্য (নির্দিষ্ট করুন) ___________		

অংশগ্রহণকারীর আইডি:|___|___|___|___|___|___|___|___|___|___|___|___|___|___|___|___|

খাদ্য নিরাপত্তা ও পুষ্টি বিষয়ক সার্ভিলেন্স

পরিচ্ছেদ 5: পরিমাপ

<table>
<tr><th>নম্বর</th><th>সূচক</th><th>প্রশ্ন</th><th>উত্তর</th><th>কোড</th><th>নির্দেশনা</th></tr>
<tr><td>501</td><td>ABA_501</td><td>পরিমাপ নেয়া শুরুর সময়</td><td colspan="2">|____|____| : |____|____|
ঘন্টা : মিনিট</td><td>24 ঘণ্টার ফরম্যাট ব্যবহার করুন</td></tr>
<tr><td>502</td><td>ABA_502</td><td>ওজন পরিমাপক যন্ত্রের আইডি</td><td>|____|____|</td><td></td><td></td></tr>
<tr><td>503</td><td>ABA_503</td><td>দৈর্ঘ্য পরিমাপক যন্ত্রের আইডি</td><td>|____|____|</td><td></td><td></td></tr>
<tr><td>504</td><td>ABA_504</td><td>ওজন নেয়ার সময় গায়ের কাপড়ের ধরন</td><td>1 = হালকা কাপড়
2 = একটু ভারী কাপড়
3 = ভারী কাপড়</td><td></td><td></td></tr>
<tr><td>505</td><td>ABA_505</td><td>পরিমাপ গ্রহণকারীর নাম</td><td></td><td></td><td></td></tr>
<tr><td>506</td><td>ABA_506</td><td>পরিমাপ গ্রহণকারীর কোড</td><td>|____|____|</td><td></td><td></td></tr>
</table>

<table>
<tr><th>নম্বর</th><th>সূচক</th><th>পরিমাপের নাম</th><th>পরিমাপ 1</th><th>পরিমাপ 2</th><th>পরিমাপ 3</th></tr>
<tr><td>507</td><td>ABA_507 A/B/C</td><td>উচ্চতা (Cm)</td><td>A.|____|____|____|.|____|</td><td>B.|____|____|____|.|____|</td><td>C.|____|____|____|.|____|
যদি A ও B এর মধ্যে পার্থক্য 0.5cm এর বেশি হয়, তাহলে ৩য় বার মাপুন</td></tr>
<tr><td>508</td><td>ABA_508 A/B/C</td><td>ওজন (Kg)</td><td>A.|___|____|____|.|____|</td><td>B.|__ _|____|____|.|____|</td><td>C.|____|____|___|. |____|
যদি A ও B এর মধ্যে পার্থক্য 0.1Kg এর বেশি হয়, তাহলে ৩য় ওজন নিন</td></tr>
<tr><td>509</td><td>ABA_509 A/B/C</td><td>চর্বি (%)</td><td>A.|____|____|____|.|____|</td><td>B.|____|____|____|.|____|</td><td>C.|____|____|____|.|____|
যদি A ও B এর মধ্যে পার্থক্য 0.5cm এর বেশি হয়, তাহলে ৩য় বার মাপুন</td></tr>
<tr><td>510</td><td>ABA_510 A/B/C</td><td>পানি (%)</td><td>A.|___|____|____|.|____|</td><td>B.|__ _|____|____|.|____|</td><td>C.|____|____|____|. |____|
যদি A ও B এর মধ্যে পার্থক্য 0.1Kg এর বেশি হয়, তাহলে ৩য় ওজন নিন</td></tr>
<tr><td>511</td><td>ABA_511 A/B/C</td><td>কোমরের পরিধি (Cm)</td><td>A.|____|____|.|____|</td><td>B.|____|____|.|____|</td><td>C.|____|____|.|____|
যদি A ও B এর মধ্যে পার্থক্য 0.5cm এর বেশি হয়, তাহলে ৩য় বার মাপুন</td></tr>
<tr><td>512</td><td>ABA_512</td><td colspan="2">পরিমাপ সম্পর্কে তথ্য সংগ্রহকারীর মন্তব্য</td><td colspan="2"></td></tr>
<tr><td>513</td><td>ABA_513</td><td colspan="2">পরিমাপ গ্রহণের শেষের সময়</td><td>|____|____| : |____|____|
ঘন্টা : মিনিট</td><td>24 ঘণ্টার ফরম্যাট ব্যবহার করুন</td></tr>
</table>

অংশগ্রহণকারীর আইডি:|___|___|___|___|___|___|___|___|___|___|___|___|___|___|___|___|

খাদ্য নিরাপত্তা ও পুষ্টি বিষয়ক সার্ভিলেন্স

মডিউল 5: কিশোরী বালিকাদের প্রশ্নপত্র

পরিচ্ছেদ 1: সাধারণ তথ্যাবলী

নম্বর	সূচক	প্রশ্ন	উত্তর	কোড	নির্দেশনা
101	AG_101	আপনার নাম (ডাক নামসহ)	____________________		
102	AG_102	আপনার জন্ম তারিখ	|____|____|/|____|____|/20|____|____| দিন মাস বছর		দিন মাস না বলতে পারলে (09/09/2050)
103	AG_103	আপনার বয়স (বয়স পূর্ণ বছরে লিখুন)	|____|____| বছর		
104	AG_104	আপনার বৈবাহিক অবস্থা?	1 = কখনই বিবাহ করিনি<br>2 = বর্তমানে বিবাহিত<br>3 = পৃথক<br>4 = তালাকপ্রাপ্ত<br>5 = বিপত্নিক/বিধবা<br>88 = জানাতে অসম্মতি		
105	AG_105	আপনি (কিশোরীর নাম) সর্বমোট কত বছর প্রাতিষ্ঠানিক শিক্ষা গ্রহণ করেছেন?<br><br>(প্রথম শ্রেণীর নিচে এবং উপানুষ্ঠিক শিক্ষা অন্তর্ভুক্ত হবে না)<br><br>*নির্দেশনাঃ*<br>*তথ্য প্রদানকারীর সর্বমোট কত বছর প্রাতিষ্ঠানিক শিক্ষা সম্পন্ন করেছেন তা লিপিবদ্ধ করুন। প্রাতিষ্ঠানিক শিক্ষা না থাকলে 00 লিখুন।*	|____|____| বছর<br>প্রাথমিক শিক্ষা/এবতেদায়ী = 5<br>মাধ্যমিক/দাখিল =10<br>উচ্চ মাধ্যমিক/ ডিপ্লোমা/আলিম= 12<br>স্নাতক/ফাজিল =16<br>স্নাতাকোত্তর/কামিল/দাওরা =18		
106	AG_106	গত 12 মাসে আপনার (কিশোরীর নাম) পেশা কি ছিল?<br><br>যদি তথ্য প্রদানকারী গত 12 মাসে একাধিক পেশায় নিযুক্ত থাকেন তাহলে তিনি যে পেশাটিতে বেশী সময় ব্যয় করেছেন সেটিকে প্রধান পেশা হিসাবে বিবেচনা করুন এবং তা লিপিবদ্ধ করুন।	1 = কৃষিকাজ (ধান)<br>2 = কৃষিকাজ (ধান ছাড়া অন্যান্য)<br>3 = কৃষি দিনমজুর<br>4 = অদক্ষ দিনমজুর<br>5 = দক্ষ দিনমজুর<br>6 = রিক্সা/ ভ্যান/ ঠেলাগাড়ী/ বেবীট্যাক্সি ড্রাইভার/ নৌকার মাঝি<br>7 = জেলে<br>8 = চাকুরীজীবি<br>9 = পেশাজীবি<br>10 = ব্যবসায়ী<br>11 = ক্ষুদে ব্যবসায়ী<br>12 = গৃহপরিচারিকা<br>13 = জুমচাষী<br>14 = উপার্জন করে না<br>15 = হাঁস/ মুরগী পালন/ পশু পালন<br>16 = হস্তশিল্প<br>17 = শাক-সব্জি চাষ<br>18 = মৎস চাষ<br>19 = গৃহিনী<br>20 = ছাত্র/ছাত্রী<br>77 = জানিনা<br>66 = বয়স 6 বছরের কম<br>99 = অন্যান্য (নির্দিষ্ট করুন) __		
107	AG_107	কিশোরীর (কিশোরীর নাম) মা সর্বমোট কত বছর প্রাতিষ্ঠানিক শিক্ষা গ্রহণ করেছেন?<br><br>(প্রথম শ্রেণীর নিচে এবং উপানুষ্ঠিক শিক্ষা অন্তর্ভুক্ত হবে না)<br>*নির্দেশনাঃ তথ্য প্রদানকারীর সর্বমোট কত বছর প্রাতিষ্ঠানিক শিক্ষা সম্পন্ন করেছেন তা লিপিবদ্ধ করুন। প্রাতিষ্ঠানিক শিক্ষা না থাকলে 00 লিখুন।*	|____|____| বছর<br>প্রাথমিক শিক্ষা/এবতেদায়ী = 5<br>মাধ্যমিক/দাখিল =10<br>উচ্চ মাধ্যমিক/ ডিপ্লোমা/আলিম= 12<br>স্নাতক/ফাজিল =16<br>স্নাতাকোত্তর/কামিল/দাওরা =18		

নম্বর	সূচক	প্রশ্ন	উত্তর	কোড	নির্দেশনা
108	AG_108	গত 12 মাসে কিশোরীর মায়ের (কিশোরীর নাম) পেশা কি ছিল?<br>*নির্দেশনাঃ*<br>যদি তথ্য প্রদানকারী গত 12 মাসে একাধিক পেশায় নিযুক্ত থাকেন তাহলে তিনি যে পেশাটিতে বেশী সময় ব্যয় করেছেন সেটিকে প্রধান পেশা হিসাবে বিবেচনা করুন এবং তা লিপিবদ্ধ করুন।	1 = কৃষিকাজ (ধান)<br>2 = কৃষিকাজ (ধান ছাড়া অন্যান্য)<br>3 = কৃষি দিনমজুর<br>4 = অদক্ষ দিনমজুর<br>5 = দক্ষ দিনমজুর<br>6 = রিক্সা/ ভ্যান/ ঠেলাগাড়ী/ বেবীট্যাক্সি ড্রাইভার/ নৌকার মাঝি<br>7 = জেলে<br>8 = চাকুরীজীবি<br>9 = পেশাজীবি<br>10 = ব্যবসায়ী<br>11 = ক্ষুদে ব্যবসায়ী<br>12 = গৃহপরিচারিকা<br>13 = জুমচাষী<br>14 = উপার্জন করে না<br>15 = হাঁস/ মুরগী পালন/ পশু পালন<br>16 = হস্তশিল্প<br>17 = শাক-সব্জি চাষ<br>18 = মৎস চাষ<br>19 = গৃহিনী<br>20 = ছাত্র/ছাত্রী<br>77 = জানিনা<br>66 = বয়স 6 বছরের কম<br>99 = অন্যান্য (নির্দিষ্ট করুন) __		
109	AG_109	কিশোরীর (কিশোরীর নাম) বাবা সর্বমোট কত বছর প্রাতিষ্ঠানিক শিক্ষা গ্রহণ করেছেন?<br><br>(প্রথম শ্রেণীর নিচে এবং উপানুষ্ঠিক শিক্ষা অন্তর্ভুক্ত হবে না)<br><br>*নির্দেশনাঃ*<br>*সর্বমোট কত বছর প্রাতিষ্ঠানিক শিক্ষা সম্পন্ন করেছেন তা লিপিবদ্ধ করুন। প্রাতিষ্ঠানিক শিক্ষা না থাকলে 00 লিখুন।*	|____|____| বছর<br>প্রাথমিক শিক্ষা/এবতেদায়ী = 5<br>মাধ্যমিক/দাখিল =10<br>উচ্চ মাধ্যমিক/ ডিপ্লোমা/আলিম= 12<br>স্নাতক/ফাজিল =16<br>স্নাতাকোত্তর/কামিল/দাওরা =18		
110	AG_110	গত 12 মাসে কিশোরীর বাবার (কিশোরীর নাম) পেশা কি ছিল?<br><br>*নির্দেশনাঃ*<br>যদি তথ্য প্রদানকারী গত 12 মাসে একাধিক পেশায় নিযুক্ত থাকেন তাহলে তিনি যে পেশাটিতে বেশী সময় ব্যয় করেছেন সেটিকে প্রধান পেশা হিসাবে বিবেচনা করুন এবং তা লিপিবদ্ধ করুন।	1 = কৃষিকাজ (ধান)<br>2 = কৃষিকাজ (ধান ছাড়া অন্যান্য)<br>3 = কৃষি দিনমজুর<br>4 = অদক্ষ দিনমজুর<br>5 = দক্ষ দিনমজুর<br>6 = রিক্সা/ ভ্যান/ ঠেলাগাড়ী/ বেবীট্যাক্সি ড্রাইভার/ নৌকার মাঝি<br>7 = জেলে<br>8 = চাকুরীজীবি<br>9 = পেশাজীবি<br>10 = ব্যবসায়ী<br>11 = ক্ষুদে ব্যবসায়ী<br>12 = গৃহপরিচারিকা<br>13 = জুমচাষী<br>14 = উপার্জন করে না<br>15 = হাঁস/ মুরগী পালন/ পশু পালন<br>16 = হস্তশিল্প<br>17 = শাক-সব্জি চাষ<br>18 = মৎস চাষ<br>19 = গৃহিনী<br>20 = ছাত্র/ছাত্রী<br>77 = জানিনা<br>66 = বয়স 6 বছরের কম<br>99 = অন্যান্য (নির্দিষ্ট করুন) __		

অংশগ্রহণকারীর আইডি:|___|___|___|___|___|___|___|___|___|___|___|___|___|___|___|___|

পরিচ্ছেদ 2ঃ খাদ্য গ্রহণের বৈচিত্র্য

নম্বর	সূচক	প্রশ্ন		গতকাল দিনে বা রাতে আপনি নিম্নবর্ণিত খাবারগুলো খেয়েছেন কি? (A)	গত 7 দিনে আপনি নিম্নবর্ণিত খাবারগুলো কতদিন খেয়েছেন? (B)	গত 7 দিনে আপনি নিম্নবর্ণিত খাবারগুলো কতবার খেয়েছেন? (C)
এখন আমি আপনাকে গতকাল দিনে বা রাতে (গতকাল সকাল 6:00 টা থেকে আজ সকাল 6:00 টা পর্যন্ত) আপনি বাড়ীতে ও বাড়ীর বাইরে যা যা খেয়েছেন তা বর্ণণা করতে অনুরোধ করছি। দয়া করে সব ধরনের খাবার, পানীয় যা আপনি সকাল, দুপুর ও রাতের খাবারের সাথে খেয়েছেন বা নাস্তা/হালকা নাস্তা করেছেন সে সম্পর্কে বলুন। আপনি খাবার তৈরীর সময় কোন খাবার খেয়ে থাকলে তাও মনে করে আমাদেরকে বলুন। আপনি গতকাল সকালে যা খেয়েছেন তা দিয়েই শুরু করুন।<br>- আপনি সকালে ঘুম থেকে উঠে কী কী খেয়েছেন? আরো কিছু খেয়েছেন কি?<br>- সকালে আরো কী কী খেয়েছেন? আরো কিছু খেয়েছেন কি?<br>- দুপুরে কী কী খেয়েছেন? আরো কিছু খেয়েছেন কি?<br>- বিকালে কী কী খেয়েছেন? আরো কিছু খেয়েছেন কি?<br>- সন্ধ্যায় কী কী খেয়েছেন? আরো কিছু খেয়েছেন কি?<br>- রাতে কী কী খেয়েছেন? আরো কিছু খেয়েছেন কি?<br>A কলামের প্রশ্নগুলোর উত্তর প্রথমে রেকর্ড করুন। A কলামের প্রশ্নগুলোর উত্তর রেকর্ড করা শেষ হলে B/ C কলামের উত্তর নিন।						
		খাবার ধরণ	খাবার নমুনা			
201	AGD_201 A/B/C	শ্বেতসার জাতীয় (শস্য জাতীয় খাবার)	ভাত, আটা রুটি, গম, মুড়ি, ভুট্টা, খিচুড়ি, বার্লি, ওট, কিনোয়া, নুডলস, পাস্তা	1 = হ্যাঁ<br>2 = না	____ ____ দিন	____ ____ বার
202	AGD_202 A/B/C	শ্বেতসার জাতীয় (মূল, কন্দ, কলা)	গোলআলু, মিষ্টিআলু, সাগু, এরারুট, কাঁচকলা, শালগম, কাসাভা, কচু, কচুমুখী, পাকাকলা, শালুক	1 = হ্যাঁ<br>2 = না	____ ____ দিন	____ ____ বার
203	AGD_203 A/B/C	ডাল ও ডাল জাতীয় খাবার	ডাল, শিমের বীচি, মটর, সয়বীন, টফু, হুমাস	1 = হ্যাঁ<br>2 = না	____ ____ দিন	____ ____ বার
204	AGD_204 A/B/C	বাদাম ও তৈলবীজ	চীনা বাদাম, পেস্তা, কাজু, অথবা যেকোন বাদাম, চিয়া সীড, তিল, তিসি, সূর্যমুখী বীজ, মিষ্টি কুমড়া বীজ	1 = হ্যাঁ<br>2 = না	____ ____ দিন	____ ____ বার
205	AGD_205 A/B/C	গাঢ় সবুজ পাতা জাতীয় শাক	সকল ধরনের পাতা জাতীয় শাক (পুঁই, কচু, কলমি), ব্রকলি	1 = হ্যাঁ<br>2 = না	____ ____ দিন	____ ____ বার
206	AGD_206 A/B/C	লাল/কমলা/ হলুদ সব্জি	মিষ্টিকুমড়া, গাজর, গাঢ় হলুদ বা কমলা মিষ্টি আলু, ও অন্যান্য লাল/ কমলা/হলুদ রঙের সব্জি	1 = হ্যাঁ<br>2 = না	____ ____ দিন	____ ____ বার
207	AGD_207 A/B/C	লাল/কমলা/ হলুদ ফলমূল	পাকা আম, পাকা পেঁপে ও অন্যান্য লাল/কমলা/ হলুদ ফলমূল	1 = হ্যাঁ<br>2 = না	____ ____ দিন	____ ____ বার
208	AGD_208 A/B/C	ভিটামিন সি- সমৃদ্ধ ফল	পেয়ারা, স্ট্রবেরী, লেবু, কমলালেবু, আঙ্গুর, আনারস, কাঁচা আম, আমলকি, কিউই, টমেটো	1 = হ্যাঁ<br>2 = না	____ ____ দিন	____ ____ বার
209	AGD_209 A/B/C	ভিটামিন সি- সমৃদ্ধ সব্জি	কাঁচা টমেটো, কাঁচা মরিচ, ব্রাসেলস স্প্রাউট, ফুলকপি, বাঁধাকপি	1 = হ্যাঁ<br>2 = না	____ ____ দিন	____ ____ বার

নম্বর	সূচক	প্রশ্ন		গতকাল দিনে বা রাতে আপনি নিম্নবর্ণিত খাবারগুলো খেয়েছেন কি? (A)	গত 7 দিনে আপনি নিম্নবর্ণিত খাবারগুলো কতদিন খেয়েছেন? (B)	গত 7 দিনে আপনি নিম্নবর্ণিত খাবারগুলো কতবার খেয়েছেন? (C)
210	AGD_210 A/B/C	অন্যান্য শাক সব্জি	শিম, অ্যাসপারাগাস, বিট, কচি বাঁশ, ফুলকপি, সেলারি, শসা, বেগুন, লেটুস, মাশরুম, মূলা, জুকিনি	1 = হ্যাঁ<br>2 = না	|____|____| দিন	|____|____| বার
211	AGD_211 A/B/C	অন্যান্য ফলমূল	আপেল, আভাকাডো, জাম, চেরী, পাকা কাঁঠাল	1 = হ্যাঁ<br>2 = না	|____|____| দিন	|____|____| বার
212	AGD_212 A/B/C	ডিম	হাঁস-মুরগী, অন্যান্য পাখী	1 = হ্যাঁ<br>2 = না	|____|____| দিন	|____|____| বার
213	AGD_213 A/B/C	অঙ্গ জাতীয় মাংস	গিলা, কলিজা, পাকস্থলী, হৃদপিণ্ড, কিডনি	1 = হ্যাঁ<br>2 = না	|____|____| দিন	|____|____| বার
214	AGD_214 A/B/C	মাংস	গরু, শুকর, বাছুর, মেষশাবক, ছাগল, মুরগী, হাঁস বা যেকোন প্রাণীর মাংস	1 = হ্যাঁ<br>2 = না	|____|____| দিন	|____|____| বার
215	AGD_215 A/B/C	ছোট মাছ	ছোট মাছের কাঁটা/হাড়সহ (কাঁচকি, মলা, ঢেলা, চাপিলা, বাতাসি, ছোট চিংড়ি, ছোট মাছের শুঁটকি)	1 = হ্যাঁ<br>2 = না	|____|____| দিন	|____|____| বার
216	AGD_216 A/B/C	বড় মাছ/সামুদ্রিক মাছ/ সামুদ্রিক খাবার	বড় মাছ, ঝিনুক, কাঁকড়া, অক্টোপাস, স্কুইড, হাঙর, বড় চিংড়ি, বড় মাছের শুঁটকি	1 = হ্যাঁ<br>2 = না	|____|____| দিন	|____|____| বার
217	AGD_217 A/B/C	দুধ ও দুগ্ধ জাতীয় খাবার	দুধ, পনির, দই এবং অন্যান্য দুগ্ধজাতীয় খাদ্য	1 = হ্যাঁ<br>2 = না	|____|____| দিন	|____|____| বার
218	AGD_218 A/B/C	পোকা ও অন্যান্য আমিষ জাতীয় খাবার	মাছের ডিম, পোকা, শামুক	1 = হ্যাঁ<br>2 = না	|____|____| দিন	|____|____| বার
219	AGD_219 A/B/C	তৈল জাতীয় খাবার	ঘি, মাখন, ক্রিম, সর, চর্বি, মার্জারিন, ম্যায়োনেজ, পাম অয়েল, উদ্ভিজ্জ তেল	1 = হ্যাঁ<br>2 = না	|____|____| দিন	|____|____| বার
220	AGD_220 A/B/C	চিনিযুক্ত এবং ভাজা খাবার	খাস্তা, চিপস ও অন্যান্য ভাজা খাবার, সিংগারা, সমোচা	1 = হ্যাঁ<br>2 = না	|____|____| দিন	|____|____| বার
221	AGD_221 A/B/C	মিষ্টি জাতীয় খাবার	চিনিযুক্ত খাবার, যেমন চকলেটস, ক্যান্ডিস, কুকিস/মিষ্টি বিস্কুট এবং কেকস, মিষ্টি পেষ্ট্রি বা আইসক্রিম, যে কোন মিষ্টি, মধু, হালুয়া, কনডেন্সড দুধ, তিলের খাজা	1 = হ্যাঁ<br>2 = না	|____|____| দিন	|____|____| বার
222	AGD_222 A/B/C	মিষ্টি পানীয়	চিনি যুক্ত চা, কোমল পানীয়, জুস, এনার্জি ড্রিংক, ইয়োগার্ট ড্রিংক, চকলেট ড্রিংক, হরলিকস, মলটোভা	1 = হ্যাঁ<br>2 = না	|____|____| দিন	|____|____| বার

নম্বর	সূচক	প্রশ্ন		গতকাল দিনে বা রাতে আপনি নিম্নবর্ণিত খাবারগুলো খেয়েছেন কি? (A)	গত 7 দিনে আপনি নিম্নবর্ণিত খাবারগুলো কতদিন খেয়েছেন? (B)	গত 7 দিনে আপনি নিম্নবর্ণিত খাবারগুলো কতবার খেয়েছেন? (C)
223	AGD_223 A/B/C	অন্যান্য পানীয় এবং খাবার	চিনি ছাড়া চা, মদ, বিয়ার, আচার, সুপ, উপরের তালিকাভুক্ত নয় এমন যেকোন খাবার	1 = হ্যাঁ<br>2 = না	|____|____| দিন	|____|____| বার
224	AGD_224 A/B/C	অন্যান্য খাবার সুস্বাদু করার দ্রব্যাদি	মসলা, ধনে পাতা, সস, রসুন, কেচাপ, লেবুর রস, পুদিনা পাতা, পান, সুপারি, তামাক পাতা, জর্দা	1 = হ্যাঁ<br>2 = না	|____|____| দিন	|____|____| বার

নম্বর	সূচক	প্রশ্ন	উত্তর	কোড	নির্দেশনা
আমি আপনাকে পরবর্তীতে যে প্রশ্নগুলো জিজ্ঞাসা করতে যাচ্ছি তা হল সচরাচর আপনি যে সকল ফলমূল ও শাক-শজি খেয়ে থাকেন সে বিষয়ে। আমার কাছে ফল ও শাক-শজির কিছু ছবি আছে। প্রতিটি ছবি এক একটি প্রমান মাপের সমান। উত্তর দেওয়ার সময় সাধারন 1টি সপ্তাহের কথা চিন্তা করুন। (মাসে 1-2 বার হলে উত্তর 00 হবে)					
225	AGD_225	সচরাচর সপ্তাহের কত দিন আপনি ফল খান ?<br>(নমুনা কার্ড দেখান)<br>*নির্দেশনাঃ*<br>*তথ্য প্রদানকারীকে নমুনা কার্ডে প্রদর্শিত ফলগুলো দেখিয়ে চিন্তা করতে বলুন। এখানে প্যাকেটজাত ফলের জুস গ্রহণযোগ্য নয় তবে বাসায় ব্লেন্ড করা ফলের জুস গ্রহণযোগ্য। সপ্তাহ বলতে ধর্মীয় বা অন্য কোন বিশেষ উপলক্ষ্য ব্যতীত একটি স্বাভাবিক সপ্তাহ বুঝায়। মাসে 1-2 বার হলে 00 লিখুন।*	দিনের সংখ্যা<br>|___|___| 77 = জানা নাই<br>(যদি 00 দিন হয়, তাহলে AGD_227-তে যান)		
226	AGD_226	সেই দিন গুলির একদিনে কতটুকু ফল খেয়েছেন?<br>(নমুনা কার্ড ও বাটি দেখান)<br>*নির্দেশনাঃ*<br>*তথ্য প্রদানকারীকে যে কোন একদিনের কথা স্মরণ করতে বলুন এবং বাটি দেখিয়ে পরিমাপ করতে বলুন।*	সারভিং সংখ্যা<br>|___|___|.|___| 77.7 = জানা নাই		
227	AGD_227	সচরাচর সপ্তাহের কত দিন আপনি শাক-সজি খান?<br>(নমুনা কার্ড দেখান)<br>*নির্দেশনাঃ*<br>*তথ্য প্রদানকারীকে নমুনা কার্ডে প্রদর্শিত শাক-সজিগুলো দেখিয়ে চিন্তা করতে বলুন। এখানে আলু শাক-সজি হিসাবে গণ্য হবে না। কাঁচা ও রান্না করা শাক-সজির প্রমান পরিমাপ আলাদা করে দেখান। মাছের সাথে রান্না করা সজির ক্ষেত্রে শুধু সজির পরিমান করতে হবে। সপ্তাহ বলতে ধর্মীয় বা অন্য কোন বিশেষ উপলক্ষ্য ব্যতীত একটি স্বাভাবিক সপ্তাহ বুঝায়। মাসে 1-2 বার হলে '00' হবে।*	দিনের সংখ্যা<br>|___|___| 77 = জানা নাই (যদি '00' দিন হয়, তাহলে AGD_229-তে যান)		

নম্বর	সূচক	প্রশ্ন	উত্তর	কোড	নির্দেশনা
228	AGD_228	সেই দিন গুলির একদিনে কতটুকু পরিমান শাক-সব্জি খেয়েছেন? (নমুনা কার্ড ও বাটি দেখান)<br><br>***নির্দেশনাঃ***<br>*তথ্য প্রদানকারীকে যে কোন একদিনের কথা স্মরণ করতে বলুন এবং বাটি দেখিয়ে পরিমাপ করতে বলুন।*	সারভিং সংখ্যা<br>|___|___|.|___| 77.7 = জানা নাই		
229	AGD_229	আপনি কি বর্তমানে কোন ধরণের ভিটামিন/খনিজ লবন আছে এমন কোন ট্যাবলেট, ক্যাপসুল, সিরাপ খান?	1 = হ্যাঁ<br>2 = না		যদি 'না' হয় তাহলে পরবর্তী পরিচ্ছেদে যান
230	AGD_230	ব্যবস্থাপত্র দেখে বা ঔষধ দেখে নাম লিখুন	A.________________<br>B.________________<br>C.________________		

অংশগ্রহণকারীর আইডি:|___|___|___|___|___|___|___|___|___|___|___|___|___|___|___|___|

খাদ্য নিরাপত্তা ও পুষ্টি বিষয়ক সার্ভিলেন্স

পরিচ্ছেদ 3ঃ আচরণগত ঝুঁকির কারণ (ধুমপান/ধোঁয়াহীন তামাক এবং শারীরিক পরিশ্রম)

নম্বর	সূচক	প্রশ্ন	উত্তর	কোড	নির্দেশনা
**মূলঃ তামাকের ব্যবহার**					
এখন আমি আপনাকে তামাক ও তামাকজাত দ্রব্যের (যেমনঃ ধূমপান, ধোঁয়াবিহীন তামাকের) ব্যবহার সম্পর্কে কিছু প্রশ্ন জিজ্ঞাসা করব।					
301	AGR_301	আপনি কি বর্তমানে কোন প্রকার ধূমপান করেন?<br>(যেমন: সিগারেট, বিড়ি, হুক্কা, চুরুট, সিগার)<br>[নমুনা কার্ড দেখান]<br>*নির্দেশনাঃ*<br>*উত্তরদাতাকে নমুনা কার্ড দেখিয়ে চিন্তা করতে বলুন যে বর্তমানে তিনি কোন দ্রব্যটি ধূমপান/ ব্যবহার করছেন।*	1 = হ্যাঁ<br>2 = না<br>(যদি না হয়, তাহলে AGR_304 এ যান)		
302	AGR_302	আপনি কি বর্তমানে প্রতিদিন ধূমপান করেন?<br><br>*নির্দেশনাঃ*<br>*এই প্রশ্নটি যারা বর্তমানে ধূমপান করেন তাদের জন্য প্রযোজ্য।*<br>***প্রতিদিন অর্থঃ***<br>*প্রায় একমাস বা তার বেশী সময় ধরে প্রতিদিন অন্তত একবার ধূমপান করা কে বুঝায়। যদি এমন হয় যে তথ্য প্রদানকারী 25 দিন হল ধূমপান শুরু করেছে এবং এখনো চলছে, সেক্ষেত্রে প্রতিদিন হিসাবে গণ্য হবে।*	1 = হ্যাঁ<br>2 = না		
303	AGR_303	কত বছর বয়সে আপনি প্রথম ধূমপান শুরু করেন?	বয়স |___|___| 77 = জানা নাই		
304	AGR_304	আপনি কি বর্তমানে কোন প্রকার ধোঁয়াবিহীন তামাক দ্রব্য ব্যবহার করেন?<br>(যেমনঃ পানের সাথে জর্দা, শুধু জর্দা, সুপারির সাথে জর্দা, পানের সাথে সাদাপাতা, তামাকযুক্ত পানমশলা, চিবিয়ে খাওয়া সাদাপাতা, খৈনি, নস্যি, গুল, ইত্যাদি)<br>[নমুনা কার্ড দেখান]<br><br>*নির্দেশনাঃ*<br>*তথ্য প্রদানকারীকে ধোঁয়বিহীন তামাক যেমনঃ জর্দা, গুল, সাদাপাতা, খৈনি, নস্যি দ্রব্যগুলো কি বর্তমানে ব্যবহার করেন কিনা তা চিন্তা করে উত্তর দিতে বলুন। এক্ষেত্রে, শুধু পান সুপারী ও চুন প্রযোজ্য হবে না। যদি তথ্য প্রদানকারী পানের সাথে জর্দা বা শুধু জর্দা, পানের সাথে সাদাপাতা বা শুধু সাদাপাতা, পানের সাথে তামাক যুক্ত পান মশলা বা শুধু তামাক যুক্ত পান মশলা খান তাহলে ধোঁয়াবিহীন তামাক সেবন হিসাবে গণ্য হবে।*	1 = হ্যাঁ<br>2 = না<br>(যদি না হয়, তাহলে AGR_306 এ যান)		
305	AGR_305	আপনি কি বর্তমানে প্রতিদিন এই ধোঁয়াবিহীন তামাক দ্রব্য ব্যবহার করেন?<br>**প্রতিদিন অর্থঃ** প্রায় একমাস বা তার বেশী সময় ধরে প্রতিদিন অন্তঃত একটি ধোঁয়াবিহীন তামাক পণ্য ব্যবহার করা কে বুঝায়। *যদি এমন হয় যে তথ্য প্রদানকারী 25 দিন হল ধূমপান শুরু করেছে এবং এখনো চলছে, সেক্ষেত্রে প্রতিদিন হিসাবে গণ্য হবে।*	1 = হ্যাঁ<br>2 = না		
306	AGR_306	কত বছর বয়সে আপনি প্রথম ধোঁয়াবিহীন তামাক দ্রব্য গ্রহণ শুরু করেন?	বয়স |___|___| 77 = জানা নাই		
307	AGR_307	আপনার বাবা-মা বা অভিভাবকরা কোনও ধরনের তামাক ব্যবহার করেন?	1 = কেউ না<br>2 = আমার বাবা বা পুরুষ অভিভাবক<br>3 = আমার মা বা মহিলা অভিভাবক<br>4 = উভয়<br>5 = আমি জানি না<br>99 = অন্যান্য (নির্দিষ্ট করুন) _______		

নম্বর	সূচক	প্রশ্ন	উত্তর	কোড	নির্দেশনা
**মূলঃ শাররীক পরিশ্রম সংক্রান্ত তথ্য**					
এর পর আমি আপনাকে সপ্তাহে আপনি বিভিন্ন ধরনের শারীরিক পরিশ্রমে যে সময় কাটান সে সম্পর্কিত কিছু প্রশ্ন করবো। আপনি নিজেকে শারীরিকভাবে সক্রিয় মনে না করলেও, অনুগ্রহ করে এই প্রশ্নগুলোর উত্তর দিন। প্রথমে আপনি কাজ করার জন্য যে সময় ব্যয় করেন সে সম্পর্কে ভাবুন। সেই কাজগুলোর কথা ভাবুন যে কাজগুলো আপনি টাকার বিনিময়ে বা বিনামূল্যে করে থাকনে। পড়াশুনা, প্রশিক্ষণ, গৃহস্থালীর কাজ, খাদ্যশস্যের চাষাবাদ, মাছ ধরা বা চাকুরী খোঁজা। এখানে অতিমাত্রার ভারী কাজ বলতে সেই কাজগুলোকে বুঝায় যে কাজগুলো করতে বেশী পরিমানে শারীরিক পরিশ্রমের প্রয়োজন হয় এবং কাজগুলো করার ফলে শ্বাস-প্রশ্বাস অথবা হৃদস্পন্দন অনেক বেড়ে যায় এবং মধ্যম মাত্রার কাজ বলতে সেই কাজগুলোকে বোঝায় যে কাজগুলো করতে মাঝারী পরিমানের শারীরিক পরিশ্রমের প্রয়োজন হয় এবং কাজগুলো করার ফলে শ্বাস-প্রশ্বাস অথবা হৃদস্পন্দন সামান্য বেড়ে যায়।<br>নির্দেশনাঃ উপরের ভূমিকাটি তথ্য প্রদানকারীকে পড়ে শুনান। এই অংশটি বাদ দেয়া যাবে না। তথ্য প্রদানকারীকে প্রথমে অবশ্যই তার দৈনন্দিন কাজগুলো সম্পর্কে চিন্তা করবে (পারিশ্রমিক ও পারিশ্রমিকবিহীন কাজ, গৃহস্থালীকাজ, খাদ্য উৎপাদন, খাওয়ার জন্য মাছ ধরা, কাজ খোঁজা, এক জায়গা থেকে অন্য জায়গায় যাওয়ার জন্য ব্যয়িত সময় এবং সবশেষে অবসর সময়ে ব্যয়িত সময়)					
**এখন আমি আপনার করা অতিমাত্রার ভারী কাজ সম্পর্কে জানতে চাইবো**					
দৈনন্দিন কাজের/ পেশাগত কাজের অংশ হিসেবে করা অতিমাত্রার ভারী কাজ					
308	AGR_308	আপনাকে কি দৈনন্দিন কাজের/পেশাগত কাজের অংশ হিসেবে শ্বাস প্রশ্বাস ও হৃদস্পন্দন অনেক বেড়ে যায় এমন কোন অতিমাত্রার ভারী কাজ/ অতিমাত্রার খেলাধুলা, শরীরচর্চা অথবা বিনোদন মূলক কাজ একনাগাড়ে কমপক্ষে 10 মিনিট ধরে করতে হয়? [অতিমাত্রার ভারী কাজ যেমন ভারী জিনিস বহন করা বা তোলা, মাটি কাটা, নির্মাণ কাজ, ধান কাটা, জাল দিয়ে মাছ ধরা ইত্যাদি, অতিমাত্রার খেলাধুলা, শরীরচর্চা অথবা বিনোদন মূলক কাজ: দৌড়ানো, কাবাডি, ফুটবল খেলা, দাড়িয়া বান্ধা, গোল্লাছুট, ইত্যাদি।] [নমুনা কার্ড দেখান]<br><br>*নির্দেশনাঃ*<br>*তথ্যপ্রদানকারীকে শুধুমাত্র কর্মস্থলের 'ভারী কাজগুলো/ অবসর সময়ে ভারী কাজের কথা' সম্পর্কে চিন্তা করতে বলুন। ঐকাজগুলোই অতিমাত্রার ভারী কাজ হিসেবে গণ্য হবে যার ফলে শ্বাস-প্রশ্বাসের ও হৃদস্পন্দনের হার অতিমাত্রায় বৃদ্ধি পায়।*	1 = হ্যাঁ<br>2 = না<br>(যদি না হয়, তাহলে AGR_311 এ যান)		
309	AGR_309	আপনি দৈনন্দিন কাজের অংশ হিসেবে সপ্তাহে কয়দিন অতিমাত্রার ভারী কাজ / অতিমাত্রার খেলাধুলা, শরীরচর্চা অথবা বিনোদন মূলক কাজ করেন?<br><br>*নির্দেশনাঃ*<br>*সাধারন একটি সপ্তাহ হচ্ছে উত্তর দাতার একটি স্বাভাবিক সপ্তাহে যে কাজ করে। বৈধ উত্তর সীমা হচ্ছে 1-7 দিন।*	|___|___| দিন<br>77 = জানিনা<br>[জানিনা হলে AGR_311 এ যান]		
310	AGR_310	সাধারনত: আপনি দিনে কত সময় ধরে অতিমাত্রার ভারী কাজ/ অতিমাত্রার খেলাধুলা, শরীরচর্চা অথবা বিনোদন মূলক কাজ করেন?<br><br>*নির্দেশনাঃ*<br>*উত্তর দাতাকে তার কোন একটি দিনের কথা (যা সহজেই মনে আসে) চিন্তা করতে বলুন যে দিন তিনি কর্মক্ষেত্রে ভারী কাজে নিযুক্ত ছিলেন/যে দিন তিনি অবসর সময়ে ভারী শারীরিক পরিশ্রম করেছিলেন। উত্তর দাতা ঐ সকল ভারী কাজগুলোকেই মনে করবেন যেগুলো একটানা 10 মি বা তার অধিক সময় ধরে করা হয়েছে। অধিক বা অস্বাভাবিক (4 ঘন্টার অধিক) উত্তরগুলো যাচাই করুন।*	____________ মিনিট		
**এখন আমি আপনার করা মাঝারি মাত্রার ভারী কাজ সম্পর্কে জানতে চাইবো।**					
দৈনন্দিন কাজের/পেশাগত কাজের বাইরে করা মাঝারী মাত্রার কাজ					

নম্বর	সূচক	প্রশ্ন	উত্তর	কোড	নির্দেশনা
311	AGR_311	আপনাকে কি দৈনন্দিন কাজের/পেশাগত কাজের অংশ হিসেবে শ্বাসপ্রশ্বাস ও হৃদস্পন্দন সামান্য বেড়ে যায় এমন কোন মাঝারি মাত্রার কাজ/ মাঝারী মাত্রার খেলাধুলা, শরীরচর্চা অথবা বিনোদন মূলক কাজ একনাগাড়ে কমপক্ষে 10 মিনিট ধরে করতে হয়? যেমন, কাপড় ধোয়া, হালকা কিছু তোলা, ঝাড়ু দেওয়া, জানালা পরিষ্কার করা, রেদা বা কুড়ুনি দিয়ে চাঁছার কাজ করা, মেঝে ঝাড়ু দেওয়া, মোছা বা পরিষ্কার করার কাজ ইত্যাদি/ দ্রুত হাঁটা, ট্রেড মিলে হাঁটা, সাইকেল চালনা, সাঁতার কাটা, ভলিবল, জগিং।<br>[নমুনা কার্ড দেখান]<br>*নির্দেশনাঃ*<br>*উত্তর দাতা কে শুধুমাত্র কর্মস্থলের / অবসর সময়ে মাঝারিমাত্রার কাজগুলো' সম্পর্কে চিন্তা করতে বলুন। ঐ কাজগুলোই মাঝারি মাত্রার কাজ হিসেবে গণ্য হবে যার ফলে শ্বাস-প্রশ্বাসের ও হৃদস্পন্দনের হার সামান্য বৃদ্ধি পায়।*	1 = হ্যাঁ<br>2 = না<br>(যদি না হয়, তাহলে AGR_314 এ যান)		
312	AGR_312	আপনি দৈনন্দিন কাজের অংশ হিসেবে সপ্তাহে কয়দিন মাঝারি মাত্রার কাজ/ মাঝারি মাত্রার খেলাধুলা, শরীরচর্চা অথবা বিনোদন মূলক কাজ করেন?<br>*নির্দেশনাঃ*<br>*সাধারন একটি সপ্তাহ হচ্ছে উত্তর দাতার একটি স্বাভাবিক সপ্তাহে যে কাজ করে। বৈধ উত্তরসীমা হচ্ছে 1-7 দিন।*	|___|___| দিন<br>77 = জানিনা<br>[জানিনা হলে AGR_314 এ যান]		
313	AGR_313	সাধারনত আপনি দিনে কত সময় ধরে মাঝারিমাত্রার কাজ/ মাঝারি মাত্রার খেলাধুলা, শরীরচর্চা অথবা বিনোদন মূলক কাজ করেন?<br>নির্দেশনাঃ<br>উত্তর দাতাকে তার কোন একটি দিনের কথা (যা সহজেই মনে আসে) চিন্তা করতে বলুন যে দিন তিনি কর্মক্ষেত্রে মাঝারিমাত্রার কাজে নিয়ুক্ত ছিলেন/ যে দিন তিনি অবসর সময়ে মাঝারি মাত্রার কাজে নিয়ুক্ত ছিলেন। উত্তরদাতা ঐ সকল মাঝারিমাত্রার কাজগুলোকে আমলে আনবেন যেগুলো একটানা *10*মি বা তার অধিক সময় ধরে করা হয়েছে। অধিক/অস্বাভাবিক (*4* ঘন্টার অধিক) উত্তরগুলো যাচাই করুন।	___________ মিনিট		
**অবসর সময়ের কাজের ধরন**					
**পরবর্তী প্রশ্নগুলো আপনার বসে বা হেলান দিয়ে কাটানো সময় সম্পর্কিত, যা কর্মস্থলে, বাড়িতে, এক জায়গা থেকে অন্য জায়গায় গাড়ী, বাস বা ট্রেনে করে যাতায়াত অথবা বন্ধুদের সাথে আড্ডায়, পড়াশোনা, কার্ড খেলা অথবা টেলিভিশন দেখার ক্ষেত্রে প্রযোজ্য। তবে এখানে ঘুমিয়ে কাটানো সময় অন্তর্ভূক্ত হবে না।**					
314	AGR_314	সাধারণত: দিনে কতটুকু সময় আপনি বসে/ হেলান দিয়ে অতিবাহিত করেন?<br>*নির্দেশনাঃ*<br>*উত্তরদাতাকে কাজকরার সময়, অফিসে, পড়াশোনার সময়, টেলিভিশন দেখার সময়, কম্পিউটার ব্যবহারের সময়, রান্নাঘরে হাতের কাজ করার সময়, বিশ্রামের সময় কতক্ষণ বসে কাটান। এখানে উত্তরদাতার ঘুমানোর সময় বিবেচ্য হবে না।*	___________ মিনিট		
315	AGR_315	সাধারণত: দিনে কতটুকু সময় আপনি টেলিভিশন দেখেন?	___________ মিনিট		

পরিচ্ছেদ 4: প্রজনন ইতিহাস, মাসিকের সময় পরিস্কার পরিচ্ছন্নতা

নম্বর	সূচক	প্রশ্ন	উত্তর	কোড	নির্দেশনা
401	AGM_401	কত বছর বয়সে আপনার প্রথম মাসিক হয়?	|____|____| বছর		এখনও শুরু না হয়ে থাকলে ০০ লিখুন
402	AGM_402	আপনার বৈবাহিক অবস্থা?	1 = কখনই বিবাহ করিনি<br>2 = বর্তমানে বিবাহিত<br>3 = পৃথক<br>4 = তালাকপ্রাপ্ত<br>5 = বিপত্নিক/বিধবা<br>88 = জানাতে অসম্মতি		উত্তর 1 হলে 405 এ যান
403	AGM_403	আপনি সারাজীবনে এই পর্যন্ত মোট কতবার গর্ভবতী হয়েছিলেন? (অ্যাবরশন, এম আর, মৃত সন্তান প্রশব, জীবিত সন্তান জন্মদান)	|____|____| বার		উত্তর “0” হলে পরিচ্ছেদ 5 এ যান
404	AGM_404	আপনি কি বর্তমানে গর্ভবতী	1 = হ্যাঁ 2 = না<br>8 = নিশ্চিত না		
মাসিক স্বাস্থ্যবিধি (যদি মাসিকের সময়কাল শুরু হয় তাহলে অনুগ্রহ করে নিচের প্রশ্নগুলো জিজ্ঞাসা করুন, যদি না হয় তাহলে পরিমাপ অংশে যান)					
405	AGM_405	মাসিকের সময় আপনি কি ব্যবহার করেন	1 = স্যানিটারি প্যাড<br>2 = পুরোনো কাপড়<br>3 = নতুন কাপড়<br>99 = অন্যান্য (নির্দিষ্ট করুন) ___________		

পরিচ্ছেদ 5: স্বাস্থ্য সেবা (যারা বিবাহিত তাদেরকেই এই প্রশ্নগুলো করতে হবে)

নম্বর	সূচক	প্রশ্ন	উত্তর	কোড	নির্দেশনা
501	AGH_501	গত 24 মাসের মধ্যে আপনি জীবিত বা মৃত কোন সন্তান জন্ম দিয়েছিলেন কি?	1 = হ্যাঁ 2 = না		যদি না হয় তাহলে পরিচ্ছেদ 6 এ যান
502	AGH_502	আপনি কত তারিখে জীবিত বা মৃত কোন সন্তান জন্ম দিয়েছিলেন?	|___|___|/|___|___|/20|___|___| দিন / মাস / বছর		
503	AGH_503	আপনার সন্তান কি এখন জীবিত আছে?	1 = হ্যাঁ 2 = না<br>88 = প্রযোজ্য নয়		
504	AGH_504	আপনার সন্তান যখন আপনার গর্ভে ছিল তখন কি গর্ভবতী হিসেবে আপনাকে নিবন্ধন করা হয়েছিল?	1 = হ্যাঁ 2 = না		
505	AGH_505	আপনার সন্তান যখন আপনার গর্ভে ছিল তখন আপনি কতবার গর্ভকালীন সেবা নিয়েছিলেন?	|____|____| বার		
506	AGH_506	আপনার সন্তান যখন আপনার গর্ভে ছিল তখন কি আপনি প্রধানত: কার কাছ থেকে গর্ভকালীন সেবা নিয়েছিলেন?<br><br>(একাধিক উত্তর হতে পারে)	1 = পাশ করা ডাক্তার<br>2 = নার্স/মিডওয়াইফ/ প্যারামেডিক<br>3 = এফ ডব্লিউ ভি (FWV)<br>4 = মেডিক্যাল এ্যাসিস্টেন্ট/ সাব এ্যাসিস্টেন্ট<br>5 = এনজিও স্বাস্থ্যকর্মী<br>6 = দক্ষ দাই<br>7 = প্রশিক্ষণপ্রাপ্ত সনাতন দাই<br>8 = সনাতন দাই<br>77 = জানিনা<br>99 = অন্যান্য (নির্দিষ্ট করুন)_____		
507	AGH_507	আপনার সন্তান যখন আপনার গর্ভে ছিল তখন কি আপনার ওজন মাপা হয়েছিল?	1 = হ্যাঁ 2 = না		

নম্বর	সূচক	প্রশ্ন	উত্তর	কোড	নির্দেশনা
508	AGH_508	আপনার সন্তান যখন আপনার গর্ভে ছিল তখন কি আপনি আয়রন ও ফলিক এসিড ট্যাবলেট খেয়েছিলেন?	1 = হ্যাঁ 2 = না		উত্তর না হলে 512 এ যান
509	AGH_509	আপনার সন্তান যখন আপনার গর্ভে ছিল তখন আপনি কোথা থেকে আয়রন ও ফলিক এসিড ট্যাবলেট পেয়েছিলেন?<br><br>(একাধিক উত্তর হতে পারে)	*সরকারী স্বাস্থ্যসেবা*<br>1 = সরকারী মাঠকর্মী<br>2 = সরকারী স্যাটেলাইট ক্লিনিক/অস্থায়ী টিকাদান (ইপিআই)কেন্দ্র<br>3 = সরকারী কমিউনিটি ক্লিনিক<br>4 = সরকারী স্বাস্থ্য ও পরিবার কল্যাণ কেন্দ্র (FWC)<br>5 = সরকারী উপজেলা স্বাস্থ্য কমপ্লেক্স<br>6 = শিশু ও মাতৃমঙ্গল কেন্দ্র<br>7 = সরকারী হাসপাতাল<br>*এনজিও স্বাস্থ্যসেবা*<br>8 = এনজিও স্বাস্থ্য ক্লিনিক ও হাসপাতাল<br>9 = এনজিও মাঠকর্মী<br>10 = কমিউনিটি নিউট্রিশন প্রোমোটার (সিএনপি)<br>*ব্যক্তিমালিকানাধীন স্বাস্থ্যসেবা*<br>11 = প্রাইভেট ডাক্তার/ক্লিনিক/ হাসপাতাল<br>12 = ফার্মেসী<br>13 = হাতুড়ে ডাক্তার/সনাতন চিকিৎসক<br>14 = হোমিওপ্যাথিক<br>77 = জানিনা<br>99 = অন্যান্য (নির্দিষ্ট করুন)______		
510	AGH_510	আপনার সন্তান যখন আপনার গর্ভে ছিল তখন আপনি কতমাস আয়রন ও ফলিক এসিড ট্যাবলেট খেয়েছিলেন?	|____|____| মাস		
511	AGH_511	আপনার সন্তান যখন আপনার গর্ভে ছিল তখন আপনি সপ্তাহে কতটি আয়রন ও ফলিক এসিড ট্যাবলেট খেয়েছিলেন?	|____|____| টি		
512	AGH_512	আপনার সন্তান যখন আপনার গর্ভে ছিল তখন কি আপনি ক্যালসিয়াম ট্যাবলেট খেয়েছিলেন?	1 = হ্যাঁ 2 = না		উত্তর না হলে 516 এ যান
513	AGH_513	আপনার সন্তান যখন আপনার গর্ভে ছিল তখন আপনি কোথা থেকে ক্যালসিয়াম ট্যাবলেট পেয়েছিলেন?<br><br>(একাধিক উত্তর হতে পারে)	*সরকারী স্বাস্থ্যসেবা*<br>1 = সরকারী মাঠকর্মী<br>2 = সরকারী স্যাটেলাইট ক্লিনিক/অস্থায়ী টিকাদান (ইপিআই)কেন্দ্র<br>3 = সরকারী কমিউনিটি ক্লিনিক<br>4 = সরকারী স্বাস্থ্য ও পরিবার কল্যাণ কেন্দ্র (FWC)<br>5 = সরকারী উপজেলা স্বাস্থ্য কমপ্লেক্স<br>6 = শিশু ও মাতৃমঙ্গল কেন্দ্র<br>7 = সরকারী হাসপাতাল<br>*এনজিও স্বাস্থ্যসেবা*<br>8 = এনজিও স্বাস্থ্য ক্লিনিক ও হাসপাতাল<br>9 = এনজিও মাঠকর্মী<br>10 = কমিউনিটি নিউট্রিশন প্রোমোটার (সিএনপি)<br>*ব্যক্তিমালিকানাধীন স্বাস্থ্যসেবা*<br>11 = প্রাইভেট ডাক্তার/ক্লিনিক/ হাসপাতাল<br>12 = ফার্মেসী<br>13 = হাতুড়ে ডাক্তার/সনাতন চিকিৎসক<br>14 = হোমিওপ্যাথিক<br>77 = জানিনা<br>99 = অন্যান্য (নির্দিষ্ট করুন)______		
514	AGH_514	আপনার সন্তান যখন আপনার গর্ভে ছিল তখন আপনি কতমাস ক্যালসিয়াম ট্যাবলেট খেয়েছিলেন?	|____|____| মাস		
515	AGH_515	আপনার সন্তান যখন আপনার গর্ভে ছিল তখন আপনি সপ্তাহে কতটি ক্যালসিয়াম ট্যাবলেট খেয়েছিলেন?	|____|____| টি		

নম্বর	সূচক	প্রশ্ন	উত্তর	কোড	নির্দেশনা
516	AGH_516	আপনার সন্তান যখন আপনার গর্ভে ছিল তখন কি ডাক্তার বা অন্য কোন স্বাস্থসেবাপ্রদানকারী আপনাকে কি পুষ্টি বিষয়ক কোন পরামর্শ দিয়েছিলেন?	1 = হ্যাঁ 2 = না		
517	AGH_517	কি কি বিষয়ের উপর পরামর্শ দিয়েছিলেন?	1 = পর্যাপ্ত খাবার গ্রহণ<br>2 = পর্যাপ্ত বিশ্রাম<br>3 = পুষ্টিকর খাবারের ধরণ<br>4 = আয়রন ও ফলিক এসিড ট্যাবলেট খাওয়ার নিয়ম<br>5 = ক্যালসিয়াম ট্যাবলেট খাওয়ার নিয়ম		
মৃত সন্তানের জন্ম হলে নিচের প্রশ্নগুলো করার প্রয়োজন নেই।					
518	AGH_518	জন্মের 3 দিনের মধ্যে আপনার সন্তানের জন্য ওজন নেয়া হয়েছিল কি?	1 = হ্যাঁ 2 = না<br>88 = প্রযোজ্য নয়		প্রযোজ্য নয় হলে পরবর্তী পরিচ্ছেদ যান
519	AGH_519	আপনার সন্তানের জন্ম ওজন কত ছিল?	|____|.|____|____|____| কেজি		
520	AGH_520	পরবর্তীতে কোন ডাক্তার বা অন্য কোন স্বাস্থসেবাপ্রদানকারী আপনার সন্তানের ওজন নিয়েছে কি?	1 = হ্যাঁ 2 = না<br>88 = প্রযোজ্য নয়		
521	AGH_521	আপনার সন্তানের বয়স অনুযায়ী ওজনের কোন চার্ট আপনার কাছে আছে কি?	1 = হ্যাঁ 2 = না		
522	AGH_522	আপনার সন্তান জন্মের পরে ডাক্তার বা অন্য কোন স্বাস্থসেবাপ্রদানকারী আপনাকে কি পুষ্টি বিষয়ক কোন পরামর্শ দিয়েছিলেন?	1 = হ্যাঁ 2 = না		
523	AGH_523	কি কি বিষয়ের উপর পরামর্শ দিয়েছিলেন?	1 = পর্যাপ্ত খাবার গ্রহণ<br>2 = পর্যাপ্ত বিশ্রাম<br>3 = পুষ্টিকর খাবারের ধরণ<br>4 = আয়রন ও ফলিক এসিড ট্যাবলেট খাওয়ার নিয়ম<br>5 = ক্যালসিয়াম ট্যাবলেট খাওয়ার নিয়ম<br>6 = প্রথম 6 মাস শুধুমাত্র বুকের দুধ খাওয়ানো<br>7 = 2 বছর বয়স পর্যন্ত বুকের দুধ খাওয়া চালিয়ে যাওয়া<br>8 = সম্পূরক খাবার খাওয়ানোর নিয়ম<br>9 = অসুস্থ অবস্থায় শিশুকে খাওয়ানোর নিয়ম<br>10 = দোকানের খাবার না খাওয়ানো		

অংশগ্রহণকারীর আইডি:|___|___|___|___|___|___|___|___|___|___|___|___|___|___|___|___|

পরিচ্ছেদ 6: মানসিক স্বাস্থ্য

নম্বর	সূচক	প্রশ্ন	উত্তর	কোড	নির্দেশনা
গত 2 সপ্তাহ ধরে আপনি কতটা ঘন ঘন নীচের সমস্যাগুলোর মুখোমুখি হয়েছেন?					
601	AGM_601	কোন কিছু করতে কম আগ্রহ বা কম আনন্দ	0 = একদমই নয়<br>1 = কোন কোন সময়<br>2 = বেশিরভাগ সময়<br>3 = সব সময়		
602	AGM_602	মন খারাপ, বিষন্ন, খিটখিটে, আশাহীন মনে হওয়া	0 = একদমই নয়<br>1 = কোন কোন সময়<br>2 = বেশিরভাগ সময়<br>3 = সব সময়		
603	AGM_603	ঘুম আসতে বা ঘুমিয়ে থাকতে অসুবিধা হওয়া বা অনেক বেশি ঘুমানো	0 = একদমই নয়<br>1 = কোন কোন সময়<br>2 = বেশিরভাগ সময়<br>3 = সব সময়		
604	AGM_604	ক্লান্ত লাগা বা অল্প শক্তি পাওয়া	0 = একদমই নয়<br>1 = কোন কোন সময়<br>2 = বেশিরভাগ সময়<br>3 = সব সময়		
605	AGM_605	খাবারে অরুচি, ওজন কমে যাওয়া বা বেশি বেশি খাওয়া	0 = একদমই নয়<br>1 = কোন কোন সময়<br>2 = বেশিরভাগ সময়<br>3 = সব সময়		
606	AGM_606	নিজের সম্পর্কে খারাপ অনুভূতি হওয়া, নিজেকে ব্যর্থ মনে করা, নিজেকে বা নিজের পরিবারকে ছোট করছি এমন মনে হওয়া	0 = একদমই নয়<br>1 = কোন কোন সময়<br>2 = বেশিরভাগ সময়<br>3 = সব সময়		
607	AGM_607	স্কুলের কাজ, কোন কিছু পড়া অথবা টিভি দেখার সময় মনোযোগ দিতে সমস্যা হওয়া	0 = একদমই নয়<br>1 = কোন কোন সময়<br>2 = বেশিরভাগ সময়<br>3 = সব সময়		
608	AGM_608	এত আস্তে কথা বলা বা চলাফেরা করা যা অন্যরা খেয়াল করতে পারে অথবা এর বিপরীত অর্থাৎ বেশি চঞ্চলতা বা স্বাভাবিকের চেয়ে বেশি নড়াচড়া করা	0 = একদমই নয়<br>1 = কোন কোন সময়<br>2 = বেশিরভাগ সময়<br>3 = সব সময়		
609	AGM_609	মরে গেলে বা নিজেকে কোনভাবে আঘাত করলে ভালো হবে এমন মনে হওয়া	0 = একদমই নয়<br>1 = কোন কোন সময়<br>2 = বেশিরভাগ সময়<br>3 = সব সময়		উত্তর 0 হলে, পরিচ্ছেদ 7 এ যান

রেফারেল (Referral)

নম্বর	সূচক	প্রশ্ন	উত্তর	কোড	নির্দেশনা
610	AGM_610	রেফার করা হয়েছে কি?	1 = হ্যাঁ 2 = না		উত্তর না হলে, পরিচ্ছেদ 7 এ যান
611	AGM_611	কোথায় রেফার করা হয়েছে	1 = উপজেলা স্বাস্থ্য কমপ্লেক্স<br>2 = জেলা হাসপাতাল<br>3 = মেডিকেল কলেজ হাসপাতাল<br>4 = ডাক্তারের চেম্বার<br>99 = অন্যান্য (নির্দিষ্ট করুন) _________		

অংশগ্রহণকারীর আইডি:|___|___|___|___|___|___|___|___|___|___|___|___|___|___|___|___|

খাদ্য নিরাপত্তা ও পুষ্টি বিষয়ক সার্ভিলেন্স

পরিচ্ছেদ 7: পরিমাপ

নম্বর	সূচক	প্রশ্ন	উত্তর	কোড	নির্দেশনা
701	AGA_701	পরিমাপ নেয়া শুরুর সময়	|___|___| : |___|___| ঘন্টা : মিনিট		24 ঘণ্টার ফরম্যাট ব্যবহার করুন
702	AGA_702	ওজন পরিমাপক যন্ত্রের আইডি	|___|___|		
703	AGA_703	দৈর্ঘ্য পরিমাপক যন্ত্রের আইডি	|___|___|		
704	AGA_704	ওজন নেয়ার সময় গায়ের কাপড়ের ধরন	1 = হালকা কাপড়<br>2 = একটু ভারী কাপড়<br>3 = ভারী কাপড়		
705	AGA_705	পরিমাপ গ্রহণকারীর নাম			
706	AGA_706	পরিমাপ গ্রহণকারীর কোড	|___|___|		

নম্বর	সূচক	পরিমাপের নাম	পরিমাপ 1	পরিমাপ 2	পরিমাপ 3
707	AGA_707 A/B/C	উচ্চতা (Cm)	A.|___|___|___|.|___|	B.|___|___|___|.|___|	C.|___|___|___|.|___| যদি A ও B এর মধ্যে পার্থক্য 0.5cm এর বেশি হয়, তাহলে ৩য় বার মাপুন
708	AGA_708 A/B/C	ওজন (Kg)	A.|___|___|___|.|___|	B.|__ _|___|___|.|___|	C.|___|___|___|. |___| যদি A ও B এর মধ্যে পার্থক্য 0.1Kg এর বেশি হয়, তাহলে ৩য় ওজন নিন
709	AGA_709 A/B/C	চর্বি (%)	A.|___|___|___|.|___|	B.|___|___|___|.|___|	C.|___|___|___|.|___| যদি A ও B এর মধ্যে পার্থক্য 0.5cm এর বেশি হয়, তাহলে ৩য় বার মাপুন
710	AGA_710 A/B/C	পানি (%)	A.|___|___|___|.|___|	B.|__ _|___|___|.|___|	C.|___|___|___|. |___| যদি A ও B এর মধ্যে পার্থক্য 0.1Kg এর বেশি হয়, তাহলে ৩য় ওজন নিন
711	AGA_711 A/B/C	কোমরের পরিধি (Cm)	A.|___|___|.|___|	B.|___|___|.|___|	C.|___|___|.|___| যদি A ও B এর মধ্যে পার্থক্য 0.5cm এর বেশি হয়, তাহলে ৩য় বার মাপুন
712	AGA_712	পরিমাপ সম্পর্কে তথ্য সংগ্রহকারীর মন্তব্য			
713	AGA_713	পরিমাপ গ্রহণের শেষের সময়	|___|___| : |___|___| ঘন্টা : মিনিট		24 ঘণ্টার ফরম্যাট ব্যবহার করুন

অংশগ্রহণকারীর আইডি:|___|___|___|___|___|___|___|___|___|___|___|___|___|___|___|___|

খাদ্য নিরাপত্তা ও পুষ্টি বিষয়ক সার্ভিলেন্স

মডিউল 6: প্রাপ্তবয়স্ক পুরুষের প্রশ্নাবলী

পরিচ্ছেদ 1: সাধারণ তথ্যাবলী

নম্বর	সূচক	প্রশ্ন	উত্তর	কোড	নির্দেশনা
101	AM_101	আপনার নাম (ডাক নামসহ)	______________________________		
102	AM_102	আপনার জন্ম তারিখ	|____|____|/|____|____|/19|____|____| দিন মাস বছর		
103	AM_103	আপনার বয়স (বয়স পূর্ণ বছরে লিখুন)	|____|____| বছর		
104	AM_104	আপনার বৈবাহিক অবস্থা?	1 = কখনই বিবাহ করিনি<br>2 = বর্তমানে বিবাহিত<br>3 = পৃথক<br>4 = তালাকপ্রাপ্ত<br>5 = বিপত্নিক/বিধবা<br>88 = জানাতে অসম্মতি		
105	AM_105	আপনি সর্বমোট কত বছর প্রাতিষ্ঠানিক শিক্ষা গ্রহণ করেছেন?<br><br>(প্রথম শ্রেণীর নিচে এবং উপানুষ্ঠিক শিক্ষা অন্তর্ভুক্ত হবে না)<br>***নির্দেশনাঃ***<br>*তথ্য প্রদানকারীর সর্বমোট কত বছর প্রাতিষ্ঠানিক শিক্ষা সম্পন্ন করেছেন তা লিপিবদ্ধ করুন। প্রাতিষ্ঠানিক শিক্ষা না থাকলে 00 লিখুন।*	|____|____| বছর<br>প্রাথমিক শিক্ষা/এবতেদায়ী = 5<br>মাধ্যমিক/দাখিল =10<br>উচ্চ মাধ্যমিক/ ডিপ্লোমা/আলিম= 12<br>স্নাতক/ফাজিল =16<br>স্নাতাকোত্তর/কামিল/দাওরা =18		
106	AM_106	গত 12 মাসে আপনার প্রধান পেশা কি ছিল?<br><br>যদি তথ্য প্রদানকারী গত 12 মাসে একাধিক পেশায় নিয়ুক্ত থাকেন তাহলে তিনি যে পেশাটিতে বেশী সময় ব্যয় করেছেন সেটিকে প্রধান পেশা হিসাবে বিবেচনা করুন এবং তা লিপিবদ্ধ করুন।	1 = কৃষিকাজ (ধান)<br>2 = কৃষিকাজ (ধান ছাড়া অন্যান্য)<br>3 = কৃষি দিনমজুর<br>4 = অদক্ষ দিনমজুর<br>5 = দক্ষ দিনমজুর<br>6 = রিক্সা/ ভ্যান/ ঠেলাগাড়ী/ বেবীট্যাক্সি ড্রাইভার/ নৌকার মাঝি<br>7 = জেলে<br>8 = চাকুরীজীবি<br>9 = পেশাজীবি<br>10 = ব্যবসায়ী<br>11 = ক্ষুদে ব্যবসায়ী<br>12 = গৃহপরিচারিকা<br>13 = জুমচাষী<br>14 = উপার্জন করে না<br>15 = হাঁস/ মুরগী পালন/ পশু পালন<br>16 = হস্তশিল্প<br>17 = শাক-সব্জি চাষ<br>18 = মৎস চাষ<br>19 = গৃহিনী<br>20 = ছাত্র/ছাত্রী<br>77 = জানিনা<br>66 = বয়স 6 বছরের কম<br>99 = অন্যান্য (নির্দিষ্ট করুন) ___		
107	AM_107	আপনার মা সর্বমোট কত বছর প্রাতিষ্ঠানিক শিক্ষা গ্রহণ করেছেন?<br><br>(প্রথম শ্রেণীর নিচে এবং উপানুষ্ঠিক শিক্ষা অন্তর্ভুক্ত হবে না)<br>***নির্দেশনাঃ*** *তথ্য প্রদানকারীর সর্বমোট কত বছর প্রাতিষ্ঠানিক শিক্ষা সম্পন্ন করেছেন তা লিপিবদ্ধ করুন। প্রাতিষ্ঠানিক শিক্ষা না থাকলে 00 লিখুন।*	|____|____| বছর<br>প্রাথমিক শিক্ষা/এবতেদায়ী = 5<br>মাধ্যমিক/দাখিল =10<br>উচ্চ মাধ্যমিক/ ডিপ্লোমা/আলিম= 12<br>স্নাতক/ফাজিল =16<br>স্নাতাকোত্তর/কামিল/দাওরা =18		

নম্বর	সূচক	প্রশ্ন	উত্তর		কোড	নির্দেশনা
108	AM_108	গত 12 মাসের মধ্যে আপনার মায়ের প্রধান পেশা কি ছিল?<br><br>যদি তথ্য প্রদানকারী গত 12 মাসে একাধিক পেশায় নিযুক্ত থাকেন তাহলে তিনি যে পেশাটিতে বেশী সময় ব্যয় করেছেন সেটিকে প্রধান পেশা হিসাবে বিবেচনা করুন এবং তা লিপিবদ্ধ করুন।	1 = কৃষিকাজ (ধান)<br>2 = কৃষিকাজ (ধান ছাড়া অন্যান্য)<br>3 = কৃষি দিনমজুর<br>4 = অদক্ষ দিনমজুর<br>5 = দক্ষ দিনমজুর<br>6 = রিক্সা/ ভ্যান/ ঠেলাগাড়ী/ বেবীট্যাক্সি ড্রাইভার/ নৌকার মাঝি<br>7 = জেলে<br>8 = চাকুরীজীবি<br>9 = পেশাজীবি<br>10 = ব্যবসায়ী	11 = ক্ষুদে ব্যবসায়ী<br>12 = গৃহপরিচারিকা<br>13 = জুমচাষী<br>14 = উপার্জন করে না<br>15 = হাঁস/ মুরগী পালন/ পশু পালন<br>16 = হস্তশিল্প<br>17 = শাক-সব্জি চাষ<br>18 = মৎস চাষ<br>19 = গৃহিনী<br>20 = ছাত্র/ছাত্রী<br>77 = জানিনা<br>66 = বয়স 6 বছরের কম<br>99 = অন্যান্য (নির্দিষ্ট করুন) ___		
109	AM_109	আপনার বাবা সর্বমোট কত বছর প্রাতিষ্ঠানিক শিক্ষা গ্রহণ করেছেন?<br>(প্রথম শ্রেণীর নিচে এবং উপানুষ্ঠিক শিক্ষা অন্তর্ভুক্ত হবে না)<br><br>***নির্দেশনাঃ***<br>*সর্বমোট কত বছর প্রাতিষ্ঠানিক শিক্ষা সম্পন্ন করেছেন তা লিপিবদ্ধ করুন। প্রাতিষ্ঠানিক শিক্ষা না থাকলে 00 লিখুন।*	|____|____| বছর<br><br>প্রাথমিক শিক্ষা/এবতেদায়ী = 5<br>মাধ্যমিক/দাখিল =10<br>উচ্চ মাধ্যমিক/ ডিপ্লোমা/আলিম= 12<br>স্নাতক/ফাজিল =16<br>স্নাতাকোত্তর/কামিল/দাওরা =18			
110	AM_110	গত 12 মাসের মধ্যে আপনার বাবার প্রধান পেশা কি ছিল?<br><br>***নির্দেশনাঃ***<br>*যদি তথ্য প্রদানকারী গত 12 মাসে একাধিক পেশায় নিযুক্ত থাকেন তাহলে তিনি যে পেশাটিতে বেশী সময় ব্যয় করেছেন সেটিকে প্রধান পেশা হিসাবে বিবেচনা করুন এবং তা লিপিবদ্ধ করুন।*	1 = কৃষিকাজ (ধান)<br>2 = কৃষিকাজ (ধান ছাড়া অন্যান্য)<br>3 = কৃষি দিনমজুর<br>4 = অদক্ষ দিনমজুর<br>5 = দক্ষ দিনমজুর<br>6 = রিক্সা/ ভ্যান/ ঠেলাগাড়ী/ বেবীট্যাক্সি ড্রাইভার/ নৌকার মাঝি<br>7 = জেলে<br>8 = চাকুরীজীবি<br>9 = পেশাজীবি<br>10 = ব্যবসায়ী	11 = ক্ষুদে ব্যবসায়ী<br>12 = গৃহপরিচারিকা<br>13 = জুমচাষী<br>14 = উপার্জন করে না<br>15 = হাঁস/ মুরগী পালন/ পশু পালন<br>16 = হস্তশিল্প<br>17 = শাক-সব্জি চাষ<br>18 = মৎস চাষ<br>19 = গৃহিনী<br>20 = ছাত্র/ছাত্রী<br>77 = জানিনা<br>66 = বয়স 6 বছরের কম<br>99 = অন্যান্য (নির্দিষ্ট করুন) ___		

পরিচ্ছেদ 2ঃ খাদ্য গ্রহণের বৈচিত্র্য

নম্বর	সূচক	প্রশ্ন		গতকাল দিনে বা রাতে আপনি নিম্নবর্ণিত খাবারগুলো খেয়েছেন কি? (A)	গত 7 দিনে আপনি নিম্নবর্ণিত খাবারগুলো কতদিন খেয়েছেন? (B)	গত 7 দিনে আপনি নিম্নবর্ণিত খাবারগুলো কতবার খেয়েছেন? (C)
এখন আমি আপনাকে গতকাল দিনে বা রাতে (গতকাল সকাল 6:00 টা থেকে আজ সকাল 6:00 টা পর্যন্ত) আপনি বাড়ীতে ও বাড়ীর বাইরে যা যা খেয়েছেন তা বর্ণণা করতে অনুরোধ করছি। দয়া করে সব ধরনের খাবার, পানীয় যা আপনি সকাল, দুপুর ও রাতের খাবারের সাথে খেয়েছেন বা নাস্তা/হালকা নাস্তা করেছেন সে সম্পর্কে বলুন। আপনি খাবার তৈরীর সময় কোন খাবার খেয়ে থাকলে তাও মনে করে আমাদেরকে বলুন। আপনি গতকাল সকালে যা খেয়েছেন তা দিয়েই শুরু করুন।<br>- আপনি সকালে ঘুম থেকে উঠে কী কী খেয়েছেন? আরো কিছু খেয়েছেন কি?<br>- সকালে আরো কী কী খেয়েছেন? আরো কিছু খেয়েছেন কি?<br>- দুপুরে কী কী খেয়েছেন? আরো কিছু খেয়েছেন কি?<br>- বিকালে কী কী খেয়েছেন? আরো কিছু খেয়েছেন কি?<br>- সন্ধ্যায় কী কী খেয়েছেন? আরো কিছু খেয়েছেন কি?<br>- রাতে কী কী খেয়েছেন? আরো কিছু খেয়েছেন কি?<br>A কলামের প্রশ্নগুলোর উত্তর প্রথমে রেকর্ড করুন। A কলামের প্রশ্নগুলোর উত্তর রেকর্ড করা শেষ হলে B/ C কলামের উত্তর নিন।						
		খাবার ধরণ	খাবার নমুনা			
201	AMD_201 A/B/C	শ্বেতসার জাতীয় (শস্য জাতীয় খাবার)	ভাত, আটা রুটি, গম, মুড়ি, ভুট্টা, খিচুড়ি, বার্লি, ওট, কিনোয়া, নুডলস, পাস্তা	1 = হ্যাঁ<br>2 = না	|____|____| দিন	|____|____| বার
202	AMD_202 A/B/C	শ্বেতসার জাতীয় (মূল, কন্দ, কলা)	গোলআলু, মিষ্টিআলু, সাগু, এরারুট, কাঁচকলা, শালগম, কাসাভা, কচু, কচুমুখী, পাকাকলা, শালুক	1 = হ্যাঁ<br>2 = না	|____|____| দিন	|____|____| বার
203	AMD_203 A/B/C	ডাল ও ডাল জাতীয় খাবার	ডাল, শিমের বীচি, মটর, সয়াবীন, টফু, হুমাস	1 = হ্যাঁ<br>2 = না	|____|____| দিন	|____|____| বার
204	AMD_204 A/B/C	বাদাম ও তৈলবীজ	চীনা বাদাম, পেস্তা, কাজু, অথবা যেকোন বাদাম, চিয়া সীড, তিল, তিসি, সূর্যমুখী বীজ, মিষ্টি কুমড়া বীজ	1 = হ্যাঁ<br>2 = না	|____|____| দিন	|____|____| বার
205	AMD_205 A/B/C	গাঢ় সবুজ পাতা জাতীয় শাক	সকল ধরনের পাতা জাতীয় শাক (পুঁই, কচু, কলমি), ব্রকলি	1 = হ্যাঁ<br>2 = না	|____|____| দিন	|____|____| বার
206	AMD_206 A/B/C	লাল/কমলা/ হলুদ সব্জি	মিষ্টিকুমড়া, গাজর, গাঢ় হলুদ বা কমলা মিষ্টি আলু, ও অন্যান্য লাল/ কমলা/হলুদ রঙের সব্জি	1 = হ্যাঁ<br>2 = না	|____|____| দিন	|____|____| বার
207	AMD_207 A/B/C	লাল/কমলা/ হলুদ ফলমূল	পাকা আম, পাকা পেঁপে ও অন্যান্য লাল/কমলা/ হলুদ ফলমূল	1 = হ্যাঁ<br>2 = না	|____|____| দিন	|____|____| বার
208	AMD_208 A/B/C	ভিটামিন সি- সমৃদ্ধ ফল	পেয়ারা, স্ট্রবেরী, লেবু, কমলালেবু, আঙ্গুর, আনারস, কাঁচা আম, আমলকি, কিউই, টমেটো	1 = হ্যাঁ<br>2 = না	|____|____| দিন	|____|____| বার
209	AMD_209 A/B/C	ভিটামিন সি- সমৃদ্ধ সব্জি	কাঁচা টমেটো, কাঁচা মরিচ, ব্রাসেলস স্প্রাউট, ফুলকপি, বাঁধাকপি	1 = হ্যাঁ<br>2 = না	|____|____| দিন	|____|____| বার

নম্বর	সূচক	প্রশ্ন		গতকাল দিনে বা রাতে আপনি নিম্নবর্ণিত খাবারগুলো খেয়েছেন কি? (A)	গত 7 দিনে আপনি নিম্নবর্ণিত খাবারগুলো কতদিন খেয়েছেন? (B)	গত 7 দিনে আপনি নিম্নবর্ণিত খাবারগুলো কতবার খেয়েছেন? (C)
210	AMD_210 A/B/C	অন্যান্য শাক সব্জি	শিম, অ্যাসপারাগাস, বিট, কচি বাঁশ, ফুলকপি, সেলারি, শসা, বেগুন, লেটুস, মাশরুম, মূলা, জুকিনি	1 = হ্যাঁ<br>2 = না	|____|____| দিন	|____|____| বার
211	AMD_211 A/B/C	অন্যান্য ফলমূল	আপেল, আভাকাডো, জাম, চেরী, পাকা কাঁঠাল	1 = হ্যাঁ<br>2 = না	|____|____| দিন	|____|____| বার
212	AMD_212 A/B/C	ডিম	হাঁস-মুরগী, অন্যান্য পাখী	1 = হ্যাঁ<br>2 = না	|____|____| দিন	|____|____| বার
213	AMD_213 A/B/C	অঙ্গ জাতীয় মাংস	গিলা, কলিজা, পাকস্থলী, হৃদপিণ্ড, কিডনি	1 = হ্যাঁ<br>2 = না	|____|____| দিন	|____|____| বার
214	AMD_214 A/B/C	মাংস	গরু, শুকর, বাছুর, মেষশাবক, ছাগল, মুরগী, হাঁস বা যেকোন প্রাণীর মাংস	1 = হ্যাঁ<br>2 = না	|____|____| দিন	|____|____| বার
215	AMD_215 A/B/C	ছোট মাছ	ছোট মাছের কাঁটা/হাড়সহ (কাঁচকি, মলা, ঢেলা, চাপিলা, বাতাসি, ছোট চিংড়ি, ছোট মাছের শুঁটকি)	1 = হ্যাঁ<br>2 = না	|____|____| দিন	|____|____| বার
216	AMD_216 A/B/C	বড় মাছ/সামুদ্রিক মাছ/ সামুদ্রিক খাবার	বড় মাছ, ঝিনুক, কাঁকড়া, অক্টোপাস, স্কুইড, হাঙর, বড় চিংড়ি, বড় মাছের শুঁটকি	1 = হ্যাঁ<br>2 = না	|____|____| দিন	|____|____| বার
217	AMD_217 A/B/C	দুধ ও দুগ্ধ জাতীয় খাবার	দুধ, পনির, দই এবং অন্যান্য দুগ্ধজাতীয় খাদ্য	1 = হ্যাঁ<br>2 = না	|____|____| দিন	|____|____| বার
218	AMD_218 A/B/C	পোকা ও অন্যান্য আমিষ জাতীয় খাবার	মাছের ডিম, পোকা, শামুক	1 = হ্যাঁ<br>2 = না	|____|____| দিন	|____|____| বার
219	AMD_219 A/B/C	তৈল জাতীয় খাবার	ঘি, মাখন, ক্রিম, সর, চর্বি, মার্জারিন, ম্যায়োনেজ, পাম অয়েল, উদ্ভিজ্জ তেল	1 = হ্যাঁ<br>2 = না	|____|____| দিন	|____|____| বার
220	AMD_220 A/B/C	চিনিযুক্ত এবং ভাজা খাবার	খাস্তা, চিপস ও অন্যান্য ভাজা খাবার, সিংগারা, সমোচা	1 = হ্যাঁ<br>2 = না	|____|____| দিন	|____|____| বার
221	AMD_221 A/B/C	মিষ্টি জাতীয় খাবার	চিনিযুক্ত খাবার, যেমন চকলেটস, ক্যান্ডিস, কুকিস/মিষ্টি বিস্কুট এবং কেকস, মিষ্টি পেষ্ট্রি বা আইসক্রিম, যে কোন মিষ্টি, মধু, হালুয়া, কনডেন্সড দুধ, তিলের খাজা	1 = হ্যাঁ<br>2 = না	|____|____| দিন	|____|____| বার
222	AMD_222 A/B/C	মিষ্টি পানীয়	চিনি যুক্ত চা, কোমল পানীয়, জুস, এনার্জি ড্রিংক, ইয়োগার্ট ড্রিংক, চকলেট ড্রিংক, হরলিকস, মলটোভা	1 = হ্যাঁ<br>2 = না	|____|____| দিন	|____|____| বার
223	AMD_223 A/B/C	অন্যান্য পানীয় এবং খাবার	চিনি ছাড়া চা, মদ, বিয়ার, আচার, সুপ, উপরের	1 = হ্যাঁ<br>2 = না	|____|____| দিন	|____|____| বার

নম্বর	সূচক	প্রশ্ন		গতকাল দিনে বা রাতে আপনি নিম্নবর্ণিত খাবারগুলো খেয়েছেন কি? (A)	গত 7 দিনে আপনি নিম্নবর্ণিত খাবারগুলো কতদিন খেয়েছেন? (B)	গত 7 দিনে আপনি নিম্নবর্ণিত খাবারগুলো কতবার খেয়েছেন? (C)
			তালিকাভুক্ত নয় এমন যেকোন খাবার			
224	AMD_224 A/B/C	অন্যান্য খাবার সুস্বাদু করার দ্রব্যাদি	মসলা, ধনে পাতা, সস, রসুন, কেচাপ, লেবুর রস, পুদিনা পাতা, পান, সুপারি, তামাক পাতা, জর্দা	1 = হ্যাঁ<br>2 = না	|____|____| দিন	|____|____| বার

নম্বর	সূচক	প্রশ্ন	উত্তর	কোড	নির্দেশনা
আমি আপনাকে পরবর্তীতে যে প্রশ্নগুলো জিজ্ঞাসা করতে যাচ্ছি তা হল সচরাচর আপনি যে সকল ফলমূল ও শাক-শব্জি খেয়ে থাকেন সে বিষয়ে। আমার কাছে ফল ও শাক-শব্জির কিছু ছবি আছে। প্রতিটি ছবি এক একটি প্রমান মাপের সমান। উত্তর দেওয়ার সময় সাধারন 1টি সপ্তাহের কথা চিন্তা করুন। (মাসে 1-2 বার হলে উত্তর 00 হবে)					
225	AMD_225	সচরাচর সপ্তাহের কত দিন আপনি ফল খান ?<br>(নমুনা কার্ড দেখান)<br>*নির্দেশনাঃ*<br>*তথ্য প্রদানকারীকে নমুনা কার্ডে প্রদর্শিত ফলগুলো দেখিয়ে চিন্তা করতে বলুন। এখানে প্যাকেটজাত ফলের জুস গ্রহণযোগ্য নয় তবে বাসায় ব্লেন্ড করা ফলের জুস গ্রহণযোগ্য। সপ্তাহ বলতে ধর্মীয় বা অন্য কোন বিশেষ উপলক্ষ্য ব্যতীত একটি স্বাভাবিক সপ্তাহ বুঝায়। মাসে 1-2 বার হলে 00 লিখুন।*	দিনের সংখ্যা |___|___| 77 = জানা নাই<br>(যদি 00 দিন হয়, তাহলে AMD_227-তে যান)		
226	AMD_226	সেই দিন গুলির একদিনে কতটুকু ফল খেয়েছেন? (নমুনা কার্ড ও বাটি দেখান)<br>*নির্দেশনাঃ*<br>*তথ্য প্রদানকারীকে যে কোন একদিনের কথা স্মরণ করতে বলুন এবং বাটি দেখিয়ে পরিমাপ করতে বলুন।*	সারভিং সংখ্যা<br>|___|___|.|___| 77.7 = জানা নাই		
227	AMD_227	সচরাচর সপ্তাহের কত দিন আপনি শাক-সব্জি খান? (নমুনা কার্ড দেখান)<br>*নির্দেশনাঃ*<br>*তথ্য প্রদানকারীকে নমুনা কার্ডে প্রদর্শিত শাক-সব্জিগুলো দেখিয়ে চিন্তা করতে বলুন। এখানে আলু শাক-সব্জি হিসাবে গণ্য হবে না। কাঁচা ও রান্না করা শাক-সব্জির প্রমান পরিমাপ আলাদা করে দেখান। মাছের সাথে রান্না করা সব্জির ক্ষেত্রে শুধু সব্জির পরিমান করতে হবে। সপ্তাহ বলতে ধর্মীয় বা অন্য কোন বিশেষ উপলক্ষ্য ব্যতীত একটি স্বাভাবিক সপ্তাহ বুঝায়। মাসে 1-2 বার হলে '00' হবে।*	দিনের সংখ্যা |___|___| 77 = জানা নাই (যদি '00' দিন হয়, তাহলে AMD_229-তে যান)		

অংশগ্রহণকারীর আইডি:|___|___|___|___|___|___|___|___|___|___|___|___|___|___|___|___|

খাদ্য নিরাপত্তা ও পুষ্টি বিষয়ক সার্ভিলেন্স

নম্বর	সূচক	প্রশ্ন	উত্তর	কোড	নির্দেশনা
228	AMD_228	সেই দিন গুলির একদিনে কতটুকু পরিমান শাক-সব্জি খেয়েছেন? (নমুনা কার্ড ও বাটি দেখান)<br>*নির্দেশনাঃ*<br>*তথ্য প্রদানকারীকে যে কোন একদিনের কথা স্মরণ করতে বলুন এবং বাটি দেখিয়ে পরিমাপ করতে বলুন।*	সারভিং সংখ্যা<br>|___|___|.|___| 77.7 = জানা নাই		
229	AMD_229	আপনি কি বর্তমানে কোন ধরণের ভিটামিন/খনিজ লবন আছে এমন কোন ট্যাবলেট, ক্যাপসুল, সিরাপ খান?	1 = হ্যাঁ<br>2 = না		যদি 'না' হয় তাহলে পরবর্তী পরিচ্ছেদে যান
230	AMD_230	ব্যবস্থাপত্র দেখে বা ঔষধ দেখে নাম লিখুন	A.____________________<br>B.____________________<br>C.____________________		ঔষধের গায়ে বড় করে যে না লেখা আছে সেই নাম লিখুন

পরিচ্ছেদ 3ঃ আচরণগত ঝুঁকির কারণ (ধুমপান/ধোঁয়াহীন তামাক এবং শারীরিক পরিশ্রম)

নম্বর	সূচক	প্রশ্ন	উত্তর	কোড	নির্দেশনা
**মূলঃ তামাকের ব্যবহার**					
এখন আমি আপনাকে তামাক ও তামাকজাত দ্রব্যের (যেমনঃ ধূমপান, ধোঁয়াবিহীন তামাকের) ব্যবহার সম্পর্কে কিছু প্রশ্ন জিজ্ঞাসা করব।					
301	AMR_301	আপনি কি বর্তমানে কোন প্রকার ধূমপান করেন?<br>(যেমন: সিগারেট, বিড়ি, হুক্কা, চুরুট, সিগার)<br>[নমুনা কার্ড দেখান]<br>*নির্দেশনাঃ*<br>*উত্তরদাতাকে নমুনা কার্ড দেখিয়ে চিন্তা করতে বলুন যে বর্তমানে তিনি কোন দ্রব্যটি ধূমপান/ ব্যবহার করছেন।*	1 = হ্যাঁ<br>2 = না<br>(যদি না হয়, তাহলে AMR_304 এ যান)		
302	AMR_302	আপনি কি বর্তমানে প্রতিদিন ধূমপান করেন?<br><br>*নির্দেশনাঃ*<br>*এই প্রশ্নটি যারা বর্তমানে ধুমপান করেন তাদের জন্য প্রযোজ্য।*<br>***প্রতিদিন অর্থঃ***<br>*প্রায় একমাস বা তার বেশী সময় ধরে প্রতিদিন অন্তত একবার ধূমপান করা কে বুঝায়। যদি এমন হয় যে তথ্য প্রদানকারী 25 দিন হল ধূমপান শুরু করেছে এবং এখনো চলছে, সেক্ষেত্রে প্রতিদিন হিসাবে গণ্য হবে।*	1 = হ্যাঁ<br>2 = না		
303	AMR_303	কত বছর বয়সে আপনি প্রথম ধূমপান শুরু করেন?	বয়স |___|___| 77 = জানা নাই		
304	AMR_304	আপনি কি বর্তমানে কোন প্রকার ধোঁয়াবিহীন তামাক দ্রব্য ব্যবহার করেন?<br>(যেমনঃ পানের সাথে জর্দা, শুধু জর্দা, সুপারির সাথে জর্দা, পানের সাথে সাদাপাতা, তামাকযুক্ত পানমশলা, চিবিয়ে খাওয়া সাদাপাতা, খৈনি, নস্যি, গুল, ইত্যাদি)<br>[নমুনা কার্ড দেখান]<br><br>*নির্দেশনাঃ*<br>*তথ্য প্রদানকারীকে ধোঁয়বিহীন তামাক যেমনঃ জর্দা, গুল, সাদাপাতা, খৈনি, নস্যি দ্রব্যগুলো কি বর্তমানে ব্যবহার করেন কিনা তা চিন্তা করে উত্তর দিতে বলুন। এক্ষেত্রে, শুধু পান সুপারী ও চুন প্রযোজ্য হবে না। যদি তথ্য প্রদানকারী পানের সাথে জর্দা বা শুধু জর্দা, পানের সাথে সাদাপাতা বা শুধু সাদাপাতা, পানের সাথে তামাক যুক্ত পান মশলা বা শুধু তামাক যুক্ত পান মশলা খান তাহলে ধোঁয়াবিহীন তামাক সেবন হিসাবে গণ্য হবে।*	1 = হ্যাঁ<br>2 = না<br>(যদি না হয়, তাহলে AMR_307 এ যান)		
305	AMR_305	আপনি কি বর্তমানে প্রতিদিন এই ধোঁয়াবিহীন তামাক দ্রব্য ব্যবহার করেন?<br>**প্রতিদিন অর্থঃ** প্রায় একমাস বা তার বেশী সময় ধরে প্রতিদিন অন্তঃত একটি ধোঁয়াবিহীন তামাক পণ্য ব্যবহার করা কে বুঝায়। *যদি এমন হয় যে তথ্য প্রদানকারী 25 দিন হল ধূমপান শুরু করেছে এবং এখনো চলছে, সেক্ষেত্রে প্রতিদিন হিসাবে গণ্য হবে।*	1 = হ্যাঁ<br>2 = না		
306	AMR_306	কত বছর বয়সে আপনি প্রথম ধোঁয়াবিহীন তামাক দ্রব্য গ্রহণ শুরু করেন?	বয়স |___|___| 77 = জানা নাই		
307	AMR_307	আপনার বাবা-মা বা অভিভাবকরা কোনও ধরনের তামাক ব্যবহার করেন?	1 = কেউ না<br>2 = আমার বাবা বা পুরুষ অভিভাবক<br>3 = আমার মা বা মহিলা অভিভাবক<br>4 = উভয়<br>5 = আমি জানি না<br>99 = অন্যান্য (নির্দিষ্ট করুন) _______		

অংশগ্রহণকারীর আইডি:|___|___|___|___|___|___|___|___|___|___|___|___|___|___|___|___|

খাদ্য নিরাপত্তা ও পুষ্টি বিষয়ক সার্ভিলেন্স

নম্বর	সূচক	প্রশ্ন	উত্তর	কোড	নির্দেশনা
**মূলঃ শাররীক পরিশ্রম সংক্রান্ত তথ্য**					
এর পর আমি আপনাকে সপ্তাহে আপনি বিভিন্ন ধরনের শারীরিক পরিশ্রমে যে সময় কাটান সে সম্পর্কিত কিছু প্রশ্ন করবো। আপনি নিজেকে শারীরিকভাবে সক্রিয় মনে না করলেও, অনুগ্রহ করে এই প্রশ্নগুলোর উত্তর দিন। প্রথমে আপনি কাজ করার জন্য যে সময় ব্যয় করেন সে সম্পর্কে ভাবুন। সেই কাজগুলোর কথা ভাবুন যে কাজগুলো আপনি টাকার বিনিময়ে বা বিনামূল্যে করে থাকনে। পড়াশুনা, প্রশিক্ষণ, গৃহস্থালীর কাজ, খাদ্যশস্যের চাষাবাদ, মাছ ধরা বা চাকুরী খোঁজা। এখানে অতিমাত্রার ভারী কাজ বলতে সেই কাজগুলোকে বুঝায় যে কাজগুলো করতে বেশী পরিমানে শারীরিক পরিশ্রমের প্রয়োজন হয় এবং কাজগুলো করার ফলে শ্বাস-প্রশ্বাস অথবা হৃদস্পন্দন অনেক বেড়ে যায় এবং মধ্যম মাত্রার কাজ বলতে সেই কাজগুলোকে বোঝায় যে কাজগুলো করতে মাঝারী পরিমানের শারীরিক পরিশ্রমের প্রয়োজন হয় এবং কাজগুলো করার ফলে শ্বাস-প্রশ্বাস অথবা হৃদস্পন্দন সামান্য বেড়ে যায়।<br>নির্দেশনাঃ উপরের ভূমিকাটি তথ্য প্রদানকারীকে পড়ে শুনান। এই অংশটি বাদ দেয়া যাবে না। তথ্য প্রদানকারীকে প্রথমে অবশ্যই তার দৈনন্দিন কাজগুলো সম্পর্কে চিন্তা করবে (পারিশ্রমিক ও পারিশ্রমিকবিহীন কাজ, গৃহস্থালীকাজ, খাদ্য উৎপাদন, খাওয়ার জন্য মাছ ধরা, কাজ খোঁজা, এক জায়গা থেকে অন্য জায়গায় যাওয়ার জন্য ব্যয়িত সময় এবং সবশেষে অবসর সময়ে ব্যয়িত সময়)					
**এখন আমি আপনার করা অতিমাত্রার ভারী কাজ সম্পর্কে জানতে চাইবো**					
দৈনন্দিন কাজের/ পেশাগত কাজের অংশ হিসেবে করা অতিমাত্রার ভারী কাজ					
308	AMR_308	আপনাকে কি দৈনন্দিন কাজের/পেশাগত কাজের অংশ হিসেবে শ্বাস প্রশ্বাস ও হৃদস্পন্দন অনেক বেড়ে যায় এমন কোন অতিমাত্রার ভারী কাজ/ অতিমাত্রার খেলাধুলা, শরীরচর্চা অথবা বিনোদন মূলক কাজ একনাগাড়ে কমপক্ষে 10 মিনিট ধরে করতে হয়? [অতিমাত্রার ভারী কাজ যেমন ভারী জিনিস বহন করা বা তোলা, মাটি কাটা, নির্মাণ কাজ, ধান কাটা, জাল দিয়ে মাছ ধরা ইত্যাদি, অতিমাত্রার খেলাধুলা, শরীরচর্চা অথবা বিনোদন মূলক কাজ: দৌড়ানো, কাবাডি, ফুটবল খেলা, দাড়িয়া বান্ধা, গোল্লাছুট, ইত্যাদি।]<br>[নমুনা কার্ড দেখান]<br>***নির্দেশনাঃ***<br>*তথ্যপ্রদানকারীকে শুধুমাত্র কর্মস্থলের 'ভারী কাজগুলো/ অবসর সময়ে ভারী কাজের কথা' সম্পর্কে চিন্তা করতে বলুন। ঐকাজগুলোই অতিমাত্রার ভারী কাজ হিসেবে গণ্য হবে যার ফলে শ্বাস-প্রশ্বাসের ও হৃদস্পন্দনের হার অতিমাত্রায় বৃদ্ধি পায়।*	1 = হ্যাঁ<br>2 = না<br>(যদি না হয়, তাহলে AMR_311 এ যান)		
309	AMR_309	আপনি দৈনন্দিন কাজের অংশ হিসেবে সপ্তাহে কয়দিন অতিমাত্রার ভারী কাজ / অতিমাত্রার খেলাধুলা, শরীরচর্চা অথবা বিনোদন মূলক কাজ করেন?<br>***নির্দেশনাঃ***<br>*সাধারন একটি সপ্তাহ হচ্ছে উত্তর দাতার একটি স্বাভাবিক সপ্তাহে যে কাজ করে। বৈধ উত্তর সীমা হচ্ছে 1-7 দিন।*	|___|___| দিন<br>77 = জানিনা<br>[জানিনা হলে AMR_311 এ যান]		
310	AMR_310	সাধারনত: আপনি দিনে কত সময় ধরে অতিমাত্রার ভারী কাজ/ অতিমাত্রার খেলাধুলা, শরীরচর্চা অথবা বিনোদন মূলক কাজ করেন?<br>***নির্দেশনাঃ***<br>*উত্তর দাতাকে তার কোন একটি দিনের কথা (যা সহজেই মনে আসে) চিন্তা করতে বলুন যে দিন তিনি কর্মক্ষেত্রে ভারী কাজে নিযুক্ত ছিলেন/যে দিন তিনি অবসর সময়ে ভারী শারীরিক পরিশ্রম করেছিলেন। উত্তর দাতা ঐ সকল ভারী কাজগুলোকেই মনে করবেন যেগুলো একটানা 10 মি বা তার অধিক সময় ধরে করা হয়েছে। অধিক বা অস্বাভাবিক (4 ঘন্টার অধিক) উত্তরগুলো যাচাই করুন।*	________<br>মিনিট		
**এখন আমি আপনার করা মাঝারি মাত্রার ভারী কাজ সম্পর্কে জানতে চাইবো।**					
দৈনন্দিন কাজের/পেশাগত কাজের বাইরে করা মাঝারী মাত্রার কাজ					

অংশগ্রহণকারীর আইডি:|___|___|___|___|___|___|___|___|___|___|___|___|___|___|___|___|

খাদ্য নিরাপত্তা ও পুষ্টি বিষয়ক সার্ভিলেন্স

নম্বর	সূচক	প্রশ্ন	উত্তর	কোড	নির্দেশনা
311	AMR_311	আপনাকে কি দৈনন্দিন কাজের/পেশাগত কাজের অংশ হিসেবে শ্বাসপ্রশ্বাস ও হৃদস্পন্দন সামান্য বেড়ে যায় এমন কোন মাঝারি মাত্রার কাজ/ মাঝারী মাত্রার খেলাধুলা, শরীরচর্চা অথবা বিনোদন মূলক কাজ একনাগাড়ে কমপক্ষে 10 মিনিট ধরে করতে হয়? যেমন, কাপড় ধোয়া, হালকা কিছু তোলা, ঝাড়ু দেওয়া, জানালা পরিষ্কার করা, রেদা বা কুন্ডুনি দিয়ে চাঁছার কাজ করা, মেঝে ঝাড়ু দেওয়া, মোছা বা পরিষ্কার করার কাজ ইত্যাদি/ দ্রুত হাঁটা, ট্রেড মিলে হাঁটা, সাইকেল চালনা, সাঁতার কাটা, ভলিবল, জগিং।<br>[নমুনা কার্ড দেখান]<br>*নির্দেশনাঃ*<br>*উত্তর দাতা কে শুধুমাত্র কর্মস্থলের / অবসর সময়ে মাঝারিমাত্রার কাজগুলো' সম্পর্কে চিন্তা করতে বলুন। ঐ কাজগুলোই মাঝারি মাত্রার কাজ হিসেবে গণ্য হবে যার ফলে শ্বাস-প্রশ্বাসের ও হৃদস্পন্দনের হার সামান্য বৃদ্ধি পায়।*	1 = হ্যাঁ<br>2 = না<br>(যদি না হয়, তাহলে AMR_314 এ যান)		
312	AMR_312	আপনি দৈনন্দিন কাজের অংশ হিসেবে সপ্তাহে কয়দিন মাঝারি মাত্রার কাজ/ মাঝারি মাত্রার খেলাধুলা, শরীরচর্চা অথবা বিনোদন মূলক কাজ করেন?<br><br>*নির্দেশনাঃ*<br>*সাধারন একটি সপ্তাহ হচ্ছে উত্তর দাতার একটি স্বাভাবিক সপ্তাহে যে কাজ করে। বৈধ উত্তরসীমা হচ্ছে 1-7 দিন।*	|___|___| দিন<br>77 = জানিনা<br>[জানিনা হলে AMR_314 এ যান]		
313	AMR_313	সাধারনত আপনি দিনে কত সময় ধরে মাঝারিমাত্রার কাজ/ মাঝারি মাত্রার খেলাধুলা, শরীরচর্চা অথবা বিনোদন মূলক কাজ করেন?<br><br>নির্দেশনাঃ<br>উত্তর দাতাকে তার কোন একটি দিনের কথা (যা সহজেই মনে আসে) চিন্তা করতে বলুন যে দিন তিনি কর্মক্ষেত্রে মাঝারিমাত্রার কাজে নিযুক্ত ছিলেন/ যে দিন তিনি অবসর সময়ে মাঝারি মাত্রার কাজে নিযুক্ত ছিলেন। উত্তরদাতা ঐ সকল মাঝারিমাত্রার কাজগুলোকে আমলে আনবেন যেগুলো একটানা *10*মি বা তার অধিক সময় ধরে করা হয়েছে। অধিক/অস্বাভাবিক (*4* ঘন্টার অধিক) উত্তরগুলো যাচাই করুন।	________<br>মিনিট		
**অবসর সময়ের কাজের ধরন**					
**পরবর্তী প্রশ্নগুলো আপনার বসে বা হেলান দিয়ে কাটানো সময় সম্পর্কিত, যা কর্মস্থলে, বাড়িতে, এক জায়গা থেকে অন্য জায়গায় গাড়ী, বাস বা ট্রেনে করে যাতায়াত অথবা বন্ধুদের সাথে আড্ডায়, পড়াশোনা, কার্ড খেলা অথবা টেলিভিশন দেখার ক্ষেত্রে প্রযোজ্য। তবে এখানে ঘুমিয়ে কাটানো সময় অন্তর্ভূক্ত হবে না।**					
314	AMR_314	সাধারণত: দিনে কতটুকু সময় আপনি বসে/ হেলান দিয়ে অতিবাহিত করেন?<br>*নির্দেশনাঃ*<br>*উত্তরদাতাকে কাজকরার সময়, অফিসে, পড়াশোনার সময়, টেলিভিশন দেখার সময়, কম্পিউটার ব্যবহারের সময়, রান্নাঘরে হাতের কাজ করার সময়, বিশ্রামের সময় কতক্ষণ বসে কাটান। এখানে উত্তরদাতার ঘুমানোর সময় বিবেচ্য হবে না।*	________<br>মিনিট		
315	AMR_315	সাধারণত: দিনে কতটুকু সময় আপনি টেলিভিশন দেখেন?	________<br>মিনিট		

পরিচ্ছেদ 4: দীর্ঘস্থায়ী রোগ

নম্বর	সূচক	প্রশ্ন	উত্তর	কোড	নির্দেশনা
401	MCD_401	কোন স্বাস্থ্যকর্মী কি আপনাকে কখনও বলেছে যে, আপনার উচ্চরক্তচাপ বা হাইপারটেনশন আছে?	1 = হ্যাঁ 2 = না		
402	MCD_402	কোন স্বাস্থ্যকর্মী কি আপনাকে কখনও বলেছেন যে, আপনার হার্টের অসুখ আছে?	1 = হ্যাঁ 2 = না		
403	MCD_403	কোন স্বাস্থ্যকর্মী কি আপনাকে কখনও বলেছেন যে, আপনার এ্যজমা/হাঁপানী/ব্রংকাইটিস বা শ্বাস কষ্টের রোগ আছে?	1 = হ্যাঁ 2 = না		
404	MCD_404	কোন স্বাস্থ্যকর্মী কি আপনাকে কখনও বলেছে যে, আপনার কিডনী বা বৃক্কের রোগ আছে?	1 = হ্যাঁ 2 = না		
405	MCD_405	কোন স্বাস্থ্যকর্মী কি আপনাকে কখনও বলেছে যে, আপনার ডায়াবেটিস আছে?	1 = হ্যাঁ 2 = না		
406	MCD_406	কোন স্বাস্থ্যকর্মী কি আপনাকে কখনও বলেছে যে, আপনার স্ট্রোক হয়েছে?	1 = হ্যাঁ 2 = না		
407	MCD_407	কোন স্বাস্থ্যকর্মী কি আপনাকে কখনও বলেছে যে, আপনার ক্যান্সার আছে?	1 = হ্যাঁ 2 = না		
408	MCD_408	কোন স্বাস্থ্যকর্মী কি আপনাকে কখনও বলেছে যে, আপনার মানসিক সমস্যা আছে?	1 = হ্যাঁ 2 = না		

পরিচ্ছেদ 5: রক্তচাপ পরিমাপ

নম্বর	সূচক	প্রশ্ন	উত্তর		কোড	নির্দেশনা
501	MBP_501	রক্তচাপ পরিমাপ নেয়া শুরুর সময়	|____|____| : |____|____| ঘন্টা : মিনিট		24 ঘন্টার ফরম্যাট ব্যবহার করুন	
**পরিমাপ নং-1** (পরিমাপ নেয়ার পূর্বে নিশ্চিত হোন যেন উত্তরদাতা কমপক্ষে 15 মিনিট বিশ্রামে থাকেন)						
**নম্বর**	**সূচক**	**পরিমাপের নাম**	**Systolic**	**Diastolic**	**পরিমাপের সময়**	
502	MBP_502 A/B/C	রক্তচাপ (mm of Hg)	A.|___|___|___|	B.|___|___|___|	C.|____|____|:|____|____| ঘন্টা : মিনিট	
**পরিমাপ নং-2** (দুইটি পরিমাপের মধ্যে 3 মিনিট বিরতি দিন)						
			**Systolic**	**Diastolic**	**পরিমাপের সময়**	
503	MBP_503 A/B/C	রক্তচাপ (mm of Hg)	A.|___|___|___|	B.|___|___|___|	C.|____|____|:|____|____| ঘন্টা : মিনিট	
**পরিমাপ-3** (যদি যে কোনটির ক্ষেত্রে 1ম ও 2য় পরিমাপের মধ্যে পার্থক্য 10 এর বেশি হয়, তাহলে ৩য় বার রক্তচাপ নিন)						
			**Systolic**	**Diastolic**	**পরিমাপের সময়**	
504	MBP_504 A/B/C	রক্তচাপ (mm of Hg)	A.|___|___|___|	B.|___|___|___|	C.|____|____|:|____|____| ঘন্টা : মিনিট	
505	MBP_505	রক্তচাপ পরিমাপ যন্ত্রের আইডি	|____|____|			
506	MBP_506	রক্তচাপ পরিমাপ গ্রহণকারীর নাম ও কোড	|____|____|			
507	MBP_507	রক্তচাপ পরিমাপ গ্রহণের শেষের সময়	|____|____| : |____|____| ঘন্টা : মিনিট		24 ঘন্টার ফরম্যাট ব্যবহার করুন	
**[যে কোন পরিমাপের সময়, Systolic BP ≥140 mm of Hg, Diastolic BP ≥90 mm of Hg-এর যে কোন একটি হলে অংশগ্রহণকারীকে নির্দিষ্ট স্বাস্থ্য কেন্দ্রে রেফার করুন ।]**						

রেফারেল (Referral)

অংশগ্রহণকারীর আইডি:|___|___|___|___|___|___|___|___|___|___|___|___|___|___|___|___|

খাদ্য নিরাপত্তা ও পুষ্টি বিষয়ক সার্ভিলেন্স

নম্বর	সূচক	প্রশ্ন	উত্তর	কোড	নির্দেশনা
508	MBP_508	রেফার করা হয়েছে কি?	1 = হ্যাঁ 2 = না		উত্তর না হলে, পরিচ্ছেদ 6 এ যান
509	MBP_509	কোথায় রেফার করা হয়েছে	1 = উপজেলা স্বাস্থ্য কমপ্লেক্স<br>2 = জেলা হাসপাতাল<br>3 = মেডিকেল কলেজ হাসপাতাল<br>4 = ডাক্তারের চেম্বার<br>99 = অন্যান্য (নির্দিষ্ট করুন) __________		

পরিচ্ছেদ 6: পরিমাপ

নম্বর	সূচক	প্রশ্ন	উত্তর	কোড	নির্দেশনা
601	AMA_601	পরিমাপ নেয়া শুরুর সময়	|____|____| : |____|____| ঘন্টা : মিনিট		24 ঘন্টার ফরম্যাট ব্যবহার করুন
602	AMA_602	ওজন পরিমাপক যন্ত্রের আইডি	|____|____|		
603	AMA_603	দৈর্ঘ্য পরিমাপক যন্ত্রের আইডি	|____|____|		
604	AMA_604	ওজন নেয়ার সময় গায়ের কাপড়ের ধরন	1 = হালকা কাপড়<br>2 = একটু ভারী কাপড়<br>3 = ভারী কাপড়		
605	AMA_605	পরিমাপ গ্রহণকারীর নাম			
606	AMA_606	পরিমাপ গ্রহণকারীর কোড	|____|____|		

নম্বর	সূচক	পরিমাপের নাম	পরিমাপ 1	পরিমাপ 2	পরিমাপ 3
607	AMA_607 A/B/C	উচ্চতা (Cm)	A.|____|____|____|.|____|	B.|____|____|____|.|____|	C.|____|____|____|.|____| যদি A ও B এর মধ্যে পার্থক্য 0.5cm এর বেশি হয়, তাহলে ৩য় বার মাপুন
608	AMA_608 A/B/C	ওজন (Kg)	A.|___|____|____|.|____|	B.|___|____|____|.|____|	C.|___|___|____|. |___| যদি A ও B এর মধ্যে পার্থক্য 0.1Kg এর বেশি হয়, তাহলে ৩য় ওজন নিন
609	AMA_609 A/B/C	চর্বি (%)	A.|____|____|____|.|____|	B.|____|____|____|.|____|	C.|____|____|____|.|____| যদি A ও B এর মধ্যে পার্থক্য 0.5cm এর বেশি হয়, তাহলে ৩য় বার মাপুন
610	AMA_610 A/B/C	পানি (%)	A.|___|____|____|.|____|	B.|__ _|____|____|.|____|	C.|____|____|____|. |____| যদি A ও B এর মধ্যে পার্থক্য 0.1Kg এর বেশি হয়, তাহলে ৩য় ওজন নিন
611	AMA_611 A/B/C	কোমরের পরিধি (Cm)	A.|____|____|.|____|	B.|____|____|.|____|	C.|____|____|.|____| যদি A ও B এর মধ্যে পার্থক্য 0.5cm এর বেশি হয়, তাহলে ৩য় বার মাপুন

নম্বর	সূচক	প্রশ্ন	উত্তর	নির্দেশনা
612	AMA_612	পরিমাপ সম্পর্কে তথ্য সংগ্রহকারীর মন্তব্য		
613	AMA_613	পরিমাপ গ্রহণের শেষের সময়	|____|____| : |____|____| ঘন্টা : মিনিট	24 ঘন্টার ফরম্যাট ব্যবহার করুন

অংশগ্রহণকারীর আইডি:|___|___|___|___|___|___|___|___|___|___|___|___|___|___|___|___|

খাদ্য নিরাপত্তা ও পুষ্টি বিষয়ক সার্ভিলেন্স

মডিউল 7: প্রাপ্তবয়স্ক মহিলার প্রশ্নপত্র

পরিচ্ছেদ 1: সাধারণ তথ্যাবলী

নম্বর	সূচক	প্রশ্ন	উত্তর	কোড	নির্দেশনা
101	AW_101	আপনার নাম (ডাক নামসহ)	________________________		
102	AW_102	আপনার বয়স	|____|____|/|____|____|/19|____|____| দিন মাস বছর		
103	AW_103	আপনার বয়স (বয়স পূর্ণবছরে লিখুন)	|____|____| বছর		
104	AW_104	আপনার বৈবাহিক অবস্থা?	1 = কখনই বিবাহ করিনি<br>2 = বর্তমানে বিবাহিত<br>3 = পৃথক<br>4 = তালাকপ্রাপ্ত<br>5 = বিপত্নিক/বিধবা<br>88 = জানাতে অসম্মতি		
105	AW_105	আপনি সর্বমোট কত বছর প্রাতিষ্ঠানিক শিক্ষা গ্রহণ করেছেন?<br><br>(প্রথম শ্রেণীর নিচে এবং উপানুষ্ঠিক শিক্ষা অন্তর্ভুক্ত হবে না)<br>***নির্দেশনাঃ***<br>*তথ্য প্রদানকারীর সর্বমোট কত বছর প্রাতিষ্ঠানিক শিক্ষা সম্পন্ন করেছেন তা লিপিবদ্ধ করুন। প্রাতিষ্ঠানিক শিক্ষা না থাকলে 00 লিখুন।*	|____|____| বছর<br><br>প্রাথমিক শিক্ষা/এবতেদায়ী = 5<br>মাধ্যমিক/দাখিল =10<br>উচ্চ মাধ্যমিক/ ডিপ্লোমা/আলিম= 12<br>স্নাতক/ফাজিল =16<br>স্নাতাকোত্তর/কামিল/দাওরা =18		
106	AW_106	গত 12 মাসে আপনার প্রধান পেশা কি ছিল?<br><br>যদি তথ্য প্রদানকারী গত 12 মাসে একাধিক পেশায় নিযুক্ত থাকেন তাহলে তিনি যে পেশাটিতে বেশী সময় ব্যয় করেছেন সেটিকে প্রধান পেশা হিসাবে বিবেচনা করুন এবং তা লিপিবদ্ধ করুন।	1 = কৃষিকাজ (ধান)<br>2 = কৃষিকাজ (ধান ছাড়া অন্যান্য)<br>3 = কৃষি দিনমজুর<br>4 = অদক্ষ দিনমজুর<br>5 = দক্ষ দিনমজুর<br>6 = রিক্সা/ ভ্যান/ ঠেলাগাড়ী/ বেবীট্যাক্সি ড্রাইভার/ নৌকার মাঝি<br>7 = জেলে<br>8 = চাকুরীজীবি<br>9 = পেশাজীবি<br>10 = ব্যবসায়ী<br>11 = ক্ষুদে ব্যবসায়ী<br>12 = গৃহপরিচারিকা<br>13 = জুমচাষী<br>14 = উপার্জন করে না<br>15 = হাঁস/ মুরগী পালন/ পশু পালন<br>16 = হস্তশিল্প<br>17 = শাক-সব্জি চাষ<br>18 = মৎস চাষ<br>19 = গৃহিনী<br>20 = ছাত্র/ছাত্রী<br>77 = জানিনা<br>66 = বয়স 6 বছরের কম<br>99 = অন্যান্য (নির্দিষ্ট করুন) __		
107	AW_107	আপনার মা সর্বমোট কত বছর প্রাতিষ্ঠানিক শিক্ষা গ্রহণ করেছেন?<br><br>(প্রথম শ্রেণীর নিচে এবং উপানুষ্ঠিক শিক্ষা অন্তর্ভুক্ত হবে না)<br>***নির্দেশনাঃ*** *তথ্য প্রদানকারীর সর্বমোট কত বছর প্রাতিষ্ঠানিক শিক্ষা সম্পন্ন করেছেন তা লিপিবদ্ধ করুন। প্রাতিষ্ঠানিক শিক্ষা না থাকলে 00 লিখুন।*	|____|____| বছর<br><br>প্রাথমিক শিক্ষা/এবতেদায়ী = 5<br>মাধ্যমিক/দাখিল =10<br>উচ্চ মাধ্যমিক/ ডিপ্লোমা/আলিম= 12<br>স্নাতক/ফাজিল =16<br>স্নাতাকোত্তর/কামিল/দাওরা =18		

নম্বর	সূচক	প্রশ্ন	উত্তর		কোড	নির্দেশনা
108	AW_108	গত 12 মাসের মধ্যে আপনার মায়ের প্রধান পেশা কি ছিল?<br><br>*যদি তথ্য প্রদানকারী গত 12 মাসে একাধিক পেশায় নিযুক্ত থাকেন তাহলে তিনি যে পেশাটিতে বেশী সময় ব্যয় করেছেন সেটিকে প্রধান পেশা হিসাবে বিবেচনা করুন এবং তা লিপিবদ্ধ করুন।*	1 = কৃষিকাজ (ধান)<br>2 = কৃষিকাজ (ধান ছাড়া অন্যান্য)<br>3 = কৃষি দিনমজুর<br>4 = অদক্ষ দিনমজুর<br>5 = দক্ষ দিনমজুর<br>6 = রিক্সা/ ভ্যান/ ঠেলাগাড়ী/ বেবীট্যাক্সি ড্রাইভার/ নৌকার মাঝি<br>7 = জেলে<br>8 = চাকুরীজীবি<br>9 = পেশাজীবি<br>10 = ব্যবসায়ী	11 = ক্ষুদে ব্যবসায়ী<br>12 = গৃহপরিচারিকা<br>13 = জুমচাষী<br>14 = উপার্জন করে না<br>15 = হাঁস/ মুরগী পালন/ পশু পালন<br>16 = হস্তশিল্প<br>17 = শাক-সব্জি চাষ<br>18 = মৎস চাষ<br>19 = গৃহিনী<br>20 = ছাত্র/ছাত্রী<br>77 = জানিনা<br>66 = বয়স 6 বছরের কম<br>99 = অন্যান্য (নির্দিষ্ট করুন) __		
109	AW_109	আপনার বাবা সর্বমোট কত বছর প্রাতিষ্ঠানিক শিক্ষা গ্রহণ (প্রথম শ্রেণীর নিচে এবং উপানুষ্ঠিক শিক্ষা অন্তর্ভুক্ত হবে না)<br><br>*নির্দেশনাঃ*<br>*সর্বমোট কত বছর প্রাতিষ্ঠানিক শিক্ষা সম্পন্ন করেছেন তা লিপিবদ্ধ করুন। প্রাতিষ্ঠানিক শিক্ষা না থাকলে 00 লিখুন।*	|____|____| বছর<br><br>প্রাথমিক শিক্ষা/এবতেদায়ী = 5<br>মাধ্যমিক/দাখিল =10<br>উচ্চ মাধ্যমিক/ ডিপ্লোমা/আলিম= 12<br>স্নাতক/ফাজিল =16<br>স্নাতাকোত্তর/কামিল/দাওরা =18			
110	AW_110	গত 12 মাসের মধ্যে আপনার বাবার প্রধান পেশা কি ছিল?<br><br>*নির্দেশনাঃ*<br>*যদি তথ্য প্রদানকারী গত 12 মাসে একাধিক পেশায় নিযুক্ত থাকেন তাহলে তিনি যে পেশাটিতে বেশী সময় ব্যয় করেছেন সেটিকে প্রধান পেশা হিসাবে বিবেচনা করুন এবং তা লিপিবদ্ধ করুন।*	1 = কৃষিকাজ (ধান)<br>2 = কৃষিকাজ (ধান ছাড়া অন্যান্য)<br>3 = কৃষি দিনমজুর<br>4 = অদক্ষ দিনমজুর<br>5 = দক্ষ দিনমজুর<br>6 = রিক্সা/ ভ্যান/ ঠেলাগাড়ী/ বেবীট্যাক্সি ড্রাইভার/ নৌকার মাঝি<br>7 = জেলে<br>8 = চাকুরীজীবি<br>9 = পেশাজীবি<br>10 = ব্যবসায়ী	11 = ক্ষুদে ব্যবসায়ী<br>12 = গৃহপরিচারিকা<br>13 = জুমচাষী<br>14 = উপার্জন করে না<br>15 = হাঁস/ মুরগী পালন/ পশু পালন<br>16 = হস্তশিল্প<br>17 = শাক-সব্জি চাষ<br>18 = মৎস চাষ<br>19 = গৃহিনী<br>20 = ছাত্র/ছাত্রী<br>77 = জানিনা<br>66 = বয়স 6 বছরের কম<br>99 = অন্যান্য (নির্দিষ্ট করুন) __		

অংশগ্রহণকারীর আইডি:|___|___|___|___|___|___|___|___|___|___|___|___|___|___|___|___|

খাদ্য নিরাপত্তা ও পুষ্টি বিষয়ক সার্ভিলেন্স

পরিচ্ছেদ 2ঃ খাদ্য গ্রহণের বৈচিত্র্য

নম্বর	সূচক	প্রশ্ন		গতকাল দিনে বা রাতে আপনি নিম্নবর্ণিত খাবারগুলো খেয়েছেন কি? (A)	গত 7 দিনে আপনি নিম্নবর্ণিত খাবারগুলো কতদিন খেয়েছেন? (B)	গত 7 দিনে আপনি নিম্নবর্ণিত খাবারগুলো কতবার খেয়েছেন? (C)
এখন আমি আপনাকে গতকাল দিনে বা রাতে (গতকাল সকাল 6:00 টা থেকে আজ সকাল 6:00 টা পর্যন্ত) আপনি বাড়ীতে ও বাড়ীর বাইরে যা যা খেয়েছেন তা বর্ণণা করতে অনুরোধ করছি। দয়া করে সব ধরনের খাবার, পানীয় যা আপনি সকাল, দুপুর ও রাতের খাবারের সাথে খেয়েছেন বা নাস্তা/হালকা নাস্তা করেছেন সে সম্পর্কে বলুন। আপনি খাবার তৈরীর সময় কোন খাবার খেয়ে থাকলে তাও মনে করে আমাদেরকে বলুন। আপনি গতকাল সকালে যা খেয়েছেন তা দিয়েই শুরু করুন।<br>- আপনি সকালে ঘুম থেকে উঠে কী কী খেয়েছেন? আরো কিছু খেয়েছেন কি?<br>- সকালে আরো কী কী খেয়েছেন? আরো কিছু খেয়েছেন কি?<br>- দুপুরে কী কী খেয়েছেন? আরো কিছু খেয়েছেন কি?<br>- বিকালে কী কী খেয়েছেন? আরো কিছু খেয়েছেন কি?<br>- সন্ধ্যায় কী কী খেয়েছেন? আরো কিছু খেয়েছেন কি?<br>- রাতে কী কী খেয়েছেন? আরো কিছু খেয়েছেন কি?<br>**A কলামের প্রশ্নগুলোর উত্তর প্রথমে রেকর্ড করুন। A কলামের প্রশ্নগুলোর উত্তর রেকর্ড করা শেষ হলে B/ C কলামের উত্তর নিন।**						
		খাবার ধরণ	খাবার নমুনা			
201	AWD_201 A/B/C	শ্বেতসার জাতীয় (শস্য জাতীয় খাবার)	ভাত, আটা রুটি, গম, মুড়ি, ভুট্টা, খিচুড়ি, বার্লি, ওট, কিনোয়া, নুডলস, পাস্তা	1 = হ্যাঁ<br>2 = না	|____|____| দিন	|____|____| বার
202	AWD_202 A/B/C	শ্বেতসার জাতীয় (মূল, কন্দ, কলা)	গোলআলু, মিষ্টিআলু, সাগু, এরারুট, কাঁচকলা, শালগম, কাসাভা, কচু, কচুমুখী, পাকাকলা, শালুক	1 = হ্যাঁ<br>2 = না	|____|____| দিন	|____|____| বার
203	AWD_203 A/B/C	ডাল ও ডাল জাতীয় খাবার	ডাল, শিমের বীচি, মটর, সয়বীন, টফু, হুমাস	1 = হ্যাঁ<br>2 = না	|____|____| দিন	|____|____| বার
204	AWD_204 A/B/C	বাদাম ও তৈলবীজ	চীনা বাদাম, পেস্তা, কাজু, অথবা যেকোন বাদাম, চিয়া সীড, তিল, তিসি, সূর্যমুখী বীজ, মিষ্টি কুমড়া বীজ	1 = হ্যাঁ<br>2 = না	|____|____| দিন	|____|____| বার
205	AWD_205 A/B/C	গাঢ় সবুজ পাতা জাতীয় শাক	সকল ধরনের পাতা জাতীয় শাক (পুঁই, কচু, কলমি), ব্রকলি	1 = হ্যাঁ<br>2 = না	|____|____| দিন	|____|____| বার
206	AWD_206 A/B/C	লাল/কমলা/ হলুদ সব্জি	মিষ্টিকুমড়া, গাজর, গাঢ় হলুদ বা কমলা মিষ্টি আলু, ও অন্যান্য লাল/ কমলা/হলুদ রঙের সব্জি	1 = হ্যাঁ<br>2 = না	|____|____| দিন	|____|____| বার
207	AWD_207 A/B/C	লাল/কমলা/ হলুদ ফলমূল	পাকা আম, পাকা পেঁপে ও অন্যান্য লাল/কমলা/ হলুদ ফলমূল	1 = হ্যাঁ<br>2 = না	|____|____| দিন	|____|____| বার
208	AWD_208 A/B/C	ভিটামিন সি- সমৃদ্ধ ফল	পেয়ারা, স্ট্রবেরী, লেবু, কমলালেবু, আঙ্গুর, আনারস, কাঁচা আম, আমলকি, কিউই, টমেটো	1 = হ্যাঁ<br>2 = না	|____|____| দিন	|____|____| বার
209	AWD_209 A/B/C	ভিটামিন সি- সমৃদ্ধ সব্জি	কাঁচা টমেটো, কাঁচা মরিচ, ব্রাসেলস স্প্রাউট, ফুলকপি, বাঁধাকপি	1 = হ্যাঁ<br>2 = না	|____|____| দিন	|____|____| বার

নম্বর	সূচক	প্রশ্ন		গতকাল দিনে বা রাতে আপনি নিম্নবর্ণিত খাবারগুলো খেয়েছেন কি? (A)	গত 7 দিনে আপনি নিম্নবর্ণিত খাবারগুলো কতদিন খেয়েছেন? (B)	গত 7 দিনে আপনি নিম্নবর্ণিত খাবারগুলো কতবার খেয়েছেন? (C)
210	AWD_210 A/B/C	অন্যান্য শাক সব্জি	শিম, অ্যাসপারাগাস, বিট, কচি বাঁশ, ফুলকপি, সেলারি, শসা, বেগুন, লেটুস, মাশরুম, মূলা, জুকিনি	1 = হ্যাঁ<br>2 = না	|___|___| দিন	|___|___| বার
211	AWD_211 A/B/C	অন্যান্য ফলমূল	আপেল, আভাকাডো, জাম, চেরী, পাকা কাঁঠাল	1 = হ্যাঁ<br>2 = না	|___|___| দিন	|___|___| বার
212	AWD_212 A/B/C	ডিম	হাঁস-মুরগী, অন্যান্য পাখী	1 = হ্যাঁ<br>2 = না	|___|___| দিন	|___|___| বার
213	AWD_213 A/B/C	অঙ্গ জাতীয় মাংস	গিলা, কলিজা, পাকস্থলী, হৃদপিণ্ড, কিডনি	1 = হ্যাঁ<br>2 = না	|___|___| দিন	|___|___| বার
214	AWD_214 A/B/C	মাংস	গরু, শুকর, বাছুর, মেষশাবক, ছাগল, মুরগী, হাঁস বা যেকোন প্রাণীর মাংস	1 = হ্যাঁ<br>2 = না	|___|___| দিন	|___|___| বার
215	AWD_215 A/B/C	ছোট মাছ	ছোট মাছের কাঁটা/হাড়সহ (কাঁচকি, মলা, ঢেলা, চাপিলা, বাতাসি, ছোট চিংড়ি, ছোট মাছের শুঁটকি)	1 = হ্যাঁ<br>2 = না	|___|___| দিন	|___|___| বার
216	AWD_216 A/B/C	বড় মাছ/সামুদ্রিক মাছ/ সামুদ্রিক খাবার	বড় মাছ, ঝিনুক, কাঁকড়া, অক্টোপাস, স্কুইড, হাঙর, বড় চিংড়ি, বড় মাছের শুঁটকি	1 = হ্যাঁ<br>2 = না	|___|___| দিন	|___|___| বার
217	AWD_217 A/B/C	দুধ ও দুগ্ধ জাতীয় খাবার	দুধ, পনির, দই এবং অন্যান্য দুগ্ধজাতীয় খাদ্য	1 = হ্যাঁ<br>2 = না	|___|___| দিন	|___|___| বার
218	AWD_218 A/B/C	পোকা ও অন্যান্য আমিষ জাতীয় খাবার	মাছের ডিম, পোকা, শামুক	1 = হ্যাঁ<br>2 = না	|___|___| দিন	|___|___| বার
219	AWD_219 A/B/C	তৈল জাতীয় খাবার	ঘি, মাখন, ক্রিম, সর, চর্বি, মার্জারিন, ম্যায়োনেজ, পাম অয়েল, উদ্ভিজ্জ তেল	1 = হ্যাঁ<br>2 = না	|___|___| দিন	|___|___| বার
220	AWD_220 A/B/C	চিনিযুক্ত এবং ভাজা খাবার	খাস্তা, চিপস ও অন্যান্য ভাজা খাবার, সিংগারা, সমোচা	1 = হ্যাঁ<br>2 = না	|___|___| দিন	|___|___| বার
221	AWD_221 A/B/C	মিষ্টি জাতীয় খাবার	চিনিযুক্ত খাবার, যেমন চকলেটস, ক্যান্ডিস, কুকিস/মিষ্টি বিস্কুট এবং কেকস, মিষ্টি পেষ্ট্রি বা আইসক্রিম, যে কোন মিষ্টি, মধু, হালুয়া, কনডেন্সড দুধ, তিলের খাজা	1 = হ্যাঁ<br>2 = না	|___|___| দিন	|___|___| বার
222	AWD_222 A/B/C	মিষ্টি পানীয়	চিনি যুক্ত চা, কোমল পানীয়, জুস, এনার্জি ড্রিংক, ইয়োগার্ট ড্রিংক, চকলেট ড্রিংক, হরলিকস, মলটোভা	1 = হ্যাঁ<br>2 = না	|___|___| দিন	|___|___| বার
223	AWD_223 A/B/C	অন্যান্য পানীয় এবং খাবার	চিনি ছাড়া চা, মদ, বিয়ার, আচার, সুপ, উপরের	1 = হ্যাঁ<br>2 = না	|___|___| দিন	|___|___| বার

অংশগ্রহণকারীর আইডি:|___|___|___|___|___|___|___|___|___|___|___|___|___|___|___|___|

নম্বর	সূচক	প্রশ্ন		গতকাল দিনে বা রাতে আপনি নিম্নবর্ণিত খাবারগুলো খেয়েছেন কি? (A)	গত 7 দিনে আপনি নিম্নবর্ণিত খাবারগুলো কতদিন খেয়েছেন? (B)	গত 7 দিনে আপনি নিম্নবর্ণিত খাবারগুলো কতবার খেয়েছেন? (C)
			তালিকাভুক্ত নয় এমন যেকোন খাবার			
224	AWD_224 A/B/C	অন্যান্য খাবার সুস্বাদু করার দ্রব্যাদি	মসলা, ধনে পাতা, সস, রসুন, কেচাপ, লেবুর রস, পুদিনা পাতা, পান, সুপারি, তামাক পাতা, জর্দা	1 = হ্যাঁ<br>2 = না	|_____|_____| দিন	|_____|_____| বার

নম্বর	সূচক	প্রশ্ন	উত্তর	কোড	নির্দেশনা
আমি আপনাকে পরবর্তীতে যে প্রশ্নগুলো জিজ্ঞাসা করতে যাচ্ছি তা হল সচরাচর আপনি যে সকল ফলমূল ও শাক-শজি খেয়ে থাকেন সে বিষয়ে। আমার কাছে ফল ও শাক-শজির কিছু ছবি আছে। প্রতিটি ছবি এক একটি প্রমান মাপের সমান। উত্তর দেওয়ার সময় সাধারন 1টি সপ্তাহের কথা চিন্তা করুন। (মাসে 1-2 বার হলে উত্তর 00 হবে)					
225	AWD_225	সচরাচর সপ্তাহের কত দিন আপনি ফল খান ? (নমুনা কার্ড দেখান)<br>***নির্দেশনাঃ***<br>*তথ্য প্রদানকারীকে নমুনা কার্ডে প্রদর্শিত ফলগুলো দেখিয়ে চিন্তা করতে বলুন। এখানে প্যাকেটজাত ফলের জুস গ্রহণযোগ্য নয় তবে বাসায় ব্লেন্ড করা ফলের জুস গ্রহণযোগ্য। সপ্তাহ বলতে ধর্মীয় বা অন্য কোন বিশেষ উপলক্ষ্য ব্যতীত একটি স্বাভাবিক সপ্তাহ বুঝায়। মাসে 1-2 বার হলে 00 লিখুন।*	দিনের সংখ্যা |___|___| 77 = জানা নাই<br>(যদি 00 দিন হয়, তাহলে AWD_227-তে যান)		
226	AWD_226	সেই দিন গুলির একদিনে কতটুকু ফল খেয়েছেন? (নমুনা কার্ড ও বাটি দেখান)<br>***নির্দেশনাঃ***<br>*তথ্য প্রদানকারীকে যে কোন একদিনের কথা স্মরণ করতে বলুন এবং বাটি দেখিয়ে পরিমাপ করতে বলুন।*	সারভিং সংখ্যা<br>|___|___|.|___| 77.7 = জানা নাই		
227	AWD_227	সচরাচর সপ্তাহের কত দিন আপনি শাক-সজি খান? (নমুনা কার্ড দেখান)<br>***নির্দেশনাঃ***<br>*তথ্য প্রদানকারীকে নমুনা কার্ডে প্রদর্শিত শাক-সজিগুলো দেখিয়ে চিন্তা করতে বলুন। এখানে আলু শাক-সজি হিসাবে গণ্য হবে না। কাঁচা ও রান্না করা শাক-সজির প্রমান পরিমাপ আলাদা করে দেখান। মাছের সাথে রান্না করা সজির ক্ষেত্রে শুধু সজির পরিমান করতে হবে। সপ্তাহ বলতে ধর্মীয় বা অন্য কোন বিশেষ উপলক্ষ্য ব্যতীত একটি স্বাভাবিক সপ্তাহ বুঝায়। মাসে 1-2 বার হলে '00' হবে।*	দিনের সংখ্যা |___|___| 77 = জানা নাই (যদি '00' দিন হয়, তাহলে AWD_229-তে যান)		
228	AWD_228	সেই দিন গুলির একদিনে কতটুকু পরিমান শাক-সজি খেয়েছেন? (নমুনা কার্ড ও বাটি দেখান)<br>***নির্দেশনাঃ***<br>*তথ্য প্রদানকারীকে যে কোন একদিনের কথা স্মরণ করতে বলুন এবং বাটি দেখিয়ে পরিমাপ করতে বলুন।*	সারভিং সংখ্যা<br>|___|___|.|___| 77.7 = জানা নাই		

নম্বর	সূচক	প্রশ্ন	উত্তর	কোড	নির্দেশনা
229	AWD_229	আপনি কি বর্তমানে কোন ধরণের ভিটামিন/খনিজ লবন আছে এমন কোন ট্যাবলেট, ক্যাপসুল, সিরাপ খান?	1 = হ্যাঁ<br>2 = না		যদি 'না' হয় তাহলে পরবর্তী পরিচ্ছেদে যান
230	AWD_230	ব্যবস্থাপত্র দেখে বা ঔষধ দেখে নাম লিখুন	A.____________________<br>B.____________________<br>C.____________________		

অংশগ্রহণকারীর আইডি:|___|___|___|___|___|___|___|___|___|___|___|___|___|___|___|___|

পরিচ্ছেদ 3ঃ আচরণগত ঝুঁকির কারণ (ধুমপান/ধোঁয়াহীন তামাক এবং শারীরিক পরিশ্রম)

নম্বর	সূচক	প্রশ্ন	উত্তর	কোড	নির্দেশনা
**মূলঃ তামাকের ব্যবহার**					
এখন আমি আপনাকে তামাক ও তামাকজাত দ্রব্যের (যেমনঃ ধূমপান, ধোঁয়াবিহীন তামাকের) ব্যবহার সম্পর্কে কিছু প্রশ্ন জিজ্ঞাসা করব।					
301	AWR_301	আপনি কি বর্তমানে কোন প্রকার ধূমপান করেন?<br>(যেমন: সিগারেট, বিড়ি, হুক্কা, চুরুট, সিগার)<br>[নমুনা কার্ড দেখান]<br>*নির্দেশনাঃ*<br>*উত্তরদাতাকে নমুনা কার্ড দেখিয়ে চিন্তা করতে বলুন যে বর্তমানে তিনি কোন দ্রব্যটি ধূমপান/ ব্যবহার করছেন।*	1 = হ্যাঁ<br>2 = না<br>(যদি না হয়, তাহলে AWR_304 এ যান)		
302	AWR_302	আপনি কি বর্তমানে প্রতিদিন ধূমপান করেন?<br><br>*নির্দেশনাঃ*<br>*এই প্রশ্নটি যারা বর্তমানে ধূমপান করেন তাদের জন্য প্রযোজ্য।*<br>***প্রতিদিন অর্থঃ***<br>*প্রায় একমাস বা তার বেশী সময় ধরে প্রতিদিন অন্তত একবার ধূমপান করা কে বুঝায়। যদি এমন হয় যে তথ্য প্রদানকারী 25 দিন হল ধূমপান শুরু করেছে এবং এখনো চলছে, সেক্ষেত্রে প্রতিদিন হিসাবে গণ্য হবে।*	1 = হ্যাঁ<br>2 = না		
303	AWR_303	কত বছর বয়সে আপনি প্রথম ধূমপান শুরু করেন?	বয়স |___|___| 77 = জানা নাই		
304	AWR_304	আপনি কি বর্তমানে কোন প্রকার ধোঁয়াবিহীন তামাক দ্রব্য ব্যবহার করেন?<br>(যেমনঃ পানের সাথে জর্দা, শুধু জর্দা, সুপারির সাথে জর্দা, পানের সাথে সাদাপাতা, তামাকযুক্ত পানমশলা, চিবিয়ে খাওয়া সাদাপাতা, খৈনি, নস্যি, গুল, ইত্যাদি)<br>[নমুনা কার্ড দেখান]<br><br>*নির্দেশনাঃ*<br>*তথ্য প্রদানকারীকে ধোঁয়বিহীন তামাক যেমনঃ জর্দা, গুল, সাদাপাতা, খৈনি, নস্যি দ্রব্যগুলো কি বর্তমানে ব্যবহার করেন কিনা তা চিন্তা করে উত্তর দিতে বলুন। এক্ষেত্রে, শুধু পান সুপারী ও চুন প্রযোজ্য হবে না। যদি তথ্য প্রদানকারী পানের সাথে জর্দা বা শুধু জর্দা, পানের সাথে সাদাপাতা বা শুধু সাদাপাতা, পানের সাথে তামাক যুক্ত পান মশলা বা শুধু তামাক যুক্ত পান মশলা খান তাহলে ধোঁয়াবিহীন তামাক সেবন হিসাবে গণ্য হবে।*	1 = হ্যাঁ<br>2 = না<br>(যদি না হয়, তাহলে AWR_307 এ যান)		
305	AWR_305	আপনি কি বর্তমানে প্রতিদিন এই ধোঁয়াবিহীন তামাক দ্রব্য ব্যবহার করেন?<br>**প্রতিদিন অর্থঃ** প্রায় একমাস বা তার বেশী সময় ধরে প্রতিদিন অন্তঃত একটি ধোঁয়াবিহীন তামাক পণ্য ব্যবহার করা কে বুঝায়। *যদি এমন হয় যে তথ্য প্রদানকারী 25 দিন হল ধূমপান শুরু করেছে এবং এখনো চলছে, সেক্ষেত্রে প্রতিদিন হিসাবে গণ্য হবে।*	1 = হ্যাঁ<br>2 = না		
306	AWR_306	কত বছর বয়সে আপনি প্রথম ধোঁয়াবিহীন তামাক দ্রব্য গ্রহণ শুরু করেন?	বয়স |___|___| 77 = জানা নাই		
307	AWR_307	আপনার বাবা-মা বা অভিভাবকরা কোনও ধরনের তামাক ব্যবহার করেন?	1 = কেউ না<br>2 = আমার বাবা বা পুরুষ অভিভাবক<br>3 = আমার মা বা মহিলা অভিভাবক<br>4 = উভয়<br>5 = আমি জানি না		

অংশগ্রহণকারীর আইডি:|___|___|___|___|___|___|___|___|___|___|___|___|___|___|___|___|

খাদ্য নিরাপত্তা ও পুষ্টি বিষয়ক সার্ভিলেন্স

নম্বর	সূচক	প্রশ্ন	উত্তর	কোড	নির্দেশনা
			99 = অন্যান্য (নির্দিষ্ট করুন) _______		
**মূলঃ শারীরিক পরিশ্রম সংক্রান্ত তথ্য**					
এর পর আমি আপনাকে সপ্তাহে আপনি বিভিন্ন ধরনের শারীরিক পরিশ্রমে যে সময় কাটান সে সম্পর্কিত কিছু প্রশ্ন করবো। আপনি নিজেকে শারীরিকভাবে সক্রিয় মনে না করলেও, অনুগ্রহ করে এই প্রশ্নগুলোর উত্তর দিন। প্রথমে আপনি কাজ করার জন্য যে সময় ব্যয় করেন সে সম্পর্কে ভাবুন। সেই কাজগুলোর কথা ভাবুন যে কাজগুলো আপনি টাকার বিনিময়ে বা বিনামূল্যে করে থাকনে। পড়াশুনা, প্রশিক্ষণ, গৃহস্থালীর কাজ, খাদ্যশস্যের চাষাবাদ, মাছ ধরা বা চাকুরী খোঁজা। এখানে অতিমাত্রার ভারী কাজ বলতে সেই কাজগুলোকে বুঝায় যে কাজগুলো করতে বেশী পরিমানে শারীরিক পরিশ্রমের প্রয়োজন হয় এবং কাজগুলো করার ফলে শ্বাস-প্রশ্বাস অথবা হৃদস্পন্দন অনেক বেড়ে যায় এবং মধ্যম মাত্রার কাজ বলতে সেই কাজগুলোকে বোঝায় যে কাজগুলো করতে মাঝারী পরিমানের শারীরিক পরিশ্রমের প্রয়োজন হয় এবং কাজগুলো করার ফলে শ্বাস-প্রশ্বাস অথবা হৃদস্পন্দন সামান্য বেড়ে যায়।<br>নির্দেশনাঃ উপরের ভূমিকাটি তথ্য প্রদানকারীকে পড়ে শুনান। এই অংশটি বাদ দেয়া যাবে না। তথ্য প্রদানকারীকে প্রথমে অবশ্যই তার দৈনন্দিন কাজগুলো সম্পর্কে চিন্তা করবে (পারিশ্রমিক ও পারিশ্রমিকবিহীন কাজ, গৃহস্থালীকাজ, খাদ্য উৎপাদন, খাওয়ার জন্য মাছ ধরা, কাজ খোঁজা, এক জায়গা থেকে অন্য জায়গায় যাওয়ার জন্য ব্যয়িত সময় এবং সবশেষে অবসর সময়ে ব্যয়িত সময়)					
**এখন আমি আপনার করা অতিমাত্রার ভারী কাজ সম্পর্কে জানতে চাইবো**					
দৈনন্দিন কাজের/ পেশাগত কাজের অংশ হিসেবে করা অতিমাত্রার ভারী কাজ					
308	AWR_308	আপনাকে কি দৈনন্দিন কাজের/পেশাগত কাজের অংশ হিসেবে শ্বাস প্রশ্বাস ও হৃদস্পন্দন অনেক বেড়ে যায় এমন কোন অতিমাত্রার ভারী কাজ/ অতিমাত্রার খেলাধুলা, শরীরচর্চা অথবা বিনোদন মূলক কাজ একনাগাড়ে কমপক্ষে 10 মিনিট ধরে করতে হয়? [অতিমাত্রার ভারী কাজ যেমন ভারী জিনিস বহন করা বা তোলা, মাটি কাটা, নির্মাণ কাজ, ধান কাটা, জাল দিয়ে মাছ ধরা ইত্যাদি, অতিমাত্রার খেলাধুলা, শরীরচর্চা অথবা বিনোদন মূলক কাজ: দৌড়ানো, কাবাডি, ফুটবল খেলা, দাড়িয়া বান্ধা, গোল্লাছুট, ইত্যাদি।]<br>[নমুনা কার্ড দেখান]<br>*নির্দেশনাঃ*<br>*তথ্যপ্রদানকারীকে শুধুমাত্র কর্মস্থলের 'ভারী কাজগুলো/ অবসর সময়ে ভারী কাজের কথা' সম্পর্কে চিন্তা করতে বলুন। ঐকাজগুলোই অতিমাত্রার ভারী উকাজ হিসেবে গণ্য হবে যার ফলে শ্বাস-প্রশ্বাসের ও হৃদস্পন্দনের হার অতিমাত্রায় বৃদ্ধি পায়।*	1 = হ্যাঁ<br>2 = না<br>(যদি না হয়, তাহলে AWR_311 এ যান)		
309	AWR_309	আপনি দৈনন্দিন কাজের অংশ হিসেবে সপ্তাহে কয়দিন অতিমাত্রার ভারী কাজ / অতিমাত্রার খেলাধুলা, শরীরচর্চা অথবা বিনোদন মূলক কাজ করেন?<br>*নির্দেশনাঃ*<br>*সাধারন একটি সপ্তাহ হচ্ছে উত্তর দাতার একটি স্বাভাবিক সপ্তাহে যে কাজ করে। বৈধ উত্তর সীমা হচ্ছে 1-7 দিন।*	|___|___| দিন<br>77 = জানিনা<br>[জানিনা হলে AWR_311 এ যান]		
310	AWR_310	সাধারনত: আপনি দিনে কত সময় ধরে অতিমাত্রার ভারী কাজ/ অতিমাত্রার খেলাধুলা, শরীরচর্চা অথবা বিনোদন মূলক কাজ করেন?<br>*নির্দেশনাঃ*<br>*উত্তর দাতাকে তার কোন একটি দিনের কথা (যা সহজেই মনে আসে) চিন্তা করতে বলুন যে দিন তিনি কর্মক্ষেত্রে ভারী কাজে নিযুক্ত ছিলেন/যে দিন তিনি অবসর সময়ে ভারী শারীরিক পরিশ্রম করেছিলেন। উত্তর দাতা ঐ সকল ভারী কাজগুলোকেই মনে করবেন যেগুলো একটানা 10 মি বা তার অধিক সময় ধরে করা হয়েছে। অধিক বা অস্বাভাবিক (4 ঘন্টার অধিক) উত্তরগুলো যাচাই করুন।*	________<br>মিনিট		
**এখন আমি আপনার করা মাঝারি মাত্রার ভারী কাজ সম্পর্কে জানতে চাইবো।**					
দৈনন্দিন কাজের/পেশাগত কাজের বাইরে করা মাঝারী মাত্রার কাজ					

নম্বর	সূচক	প্রশ্ন	উত্তর	কোড	নির্দেশনা
311	AWR_311	আপনাকে কি দৈনন্দিন কাজের/পেশাগত কাজের অংশ হিসেবে শ্বাসপ্রশ্বাস ও হৃদস্পন্দন সামান্য বেড়ে যায় এমন কোন মাঝারি মাত্রার কাজ/ মাঝারী মাত্রার খেলাধুলা, শরীরচর্চা অথবা বিনোদন মূলক কাজ একনাগাড়ে কমপক্ষে 10 মিনিট ধরে করতে হয়? যেমন, কাপড় ধোয়া, হালকা কিছু তোলা, ঝাড়ু দেওয়া, জানালা পরিষ্কার করা, রেদা বা কুড়ুনি দিয়ে চাঁছার কাজ করা, মেঝে ঝাড়ু দেওয়া, মোছা বা পরিষ্কার করার কাজ ইত্যাদি/ দ্রুত হাঁটা, ট্রেড মিলে হাঁটা, সাইকেল চালনা, সাঁতার কাটা, ভলিবল, জগিং। [নমুনা কার্ড দেখান]<br>***নির্দেশনাঃ***<br>*উত্তর দাতা কে শুধুমাত্র কর্মস্থলের / অবসর সময়ে মাঝারিমাত্রার কাজগুলো' সম্পর্কে চিন্তা করতে বলুন। ঐ কাজগুলোই মাঝারি মাত্রার কাজ হিসেবে গণ্য হবে যার ফলে শ্বাস-প্রশ্বাসের ও হৃদস্পন্দনের হার সামান্য বৃদ্ধি পায়।*	1 = হ্যাঁ<br>2 = না<br>(যদি না হয়, তাহলে AWR_314 এ যান)		
312	AWR_312	আপনি দৈনন্দিন কাজের অংশ হিসেবে সপ্তাহে কয়দিন মাঝারি মাত্রার কাজ/ মাঝারি মাত্রার খেলাধুলা, শরীরচর্চা অথবা বিনোদন মূলক কাজ করেন?<br><br>***নির্দেশনাঃ***<br>*সাধারন একটি সপ্তাহ হচ্ছে উত্তর দাতার একটি স্বাভাবিক সপ্তাহে যে কাজ করে। বৈধ উত্তরসীমা হচ্ছে 1-7 দিন।*	|___|___| দিন<br>77 = জানিনা<br>[জানিনা হলে AWR_314 এ যান]		
313	AWR_313	সাধারনত আপনি দিনে কত সময় ধরে মাঝারিমাত্রার কাজ/ মাঝারি মাত্রার খেলাধুলা, শরীরচর্চা অথবা বিনোদন মূলক কাজ করেন?<br><br>নির্দেশনাঃ<br>উত্তর দাতাকে তার কোন একটি দিনের কথা (যা সহজেই মনে আসে) চিন্তা করতে বলুন যে দিন তিনি কর্মক্ষেত্রে মাঝারিমাত্রার কাজে নিযুক্ত ছিলেন/ যে দিন তিনি অবসর সময়ে মাঝারি মাত্রার কাজে নিযুক্ত ছিলেন। উত্তরদাতা ঐ সকল মাঝারিমাত্রার কাজগুলোকে আমলে আনবেন যেগুলো একটানা *10*মি বা তার অধিক সময় ধরে করা হয়েছে। অধিক/অস্বাভাবিক (*4* ঘন্টার অধিক) উত্তরগুলো যাচাই করুন।	________<br>মিনিট		
**অবসর সময়ের কাজের ধরন**					
**পরবর্তী প্রশ্নগুলো আপনার বসে বা হেলান দিয়ে কাটানো সময় সম্পর্কিত, যা কর্মস্থলে, বাড়িতে, এক জায়গা থেকে অন্য জায়গায় গাড়ী, বাস বা ট্রেনে করে যাতায়াত অথবা বন্ধুদের সাথে আড্ডায়, পড়াশোনা, কার্ড খেলা অথবা টেলিভিশন দেখার ক্ষেত্রে প্রযোজ্য। তবে এখানে ঘুমিয়ে কাটানো সময় অন্তর্ভূক্ত হবে না।**					
314	AWR_314	সাধারণত: দিনে কতটুকু সময় আপনি বসে/ হেলান দিয়ে অতিবাহিত করেন?<br>***নির্দেশনাঃ***<br>*উত্তরদাতাকে কাজকরার সময়, অফিসে, পড়াশোনার সময়, টেলিভিশন দেখার সময়, কম্পিউটার ব্যবহারের সময়, রান্নাঘরে হাতের কাজ করার সময়, বিশ্রামের সময় কতক্ষণ বসে কাটান। এখানে উত্তরদাতার ঘুমানোর সময় বিবেচ্য হবে না।*	________<br>মিনিট		

অংশগ্রহণকারীর আইডি:|___|___|___|___|___|___|___|___|___|___|___|___|___|___|___|___|

খাদ্য নিরাপত্তা ও পুষ্টি বিষয়ক সার্ভিলেন্স

নম্বর	সূচক	প্রশ্ন	উত্তর	কোড	নির্দেশনা
315	AWR_315	সাধারণত: দিনে কতটুকু সময় আপনি টেলিভিশন দেখেন?	________ মিনিট		

পরিচ্ছেদ 4: দীর্ঘস্থায়ী রোগ

নম্বর	সূচক	প্রশ্ন	উত্তর	কোড	নির্দেশনা
401	WCD_401	কোন স্বাস্থ্যকর্মী কি আপনাকে কখনও বলেছেন যে, আপনার উচ্চরক্তচাপ বা হাইপারটেনশন আছে (গর্ভবতী থাকার অবস্থায় হলে এবং পরবর্তীতে ভালো হয়ে গেলে গণ্য হবেনা)?	1 = হ্যাঁ 2 = না		
402	WCD_402	কোন স্বাস্থ্যকর্মী কি আপনাকে কখনও বলেছেন যে, আপনার হার্টের অসুখ আছে?	1 = হ্যাঁ 2 = না		
403	WCD_403	কোন স্বাস্থ্যকর্মী কি আপনাকে কখনও বলেছেন যে, আপনার এ্যজমা/হাঁপানী/ব্রংকাইটিস বা শ্বাস কষ্টের রোগ আছে?	1 = হ্যাঁ 2 = না		
404	WCD_404	কোন স্বাস্থ্যকর্মী কি আপনাকে কখনও বলেছেন যে, আপনার কিডনী বা বৃক্কের রোগ আছে?	1 = হ্যাঁ 2 = না		
405	WCD_405	কোন স্বাস্থ্যকর্মী কি আপনাকে কখনও বলেছেন যে, আপনার ডায়াবেটিস আছে?	1 = হ্যাঁ 2 = না		
406	WCD_406	কোন স্বাস্থ্যকর্মী কি আপনাকে কখনও বলেছেন যে, আপনার স্ট্রোক হয়েছে?	1 = হ্যাঁ 2 = না		
407	WCD_407	কোন স্বাস্থ্যকর্মী কি আপনাকে কখনও বলেছেন যে, আপনার কোন ধরনের ক্যান্সার আছে?	1 = হ্যাঁ 2 = না		
408	WCD_408	কোন স্বাস্থ্যকর্মী কি আপনাকে কখনও বলেছেন যে, আপনার কোন মানসিক সমস্যা/রোগ আছে?	1 = হ্যাঁ 2 = না		

পরিচ্ছেদ 5: প্রজনন ইতিহাস, মাসিক

এখন আমি আপনাকে প্রজনন ইতিহাস ও মাসিক সম্পর্কে প্রশ্ন করব। তথ্যের গোপনীয়তার জন্য আপনাকে নিশ্চিয়তা দিচ্ছি। আমি কি শুরু করতে পারি?

নম্বর	সূচক	প্রশ্ন	উত্তর	কোড	নির্দেশনা
501	AWM_501	কোন বয়সে আপনার মাসিক শুরু হয়েছিল?	|____|____|বছর		এখনও শুরু না হয়ে থাকলে "00" লিখুন এবং পরবর্তী পরিচ্ছেদে যান
502	AWM_502	মাসিকের সময় আপনি কি কি সামগ্রী ব্যবহার করেন?	1 = স্যানেটারী নেপকিন<br>2 = পুরাতন কাপড়<br>3 = নতুন কাপড়<br>99 = অন্যান্য (নির্দ্দিষ্ট করুন)_____		
503	AWM_503	সারা জীবনে আপনি কতবার গর্ভবর্তী হয়েছেন? (গর্ভপাত, মাসিক নিয়মিতকরন, মৃতজন্ম, জীবিত জন্মসহ)	|____|____| সংখ্যা		উত্তর "0" হলে 505 নং প্রশ্নে যান
504	AWM_504	সারা জীবনে আপনি কতবার জীবিত শিশুর জন্ম দিয়েছেন?	|____|____| সংখ্যা		
505	AWM_505	বর্তমানে কি আপনার মাসিক হচ্ছে?	1 = হ্যাঁ 2 = না		উত্তর হ্যাঁ হলে 507 নং প্রশ্নে যান
506	AWM_506	আপনার মাসিক না হওয়ার কারন কি?	1 = আমি গর্ভবতী<br>2 = আমি গর্ভবতী হতে পারি<br>3 = আমার মাসিক বন্ধ হয়ে গেছে		উত্তর 3 হলে 508 নং প্রশ্নে যান

নম্বর	সূচক	প্রশ্ন	উত্তর	কোড	নির্দেশনা
			99 = অন্যান্য (নির্দিষ্ট করুন)______		
507	AWM_507	আপনার মাসিকের শেষ তারিখ কবে? (LMP)	|____|____|/|____|____|/20|____|____| দিন মাস বছর		পরবর্তী পরিচ্ছেদে যান
508	AWM_508	কত বছর বয়সে আপনার মাসিক বন্ধ হয়েছে?	|____|____| বছর		

পরিচ্ছেদ 6: স্বাস্থ্য সেবা

নম্বর	সূচক	প্রশ্ন	উত্তর	কোড	নির্দেশনা
601	AWH_601	গত 24 মাসের মধ্যে আপনি জীবিত বা মৃত কোন সন্তান জন্ম দিয়েছিলেন কি?	1 = হ্যাঁ 2 = না		উত্তর না হলে পরিচ্ছেদ 7 এ যান
602	AWH_602	আপনি কত তারিখে জীবিত বা মৃত কোন সন্তান জন্ম দিয়েছিলেন?	|___|___|/|___|___|/20|___|___| দিন / মাস / বছর		
603	AWH_603	আপনার সন্তান কি এখন জীবিত আছে?	1 = হ্যাঁ 2 = না<br>88 = প্রযোজ্য নয়		
604	AWH_604	আপনার সন্তান যখন আপনার গর্ভে ছিল তখন কি গর্ভবতী হিসেবে আপনাকে নিবন্ধন করা হয়েছিল?	1 = হ্যাঁ 2 = না		
605	AWH_605	আপনার সন্তান যখন আপনার গর্ভে ছিল তখন আপনি কতবার গর্ভকালীন সেবা নিয়েছিলেন?	|____|____| বার		
606	AWH_606	আপনার সন্তান যখন আপনার গর্ভে ছিল তখন আপনি প্রধানত: কার কাছ থেকে গর্ভকালীন সেবা নিয়েছিলেন?<br><br>(একাধিক উত্তর হতে পারে)	1 = পাশ করা ডাক্তার<br>2 = নার্স/মিডওয়াইফ/ প্যারামেডিক<br>3 = এফ ডব্লিউ ভি (FWV)<br>4 = মেডিক্যাল এ্যাসিস্টেন্ট/ সাব এ্যাসিস্টেন্ট<br>5 = এনজিও স্বাস্থ্যকর্মী<br>6 = দক্ষ দাই<br>7 = প্রশিক্ষণপ্রাপ্ত সনাতন দাই<br>8 = সনাতন দাই<br>77 = জানিনা<br>99 = অন্যান্য (নির্দ্দিষ্ট করুন)______		
607	AWH_607	আপনার সন্তান যখন আপনার গর্ভে ছিল তখন কি আপনার ওজন মাপা হয়েছিল?	1 = হ্যাঁ 2 = না		
608	AWH_608	আপনার সন্তান যখন আপনার গর্ভে ছিল তখন কি আপনি আয়রন ও ফলিক এসিড ট্যাবলেট খেয়েছিলেন?	1 = হ্যাঁ 2 = না		উত্তর না হলে 612 এ যান
609	AWH_609	আপনার সন্তান যখন আপনার গর্ভে ছিল তখন কি আপনি কোথা থেকে আয়রন ও ফলিক এসিড ট্যাবলেট পেয়েছিলেন?<br><br>(একাধিক উত্তর হতে পারে)	*সরকারী স্বাস্থ্যসেবা*<br>1 = সরকারী মাঠকর্মী<br>2 = সরকারী স্যাটেলাইট ক্লিনিক/অস্থায়ী টিকাদান (ইপিআই)কেন্দ্র<br>3 = সরকারী কমিউনিটি ক্লিনিক<br>4 = সরকারী স্বাস্থ্য ও পরিবার কল্যাণ কেন্দ্র (FWC)<br>5 = সরকারী উপজেলা স্বাস্থ্য কমপ্লেক্স<br>6 = শিশু ও মাতৃমঙ্গল কেন্দ্র<br>7 = সরকারী হাসপাতাল<br>*এনজিও স্বাস্থ্যসেবা*<br>8 = এনজিও স্বাস্থ্য ক্লিনিক ও হাসপাতাল<br>9 = এনজিও মাঠকর্মী<br>10 = কমিউনিটি নিউট্রিশন প্রোমোটার (সিএনপি)<br>*ব্যক্তিমালিকানাধীন স্বাস্থ্যসেবা*<br>11 = প্রাইভেট ডাক্তার/ক্লিনিক/ হাসপাতাল<br>12 = ফার্মেসী<br>13 = হাতুড়ে ডাক্তার/সনাতন চিকিৎসক<br>14 = হোমিওপ্যাথিক<br>77 = জানিনা<br>99 = অন্যান্য (নির্দ্দিষ্ট করুন)______		
610	AWH_610	আপনার সন্তান যখন আপনার গর্ভে ছিল তখন আপনি কতমাস আয়রন ও ফলিক এসিড ট্যাবলেট খেয়েছিলেন?	|_____|_____| মাস		
611	AWH_611	আপনার সন্তান যখন আপনার গর্ভে ছিল তখন আপনি সপ্তাহে কতটি আয়রন ও ফলিক এসিড ট্যাবলেট খেয়েছিলেন?	|____|____| টি		

অংশগ্রহণকারীর আইডি:|___|___|___|___|___|___|___|___|___|___|___|___|___|___|___|___|

নম্বর	সূচক	প্রশ্ন	উত্তর	কোড	নির্দেশনা
612	AWH_612	আপনার সন্তান যখন আপনার গর্ভে ছিল তখন কি আপনি ক্যালসিয়াম ট্যাবলেট খেয়েছিলেন?	1 = হ্যাঁ 2 = না		উত্তর না হলে 618 এ যান
613	AWH_613	আপনার সন্তান যখন আপনার গর্ভে ছিল তখন আপনি কোথা থেকে ক্যালসিয়াম ট্যাবলেট পেয়েছিলেন?<br><br>(একাধিক উত্তর হতে পারে)	***সরকারী স্বাস্থ্যসেবা***<br>1 = সরকারী মাঠকর্মী<br>2 = সরকারী স্যাটেলাইট ক্লিনিক/অস্থায়ী টিকাদান (ইপিআই)কেন্দ্র<br>3 = সরকারী কমিউনিটি ক্লিনিক<br>4 = সরকারী স্বাস্থ্য ও পরিবার কল্যাণ কেন্দ্র (FWC)<br>5 = সরকারী উপজেলা স্বাস্থ্য কমপ্লেক্স<br>6 = শিশু ও মাতৃমঙ্গল কেন্দ্র<br>7 = সরকারী হাসপাতাল<br>***এনজিও স্বাস্থ্যসেবা***<br>8 = এনজিও স্বাস্থ্য ক্লিনিক ও হাসপাতাল<br>9 = এনজিও মাঠকর্মী<br>10 = কমিউনিটি নিউট্রিশন প্রোমোটার (সিএনপি)<br>***ব্যক্তিমালিকানাধীন স্বাস্থ্যসেবা***<br>11 = প্রাইভেট ডাক্তার/ক্লিনিক/ হাসপাতাল<br>12 = ফার্মেসী<br>13 = হাতুড়ে ডাক্তার/সনাতন চিকিৎসক<br>14 = হোমিওপ্যাথিক<br>77 = জানিনা<br>99 = অন্যান্য (নির্দিষ্ট করুন)_____		
614	AWH_614	আপনার সন্তান যখন আপনার গর্ভে ছিল তখন আপনি কতমাস ক্যালসিয়াম ট্যাবলেট খেয়েছিলেন?	|____|____| মাস		
615	AWH_615	আপনার সন্তান যখন আপনার গর্ভে ছিল তখন আপনি সপ্তাহে কতটি ক্যালসিয়াম ট্যাবলেট খেয়েছিলেন?	|____|____| টি		
616	AWH_616	আপনার সন্তান যখন আপনার গর্ভে ছিল তখন কি ডাক্তার বা অন্য কোন স্বাস্থসেবাপ্রদানকারী আপনাকে কি পুষ্টি বিষয়ক কোন পরামর্শ দিয়েছিলেন?	1 = হ্যাঁ 2 = না		উত্তর না হলে 618 এ যান
617	AWH_617	কি কি বিষয়ের উপর পরামর্শ দিয়েছিলেন?	1 = পর্যাপ্ত খাবার গ্রহণ<br>2 = পর্যাপ্ত বিশ্রাম<br>3 = পুষ্টিকর খাবারের ধরণ<br>4 = আয়রন ও ফলিক এসিড ট্যাবলেট খাওয়ার নিয়ম<br>5 = ক্যালসিয়াম ট্যাবলেট খাওয়ার নিয়ম		
**মৃত সন্তানের জন্ম হলে নিচের প্রশ্নগুলো করার প্রয়োজন নেই।**					
618	AWH_618	জন্মের 3 দিনের মধ্যে আপনার সন্তানের জন্ম ওজন নেয়া হয়েছিল কি?	1 = হ্যাঁ 2 = না<br>88 = প্রযোজ্য নয়		উত্তর না হলে 620 এ যান
619	AWH_619	আপনার সন্তানের জন্ম ওজন কত ছিল?	|____|.|____|____|____| কেজি<br>7.777 = জানিনা		
620	AWH_620	পরবর্তীতে কোন ডাক্তার বা অন্য কোন স্বাস্থসেবাপ্রদানকারী আপনার সন্তানের ওজন নিয়েছে কি?	1 = হ্যাঁ 2 = না<br>88 = প্রযোজ্য নয়		
621	AWH_621	আপনার সন্তানের বয়স অনুযায়ী ওজনের কোন চার্ট আপনার কাছে আছে কি?	1 = হ্যাঁ 2 = না		
622	AWH_622	আপনার সন্তান জন্মের পরে ডাক্তার বা অন্য কোন স্বাস্থসেবাপ্রদানকারী আপনাকে কি পুষ্টি বিষয়ক কোন পরামর্শ দিয়েছিলেন?	1 = হ্যাঁ 2 = না		উত্তর না হলে পরবর্তী পরিচ্ছেদ এ যান
623	AWH_623	কি কি বিষয়ের উপর পরামর্শ দিয়েছিলেন?	1 = পর্যাপ্ত খাবার গ্রহণ<br>2 = পর্যাপ্ত বিশ্রাম<br>3 = পুষ্টিকর খাবারের ধরণ<br>6 = প্রথম 6 মাস শুধুমাত্র বুকের দুধ খাওয়ানো<br>7 = 2 বছর বয়স পর্যন্ত বুকের দুধ খাওয়া চালিয়ে যাওয়া		

নম্বর	সূচক	প্রশ্ন	উত্তর	কোড	নির্দেশনা
			4 = আয়রন ও ফলিক এসিড ট্যাবলেট খাওয়ার নিয়ম<br>5 = ক্যালসিয়াম ট্যাবলেট খাওয়ার নিয়ম<br>8 = সম্পূরক খাবার খাওয়ানোর নিয়ম<br>9 = অসুস্থ অবস্থায় শিশুকে খাওয়ানোর নিয়ম<br>10 = দোকানের খাবার না খাওয়ানো		

অংশগ্রহণকারীর আইডি:|___|___|___|___|___|___|___|___|___|___|___|___|___|___|___|___|

পরিচ্ছেদ 7: রক্তচাপ পরিমাপ

নম্বর	সূচক	প্রশ্ন	উত্তর			কোড	নির্দেশনা
701	WBP_701	রক্তচাপ পরিমাপ নেয়া শুরুর সময়	|____|____| : |____|____| ঘন্টা : মিনিট			24 ঘণ্টার ফরম্যাট ব্যবহার করুন	
**পরিমাপ নং-1** (পরিমাপ নেয়ার পূর্বে নিশ্চিত হোন যেন উত্তরদাতা কমপক্ষে 15 মিনিট বিশ্রামে থাকেন)							
**নম্বর**	**সূচক**	**পরিমাপের নাম**	**Systolic**	**Diastolic**	**পরিমাপের সময়**		
702	WBP_702 A/B/C	রক্তচাপ (mm of Hg)	A.|___|___|___|	B.|___|___|___|	C.|____|____|:|____|____| ঘন্টা : মিনিট		
**পরিমাপ নং-2** (দুইটি পরিমাপের মধ্যে 3 মিনিট বিরতি দিন)							
703	WBP_703 A/B/C	রক্তচাপ (mm of Hg)	**Systolic**<br>A.|___|___|___|	**Diastolic**<br>B.|___|___|___|	**পরিমাপের সময়**<br>C.|____|____|:|____|____| ঘন্টা : মিনিট		
**পরিমাপ-3** (যদি যে কোনটির ক্ষেত্রে 1ম ও 2য় পরিমাপের মধ্যে পার্থক্য 10 এর বেশি হয়, তাহলে ৩য় বার রক্তচাপ নিন)							
704	WBP_704 A/B/C	রক্তচাপ (mm of Hg)	**Systolic**<br>A.|___|___|___|	**Diastolic**<br>B.|___|___|___|	**পরিমাপের সময়**<br>C.|____|____|:|____|____| ঘন্টা : মিনিট		
705	WBP_705	রক্তচাপ পরিমাপ যন্ত্রের আইডি	|____|____|				
706	WBP_706	রক্তচাপ পরিমাপ গ্রহণকারীর নাম ও কোড	|____|____|				আপনার পুরা নাম এবং কোড লিখুন
707	WBP_707	রক্তচাপ পরিমাপ গ্রহণের শেষের সময়	|____|____| : |____|____| ঘন্টা : মিনিট			24 ঘণ্টার ফরম্যাট ব্যবহার করুন	
**[যে কোন পরিমাপের সময়, Systolic BP ≥140 mm of Hg, Diastolic BP ≥90 mm of Hg-এর যে কোন একটি হলে অংশগ্রহণকারীকে নির্দিষ্ট স্বাস্থ্য কেন্দ্রে রেফার করুন ।]**							

রেফারেল (Referral)

নম্বর	সূচক	প্রশ্ন	উত্তর	কোড	নির্দেশনা
708	WBP_708	রেফার করা হয়েছে কি?	1 = হ্যাঁ 2 = না		উত্তর না হলে, পরিচ্ছেদ 8 এ যান
709	WBP_709	কোথায় রেফার করা হয়েছে	1 = উপজেলা স্বাস্থ্য কমপ্লেক্স<br>2 = জেলা হাসপাতাল<br>3 = মেডিকেল কলেজ হাসপাতাল<br>4 = ডাক্তারের চেম্বার<br>99 = অন্যান্য (নির্দিষ্ট করুন)_____		

পরিচ্ছেদ 8: পরিমাপ

<table>
<tr><th>নম্বর</th><th>সূচক</th><th colspan="2">প্রশ্ন</th><th>উত্তর</th><th>কোড</th><th>নির্দেশনা</th></tr>
<tr><td>801</td><td>AWA_801</td><td colspan="2">পরিমাপ নেয়া শুরুর সময়</td><td>|____|____| : |____|____|
ঘন্টা : মিনিট</td><td></td><td>24 ঘণ্টার ফরম্যাট ব্যবহার করুন</td></tr>
<tr><td>802</td><td>AWA_802</td><td colspan="2">ওজন পরিমাপক যন্ত্রের আইডি</td><td>|____|____|</td><td></td><td></td></tr>
<tr><td>803</td><td>AWA_803</td><td colspan="2">উচ্চতা পরিমাপক যন্ত্রের আইডি</td><td>|____|____|</td><td></td><td></td></tr>
<tr><td>804</td><td>AWA_804</td><td colspan="2">ওজন নেয়ার সময় গায়ের কাপড়ের ধরন</td><td>1 = হালকা কাপড়
2 = একটু ভারী কাপড়
3 = ভারী কাপড়</td><td></td><td></td></tr>
<tr><td>805</td><td>AWA_805</td><td colspan="2">পরিমাপ গ্রহণকারীর নাম</td><td></td><td></td><td></td></tr>
<tr><td>806</td><td>AWA_806</td><td colspan="2">পরিমাপ গ্রহণকারীর কোড</td><td>|____|____|</td><td></td><td></td></tr>
<tr><th>নম্বর</th><th>সূচক</th><th>পরিমাপের নাম</th><th>পরিমাপ 1</th><th>পরিমাপ 2</th><th colspan="2">পরিমাপ 3</th></tr>
<tr><td>807</td><td>AWA_807 A/B/C</td><td>উচ্চতা (Cm)</td><td>A.|____|____|____|.|____|</td><td>B.|____|____|____|.|____|</td><td colspan="2">C.|____|____|____|.|____|
যদি A ও B এর মধ্যে পার্থক্য 0.5cm এর বেশি হয়, তাহলে ৩য় বার মাপুন</td></tr>
<tr><td>808</td><td>AWA_808 A/B/C</td><td>ওজন (Kg)</td><td>A.|___|____|____|.|____|</td><td>B.|___|___|___|.|___|</td><td colspan="2">C.|___|___|___|. |___|
যদি A ও B এর মধ্যে পার্থক্য 0.1Kg এর বেশি হয়, তাহলে ৩য় ওজন নিন</td></tr>
<tr><td>809</td><td>AWA_809 A/B/C</td><td>চর্বি (%)</td><td>A.|____|____|____|.|____|</td><td>B.|____|____|____|.|____|</td><td colspan="2">C.|____|____|____|.|____|
যদি A ও B এর মধ্যে পার্থক্য 0.5cm এর বেশি হয়, তাহলে ৩য় বার মাপুন</td></tr>
<tr><td>810</td><td>AWA_810 A/B/C</td><td>পানি (%)</td><td>A.|___|____|____|.|____|</td><td>B.|__ _|___|___|.|____|</td><td colspan="2">C.|____|____|____|. |____|
যদি A ও B এর মধ্যে পার্থক্য 0.10Kg এর বেশি হয়, তাহলে ৩য় ওজন নিন</td></tr>
<tr><td>811</td><td>AWA_811 A/B/C</td><td>কোমরের পরিধি (Cm)</td><td>A.|____|____|.|____|</td><td>B.|____|____|.|____|</td><td colspan="2">C.|____|____|.|____|
যদি A ও B এর মধ্যে পার্থক্য 0.5cm এর বেশি হয়, তাহলে ৩য় বার মাপুন</td></tr>
<tr><td>812</td><td>AWA_812</td><td colspan="2">পরিমাপ সম্পর্কে তথ্য সংগ্রহকারীর মন্তব্য</td><td colspan="3"></td></tr>
<tr><td>813</td><td>AWA_813</td><td colspan="2">পরিমাপ গ্রহণের শেষের সময়</td><td colspan="2">|____|____| : |____|____|
ঘন্টা : মিনিট</td><td>24 ঘণ্টার ফরম্যাট ব্যবহার করুন</td></tr>
</table>

অংশগ্রহণকারীর আইডি:|___|___|___|___|___|___|___|___|___|___|___|___|___|___|___|___|

খাদ্য নিরাপত্তা ও পুষ্টি বিষয়ক সার্ভিলেন্স

মডিউল 8: বৃদ্ধ বা বৃদ্ধার তথ্যাবলী

পরিচ্ছেদ 1: সাধারণ তথ্যাবলী

নম্বর	সূচক	প্রশ্ন	উত্তর	কোড	নির্দেশনা
101	EM_101	আপনার নাম (ডাক নামসহ)	______________________________		
102	EM_102	আপনার জন্ম তারিখ	|____|____|/|____|____|/19|____|____| দিন মাস বছর		
103	EM_103	আপনার বয়স (বয়স পূর্ন বছরে লিখুন)	|____|____| বছর		
104	EM_104	লিঙ্গ	1 = পুরুষ 2 = মহিলা		
105	EM_105	আপনার বৈবাহিক অবস্থা?	1 = কখনই বিবাহ করিনি<br>2 = বর্তমানে বিবাহিত<br>3 = পৃথক<br>4 = তালাকপ্রাপ্ত<br>5 = বিপত্নিক/বিধবা<br>88 = জানাতে অসম্মতি		
106	EM_106	আপনি সর্বমোট কত বছর প্রাতিষ্ঠানিক শিক্ষা গ্রহণ করেছেন?<br><br>(প্রথম শ্রেণীর নিচে এবং উপানুষ্ঠিক শিক্ষা অন্তর্ভুক্ত হবে না)<br>***নির্দেশনাঃ***<br>*তথ্য প্রদানকারীর সর্বমোট কত বছর প্রাতিষ্ঠানিক শিক্ষা সম্পন্ন করেছেন তা লিপিবদ্ধ করুন। প্রাতিষ্ঠানিক শিক্ষা না থাকলে 00 লিখুন।*	|____|____| বছর<br>প্রাথমিক শিক্ষা/এবতেদায়ী = 5<br>মাধ্যমিক/দাখিল =10<br>উচ্চ মাধ্যমিক/ ডিপ্লোমা/আলিম= 12<br>স্নাতক/ফাজিল =16<br>স্নাতাকোত্তর/কামিল/দাওরা =18		
107	EM_107	গত 12 মাসে আপনার প্রধান পেশা কি ছিল?<br><br>যদি তথ্য প্রদানকারী গত 12 মাসে একাধিক পেশায় নিযুক্ত থাকেন তাহলে তিনি যে পেশাটিতে বেশী সময় ব্যয় করেছেন সেটিকে প্রধান পেশা হিসাবে বিবেচনা করুন এবং তা লিপিবদ্ধ করুন।	1 = কৃষিকাজ (ধান)<br>2 = কৃষিকাজ (ধান ছাড়া অন্যান্য)<br>3 = কৃষি দিনমজুর<br>4 = অদক্ষ দিনমজুর<br>5 = দক্ষ দিনমজুর<br>6 = রিক্সা/ ভ্যান/ ঠেলাগাড়ী/ বেবীট্যাক্সি ড্রাইভার/ নৌকার মাঝি<br>7 = জেলে<br>8 = চাকুরীজীবি<br>9 = পেশাজীবি<br>10 = ব্যবসায়ী<br>11 = ক্ষুদে ব্যবসায়ী<br>12 = গৃহপরিচারিকা<br>13 = জুমচাষী<br>14 = উপার্জন করে না<br>15 = হাঁস/ মুরগী পালন/ পশু পালন<br>16 = হস্তশিল্প<br>17 = শাক-সব্জি চাষ<br>18 = মৎস চাষ<br>19 = গৃহিনী<br>20 = ছাত্র/ছাত্রী<br>77 = জানিনা<br>66 = বয়স 6 বছরের কম<br>99 = অন্যান্য (নির্দিষ্ট করুন) ___		
108	EM_108	আপনি কি সরকারী বা বেসরকারী প্রতিষ্ঠান হতে কান ধরণের পেনশন পান?	1 = হ্যাঁ 2 = না		
109	EM_109	আপনি কি কোন ধরণের বয়স্ক ভাতা পান?	1 = হ্যাঁ 2 = না		
110	EM_110	সব ধরনের উৎস হতে আপনার গড় মাসিক আয় কত?	টাকা:----------		কোন আয় না থাকলে 00 লিখুন

পরিচ্ছেদ 2ঃ খাদ্য গ্রহণের বৈচিত্র্য

নম্বর	সূচক	প্রশ্ন		গতকাল দিনে বা রাতে আপনি নিম্নবর্ণিত খাবারগুলো খেয়েছেন কি? (A)	গত 7 দিনে আপনি নিম্নবর্ণিত খাবারগুলো কতদিন খেয়েছেন? (B)	গত 7 দিনে আপনি নিম্নবর্ণিত খাবারগুলো কতবার খেয়েছেন? (C)
এখন আমি আপনাকে গতকাল দিনে বা রাতে (গতকাল সকাল 6:00 টা থেকে আজ সকাল 6:00 টা পর্যন্ত) আপনি বাড়ীতে ও বাড়ীর বাইরে যা যা খেয়েছেন তা বর্ণণা করতে অনুরোধ করছি। দয়া করে সব ধরনের খাবার, পানীয় যা আপনি সকাল, দুপুর ও রাতের খাবারের সাথে খেয়েছেন বা নাস্তা/হালকা নাস্তা করেছেন সে সম্পর্কে বলুন। আপনি খাবার তৈরীর সময় কোন খাবার খেয়ে থাকলে তাও মনে করে আমাদেরকে বলুন। আপনি গতকাল সকালে যা খেয়েছেন তা দিয়েই শুরু করুন।<br>- আপনি সকালে ঘুম থেকে উঠে কী কী খেয়েছেন? আরো কিছু খেয়েছেন কি?<br>- সকালে আরো কী কী খেয়েছেন? আরো কিছু খেয়েছেন কি?<br>- দুপুরে কী কী খেয়েছেন? আরো কিছু খেয়েছেন কি?<br>- বিকালে কী কী খেয়েছেন? আরো কিছু খেয়েছেন কি?<br>- সন্ধ্যায় কী কী খেয়েছেন? আরো কিছু খেয়েছেন কি?<br>- রাতে কী কী খেয়েছেন? আরো কিছু খেয়েছেন কি?<br>A কলামের প্রশ্নগুলোর উত্তর প্রথমে রেকর্ড করুন। A কলামের প্রশ্নগুলোর উত্তর রেকর্ড করা শেষ হলে B/ C কলামের উত্তর নিন।						
		খাবার ধরণ	খাবার নমুনা			
201	EDD_201 A/B/C	শ্বেতসার জাতীয় (শস্য জাতীয় খাবার)	ভাত, আটা রুটি, গম, মুড়ি, ভুট্টা, খিচুড়ি, বার্লি, ওট, কিনোয়া, নুডলস, পাস্তা	1 = হ্যাঁ<br>2 = না	|____|____| দিন	|____|____| বার
202	EDD_202 A/B/C	শ্বেতসার জাতীয় (মূল, কন্দ, কলা)	গোলআলু, মিষ্টিআলু, সাগু, এরারুট, কাঁচকলা, শালগম, কাসাভা, কচু, কচুমুখী, পাকাকলা, শালুক	1 = হ্যাঁ<br>2 = না	|____|____| দিন	|____|____| বার
203	EDD_203 A/B/C	ডাল ও ডাল জাতীয় খাবার	ডাল, শিমের বীচি, মটর, সয়বীন, টফু, হুমাস	1 = হ্যাঁ<br>2 = না	|____|____| দিন	|____|____| বার
204	EDD_204 A/B/C	বাদাম ও তৈলবীজ	চীনা বাদাম, পেস্তা, কাজু, অথবা যেকোন বাদাম, চিয়া সীড, তিল, তিসি, সূর্যমুখী বীজ, মিষ্টি কুমড়া বীজ	1 = হ্যাঁ<br>2 = না	|____|____| দিন	|____|____| বার
205	EDD_205 A/B/C	গাঢ় সবুজ পাতা জাতীয় শাক	সকল ধরনের পাতা জাতীয় শাক (পুঁই, কচু, কলমি), ব্রকলি	1 = হ্যাঁ<br>2 = না	|____|____| দিন	|____|____| বার
206	EDD_206 A/B/C	লাল/কমলা/ হলুদ সব্জি	মিষ্টিকুমড়া, গাজর, গাঢ় হলুদ বা কমলা মিষ্টি আলু, ও অন্যান্য লাল/ কমলা/হলুদ রঙের সব্জি	1 = হ্যাঁ<br>2 = না	|____|____| দিন	|____|____| বার
207	EDD_207 A/B/C	লাল/কমলা/ হলুদ ফলমূল	পাকা আম, পাকা পেঁপে ও অন্যান্য লাল/কমলা/ হলুদ ফলমূল	1 = হ্যাঁ<br>2 = না	|____|____| দিন	|____|____| বার
208	EDD_208 A/B/C	ভিটামিন সি- সমৃদ্ধ ফল	পেয়ারা, স্ট্রবেরী, লেবু, কমলালেবু, আঙ্গুর, আনারস, কাঁচা আম, আমলকি, কিউই, টমেটো	1 = হ্যাঁ<br>2 = না	|____|____| দিন	|____|____| বার
209	EDD_209 A/B/C	ভিটামিন সি- সমৃদ্ধ সব্জি	কাঁচা টমেটো, কাঁচা মরিচ, ব্রাসেলস স্প্রাউট, ফুলকপি, বাঁধাকপি	1 = হ্যাঁ<br>2 = না	|____|____| দিন	|____|____| বার
210	EDD_210 A/B/C	অন্যান্য শাক সব্জি	শিম, অ্যাসপারাগাস, বিট, কচি বাঁশ, ফুলকপি, সেলারি, শসা,	1 = হ্যাঁ<br>2 = না	|____|____| দিন	|____|____| বার

নম্বর	সূচক	প্রশ্ন		গতকাল দিনে বা রাতে আপনি নিম্নবর্ণিত খাবারগুলো খেয়েছেন কি? (A)	গত 7 দিনে আপনি নিম্নবর্ণিত খাবারগুলো কতদিন খেয়েছেন? (B)	গত 7 দিনে আপনি নিম্নবর্ণিত খাবারগুলো কতবার খেয়েছেন? (C)
			বেগুন, লেটুস, মাশরুম, মূলা, জুকিনি			
211	EDD_211 A/B/C	অন্যান্য ফলমূল	আপেল, আভাকাডো, জাম, চেরী, পাকা কাঁঠাল	1 = হ্যাঁ<br>2 = না	|___|___| দিন	|___|___| বার
212	EDD_212 A/B/C	ডিম	হাঁস-মুরগী, অন্যান্য পাখী	1 = হ্যাঁ<br>2 = না	|___|___| দিন	|___|___| বার
213	EDD_213 A/B/C	অঙ্গ জাতীয় মাংস	গিলা, কলিজা, পাকস্থলী, হৃদপিণ্ড, কিডনি	1 = হ্যাঁ<br>2 = না	|___|___| দিন	|___|___| বার
214	EDD_214 A/B/C	মাংস	গরু, শুকর, বাছুর, মেষশাবক, ছাগল, মুরগী, হাঁস বা যেকোন প্রাণীর মাংস	1 = হ্যাঁ<br>2 = না	|___|___| দিন	|___|___| বার
215	EDD_215 A/B/C	ছোট মাছ	ছোট মাছের কাঁটা/হাড়সহ (কাঁচকি, মলা, ঢেলা, চাপিলা, বাতাসি, ছোট চিংড়ি, ছোট মাছের শুঁটকি)	1 = হ্যাঁ<br>2 = না	|___|___| দিন	|___|___| বার
216	EDD_216 A/B/C	বড় মাছ/সামুদ্রিক মাছ/ সামুদ্রিক খাবার	বড় মাছ, ঝিনুক, কাঁকড়া, অক্টোপাস, স্কুইড, হাঙর, বড় চিংড়ি, বড় মাছের শুঁটকি	1 = হ্যাঁ<br>2 = না	|___|___| দিন	|___|___| বার
217	EDD_217 A/B/C	দুধ ও দুগ্ধ জাতীয় খাবার	দুধ, পনির, দই এবং অন্যান্য দুগ্ধজাতীয় খাদ্য	1 = হ্যাঁ<br>2 = না	|___|___| দিন	|___|___| বার
218	EDD_218 A/B/C	পোকা ও অন্যান্য আমিষ জাতীয় খাবার	মাছের ডিম, পোকা, শামুক	1 = হ্যাঁ<br>2 = না	|___|___| দিন	|___|___| বার
219	EDD_219 A/B/C	তৈল জাতীয় খাবার	ঘি, মাখন, ক্রিম, সর, চর্বি, মার্জারিন, ম্যায়োনেজ, পাম অয়েল, উদ্ভিজ্জ তেল	1 = হ্যাঁ<br>2 = না	|___|___| দিন	|___|___| বার
220	EDD_220 A/B/C	চিনিযুক্ত এবং ভাজা খাবার	খাস্তা, চিপস ও অন্যান্য ভাজা খাবার, সিংগারা, সমোচা	1 = হ্যাঁ<br>2 = না	|___|___| দিন	|___|___| বার
221	EDD_221 A/B/C	মিষ্টি জাতীয় খাবার	চিনিযুক্ত খাবার, যেমন চকলেটস, ক্যান্ডিস, কুকিস/মিষ্টি বিস্কুট এবং কেকস, মিষ্টি পেষ্ট্রি বা আইসক্রিম, যে কোন মিষ্টি, মধু, হালুয়া, কনডেন্সড দুধ, তিলের খাজা	1 = হ্যাঁ<br>2 = না	|___|___| দিন	|___|___| বার
222	EDD_222 A/B/C	মিষ্টি পানীয়	চিনি যুক্ত চা, কোমল পানীয়, জুস, এনার্জি ড্রিংক, ইয়োগার্ট ড্রিংক, চকলেট ড্রিংক, হরলিকস, মলটোভা	1 = হ্যাঁ<br>2 = না	|___|___| দিন	|___|___| বার
223	EDD_223 A/B/C	অন্যান্য পানীয় এবং খাবার	চিনি ছাড়া চা, মদ, বিয়ার, আচার, সুপ, উপরের তালিকাভুক্ত নয় এমন যেকোন খাবার	1 = হ্যাঁ<br>2 = না	|___|___| দিন	|___|___| বার

নম্বর	সূচক	প্রশ্ন		গতকাল দিনে বা রাতে আপনি নিম্নবর্ণিত খাবারগুলো খেয়েছেন কি? (A)	গত 7 দিনে আপনি নিম্নবর্ণিত খাবারগুলো কতদিন খেয়েছেন? (B)	গত 7 দিনে আপনি নিম্নবর্ণিত খাবারগুলো কতবার খেয়েছেন? (C)
224	EDD_224 A/B/C	অন্যান্য খাবার সুস্বাদু করার দ্রব্যাদি	মসলা, ধনে পাতা, সস, রসুন, কেচাপ, লেবুর রস, পুদিনা পাতা, পান, সুপারি, তামাক পাতা, জর্দা	1 = হ্যাঁ<br>2 = না	|____|____| দিন	|____|____| বার

নম্বর	সূচক	প্রশ্ন	উত্তর	কোড	নির্দেশনা
আমি আপনাকে পরবর্তীতে যে প্রশ্নগুলো জিজ্ঞাসা করতে যাচ্ছি তা হল সচরাচর আপনি যে সকল ফলমূল ও শাক-শব্জি খেয়ে থাকেন সে বিষয়ে। আমার কাছে ফল ও শাক-শব্জির কিছু ছবি আছে। প্রতিটি ছবি এক একটি প্রমান মাপের সমান। উত্তর দেওয়ার সময় সাধারন 1টি সপ্তাহের কথা চিন্তা করুন। (মাসে 1-2 বার হলে উত্তর 00 হবে)					
225	EDD_225	সচরাচর সপ্তাহের কত দিন আপনি ফল খান ?<br>(নমুনা কার্ড দেখান)<br>*নির্দেশনাঃ*<br>*তথ্য প্রদানকারীকে নমুনা কার্ডে প্রদর্শিত ফলগুলো দেখিয়ে চিন্তা করতে বলুন। এখানে প্যাকেটজাত ফলের জুস গ্রহণযোগ্য নয় তবে বাসায় ব্লেন্ড করা ফলের জুস গ্রহণযোগ্য। সপ্তাহ বলতে ধর্মীয় বা অন্য কোন বিশেষ উপলক্ষ্য ব্যতীত একটি স্বাভাবিক সপ্তাহ বুঝায়। মাসে 1-2 বার হলে 00 লিখুন।*	দিনের সংখ্যা<br>|___|___| 77 = জানা নাই<br>(যদি 00 দিন হয়, তাহলে EDD_227-তে যান)		
226	EDD_226	সেই দিন গুলির একদিনে কতটুকু ফল খেয়েছেন? (নমুনা কার্ড ও বাটি দেখান)<br>*নির্দেশনাঃ*<br>*তথ্য প্রদানকারীকে যে কোন একদিনের কথা স্মরণ করতে বলুন এবং বাটি দেখিয়ে পরিমাপ করতে বলুন।*	সারভিং সংখ্যা<br>|___|___|.|___| 77.7 = জানা নাই		
227	EDD_227	সচরাচর সপ্তাহের কত দিন আপনি শাক-সব্জি খান?<br>(নমুনা কার্ড দেখান)<br>*নির্দেশনাঃ*<br>*তথ্য প্রদানকারীকে নমুনা কার্ডে প্রদর্শিত শাক-সব্জিগুলো দেখিয়ে চিন্তা করতে বলুন। এখানে আলু শাক-সব্জি হিসাবে গণ্য হবে না। কাঁচা ও রান্না করা শাক-সব্জির প্রমান পরিমাপ আলাদা করে দেখান। মাছের সাথে রান্না করা সব্জির ক্ষেত্রে শুধু সব্জির পরিমান করতে হবে। সপ্তাহ বলতে ধর্মীয় বা অন্য কোন বিশেষ উপলক্ষ্য ব্যতীত একটি স্বাভাবিক সপ্তাহ বুঝায়। মাসে 1-2 বার হলে '00' হবে।*	দিনের সংখ্যা<br>|___|___| 77 = জানা নাই (যদি '00' দিন হয়, তাহলে EDD_229-তে যান)		
228	EDD_228	সই দিন গুলির একদিনে কতটুকু পরিমান শাক-সব্জি খেয়েছেন? (নমুনা কার্ড ও বাটি দেখান)<br>*নির্দেশনাঃ*<br>*তথ্য প্রদানকারীকে যে কোন একদিনের কথা স্মরণ করতে বলুন এবং বাটি দেখিয়ে পরিমাপ করতে বলুন।*	সারভিং সংখ্যা<br>|___|___|.|___| 77.7 = জানা নাই		
229	EDD_229	আপনি কি বর্তমানে কোন ধরণের ভিটামিন/খনিজ লবন আছে এমন কোন ট্যাবলেট, ক্যাপসুল, সিরাপ খান?	1 = হ্যাঁ<br>2 = না		যদি 'না' হয় তাহলে পরবর্তী পরিচ্ছেদে যান

অংশগ্রহণকারীর আইডি:|___|___|___|___|___|___|___|___|___|___|___|___|___|___|___|___|

খাদ্য নিরাপত্তা ও পুষ্টি বিষয়ক সার্ভিলেন্স

নম্বর	সূচক	প্রশ্ন	উত্তর	কোড	নির্দেশনা
230	EDD_230	ব্যবস্থাপত্র দেখে বা ঔষধ দেখে নাম লিখুন	A.____________________<br>B.____________________<br>C.____________________		

অংশগ্রহণকারীর আইডি:|___|___|___|___|___|___|___|___|___|___|___|___|___|___|___|___|

খাদ্য নিরাপত্তা ও পুষ্টি বিষয়ক সার্ভিলেন্স

পরিচ্ছেদ 3ঃ আচরণগত ঝুঁকির কারণ (ধুমপান/ধোঁয়াহীন তামাক এবং শারীরিক পরিশ্রম)

নম্বর	সূচক	প্রশ্ন	উত্তর	কোড	নির্দেশনা
**মূলঃ তামাকের ব্যবহার**					
এখন আমি আপনাকে তামাক ও তামাকজাত দ্রব্যের (যেমনঃ ধূমপান, ধোঁয়াবিহীন তামাকের) ব্যবহার সম্পর্কে কিছু প্রশ্ন জিজ্ঞাসা করব।					
301	ER_301	আপনি কি বর্তমানে কোন প্রকার ধূমপান করেন?<br>(যেমন: সিগারেট, বিড়ি, হুক্কা, চুরুট, সিগার)<br>[নমুনা কার্ড দেখান]<br>***নির্দেশনাঃ***<br>*উত্তরদাতাকে নমুনা কার্ড দেখিয়ে চিন্তা করতে বলুন যে বর্তমানে তিনি কোন দ্রব্যটি ধূমপান/ ব্যবহার করছেন।*	1 = হ্যাঁ<br>2 = না<br>(যদি না হয়, তাহলে ER_304 এ যান)		
302	ER_302	আপনি কি বর্তমানে প্রতিদিন ধূমপান করেন?<br>***নির্দেশনাঃ***<br>*এই প্রশ্নটি যারা বর্তমানে ধূমপান করেন তাদের জন্য প্রযোজ্য।*<br>***প্রতিদিন অর্থঃ***<br>*প্রায় একমাস বা তার বেশী সময় ধরে প্রতিদিন অন্তত একবার ধূমপান করা কে বুঝায়। যদি এমন হয় যে তথ্য প্রদানকারী 25 দিন হল ধূমপান শুরু করেছে এবং এখনো চলছে, সেক্ষেত্রে প্রতিদিন হিসাবে গণ্য হবে।*	1 = হ্যাঁ<br>2 = না		
303	ER_303	কত বছর বয়সে আপনি প্রথম ধূমপান শুরু করেন?	বয়স |___|___| 77 = জানা নাই		
304	ER_304	আপনি কি বর্তমানে কোন প্রকার ধোঁয়াবিহীন তামাক দ্রব্য ব্যবহার করেন?<br>(যেমনঃ পানের সাথে জর্দা, শুধু জর্দা, সুপারির সাথে জর্দা, পানের সাথে সাদাপাতা, তামাকযুক্ত পানমশলা, চিবিয়ে খাওয়া সাদাপাতা, খৈনি, নস্যি, গুল, ইত্যাদি)<br>[নমুনা কার্ড দেখান]<br>***নির্দেশনাঃ***<br>*তথ্য প্রদানকারীকে ধোঁয়বিহীন তামাক যেমনঃ জর্দা, গুল, সাদাপাতা, খৈনি, নস্যি দ্রব্যগুলো কি বর্তমানে ব্যবহার করেন কিনা তা চিন্তা করে উত্তর দিতে বলুন। এক্ষেত্রে, শুধু পান সুপারী ও চুন প্রযোজ্য হবে না। যদি তথ্য প্রদানকারী পানের সাথে জর্দা বা শুধু জর্দা, পানের সাথে সাদাপাতা বা শুধু সাদাপাতা, পানের সাথে তামাক যুক্ত পান মশলা বা শুধু তামাক যুক্ত পান মশলা খান তাহলে ধোঁয়াবিহীন তামাক সেবন হিসাবে গণ্য হবে।*	1 = হ্যাঁ<br>2 = না<br>(যদি না হয়, তাহলে ER_307 এ যান)		
305	ER_305	আপনি কি বর্তমানে প্রতিদিন এই ধোঁয়াবিহীন তামাক দ্রব্য ব্যবহার করেন?<br>**প্রতিদিন অর্থঃ** প্রায় একমাস বা তার বেশী সময় ধরে প্রতিদিন অন্তঃত একটি ধোঁয়াবিহীন তামাক পণ্য ব্যবহার করা কে বুঝায়। *যদি এমন হয় যে তথ্য প্রদানকারী 25 দিন হল ধূমপান শুরু করেছে এবং এখনো চলছে, সেক্ষেত্রে প্রতিদিন হিসাবে গণ্য হবে।*	1 = হ্যাঁ<br>2 = না		
306	ER_306	কত বছর বয়সে আপনি প্রথম ধোঁয়াবিহীন তামাক দ্রব্য গ্রহণ শুরু করেন?	বয়স |___|___| 77 = জানা নাই		
307	ER_307	আপনার বাবা-মা বা অভিভাবকরা কোনও ধরনের তামাক ব্যবহার করেন?	1 = কেউ না<br>2 = আমার বাবা বা পুরুষ অভিভাবক<br>3 = আমার মা বা মহিলা অভিভাবক<br>4 = উভয়<br>5 = আমি জানি না<br>99 = অন্যান্য (নির্দিষ্ট করুন) ______		

অংশগ্রহণকারীর আইডি:|___|___|___|___|___|___|___|___|___|___|___|___|___|___|___|___|

খাদ্য নিরাপত্তা ও পুষ্টি বিষয়ক সার্ভিলেন্স

নম্বর	সূচক	প্রশ্ন	উত্তর	কোড	নির্দেশনা
**মূলঃ শারীরিক পরিশ্রম সংক্রান্ত তথ্য**					
এর পর আমি আপনাকে সপ্তাহে আপনি বিভিন্ন ধরনের শারীরিক পরিশ্রমে যে সময় কাটান সে সম্পর্কিত কিছু প্রশ্ন করবো। আপনি নিজেকে শারীরিকভাবে সক্রিয় মনে না করলেও, অনুগ্রহ করে এই প্রশ্নগুলোর উত্তর দিন। প্রথমে আপনি কাজ করার জন্য যে সময় ব্যয় করেন সে সম্পর্কে ভাবুন। সেই কাজগুলোর কথা ভাবুন যে কাজগুলো আপনি টাকার বিনিময়ে বা বিনামূল্যে করে থাকনে। পড়াশুনা, প্রশিক্ষণ, গৃহস্থালীর কাজ, খাদ্যশস্যের চাষাবাদ, মাছ ধরা বা চাকুরী খোঁজা। এখানে অতিমাত্রার ভারী কাজ বলতে সেই কাজগুলোকে বুঝায় যে কাজগুলো করতে বেশী পরিমানে শারীরিক পরিশ্রমের প্রয়োজন হয় এবং কাজগুলো করার ফলে শ্বাস-প্রশ্বাস অথবা হৃদস্পন্দন অনেক বেড়ে যায় এবং মধ্যম মাত্রার কাজ বলতে সেই কাজগুলোকে বোঝায় যে কাজগুলো করতে মাঝারী পরিমানের শারীরিক পরিশ্রমের প্রয়োজন হয় এবং কাজগুলো করার ফলে শ্বাস-প্রশ্বাস অথবা হৃদস্পন্দন সামান্য বেড়ে যায়।<br><br>নির্দেশনাঃ উপরের ভূমিকাটি তথ্য প্রদানকারীকে পড়ে শুনান। এই অংশটি বাদ দেয়া যাবে না। তথ্য প্রদানকারীকে প্রথমে অবশ্যই তার দৈনন্দিন কাজগুলো সম্পর্কে চিন্তা করবে (পারিশ্রমিক ও পারিশ্রমিকবিহীন কাজ, গৃহস্থালীকাজ, খাদ্য উৎপাদন, খাওয়ার জন্য মাছ ধরা, কাজ খোঁজা, এক জায়গা থেকে অন্য জায়গায় যাওয়ার জন্য ব্যয়িত সময় এবং সবশেষে অবসর সময়ে ব্যয়িত সময়)					
**এখন আমি আপনার করা অতিমাত্রার ভারী কাজ সম্পর্কে জানতে চাইবো**					
দৈনন্দিন কাজের/ পেশাগত কাজের অংশ হিসেবে করা অতিমাত্রার ভারী কাজ					
308	ER_308	আপনাকে কি দৈনন্দিন কাজের/পেশাগত কাজের অংশ হিসেবে শ্বাস প্রশ্বাস ও হৃদস্পন্দন অনেক বেড়ে যায় এমন কোন অতিমাত্রার ভারী কাজ/ অতিমাত্রার খেলাধুলা, শরীরচর্চা অথবা বিনোদন মূলক কাজ একনাগাড়ে কমপক্ষে 10 মিনিট ধরে করতে হয়? [অতিমাত্রার ভারী কাজ যেমন ভারী জিনিস বহন করা বা তোলা, মাটি কাটা, নির্মাণ কাজ, ধান কাটা, জাল দিয়ে মাছ ধরা ইত্যাদি, অতিমাত্রার খেলাধুলা, শরীরচর্চা অথবা বিনোদন মূলক কাজ: দৌড়ানো, কাবাডি, ফুটবল খেলা, দাড়িয়া বান্ধা, গোল্লাছুট, ইত্যাদি।]<br>[নমুনা কার্ড দেখান]<br>***নির্দেশনাঃ***<br>*তথ্যপ্রদানকারীকে শুধুমাত্র কর্মস্থলের 'ভারী কাজগুলো/ অবসর সময়ে ভারী কাজের কথা' সম্পর্কে চিন্তা করতে বলুন। ঐকাজগুলোই অতিমাত্রার ভারী কাজ হিসেবে গণ্য হবে যার ফলে শ্বাস-প্রশ্বাসের ও হৃদস্পন্দনের হার অতিমাত্রায় বৃদ্ধি পায়।*	1 = হ্যাঁ<br>2 = না<br>(যদি না হয়, তাহলে ER_311 এ যান)		
309	ER_309	আপনি দৈনন্দিন কাজের অংশ হিসেবে সপ্তাহে কয়দিন অতিমাত্রার ভারী কাজ / অতিমাত্রার খেলাধুলা, শরীরচর্চা অথবা বিনোদন মূলক কাজ করেন?<br>***নির্দেশনাঃ***<br>*সাধারন একটি সপ্তাহ হচ্ছে উত্তর দাতার একটি স্বাভাবিক সপ্তাহে যে কাজ করে। বৈধ উত্তর সীমা হচ্ছে 1-7 দিন।*	|___|___| দিন<br>77 = জানিনা<br>[জানিনা হলে ER_311 এ যান]		
310	ER_310	সাধারনত: আপনি দিনে কত সময় ধরে অতিমাত্রার ভারী কাজ/ অতিমাত্রার খেলাধুলা, শরীরচর্চা অথবা বিনোদন মূলক কাজ করেন?<br>***নির্দেশনাঃ***<br>*উত্তর দাতাকে তার কোন একটি দিনের কথা (যা সহজেই মনে আসে) চিন্তা করতে বলুন যে দিন তিনি কর্মক্ষেত্রে ভারী কাজে নিযুক্ত ছিলেন/যে দিন তিনি অবসর সময়ে ভারী শারীরিক পরিশ্রম করেছিলেন। উত্তর দাতা ঐ সকল ভারী কাজগুলোকেই মনে করবেন যেগুলো একটানা 10 মি বা তার অধিক সময় ধরে করা হয়েছে। অধিক বা অস্বাভাবিক (4 ঘন্টার অধিক) উত্তরগুলো যাচাই করুন।*	___________<br>মিনিট		
**এখন আমি আপনার করা মাঝারি মাত্রার ভারী কাজ সম্পর্কে জানতে চাইবো।**					
দৈনন্দিন কাজের/পেশাগত কাজের বাইরে করা মাঝারী মাত্রার কাজ					

নম্বর	সূচক	প্রশ্ন	উত্তর	কোড	নির্দেশনা
311	ER_311	আপনাকে কি দৈনন্দিন কাজের/পেশাগত কাজের অংশ হিসেবে শ্বাসপ্রশ্বাস ও হৃদস্পন্দন সামান্য বেড়ে যায় এমন কোন মাঝারি মাত্রার কাজ/ মাঝারী মাত্রার খেলাধুলা, শরীরচর্চা অথবা বিনোদন মূলক কাজ একনাগাড়ে কমপক্ষে 10 মিনিট ধরে করতে হয়? যেমন, কাপড় ধোয়া, হালকা কিছু তোলা, ঝাড়ু দেওয়া, জানালা পরিষ্কার করা, রেদা বা কুড়ুনি দিয়ে চাঁছার কাজ করা, মেঝে ঝাড়ু দেওয়া, মোছা বা পরিষ্কার করার কাজ ইত্যাদি/ দ্রুত হাঁটা, ট্রেড মিলে হাঁটা, সাইকেল চালনা, সাঁতার কাটা, ভলিবল, জগিং। [নমুনা কার্ড দেখান]<br>***নির্দেশনাঃ***<br>*উত্তর দাতা কে শুধুমাত্র কর্মস্থলের / অবসর সময়ে মাঝারিমাত্রার কাজগুলো' সম্পর্কে চিন্তা করতে বলুন। ঐ কাজগুলোই মাঝারি মাত্রার কাজ হিসেবে গণ্য হবে যার ফলে শ্বাস-প্রশ্বাসের ও হৃদস্পন্দনের হার সামান্য বৃদ্ধি পায়।*	1 = হ্যাঁ<br>2 = না<br>(যদি না হয়, তাহলে ER_314 এ যান)		
312	ER_312	আপনি দৈনন্দিন কাজের অংশ হিসেবে সপ্তাহে কয়দিন মাঝারি মাত্রার কাজ/ মাঝারি মাত্রার খেলাধুলা, শরীরচর্চা অথবা বিনোদন মূলক কাজ করেন?<br>***নির্দেশনাঃ***<br>*সাধারন একটি সপ্তাহ হচ্ছে উত্তর দাতার একটি স্বাভাবিক সপ্তাহে যে কাজ করে। বৈধ উত্তরসীমা হচ্ছে 1-7 দিন।*	|___|___| দিন<br>77 = জানিনা<br>[জানিনা হলে ER_314 এ যান]		
313	ER_313	সাধারনত আপনি দিনে কত সময় ধরে মাঝারিমাত্রার কাজ/ মাঝারি মাত্রার খেলাধুলা, শরীরচর্চা অথবা বিনোদন মূলক কাজ করেন?<br>নির্দেশনাঃ<br>উত্তর দাতাকে তার কোন একটি দিনের কথা (যা সহজেই মনে আসে) চিন্তা করতে বলুন যে দিন তিনি কর্মক্ষেত্রে মাঝারিমাত্রার কাজে নিযুক্ত ছিলেন/ যে দিন তিনি অবসর সময়ে মাঝারি মাত্রার কাজে নিযুক্ত ছিলেন। উত্তরদাতা ঐ সকল মাঝারিমাত্রার কাজগুলোকে আমলে আনবেন যেগুলো একটানা 10মি বা তার অধিক সময় ধরে করা হয়েছে। অধিক/অস্বাভাবিক (4 ঘন্টার অধিক) উত্তরগুলো যাচাই করুন।	______ মিনিট		
**অবসর সময়ের কাজের ধরন**					
**পরবর্তী প্রশ্নগুলো আপনার বসে বা হেলান দিয়ে কাটানো সময় সম্পর্কিত, যা কর্মস্থলে, বাড়িতে, এক জায়গা থেকে অন্য জায়গায় গাড়ী, বাস বা ট্রেনে করে যাতায়াত অথবা বন্ধুদের সাথে আড্ডায়, পড়াশোনা, কার্ড খেলা অথবা টেলিভিশন দেখার ক্ষেত্রে প্রযোজ্য। তবে এখানে ঘুমিয়ে কাটানো সময় অন্তর্ভূক্ত হবে না।**					
314	ER_314	সাধারণত: দিনে কতটুকু সময় আপনি বসে/ হেলান দিয়ে অতিবাহিত করেন?<br>***নির্দেশনাঃ***<br>*উত্তরদাতাকে কাজকরার সময়, অফিসে, পড়াশোনার সময়, টেলিভিশন দেখার সময়, কম্পিউটার ব্যবহারের সময়, রান্নাঘরে হাতের কাজ করার সময়, বিশ্রামের সময় কতক্ষণ বসে কাটান। এখানে উত্তরদাতার ঘুমানোর সময় বিবেচ্য হবে না।*	______ মিনিট		
315	ER_315	সাধারণত: দিনে কতটুকু সময় আপনি টেলিভিশন দেখেন?	______ মিনিট		

পরিচ্ছেদ 4: দীর্ঘস্থায়ী রোগ

অংশগ্রহণকারীর আইডি:|___|___|___|___|___|___|___|___|___|___|___|___|___|___|___|___|

নম্বর	সূচক	প্রশ্ন	উত্তর	কোড	নির্দেশনা
401	ECD_401	কোন স্বাস্থ্যকর্মী কি আপনাকে কখনও বলেছেন যে, আপনার উচ্চরক্তচাপ বা হাইপারটেনশন আছে (গর্ভবতী থাকার অবস্থায় হলে এবং পরবর্তীতে ভালো হয়ে গেলে গণ্য হবেনা)?	1 = হ্যাঁ 2 = না		
402	ECD_402	কোন স্বাস্থ্যকর্মী কি আপনাকে কখনও বলেছেন যে, আপনার হার্টের অসুখ আছে?	1 = হ্যাঁ 2 = না		
403	ECD_403	কোন স্বাস্থ্যকর্মী কি আপনাকে কখনও বলেছেন যে, আপনার এ্যজমা/হাঁপানী/ব্রংকাইটিস বা শ্বাস কষ্টের রোগ আছে?	1 = হ্যাঁ 2 = না		
404	ECD_404	কোন স্বাস্থ্যকর্মী কি আপনাকে কখনও বলেছেন যে, আপনার কিডনী বা বৃক্কের রোগ আছে?	1 = হ্যাঁ 2 = না		
405	ECD_405	কোন স্বাস্থ্যকর্মী কি আপনাকে কখনও বলেছেন যে, আপনার ডায়াবেটিস আছে?	1 = হ্যাঁ 2 = না		
406	ECD_406	কোন স্বাস্থ্যকর্মী কি আপনাকে কখনও বলেছেন যে, আপনার স্ট্রোক হয়েছে?	1 = হ্যাঁ 2 = না		
407	ECD_407	কোন স্বাস্থ্যকর্মী কি আপনাকে কখনও বলেছেন যে, আপনার কোন ধরনের ক্যান্সার আছে?	1 = হ্যাঁ 2 = না		
408	ECD_408	কোন স্বাস্থ্যকর্মী কি আপনাকে কখনও বলেছেন যে, আপনার কোন মানসিক সমস্যা/রোগ আছে?	1 = হ্যাঁ 2 = না		
409	ECD_409	কোন স্বাস্থ্যকর্মী কি আপনাকে কখনও বলেছেন যে, আপনার রক্তে কোলেস্টেরল বেশি আছে?	1 = হ্যাঁ 2 = না		

পরিচ্ছেদ 5: মিনি নিউট্রিশন অ্যাসেসমেন্ট স্ক্রিনিং প্রশ্নাবলী

নম্বর	সূচক	প্রশ্ন	উত্তর	কোড	নির্দেশনা
501	EMN_501	বিগত 3 মাস ধরে ক্ষুদা মন্দা, হজমের সমস্যা, চিবানো বা গিলতে অসুবিধার কারনে আপনার খাবার গ্রহণ কি পরিমান কমেছে?	0 = অত্যাধিক পরিমানে কমেছে<br>1 = মাঝারী পরিমানে কমেছে<br>2 = খাবার গ্রহণ কমেনি		
502	EMN_502	বিগত 3 মাসে আপনার কি পরিমান ওজন কমেছে?	0 = ওজন 3 কেজির বেশী কমেছে (6.6 পাউন্ডস)<br>1 = জানেন না<br>2 = 1 থেকে 3 কেজির মধ্যে ওজন কমেছে (2.2 এবং 6.6 পাউন্ডস)<br>3 = ওজন কমেনি		
503	EMN_503	আপনি আপনার বর্তমান চলাফেরা কিভাবে মূল্যায়ন করেন?	0 = বিছানা বা চেয়ারে সীমাবদ্ধ থাকেন<br>1 = বিছানা/চেয়ার থেকে উঠতে সক্ষম, কিন্তু বাইরে যেতে পারেন না<br>2 = বাহিরে বের হতে পারেন		
504	EMN_504	বিগত 3 মাসে আপনি কি কোনো মানসিক চাপ বা তীব্র কোনো রোগে ভুগেছেন?	0 = হ্যাঁ<br>2 = না		
505	EMN_505	আপনি আপনার স্নায়বিক ও মানসিক সমস্যা কিভাবে মূল্যায়ন করেন?	0 = অত্যাধিক ভুলে যাওয়া বা বিষন্নতা<br>1 = কিছুটা ভুলে যাওয়া<br>2 = কোনো মানসিক সমস্যা নেই		

সেকশন 6ঃ জীবনযাপনের মান সংক্রান্ত প্রশ্ন (QoL)

নম্বর	সূচক	প্রশ্ন	উত্তর	কোড	নির্দেশনা
601	EQL_601	চলাফেরা	1 = আমার হাঁটাচলা করতে কোন সমস্যা হয় না<br>2 = আমার হাঁটাচলা করতে সামান্য সমস্যা হয়<br>3 = আমার হাঁটাচলা করতে মাঝারী মাত্রার সমস্যা হয়<br>4 = আমার হাঁটাচলা করতে খুব সমস্যা হয়<br>5 = আমি হাঁটাচলা করতে পারি না		
602	EQL_602	নিজের যত্ন	1 = আমার গোছল করতে অথবা জামা-কাপড় পড়তে কোন সমস্যা হয় না<br>2 = আমার গোছল করতে অথবা জামা-কাপড় পড়তে সামান্য সমস্যা হয়<br>3 = আমার গোছল করতে অথবা জামা-কাপড় পড়তে মাঝারী মাত্রার সমস্যা হয়<br>4 = আমার গোছল করতে অথবা জামা-কাপড় পড়তে খুব সমস্যা হয়<br>5 = আমি নিজে গোছল করতে অথবা জামা-কাপড় পড়তে পারি না		
603	EQL_603	স্বাভাবিক কাজকর্ম যেমন: পড়াশোনা, ঘরের কাজ, পরিবারের সাথে সময় কাটানো, অবসর সময় কাটানো।	1 = আমার স্বাভাবিক কাজ করতে কোন সমস্যা হয় না<br>2 = আমার স্বাভাবিক কাজ করতে সামান্য সমস্যা হয়<br>3 = আমার স্বাভাবিক কাজ করতে মাঝারী মাত্রার সমস্যা হয়<br>4 = আমার স্বাভাবিক কাজ করতে খুব সমস্যা হয়<br>5 = আমি স্বাভাবিক কাজ করতে পারি না		
604	EQL_604	ব্যথা/অস্বস্তি	1 = আমার কোন ব্যথা বা অস্বস্তি নেই<br>2 = আমার সামান্য ব্যথা বা অস্বস্তি আছে<br>3 = আমার মাঝারী মাত্রার ব্যথা বা অস্বস্তি আছে<br>4 = আমার বেশ ব্যথা বা অস্বস্তি আছে<br>5 = আমার খুব বেশী ব্যথা বা অস্বস্তি আছে		
605	EQL_605	দুঃশ্চিন্তা/হতাশা	1 = আমি চিন্তিত বা হতাশ নই<br>2 = আমি সামান্য চিন্তিত বা হতাশ<br>3 = আমি মাঝারী মাত্রায় চিন্তিত বা হতাশ<br>4 = আমি বেশ চিন্তিত বা হতাশ<br>5 = আমি খুব বেশী চিন্তিত বা হতাশ		
আমরা এখন আপনার স্বাস্থ্য আজ কতটা ভালো বা খারাপ সে সম্পর্কে জানতে চাই। এটা একটা স্কেল যাতে 0 থেকে 100 পর্যন্ত নম্বর দেয়া আছে। 100 নম্বরের মানে হচ্ছে আপনার মতে সবচেয়ে ভালো স্বাস্থ্য। 0 নম্বরের মানে হচ্ছে আপনার মতে সবচেয়ে খারাপ স্বাস্থ্য। আপনার মতে আপনার স্বাস্থ্য আজ কেমন তা জানানোর জন্য এই স্কেলের উপর X চিহ্ন দিন।<br>*[ইকিউ ভিএএস স্কেলটি সাক্ষাৎকারপ্রদানকারীকে দিন এবং স্কেলের উপর X চিহ্ন দিতে বলুন।]*					
605	EQL_605	স্বাস্থ্য স্কোর	|___|___|___|		

অংশগ্রহণকারীর আইডি:|___|___|___|___|___|___|___|___|___|___|___|___|___|___|___|___|

পরিচ্ছেদ 7: রক্তচাপ পরিমাপ

নম্বর	সূচক	প্রশ্ন	উত্তর			কোড	নির্দেশনা
701	EBP_701	রক্তচাপ পরিমাপ নেয়া শুরুর সময়	|____|____| : |____|____| ঘন্টা : মিনিট			24 ঘণ্টার ফরম্যাট ব্যবহার করুন	
**পরিমাপ নং-1** (পরিমাপ নেয়ার পূর্বে নিশ্চিত হোন যেন উত্তরদাতা কমপক্ষে 15 মিনিট বিশ্রামে থাকেন)							
নম্বর	সূচক	পরিমাপের নাম	Systolic	Diastolic	পরিমাপের সময়		
702	EBP_702 A/B/C	রক্তচাপ (mm of Hg)	A.|___|___|___|	B.|___|___|___|	C.|____|____|:|____|____| ঘন্টা : মিনিট		
**পরিমাপ নং-2** (দুইটি পরিমাপের মধ্যে 3 মিনিট বিরতি দিন)							
703	EBP_703 A/B/C	রক্তচাপ (mm of Hg)	Systolic<br>A.|___|___|___|	Diastolic<br>B.|___|___|___|	পরিমাপের সময়<br>C.|____|____|:|____|____| ঘন্টা : মিনিট		
**পরিমাপ-3** (যদি যে কোনটির ক্ষেত্রে 1ম ও 2য় পরিমাপের মধ্যে পার্থক্য 10 এর বেশি হয়, তাহলে ৩য় বার রক্তচাপ নিন)							
704	EBP_704 A/B/C	রক্তচাপ (mm of Hg)	Systolic<br>A.|___|___|___|	Diastolic<br>B.|___|___|___|	পরিমাপের সময়<br>C.|____|____|:|____|____| ঘন্টা : মিনিট		
705	EBP_705	রক্তচাপ পরিমাপ যন্ত্রের আইডি	|____|____|				
706	EBP_706	রক্তচাপ পরিমাপ গ্রহণকারীর নাম ও কোড	|____|____|				আপনার পুরা নাম এবং কোড লিখুন
707	EBP_707	রক্তচাপ পরিমাপ গ্রহণের শেষের সময়	|____|____| : |____|____| ঘন্টা : মিনিট				24 ঘণ্টার ফরম্যাট ব্যবহার করুন
**[যে কোন পরিমাপের সময়, Systolic BP ≥140 mm of Hg, Diastolic BP ≥90 mm of Hg-এর যে কোন একটি হলে অংশগ্রহণকারীকে নির্দিষ্ট স্বাস্থ্য কেন্দ্রে রেফার করুন ।]**							

রেফারেল (Referral)

নম্বর	সূচক	প্রশ্ন	উত্তর	কোড	নির্দেশনা
708	EBP_708	রেফার করা হয়েছে কি?	1 = হ্যাঁ 2 = না		উত্তর না হলে, পরিচ্ছেদ 8 এ যান
709	EBP_709	কোথায় রেফার করা হয়েছে	1 = উপজেলা স্বাস্থ্য কমপ্লেক্স<br>2 = জেলা হাসপাতাল<br>3 = মেডিকেল কলেজ হাসপাতাল<br>4 = ডাক্তারের চেম্বার<br>99 = অন্যান্য (নির্দিষ্ট করুন)______		

অংশগ্রহণকারীর আইডি:|___|___|___|___|___|___|___|___|___|___|___|___|___|___|___|___|

খাদ্য নিরাপত্তা ও পুষ্টি বিষয়ক সার্ভিলেন্স

পরিচ্ছেদ 8: পরিমাপ

নম্বর	সূচক	প্রশ্ন	উত্তর	কোড	নির্দেশনা
801	EA_801	পরিমাপ নেয়া শুরুর সময়	|____|____| : |____|____| ঘন্টা : মিনিট		24 ঘন্টার ফরম্যাট ব্যবহার করুন
802	EA_802	ওজন পরিমাপক যন্ত্রের আইডি	|____|____|		
803	EA_803	দৈর্ঘ্য পরিমাপক যন্ত্রের আইডি	|____|____|		
804	EA_804	ওজন নেয়ার সময় গায়ের কাপড়ের ধরন	1 = হালকা কাপড়<br>2 = একটু ভারী কাপড়<br>3 = ভারী কাপড়		
805	EA_805	পরিমাপ গ্রহণকারীর নাম			
806	EA_806	পরিমাপ গ্রহণকারীর কোড	|____|____|		

নম্বর	সূচক	পরিমাপের নাম	পরিমাপ 1	পরিমাপ 2	পরিমাপ 3
807	EA_807 A/B/C	উচ্চতা (Cm)	A.|____|____|____|.|____|	B.|____|____|____|.|____|	C.|____|____|____|.|____| যদি A ও B এর মধ্যে পার্থক্য 0.5cm এর বেশি হয়, তাহলে ৩য় বার মাপুন
808	EA_808 A/B/C	ওজন (Kg)	A.|____|____|____|.|____|	B.|____|____|____|.|____|	C.|____|____|____|. |____| যদি A ও B এর মধ্যে পার্থক্য 0.1Kg এর বেশি হয়, তাহদলে ৩য় ওজন নিন
809	EA_809 A/B/C	চর্বি (%)	A.|____|____|____|.|____|	B.|____|____|____|.|____|	C.|____|____|____|.|____| যদি A ও B এর মধ্যে পার্থক্য 0.5cm এর বেশি হয়, তাহলে ৩য় বার মাপুন
810	EA_810 A/B/C	পানি (%)	A.|____|____|____|.|____|	B.|__ _|____|____|.|____|	C.|____|____|____|. |____| যদি A ও B এর মধ্যে পার্থক্য 0.1Kg এর বেশি হয়, তাহদলে ৩য় ওজন নিন
811	EA_811 A/B/C	বাম বাহুর পরিধি (Cm)	A.|____|____|.|____|	B.|____|____|.|____|	C. |____|____|.|____| যদি A ও B এর মধ্যে পার্থক্য 0.5cm এর বেশি হয়, তাহলে ৩য় বার মাপুন
812	EA_812 A/B/C	পায়ের গোছার পরিধি (Cm)	A.|____|____|.|____|	B.|____|____|.|____|	C.|____|____|.|____| যদি A ও B এর মধ্যে পার্থক্য 0.5cm এর বেশি হয়, তাহলে ৩য় বার মাপুন
813	EA_813 A/B/C	কোমরের পরিধি (Cm)	A.|____|____|.|____|	B.|____|____|.|____|	C.|____|____|.|____| যদি A ও B এর মধ্যে পার্থক্য 0.5cm এর বেশি হয়, তাহলে ৩য় বার মাপুন
814	EA_814	পরিমাপ সম্পর্কে তথ্য সংগ্রহকারীর মন্তব্য			
815	EA_815	পরিমাপ গ্রহণের শেষের সময়	|____|____| : |____|____| ঘন্টা : মিনিট		24 ঘন্টার ফরম্যাট ব্যবহার করুন

মডিউল 9: মডিউল এর পরিকল্পনা

নম্বর	সূচক	প্রশ্ন	উত্তর		কোড	নির্দেশনা
101	PM_101	কোন কোন মডিউল সম্পন্ন করার কথা ছিল?	A= মডিউল 2 : খানার তথ্য	1 = হ্যাঁ<br>2 = না		
			B= মডিউল 3 : শিশুর তথ্য	1 = হ্যাঁ<br>2 = না		
			C= মডিউল 4 : কিশোরের তথ্য	1 = হ্যাঁ<br>2 = না		
			D= মডিউল 5 : কিশোরীর তথ্য	1 = হ্যাঁ<br>2 = না		
			E= মডিউল 6 : প্রাপ্তবয়স্ক পুরুষের তথ্য	1 = হ্যাঁ<br>2 = না		
			F= মডিউল 7 : প্রাপ্তবয়স্ক মহিলার তথ্য	1 = হ্যাঁ<br>2 = না		
			G= মডিউল 8 : বৃদ্ধ/বৃদ্ধার তথ্য	1 = হ্যাঁ<br>2 = না		
102	PM_102	সম্পন্ন মডিউল	A= মডিউল 2 : খানার তথ্য	1 = হ্যাঁ<br>2 = না		
			B= মডিউল 3 : শিশু তথ্য	1 = হ্যাঁ<br>2 = না		
			C= মডিউল 4 : কিশোর তথ্য	1 = হ্যাঁ<br>2 = না		
			D= মডিউল 5 : কিশোরীর তথ্য	1 = হ্যাঁ<br>2 = না		
			E= মডিউল 6 : প্রাপ্তবয়স্ক পুরুষের তথ্য	1 = হ্যাঁ<br>2 = না		
			F= মডিউল 7 : প্রাপ্তবয়স্ক মহিলার তথ্য	1 = হ্যাঁ<br>2 = না		
			G = মডিউল 8 : বৃদ্ধ/বৃদ্ধার তথ্য	1 = হ্যাঁ<br>2 = না		
103	PM_103	পূনরায় সাক্ষাৎকার নেয়ার প্রয়োজন আছে কি?	1 = হ্যাঁ 2 = না			
104	PM_104	পূনরায় সাক্ষাৎকার নেয়ার তারিখ?	|___|___|/|___|___|/20|___|___| দিন / মাস / বছর			
105	PM_105	সাক্ষাৎকার গ্রহণের শেষের তারিখ	|___|___|/|___|___|/20|___|___| দিন / মাস / বছর			
106	PM_106	সাক্ষাৎকার গ্রহণের শেষের সময়	|____|____| : |____|____| ঘন্টা : মিনিট			24 ঘণ্টার ফরম্যাট ব্যবহার করুন
107	PM_107	সাক্ষাৎকার গ্রহণকারীর মন্তব্য				

Thank you for your participation and cooperation
