# Supplementary material for: Ten-years cardiovascular risk among Bangladeshi population using non-laboratory-based risk chart of the World Health Organization: Findings from a nationally representative survey
Source: PLoS One. 2021 May 26;16(5):e0251967. doi: 10.1371/journal.pone.0251967 (PMC8153482; doi:10.1371/journal.pone.0251967)
Supplement: Attachment — Submitted filename: revision bangladeshi.pdf [file pone.0251967.s006.pdf]

Ten-years cardiovascular risk among the Bangladeshi population using non-laboratory-based risk chart of the World Health Organization: findings from a nationally representative survey

In the present paper, Abu Abdullah Mohammad Hanif and Colleagues aimed to investigate the role of a non-laboratory based cardiovascular disease (CVD) risk chart, recently developed by the World Health Organization (WHO)[1], in assessing the prevalence of ten years CVD burden among Bangladeshi population aged 40-74 years. Parameters used in the risk algorithm were age, sex, smoking status, body mass index and systolic blood pressure. They obtain six groups based on the calculated risk score: no risk (<5%), low risk (5% to <10%), moderate risk (10% to <20%), high risk (20% to <30%) and very high risk (≥30%). Then Authors listed potential factors associated with elevated CVD risk (defined as CVD risk ≥10%) among the subjects studied, based on literature review and considering data availability from this survey. Sociodemographic variables included place of residence (rural, non-slum urban, and slum), education, household income, marital status and religion; among the behavioral variables were physical activity, sedentary time, fruits and vegetable consumption, and smokeless tobacco consumption status.

So the study deals with an interesting issue concerning resource optimization topic; in low income countries a non-laboratory based chart could help the health care workers in screening, primary management counseling and referral of patients at higher risk of cardiovascular events to further evaluation. On the other hand, there are some methodological limitations that could weak the strength of these findings, in particular data accuracy and poor method description.

Major points

- In the method session about sample size and sampling techniques, Authors have selected patients according to origin area: rural, urban and slum zone. For each cluster research assistants listed households based on age groups; could author clarify age range and better defined it (year of age or age range as listed in table 1?). Moreover, it could be interesting screening patients also by gender in order to have balanced groups of the same age

- In the method session authors recorded "self-reported diabetes" if subjects had declared this comorbidity; it could be useful regarding the elevated risk of CVD in this cluster of patients also checking old blood chemistry exams, when available.

- Method session need further explanation; authors refer to a non-laboratory based CVD risk chart, recently developed by the World Health Organization[1]; but in the

aforementioned model were included participants without a known baseline history of cardiovascular disease; this aspect is not clarified in the study. Moreover, there are a lot of relevant parameters that are associated to an elevated risk of CVD, such as family history, chronic renal disease, carotid atherosclerosis, that have not been reported. All these variables are easily obtainable through an interview and have a significantly weight in the selection of clusters of subjects at higher risk of adverse events.

- In the “explanatory variables” session Authors defined outcome variable as elevated CVD risk if the total CVD risk score was ≥10% and CVD risk if the risk score was <10%; how they establish this cut-off ? This choice needs further explanation.

- In the Statistical analysis section Authors carry out bivariate logistic regression analysis, but they don’t itemize the variables sorted. Why did Authors employ a bivariable approach rather than a two-step univariable-multivariable logistic regression analysis?

- In the result section Authors state that they extracted data of 7,757 males and females aged from 40 to 74 years old as the WHO CVD risk chart is only applicable for this age groups, please clarify this content, since in the referral model[1] were included participants aged 40-80 years old.

- An explicative graphic of the study design could be helpful in evaluating patient screening from the initial 17,323 households to the effective 7,757 subjects studied.

- In Table 1, are p-values referred to multivariable comparisons? Probably in this situation a sub-analysis for significantly different variables should be performed.

- Could authors explain why in table 2 they considered only sociodemographic characteristics?

- All results are itemized in four tables; this presentation method could make occult some relevant data; a figure about the prevalence of CVD in the different risk categories could focus on the topic of the paper.

- Could authors explain what are the multivariable analysis adjusted for and which is the covariate?

- In the discussion session Authors state “this analysis revealed that without even any laboratory procedures, the primary health care worker would be able to assess the CVD risk of the population and initiate management/referral procedure following national protocols for the CVD risk” (lines 363-365); without follow-up data and in the absence of a comparison with a lab-based analysis (also in a subgroup of their sample size) they could not sentencing this content.

- In the discussion session the assertion: “insufficient physical activity may cause overweight, high blood pressure, and increase the level of bad cholesterol; tobacco can damage and narrow the blood vessels with harmful substances and, high blood sugar levels can damage the blood vessels, and all of these eventually cause CVDs” (lines 393-396) is too generic; please reward.

- Finally, considerations about the usefulness of a non lab-based algorithm adopted by physicians for implementing subsequent prevention and management options (line 397-399) can only be hypothesized, since follow-up data are missing.

Minor points

- Please correct line 294 where we read 189% vs. 16%

- References are to be corrected

- Please grammatically correct line 295 where probably verb is missing

- Please correct line 317: “,)”

- Please correct line 339: a bracket is missing

References

1) Kaptoge S, Pennells L, Bacquer DD, Cooney MT, Kavousi M, Stevens G, et al. World Health Organization cardiovascular disease risk charts: revised models to estimate risk in 21 global regions. The Lancet Global Health. 2019;7(10):e1332-e45
