# Supplementary material for: Ten-years cardiovascular risk among Bangladeshi population using non-laboratory-based risk chart of the World Health Organization: Findings from a nationally representative survey
Source: PLoS One. 2021 May 26;16(5):e0251967. doi: 10.1371/journal.pone.0251967 (PMC8153482; doi:10.1371/journal.pone.0251967)
Supplement: Attachment — Submitted filename: Response to reviewers.docx [file pone.0251967.s008.docx]

PONE-D-20-10140R1
Ten-years cardiovascular risk among the Bangladeshi population using non-laboratory-based risk chart of the World Health Organization: findings from a nationally representative survey

We are thankful to the Editor and the reviewers for providing feedback on the revised manuscript. Below are the responses from the authors of this manuscript:

Journal Requirements:

Please review your reference list to ensure that it is complete and correct. If you have cited papers that have been retracted, please include the rationale for doing so in the manuscript text, or remove these references and replace them with relevant current references. Any changes to the reference list should be mentioned in the rebuttal letter that accompanies your revised manuscript. If you need to cite a retracted article, indicate the article’s retracted status in the References list and also include a citation and full reference for the retraction notice.

Response from author: We checked the reference list and corrected the incompleteness of some references we have found but as we have used used reference manager to correct the references, the changes do not reflect as tracked change. However, we did not include any new reference. We also checked each of the articles cited in our manuscript, and we did not find any retracted article.

Reviewer #1: The Authors have reviewed extensively the paper and answered appropriately to the comments made by the Reviewers.
I have still two minor issues: the p values in the tables should be mentioned and not eliminated at all, now that the Tables have been clarified for the reader.

Response from author: Noted with thanks. We reinstated the p-value of the Chi square test conducted between sex and the listed characteristics of the study participants in table 1 and between risk categoris and the listed characteristics of the study participants in table 2.

Also, in the Methods section, an effect size of 1.6 is mentioned, which is very wide. The sample size calculated might therefore be misleading. Please clarify.

Response from author: Noted with thanks.

1.6 mentioned in the manuscript is not effect size, it is design effect. We are sorry for the confusion. Below is the clarification. The design effect (DEF), we calculated using the following formula:

Design Effect = 1 + (n-1)*icc

In our study, the cluster sample size was 62, and we the intra-cluster correlation was 0.01 [1].

Reviewer #2: The authors have adequately addressed my concerns. The study deals with an interesting issue regarding resource optimization topic. A non-laboratory based chart could help health care workers in screening, primary management counseling and referral of patients at higher risk cardiovascular events to further evaluation.

Response from author: Thank you so much!

References:

1.	Singh J, Liddy C, Hogg W, Taljaard M. Intracluster correlation coefficients for sample size calculations related to cardiovascular disease prevention and management in primary care practices. BMC Res Notes. 2015;8: 89. doi:10.1186/s13104-015-1042-y
